# Supplementary material for: Metal-free photocatalytic cross-electrophile coupling enables C1 homologation and alkylation of carboxylic acids with aldehydes
Source: Nat Commun. 2024 Feb 19;15:1509. doi: 10.1038/s41467-024-45804-z (PMC10876646; doi:10.1038/s41467-024-45804-z)
Supplement: Supplementary file 1 — Supplementary Information [file 41467_2024_45804_MOESM1_ESM.pdf]

Supplementary Information

Metal-free Photocatalytic Cross-Electrophile Coupling enables C1 Homologation and Alkylation of Carboxylic Acids with Aldehydes

Stefano Bonciolini,[1,†] Antonio Pulcinella,[1,†] Matteo Leone,[1,2] Debora Schiroli,[1,3] Adrián Luguera Ruiz,[1,4] Andrea Sorato,[1] Maryne A. J. Dubois,[5] Ranganath Gopalakrishnan,[5] Geraldine Masson,[2] Nicola Della Ca',[3] Stefano Protti,[4] Maurizio Fagnoni,[4] Eli Zysman-Colman,[6] Magnus Johansson,[5] and Timothy Noël[1,*]

[1] Flow Chemistry Group, Van 't Hoff Institute for Molecular Sciences (HIMS), University of Amsterdam, Science Park 904, 1098 XH Amsterdam, The Netherlands.

[2] Institut de Chimie des Substances Naturelles, CNRS, Univ. Paris-Saclay, 1 Avenue de la Terrasse, 91198 Gif-sur-Yvette Cedex, France.

[3] SynCat Lab, Department of Chemistry, Life Sciences and Environmental Sustainability, University of Parma, 43124 Parma, Italy.

[4] PhotoGreen Lab, Department of Chemistry, University of Pavia, 27100 Pavia, Italy.

[5] Medicinal Chemistry, Research and Early Development, Cardiovascular, Renal and Metabolism (CVRM), BioPharmaceuticals R&D, AstraZeneca, Gothenburg, Sweden.

[6] Organic Semiconductor Centre, EaStCHEM School of Chemistry, Purdie Building, North Haugh University of St Andrews, St Andrews, Fife, KY16 9ST UK.

[†] These authors contributed equally to this work.

* *Email:* *t.noel@uva.nl*

Table of Contents

1. General information 4

2. Supplementary Figures of Starting Materials 5

3. Synthesis of Starting Materials 7

3.1 Synthesis of 4-(trifluoromethyl)benzenesulfonohydrazide 7

3.2 Synthesis of ethyl glyoxylate-derived 4-trifluoromethylphenyl sulfonyl hydrazones **2a** 7

3.3 General procedure (**GP1**) for the preparation of 4-(trifluoromethyl)benzenesulfonyl hydrazone compounds **2b-2o** 7

3.4 General procedure (**GP2**) for the preparation of NHPI Redox Active Esters **1a-1u**, **1aa-1af** 8

4. Reaction setup 9

5. Optimization 11

5.1 Preliminary attempts of the C1 Homologation reaction 11

5.2 Optimization of the C1 Homologation Reaction 12

5.3 Optimization Alkylation Reaction 16

5.4 Sensitivity Assessment C1 Homologation 18

6. General procedure 3 (**GP3**): Photochemical radical addition to benzenesulfonyl hydrazones and subsequent fragmentation of the benzenesulfonyl hydrazide 19

7. Solid Phase Synthesis of Peptides and Purification 19

7.1 General Procedure 4 (**GP4**): Synthesis of Redox Active Esters (RAEs) on Resin 23

7.2 General Procedure 5 (**GP5**): On resin photochemical radical addition to Aryl Sulfonyl Hydrazones 2a and 2b 24

7.3 General Procedure 6 (**GP6**): Cleavage of the Alkylated Sulfonyl Hydrazide Intermediate on Resin. 25

7.4 Optimization of the on-resin photochemical alkylation of peptides 26

7.5 Late Stage Functionalization of Peptides on Solid Phase: Characterization Data 32

8. Mechanistic investigation 35

8.1 UV-Vis Characterization 35

8.2 TEMPO Radical Trapping 38

8.3 Radical Clock Experiment 39

9. Scale-up procedure for compounds **3** and **28** 40

9.1 Scale-up in Batch 40

9.2 Scale-up in Continuous Flow 41

Selected Entries of Optimization of the C1 Homologation in Flow 41

Scale-up (4 mmol) of Compound **3** in Flow 42

10. Characterization data of synthesized compound 43

10.1 Characterization of sulfonyl hydrazones **2d-2f**, **2k-2m** 43

10.2 Characterization of NHPI esters **1c**, **1e**, **1f**, **1t**, **1u**, **1ab-1af** 45

10.3 Characterization of compounds **3-26** (C1 homologation) 48

10.4 Characterization of compounds **27-58** (alkylation) 55

11. Limitation of the scope 65
12. NMR spectra of 4-(trifluoromethyl)benzenesulfonohydrazide 66
13. NMR spectra of sulfonylhydrazones **2d-2f**, **2k-2m** 69
14. NMR spectra of NHPI esters **1c**, **1e**, **1f**, **1t**, **1u**, **1ab-1af** 81
15. NMR spectra of products **3-26** (C1 homologation) 91
16. NMR spectra of products **27-58** (alkylation) 116
17. References 150

1. General information

All reagents and solvents were used as received without further purification, unless stated otherwise. Reagents and solvents were bought from Sigma Aldrich, TCI, Fluorochem and Fisher Scientific and, if applicable, kept under argon atmosphere. Technical solvents were bought from VWR International and Biosolve, and were used as received. Photocatalyst 4CzIPN and 3DPA2FBN were prepared according to a published procedure.[1,2] Aldehydes (**3a-3d**) were prepared according to a published procedure.[3,4] Disposable syringes were purchased from Laboratory Glass Specialist. Product isolation was performed manually, using silica (P60, SILICYCLE) or automatically, using Biotage® Isolation Four, with Biotage® SNAP KP-Sil 4 or 10 g flash chromatography cartridges. Polygoprep 60-50 C18 silica from Macherey-Nagel was used for reverse-phase chromatography. TLC analysis was performed using Silica on aluminum foils TLC plates (F254, Supelco Sigma-Aldrich™) with visualization under ultraviolet light (254 nm and 365 nm) or appropriate TLC staining (cerium ammonium molybdate or potassium permanganate). ^{1}H (400 MHz), ^{13}C (101 MHz), ^{19}F NMR (376 MHz) spectra were recorded unless stated otherwise at ambient temperature using a Bruker AV400 or a Bruker AV300. ^{1}H NMR spectra are reported in parts per million (ppm) downfield relative to $CDCl_3$ (7.26 ppm) and all ^{13}C NMR spectra are reported in ppm relative to $CDCl_3$ (77.16 ppm) unless stated otherwise. The following abbreviations have been adopted to describe the multiplicity: bs (broad singlet), s (singlet), d (doublet), t (triplet), q (quartet), p (pentet), h (hextet), hept (heptet), m (multiplet), dd (double doublet), td (triple doublet), tt (triplet of triplets). Coupling constants (*J*) are reported in hertz (Hz). NMR data were processed using the MestReNova 14.1.0 software package. Known products were characterized through comparison with the corresponding ^{1}H NMR and ^{13}C NMR from literature. High resolution mass spectra (HRMS) were collected on an AccuTOF LC, JMS-T100LP Mass spectrometer (JEOL, Japen. UV-Vis spectra were recorded with a double beam spectrophotometer Shimadzu UV2600 equipped with a deuterium lamp (190-350 nm), a halogen lamp (330-900 nm) and a photomultiplier (Hamamatsu R928). HPLC analysis were performed on a Shimadzu apparatus equipped with a diode array detector (DAD) and column temperature control module. Liquid chromatograph (LC-20AD); Autosampler (SIL-20A); Diode Array detector (SPD-M20A); Column oven (CTO-20AC); Degasser (DGU-$20A_5$). The names of all products were generated using the PerkinElmer ChemBioDraw Ultra v.12.0.2 software package.

For the photochemical batch experiments and scale-up (1 mmol), a 3D-printed (PLA) reactor internally coated with aluminum foil and equipped with a specific 3D-printed (PLA) lid serving as vials holder and lamp holder was used (see section 4 for details).

2. Supplementary Figures of Starting Materials

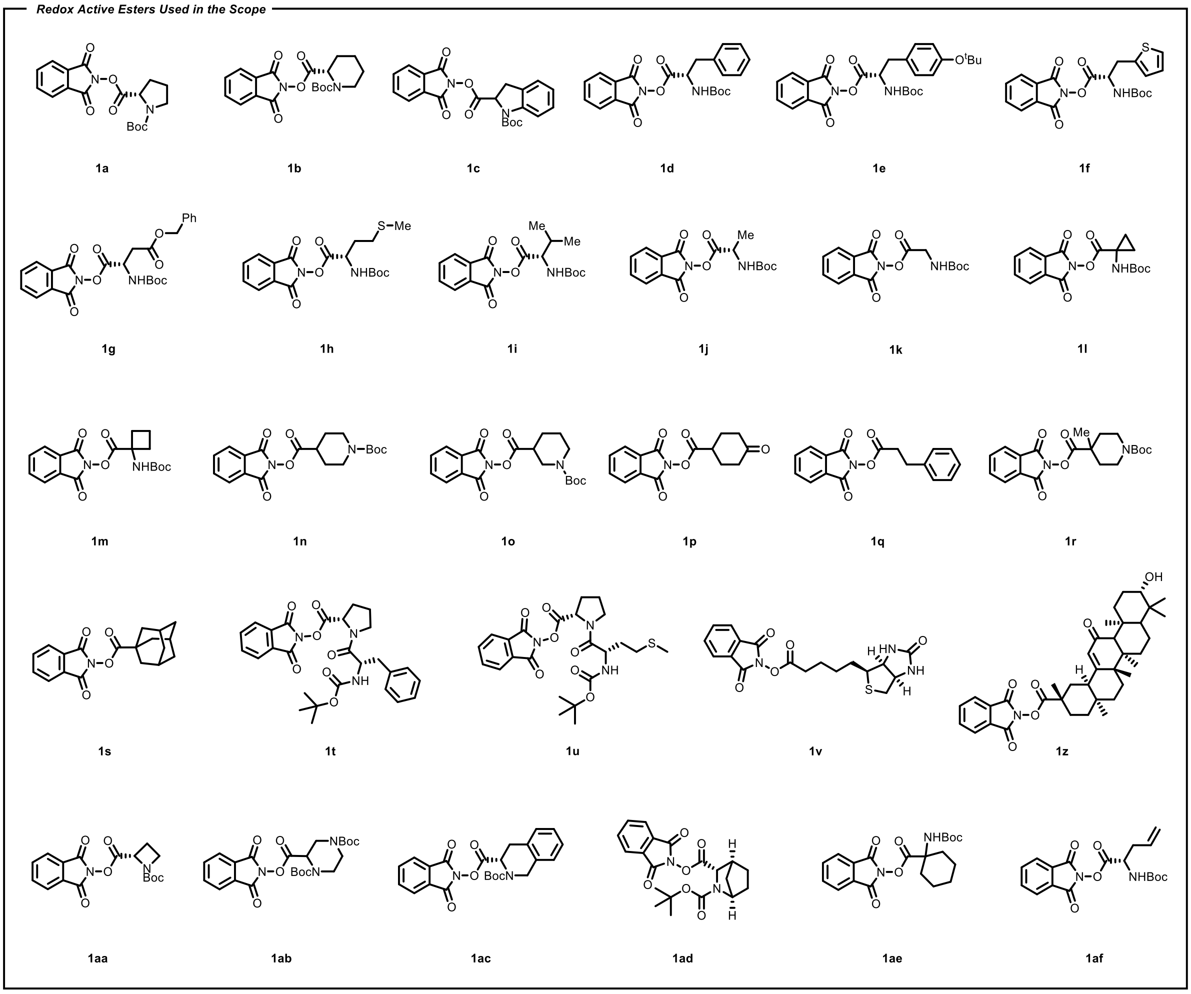

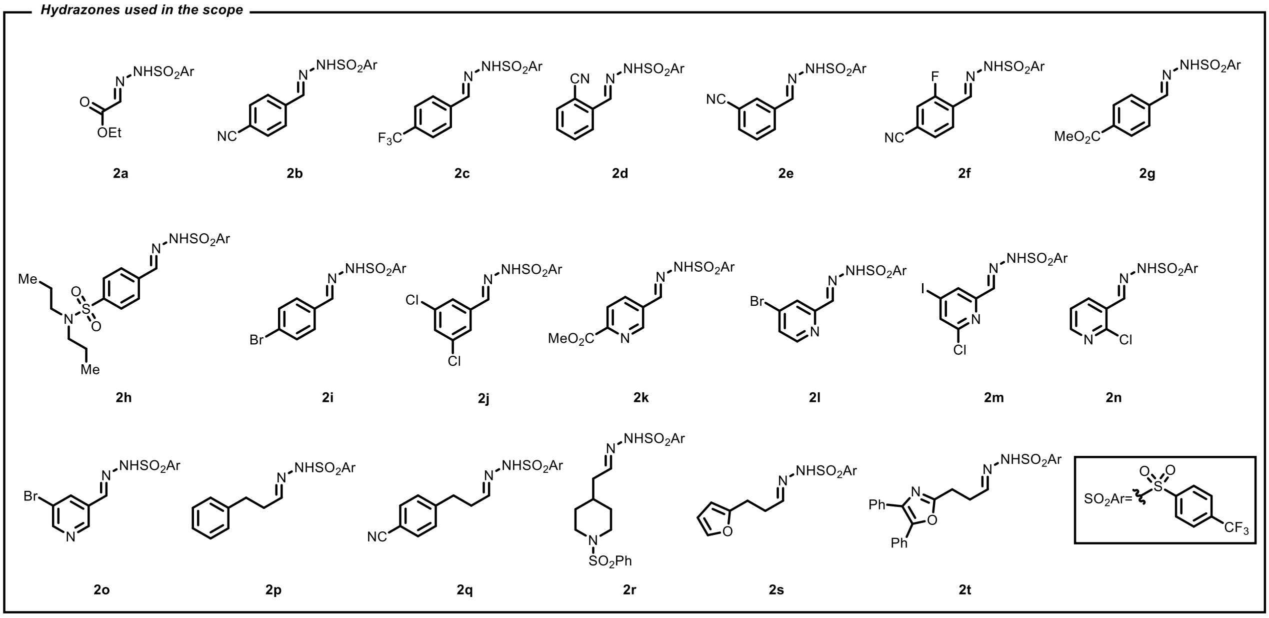

Newly Synthesized Aldehydes

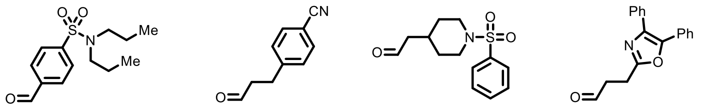

3a **3b** **3c** **3d**

3. Synthesis of Starting Materials

3.1 Synthesis of 4-(trifluoromethyl)benzenesulfonohydrazide

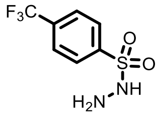

Hydrazine hydrate (108 mmol, 5.3 mL, 3 equiv.) was added dropwise to a solution of 4-(trifluoromethyl)benzenesulfonyl chloride (36 mmol, 8.81 g) in 180 mL of THF at 0 °C and stirred for 30 minutes at the same temperature. The reaction mixture was then diluted with ethyl acetate and washed five times with brine. The organic layer was dried over Na_2SO_4, filtered and the solvent was removed under reduced pressure to afford the desired compound as a white solid (8.20 g, 34.0 mmol., 95% yield).

^{1}H NMR (500 MHz, DMSO-*d*$_6$) δ 8.67 (bs, 1H), 8.04 (d, *J* = 8.3 Hz, 2H), 7.98 (d, *J* = 8.3 Hz, 2H), 4.29 (bs, 2H).
^{13}C NMR (126 MHz, DMSO *d*$_6$) δ 142.5, 132.4 (q, *J* = 32.2 Hz), 128.7, 126.2 (q, *J* = 3.8 Hz), 123.6 (q, *J* = 272.8 Hz).
^{19}F NMR (470 MHz, DMSO *d*$_6$) δ -61.77.
HRMS (ESI+) (m/z): $[M+H]^+$ calcd. for $C_7H_7F_3N_2O_2S$, 241.0259; found: 241.0269.

3.2 Synthesis of ethyl glyoxylate-derived 4-trifluoromethylphenyl sulfonyl hydrazones 2a

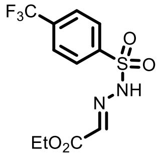

To a 0.3 M solution of 4-(trifluoromethyl)benzenesulfonohydrazide (1.20 g, 5.00 mmol, 1.0 equiv.) in dry ethanol, ethyl glyoxylate (50% soln. toluene, 1.02 g, 1.02 mL, 5.0 mmol, 1.0 equiv.) was added dropwise. The reaction mixture was stirred for 1.5 h, then the solvent was removed under vacuum. The resulting solid was washed with Pentane:Ethyl Acetate 10:1 and isolated as a white solid after filtration (1.45 g, 89% yield). The protocol can be extended to a 20 mmol scale (80% yield). Characterization data are in accordance with literature.[4]

^{1}H NMR (400 MHz, $CDCl_3$) δ 12.2 (s, 1H), 8.1 (d, *J* = 8 Hz, 2H), 7.8 (d, *J* = 8 Hz, 2H), 6.8 (s, 1H), 4.3 (q, *J* = 7 Hz, 2H), 1.32 (t, *J* = 7 Hz, 3H).
^{13}C NMR (101 MHz, $CDCl_3$) δ 161.8, 141.9, 135.5 (q, *J* = 33 Hz), 129.2, 128.6, 126.5 (q, *J* = 4 Hz), 123.2 (q, *J* = 273 Hz), 62.2, 14.0.
^{19}F NMR (282 MHz, $CDCl_3$) δ -63.28.

3.3 General procedure (GP1) for the preparation of 4-(trifluoromethyl)benzenesulfonyl hydrazone compounds 2b-2o

To a suspension of 4-(trifluoromethyl)benzenesulfonohydrazide (1.0 equiv.) in ethanol (0.8 M), the corresponding aldehyde (1.0 equiv.) was added portionwise and the reaction mixture was stirred at room temperature for 3-16 hours. In case a solid crashed out of the solution, it was filtered, washed with pentane and dried to afford the desired aryl sulfonylhydrazone. For those compounds that did not precipitate, the solvent was removed under reduced pressure and the obtained solid was washed with pentane, filtered and dried to afford the desired aryl sulfonylhydrazone. For all newly reported benzenesulfonyl hydrazones see section 10.1 for characterization data. Aliphatic Sulfonyl Hydrazones **2p-2t** were prepared in accordance to a literature procedure and used without any further purification.[5]

3.4 General procedure (GP2) for the preparation of NHPI Redox Active Esters 1a-1u, 1aa-1af

To a vigorous stirring solution of carboxylic acid (2.0-30 mmol, 1.0 equiv.), 4-dimethylaminopyridine (0.1 equiv.) and N-hydroxyphthalimide (1.0 equiv.) in CH_2Cl_2 (0.3 M), N,N′-Diisopropylcarbodiimide (1.0 equiv.) was added dropwise. Then, the reaction mixture was stirred overnight at room temperature. Upon completion, the mixture was filtered and the solvent was removed under reduced pressure. Purification via silica gel column chromatography afforded the desired product. For all newly reported redox-active esters see section 10.2 for characterization data. RAEs **1v**[6], **1z**[7] were prepared in accordance to literature procedures.

4. Reaction setup

4 vials photoreactor (UFO reactor) used at the University of Amsterdam

Four reactions were irradiating simultaneously using the photoreactor described below. A 40W Kessil PR160L-456 nm was used as LED lamp, while the temperature was maintained around 30 °C via a fan positioned under the reactor. The assembled set-up was placed behind UV-light shielding amber acrylic for all duration of the reaction.

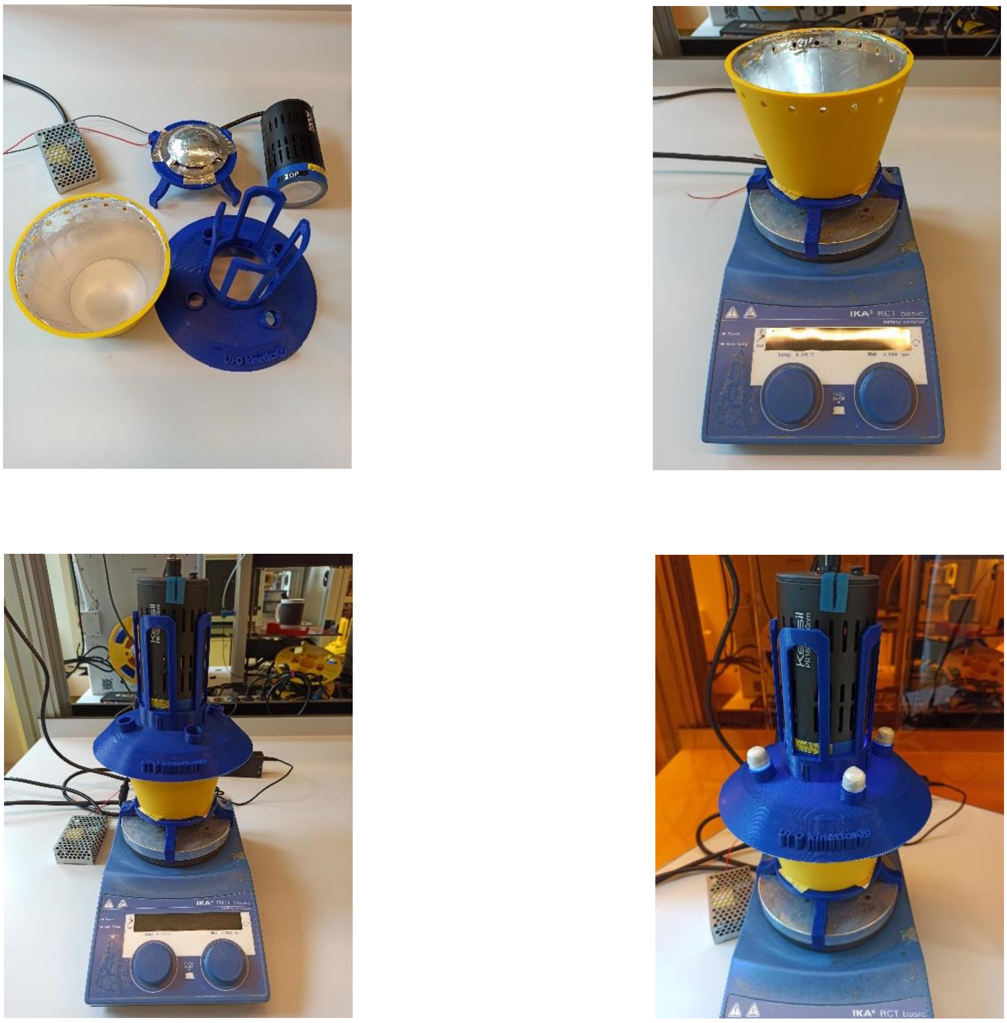

Supplementary Figure 1. Photoreactor used for the optimization and scope

4 vials photoreactor (1 mmol scale) used at the University of Amsterdam

The oven-dried 30 mL vial equipped with a stirring bar was irradiated using the photoreactor described below. A 40W Kessil PR160L-456 nm was used as LED lamp, while the temperature was maintained around 30 °C via a fan positioned under the reactor. The assembled set-up was placed behind UV-light shielding amber acrylic for all duration of the reaction. 4 vials can be irradiated simultaneously to ensure a productivity of 4 mmol scale on a per-reactor basis.

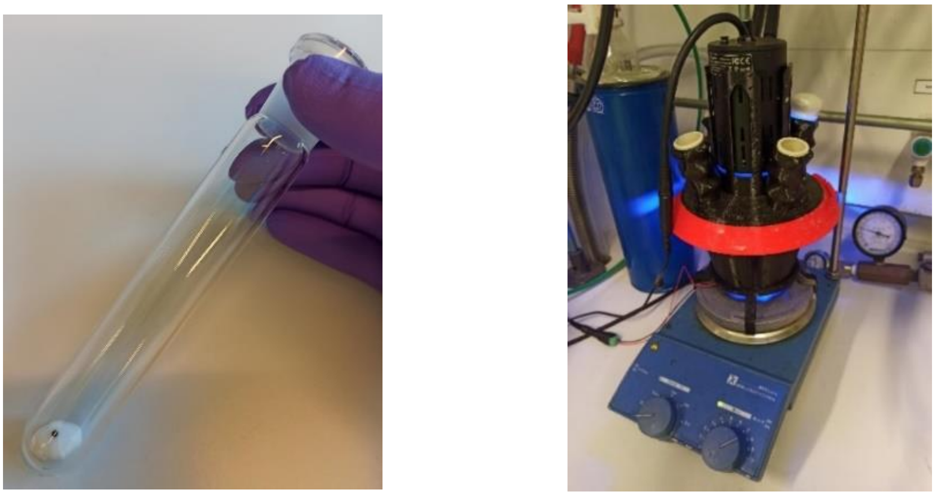

Supplementary Figure 2. Photoreactor set-up used for 1 mmol scale

6 vials photoreactor (Blue Box reactor) used in AstraZeneca, Gothenburg site (SE).

Reactions for the On Resin Photochemical LSF of peptides carried out in AstraZeneca were irradiated using the photoreactor described below. A 40W Kessil PR160L-456 nm was used as LED lamp, while the temperature was maintained below 30 °C via a fan positioned under the reactor. The assembled set-up was placed behind UV-light shielding amber acrylic for all duration of the reaction.

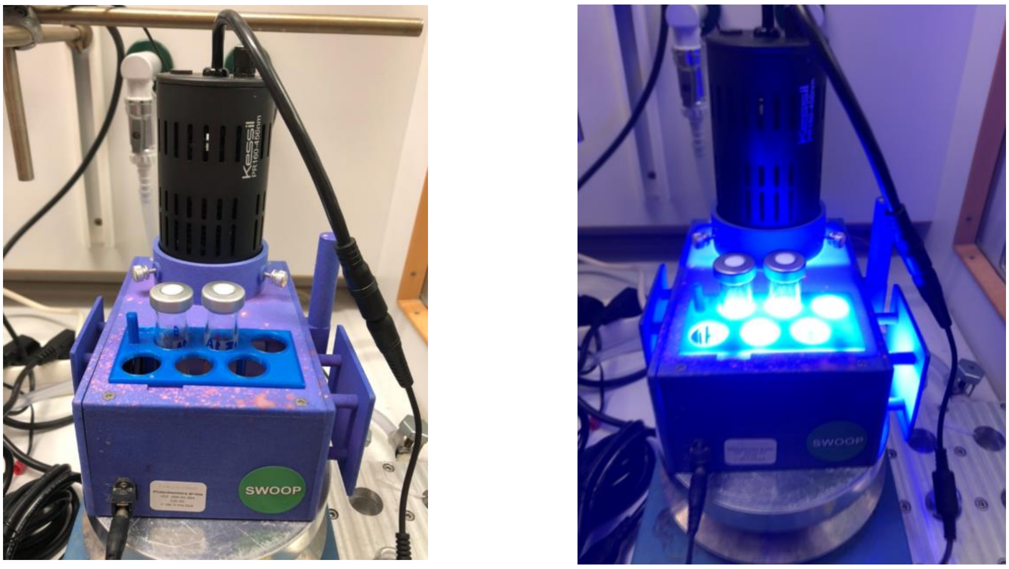

Supplementary Figure 3. Photoreactor set-up used for optimization and scope of the LSF of Peptides on Resin

5. Optimization

5.1 Preliminary attempts of the C1 Homologation reaction

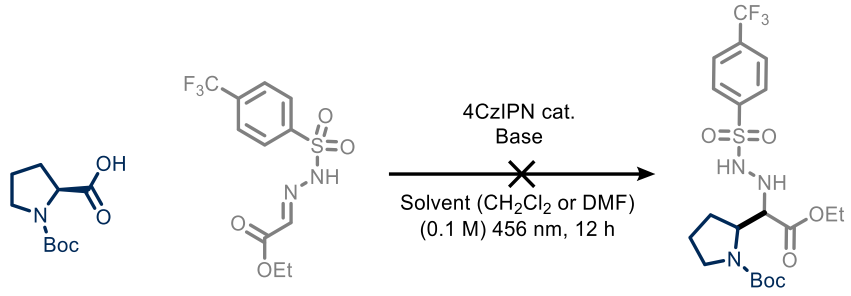

1 (1.0 equiv) **2** (2.0 equiv) **int-a**

Preliminary efforts were directed towards a redox neutral decarboxylative homologation of free carboxylic acids. From this study we concluded the incompatibility of **2** with a base, still necessary for the decarboxylative event to occur. Thus, we then started investigating the reactivity of redox active esters as their activation does not require the presence of a base.

5.2 Optimization of the C1 Homologation Reaction

Supplementary Table 1. First PCs screening (2.0 equiv. **2a**).

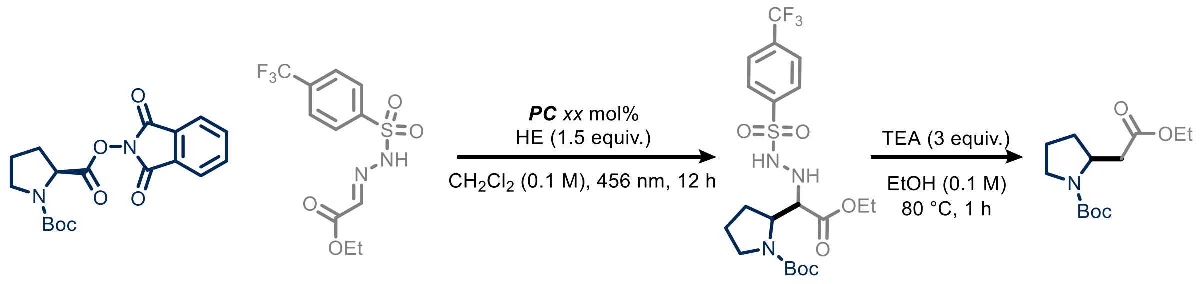

1a (1.0 equiv) **2a** (2.0 equiv) **int-a** **3**

PC	Variations	Yield of **3**[a]
$Ru(bpy)_3(PF_6)_2$ 1 mol%		65%
$Ru(bpy)_3(PF_6)_2$ 1 mol%	2.0 equiv. **1a**, 1.0 equiv. **2a**	44%
$Ru(bpy)_3(PF_6)_2$ 1 mol%	2.0 equiv. **HE**	67%
$Ir(ppy)_3$ 1 mol%		30%
4CzIPN 5 mol%		64%
$3DPA_2FBN$ 5 mol%		54%
Eosin-Y$(Na)_2$ 10 mol%		81%
Eosin-Y$(Na)_2$ 5 mol%		70%
No PC		n.d
No PC	390 nm	40%

Reaction conditions: **1a**, **2a**, HE and **PC** in 2 mL of dichloromethane (0.1 M) as indicated.
[a]Determined via ^1H NMR using trichloroethylene as external standard.
Cleavage conditions: Solvent switch to Ethanol (0.1 M), TEA (3 equiv.), 80 °C.

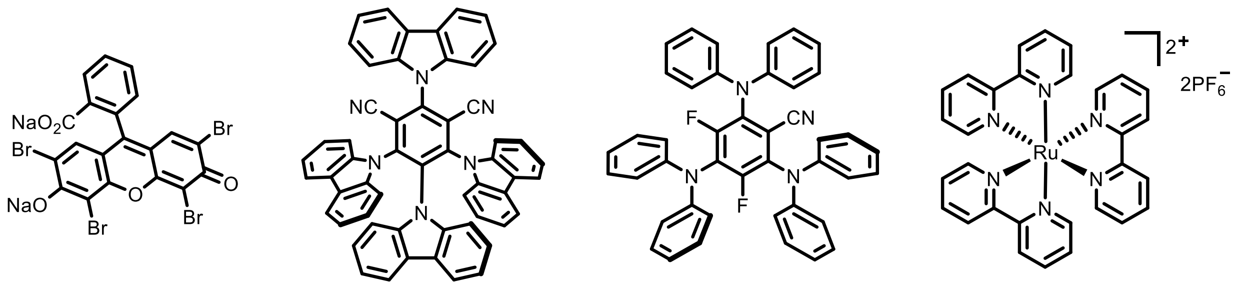

$EYNa_2$ **4CzIPN** **3DPA2FBN** **$Ru(bpy)_3(PF_6)_2$**

Supplementary Table 2. Solvent Screening (2.0 equiv. **2a**)

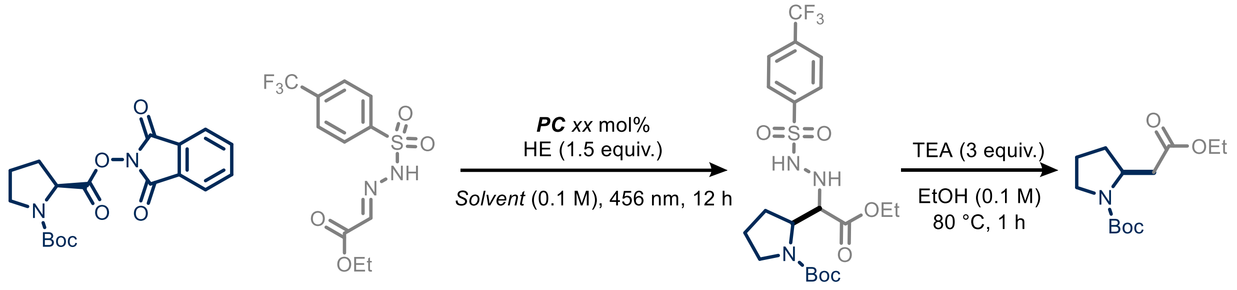

1a (1.0 equiv) **2a** (2.0 equiv) **int-a** **3**

**PC**	Photo Solvent	Cleavage Solvent	Yield of **3**[a]
Eosin-Y(Na)$_2$ 10 mol%	CH_2Cl_2	EtOH	81%
Eosin-Y(Na)$_2$ 10 mol%	EtOH	EtOH	messy crude
Eosin-Y(Na)$_2$ 10 mol%	DMF	EtOH	messy crude
Eosin-Y(Na)$_2$ 10 mol%	MeCN	EtOH	messy crude
Ru(bpy)$_3$(PF$_6$)$_2$ 1 mol%	MeCN	EtOH	messy crude
Ru(bpy)$_3$(PF$_6$)$_2$ 1 mol%	TFT	EtOH	64%
Ru(bpy)$_3$(PF$_6$)$_2$ 1 mol%	DMF	EtOH	71%
Ru(bpy)$_3$(PF$_6$)$_2$ 1 mol%	DMF	EtOH:DMF 1:1 (0.05 M)	63%

Reaction conditions: **1a**, **2a**, HE and **PC** in 2 mL of indicated solvent (0.1 M).
[a]Determined via ^{1}H NMR using trichloroethylene as external standard.
Cleavage conditions: Solvent switch to EtOH (0.1 M), TEA (3 equiv.), 80 °C.

Supplementary Table 3. Additional Screening (2.0 equiv. **2a**)

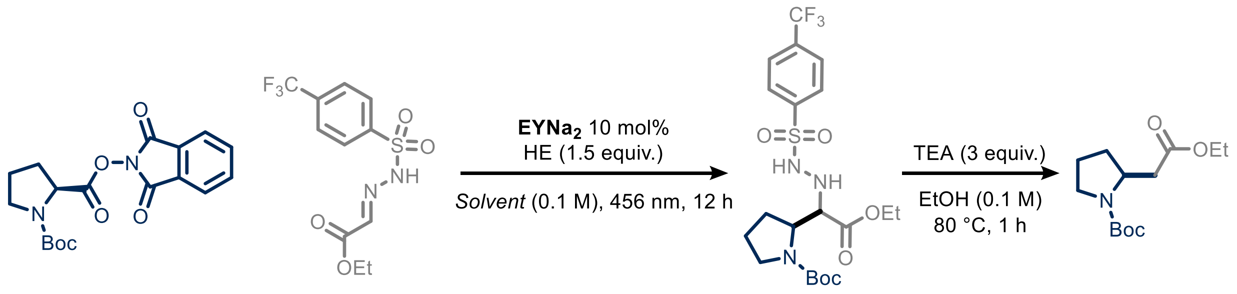

1a (1.0 equiv) **2a** (2.0 equiv) **int-a** **3**

Photo Solvent	Additive	Yield of **3**[a]
DCE	-	54%
Toluene	-	68%
THF	-	traces
Acetone	-	25%[b]
DCM	-	90%[b,c]
DCM	TFA 20 mol%	90%
DCM	HFIP 20 mol%	84%

Reaction conditions: **1a**, **2a**, HE and **PC** in 2 mL of indicated solvent (0.1 M).
[a]Determined via ^{1}H NMR using trichloroethylene as external standard.
[b]1 equiv. **2a.** [c]Buthanol was used for the cleavage.
Cleavage conditions: Solvent switch to Ethanol (0.1 M), TEA (3 equiv.), 80 °C.

Supplementary Table 4. Additional PCs screening (1.0 equiv. **2a**)

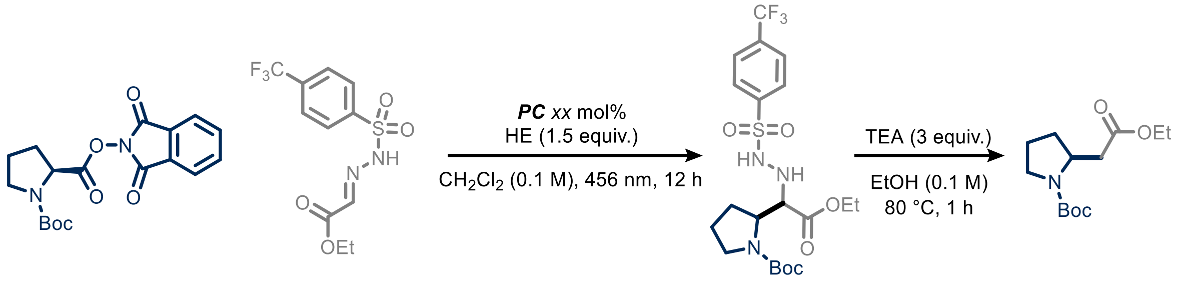

1a (1.0 equiv) **2a** (1.0 equiv) **int-a** **3**

**PC**	Yield of **3**[a]
$Ru(bpy)_3(PF_6)_2$ 1 mol%	66%
4CzIPN 5 mol%	61%
$3DPA_2FBN$ 5 mol%	53%
EYH_2 10 mol%	75%
$EYNa_2$ 10 mol%	90%

Reaction conditions: **1a**, **2a**, HE and **PC** in 2 mL of dichloromethane (0.1 M).
[a]Determined via ^{1}H NMR using trichloroethylene as external standard.
Cleavage conditions: Solvent switch to Ethanol (0.1 M), TEA (3 equiv.), 80 °C.

Supplementary Table 5. Reductive quenchers screening.

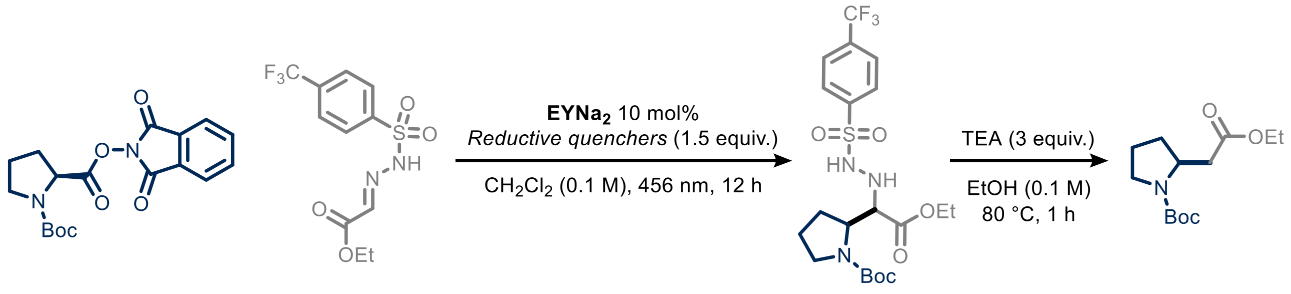

1a (1.0 equiv) **2a** (1.0 equiv) **int-a** **3**

Reductive quenchers	Yield of **3**[a]
DABCO	n.d.
Tetramethylguanidine	n.d.
DIPEA	n.d.
without HE	n.d.
HE	90%

Reaction conditions: **1a**, **2a**, Reductive Quenchers and **PC** in 2 mL of dichloromethane (0.1 M).
[a]Determined via ^{1}H NMR using trichloroethylene as external standard.
Cleavage conditions: Solvent switch to Ethanol (0.1 M), TEA (3 equiv.), 80 °C.

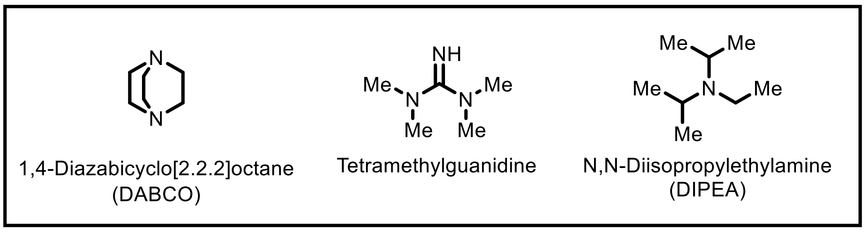

5.3 Optimization Alkylation Reaction

Supplementary Table 6. Solvent Screening.

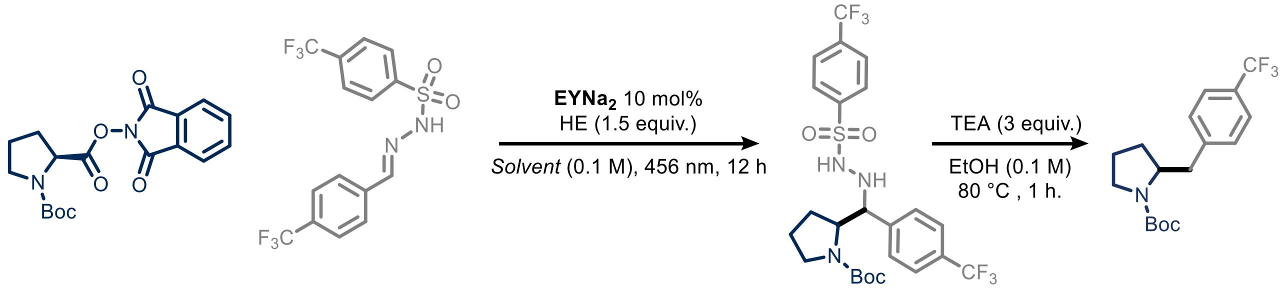

Solvent	Yield of **45**[a]
Acetone	43%
EtOAc	37%
MeCN	50%
Toluene	21%
CH_2Cl_2	57%
CH_2Cl_2 + 20 equiv. H_2O	56%
CH_2Cl_2 + 50 equiv. H_2O	56%
CH_2Cl_2 (no degass)	54%

Reaction conditions: **1a**, **2c**, HE and **EYNa$_2$** in 2 mL of indicated solvent (0.1 M).
[a]Determined via GC-FID calibration curve using dodecane as external standard.
Cleavage conditions: Solvent switch to Ethanol (0.1 M), TEA (3 equiv.), 80 °C.

Supplementary Table 7. Additional Screening.

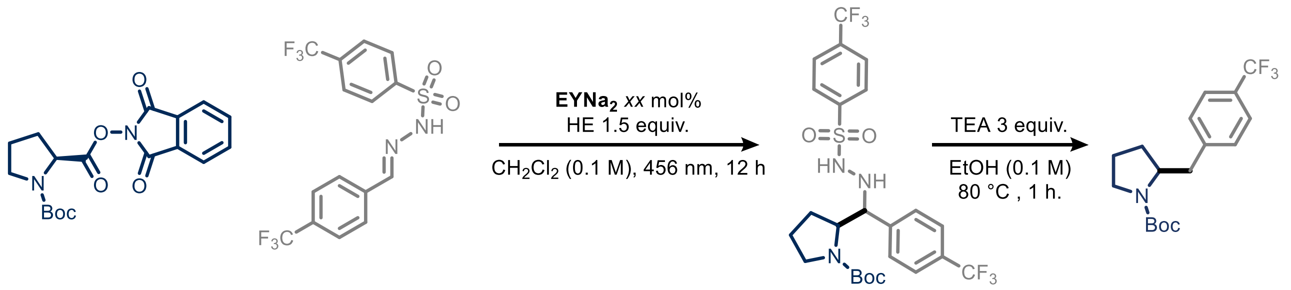

1a (1.0 equiv.) **2c** (1.0 equiv.) **int-b** **45**

Catalyst Loading	Variations	Yield of **45**[a]
10 mol%	-	57%
10 mol%	**EYH$_2$** was used	55%
10 mol%	**EYH$_2$** and **2b** were used	61%
10 mol%	green light 525 nm	57%
10 mol%	0.05 M DCM	58%
20 mol%	-	45%
10 mol%	2 equiv. RAE **1a**	52%
20 mol%	2 equiv. RAE **1a**	58%
10 mol%	RAE **1a-4Cl** was used	44%
10 mol%	1 equiv. HE	46%
10 mol%	2 equiv. HE	38%
10 mol%	(L)-Boc-Phe-OH (1 equiv.) as additive	50%
10 mol%	hydrazone **2p** was used	25%
10 mol%	2 equiv. **1a**, hydrazone **2p** was used	50%

Reaction conditions: **1a**, **2c**, HE and **EYNa$_2$** in 2 mL of indicated solvent (0.1 M).
[a]Determined via GC-FID calibration curve using dodecane as external standard.
Cleavage conditions: Solvent switch to Ethanol (0.1 M), TEA (3 equiv.), 80 °C.

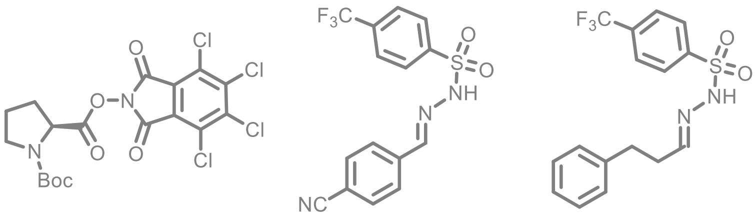

1a-4Cl **2b** **2p**

5.4 Sensitivity Assessment C1 Homologation

In order to expand the methodology to the late stage functionalization of peptides on solid phase (rink amide resin), we set to explore the compatibility of protected amino acids in the protocol.

Supplementary Table 8. Sensitivity Assessment of the C1 Homologation Reaction.

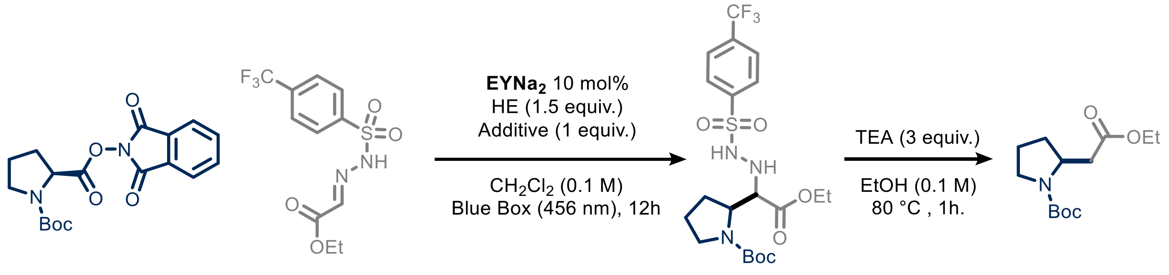

1a (1.0 equiv) **2a** (1.0 equiv) **int-a** **3**

conversion **1**	Additive	Yield of **3**
100%	none	91%
100%	Boc-Ph-OH	95%
100%	Fmoc-Ph-OH	95%
100%	Fmoc-Ala-OH	90%
100%	Fmoc-Lys(Boc)-OH	93%
100%	Fmoc-Arg(Pbf)-OH	70%
100%	Fmoc-Trp(Boc)-OH	88%
100%	Fmoc-Cys(Trt)-OH	77%
100%	Fmoc-Met-OH	82%
100%	Fmoc-Lys(Boc)-OH	93%
100%	Fmoc-Hyst(Trt)-OH	75%

Reaction conditions: **1a** (0.3 mmol, 1 equiv.), **2a** (0.33 mmol, 1.1 equiv.), Hantzsch (1.5 equiv.), Additive (1.0 equiv.) and PC in 3 mL CH_2Cl_2 (0.1 M).
^{1}H NMR (trichloroethylene external standard).

In the specific, we opted for Fmoc-protected amino acids bearing fully protected side chains due to their availability and low price. Despite Fmoc removal was envisioned during the fragmentation of the intermediate, the goal of the assessment was to evaluate the compatibility of the functional groups of amino acids side chains, that being redox active, could disrupt the desired reactivity.

6. General procedure 3 (GP3): Photochemical radical addition to benzenesulfonyl hydrazones and subsequent fragmentation of the benzenesulfonyl hydrazide

In a typical experiment, to an oven-dried 7 mL vial equipped with a stirring bar were added 4-(trifluoromethyl)sulfonyl hydrazone (0.300 mmol, 1 equiv.), redox active ester (0.300 mmol, 1.0 equiv.), Hantzsch ester (114 mg, 0.450 mmol, 1.5 equiv.) and **$EYNa_2$** (20.8 mg, 10 mol%).and the vial was sealed with a rubber septum. Subsequently, dry and degassed dichloromethane (3 mL) was added under N_2 atmosphere (0.1 M). The vial was stirred and irradiated in the UFO photochemical reactor (**See section 4**) for 12 h. The temperature was maintained at 30 °C during the course of the reaction.
Then, the vial was removed from the photochemical reactor and the solvent was evaporated under reduced pressure. The obtained crude mixture was then dissolved in 3 mL of ethanol (0.1 M), TEA was added (3.0 equiv., 0.9 mmol, 125 µL) and the vial was placed in an oil bath at 80°C for 1 h. The reaction mixture was cooled to rt and the solvent was removed under reduced pressure. The obtained crude was diluted with diethyl ether and washed with 1 M HCl. The combined organic layers were dried over Na_2SO_4, filtered and the solvent was removed under reduced pressure. The crude reaction mixture was then purified by flash column chromatography on silica gel.

7. Solid Phase Synthesis of Peptides and Purification

The peptides were synthesized via Fmoc solid-phase peptide synthesis using a Biotage® Initiator+ AlstraTM automated peptide synthesizer. Peptide syntheses were monitored by reversed-phase (RP) UPLC-MS. Analytical RP-UPLC-MS was performed on a Waters Acquity UPLC system (PDA, sample manager, sample organizer, column oven modules) and Waters SQD2 mass spectrometer using the following column: Waters Acquity CSH C18 column, 130Å, 1.7 µm, 50 × 2.1 mm at a flow rate of 0.5 mL/min at 45 °C.
A linear gradient of mobile phase: A=H_2O + 10 mM formic acid, 1 mM ammonia and 0.03% TFA and B=acetonitrile/H_2O 95/5 v/v + 10 mM formic acid, 1 mM ammonia and 0.03% TFA was used with detection from 210 - 350 nm.
High resolution mass spectra of the purified peptides (HRMS) were collected using a Water Synapt G2Si QTOF Mass Spectrometer. Column: Aquity UPLC CSH C19 100 mm x 2.1 mm, 1.7 µm particles. Mobile Phases: 1mM ammonium formate, 10 mM formic acid, 0.03 % TFA, pH=3, in MilliQ (A) and MeCN (B), respectively. Flow Rate: 0.5 mL/min. Purity: Relative absorbance at 214 nm.
Water was purified using a Millipore MilliQ water purification system. Peptides were synthesized using standard Fmoc SPPS. Fmoc-amino acids were purchased from Chem-Impex International, Inc., with the following side-chain protection: Fmoc-Arg(Pbf)-OH, Fmoc-Asn(Trt)-OH, Fmoc-Asp(OtBu)-OH, Fmoc-Cys(Trt)-OH, Fmoc-Gln(Trt)-OH, Fmoc-Glu(OtBu)-OH, Fmoc-His(Trt)-OH, Fmoc-Lys(Boc)-OH, Fmoc-Ser(tBu)-OH, Fmoc-Thr(tBu)-OH, Fmoc-Trp(Boc)-OH, Fmoc-Tyr(tBu)-OH amd Fmoc-(D)-Phe-OH. Oxyma was purchased from Chem-Impex International, Inc., DIPEA and FITC from Sigma-Aldrich. Acetic anhydride was purchased from Acros Organics.
Peptide synthesis
General Protocol for SPPS. The peptides were synthesized via Fmoc solid-phase peptide. The peptide chains were then assembled following **method A** and then N-terminal acetylation was performed according **method B** before being cleaved from the resin, deprotected according to **method C** and purified.
Method A: Automated Fmoc SPPS, Biotage® Initiator+ AlstraTM automated microwave peptide synthesizer. Rink Amide MBHA resin (final loading 0.77 mmol/g) was swollen in CH_2Cl_2 for 10 min and then the solvent was drained. The Fmoc N-protecting group was removed with 20% piperidine in DMF (2 x 5 min) at room temperature. The amino acids (4 equiv.) dissolved in DMF (0.2 M) were repeatedly coupled with DIC (4 equiv.) in DMF (2 M) and OXYMA (4 equiv.) in DMF (0.5 M) at 40 degrees for 10 min. Washing of the resin between the coupling steps was performed with EtOAc.DMSO (9:1). The resin was finally washed with CH_2Cl_2 (2 x 5 mL).
Method D: Acetylation of the N terminal position. After the final Fmoc deprotection, the resin was swollen with CH_2Cl_2 (2 x 5 mL) and drained. A solution of NMP: acetic anhydride: 2,6 Lutidine 90:5:5, (9 mL) was added to the resin and stirred at room temperature for 60 min before the mixture was drained. This procedure was repeated twice, and the resin was washed with DMF (3 x 5 mL) and CH_2Cl_2 (3 x 5 mL).
Method E: Cleavage from resin/side-chain deprotection. A solution of TFA/water/DODT/TIS 90:2.5:2.5:5 (10 mL) was added to the dry resin. The reaction mixture was shaken at room temperature for 2 h. The resin was rinsed with TFA (2 x 0.3 mL) and the TFA solution collected in a flask and concentrated under reduced pressure to reduce

the total volume. The solution was then poured in cold diethyl ether. The precipitated peptide was centrifuged, and the crude peptide was lyophilized from acetonitrile-water.

General method for Alloc deprotection

The resin bound peptide was suspended in anhydrous CH_2Cl_2 followed by the addition of phenylsilane (25 equiv.) and $Pd[(C_6H_5)_3P]_4$ (0.25 equiv.) and the suspension was shaken for 1 h followed by removal of the liquid and the addition of fresh reagents. After 1 h, the peptidyl resin was drained and washed with CH_2Cl_2 (3 x 5 mL), DMF (3 x 5 mL).

Coupling of H-Pro-OAllyl·TFA

A solution of H-Pro-OAllyl·TFA (10.0 equiv.), PyBOP (10.0 equiv.) and DIPEA (20.0 equiv.) in DMF (final concentration 0.1 M) was added to the resin (1.0 equiv.) and shaken. After 3 h, the resin was drained and washed with DMF (5 x 3 mL) and CH_2Cl_2 (5 x 3 mL).

Purification

Reverse phase HPLC (Shimadzu Preparative LCMS Nexera LC-40), (solvent system: A: MeCN and B: 5% TFA in H_2O). Column: Shim-pack GIST, 5µm C18, 20 x 250 mm. Collected fraction were lyophilized to afford the desired peptide.

Synthetic Route to Peptide P0

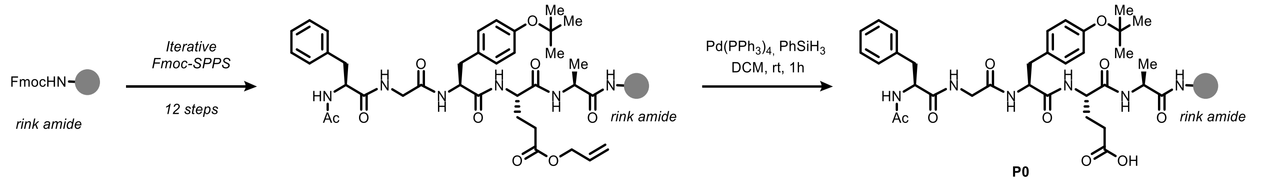

Supplementary Figure 4: Synthesis of peptide **P0**.

Synthetic Route to Peptide P1

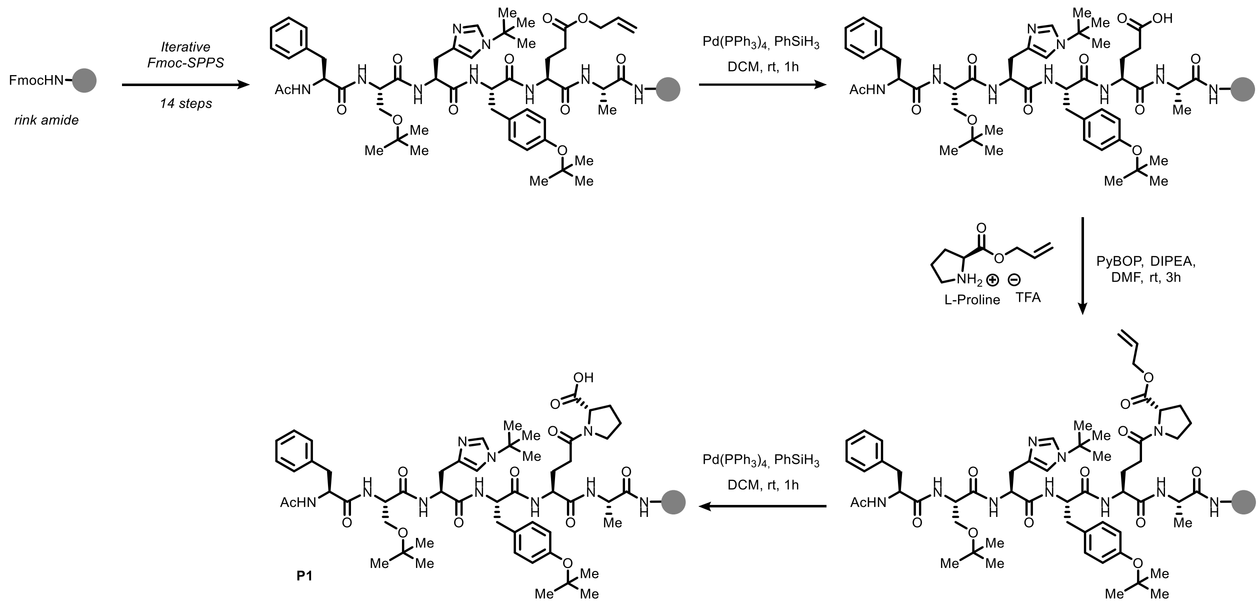

Supplementary Figure 5: Synthesis of peptide **P1**.

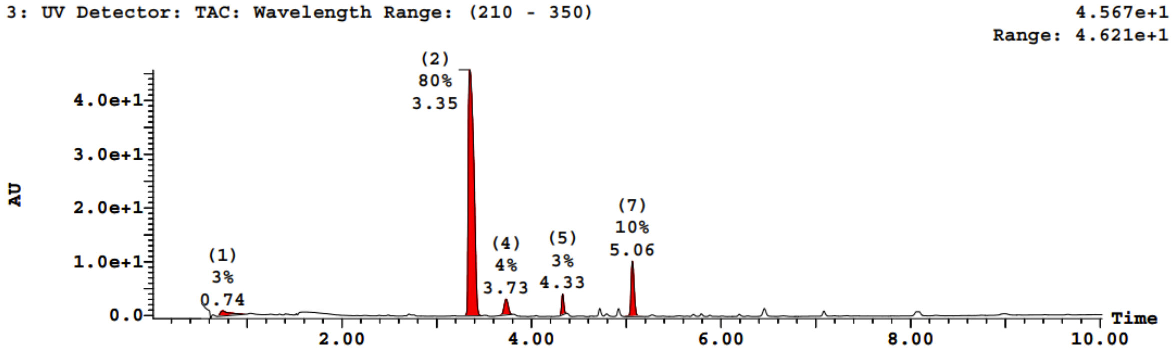

Supplementary Figure 6: UPLC-MS Trace of the resin-bound crude peptide P1. Analyzed by cleavage of a fraction of resin beads by treatment with TFA/DODT/Water/TIS (90: 2.5: 2.5: 5, v/v) for 1 h at rt. Conditions: (3 to 43 % B over 10 min).

Synthetic Route to Peptide P2

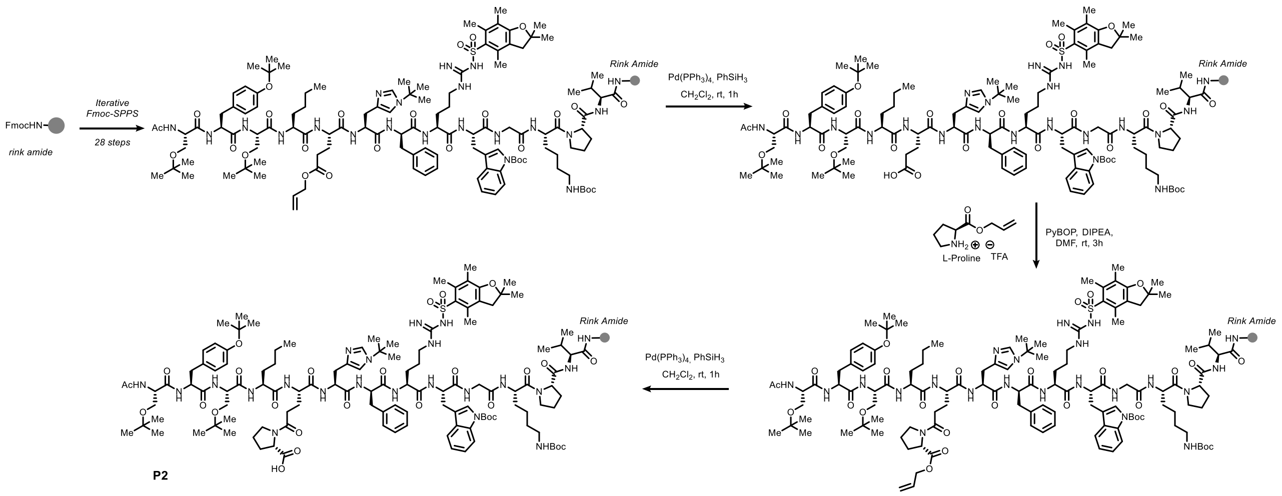

Supplementary Figure 7: Synthesis of peptide **P2**.

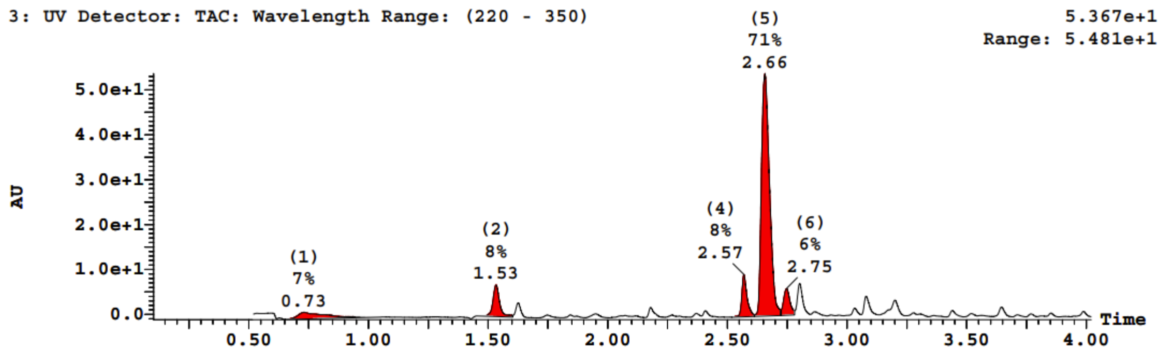

Supplementary Figure 8: UPLC-MS Trace of the resin-bound crude peptide P2. Analyzed by cleavage of a fraction of resin beads by treatment with TFA/DODT/Water/TIS (90: 2.5: 2.5: 5, v/v) for 1 h at rt. Conditions: (3 to 43 % B over 4 min).

7.1 General Procedure 4 (GP4): Synthesis of Redox Active Esters (RAEs) on Resin

The resin-bound peptide (30 µmol), N-hydroxyphthalimide (NHPI) (98 mg, 0.6 mmol, 20.0 equiv.) and DMAP (7.3 mg, 0.06 mmol, 2.0 equiv.) were added to the the fritted syringe. Then, a solution of DIC (94 µL, 0.6 mmol, 20.0 equiv.) in dry DMF (25-50 mM concentration with respect to the resin-bound peptide) was withdrawn with the syringe containing the solids. The syringe containing the resin and all the activating agents was capped, sealed with Teflon tape, located in a plastic casing. Then was placed in a falcon tube containing water and agitated on a orbital shaker at the indicated temperature for 2h. The activation solution was then drained and the resin washed with dry DMF (5 x 3 mL), dry CH_2Cl_2 (3 x 5 mL).

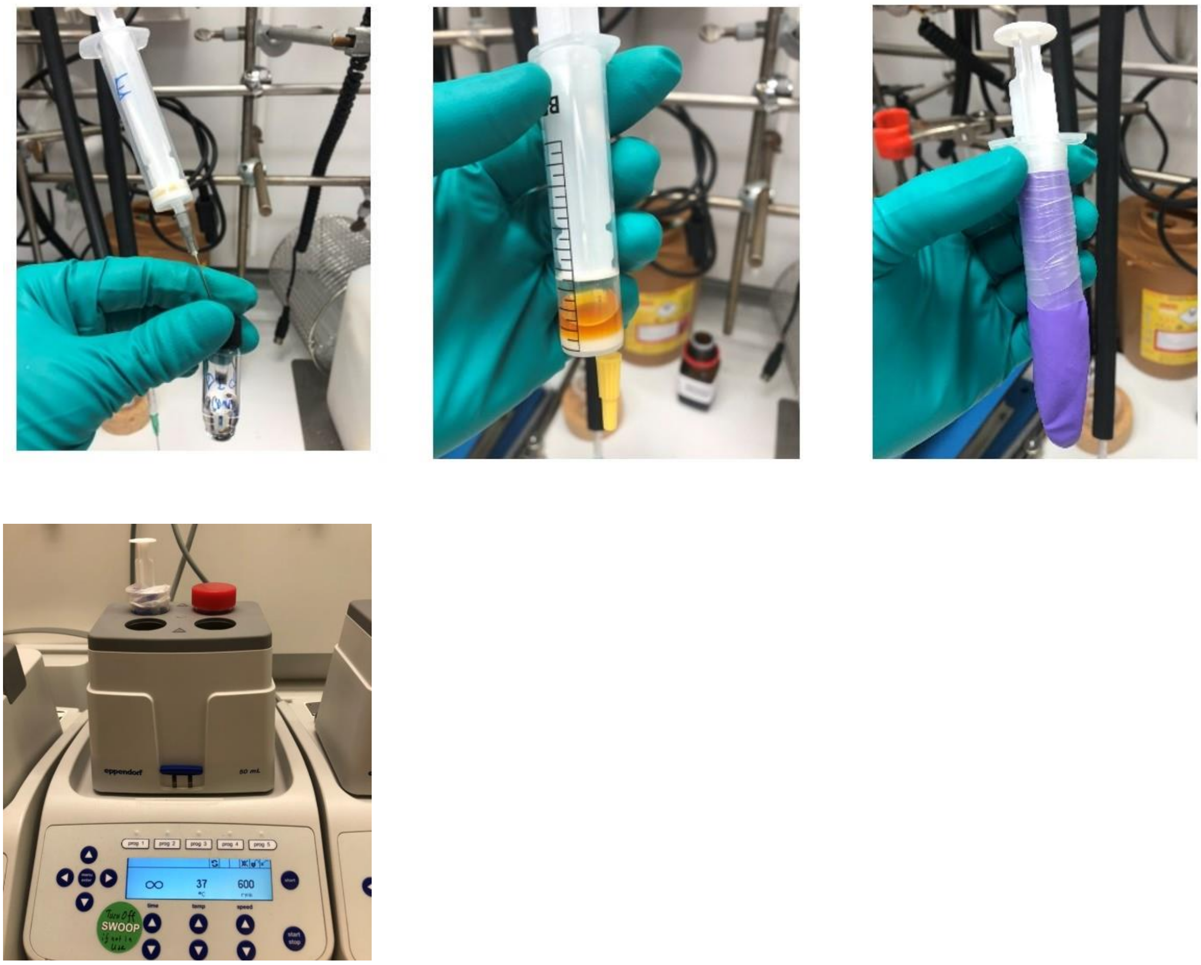

Supplementary Figure 9: Graphical procedure for the synthesis of redox active esters on resins.

7.2 General Procedure 5 (GP5): On resin photochemical radical addition to Aryl Sulfonyl Hydrazones 2a and 2b

After the activation step (GP4), the resin-bound peptide was subjected to the photochemical coupling without any purification. After drying, the resin (30 µmol, 1 equiv.) was transferred to an oven-dried 7 mL vial equipped with a stirring bar. Then, 4-(trifluoromethyl)sulfonyl hydrazone **2a** or **2b** (29 mg, 90 µmol, 3 equiv.), Hantzsch Ester (34 mg, 135 µmol, 4.5 equiv.) and **EYNa$_2$** (6.2 mg, 9 µmol, 0.3 equiv.) were added. The vial was sealed and 90 µL of dry CH_2Cl_2 were added (33 mM concentration in respect to the resin bound peptide) and stirred for 12h in the Blue Box photochemical reactor (See Section 4 for the Setup details).

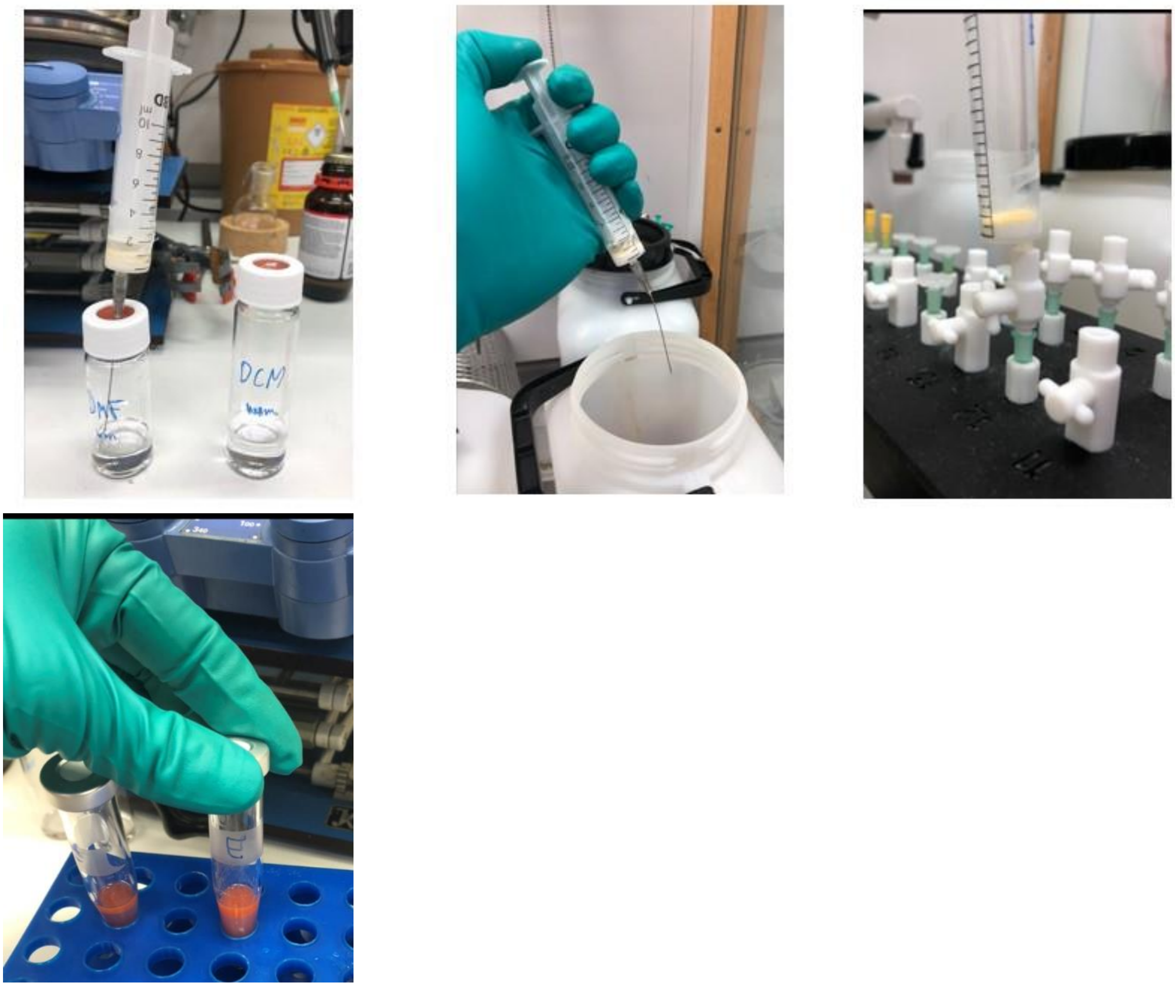

Supplementary Figure 10: Graphical procedure for the photochemical step on resin.

7.3 General Procedure 6 (GP6): Cleavage of the Alkylated Sulfonyl Hydrazide Intermediate on Resin

Then, the suspension containing the resin-bound peptide was transferred to a frittered syringe, drained and washed with DMF (5 x 3 mL) and CH_2Cl_2 (5 x 3 mL). After drying the resin, it was transferred to a falcon tube and a 90 µL (1:1 v:v) solution of EtOH-DMF was added (33 mM final concentration) followed by the addition of TEA (38 µL, 9 equiv., 270 µmol). The tube was closed and agitated on a orbital shaker for 2 h at 80 °C degrees. Next, the solution was transferred to a frittered syringe, and the resin was draine, washed with dry DMF (5 x 3 mL) and dry CH_2Cl_2 (5 x 3 mL). The reaction was analyzed by cleavage of a fraction of resin beads by treatment with TFA/DODT/Water/TIS (90: 2.5: 2.5: 5, v/v) for 1 h at rt and analysis by UPLC-MS.

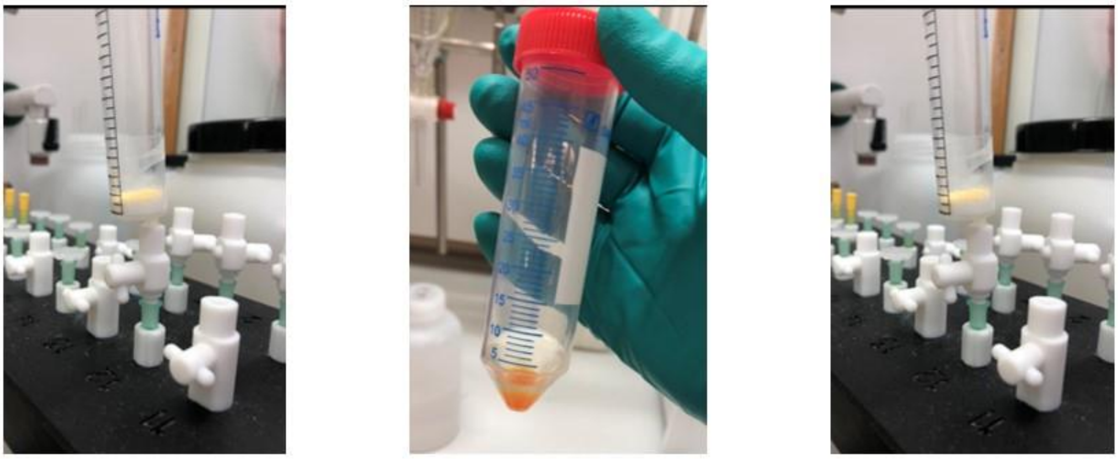

Supplementary Figure 11: Graphical procedure for the cleavage step on resin.

7.4 Optimization of the on-resin photochemical alkylation of peptides

First round of optimization of the on-resin photochemical alkylation for peptide P0

Supplementary Table 9. First round of reaction screening.

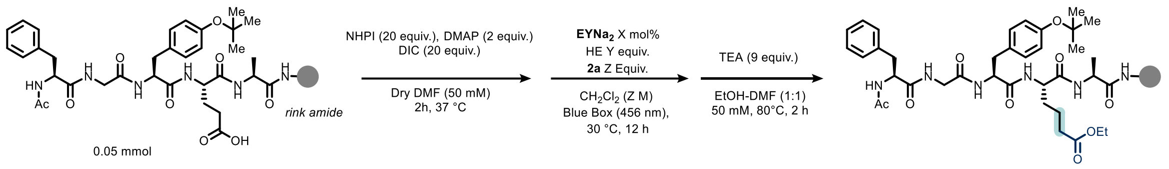

Entry	conversion	Concentration	**$EYNa_2$ %**	**HE Equiv.**	**2a Equiv.**	Product (peak **5**)	Aspartimide (cyclization) (peak **2**)
1	100%	100 mM	10	1.5	1	traces	70%
2	100%	50 mM	20	3	2	10%	50%
**3**	**100%**	**33 mM**	**30**	**4.5**	**3**	**20%**	**34%**
4	100%	33 mM	10	1.5	1	10%	48%
5	100%	25 mM	30	4.5	3	18%	53%

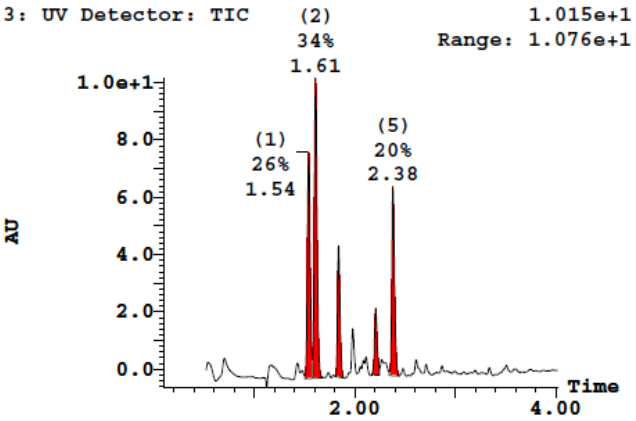

Supplementary Figure 12: UPLC-MS Trace of Entry 3. Analyzed by cleavage of a fraction of resin beads by treatment with TFA/DODT/Water/TIS (90: 2.5: 2.5: 5, v/v) for 1 h at rt. Conditions: (10 to 50 % B over 4 min).

Optimization of the on-resin redox active ester synthesis

The optimization reported above revealed that the formation of aspartimide could not be completely suppressed. Thus, we envisioned that the formation of the side product might derive from an inefficient formation of the RAE or from inadequate reaction conditions of this activation step. In this section is reported the optimization toward the formation of RAE intermediate.

Supplementary Table 10. Redox active ester optimization screening.

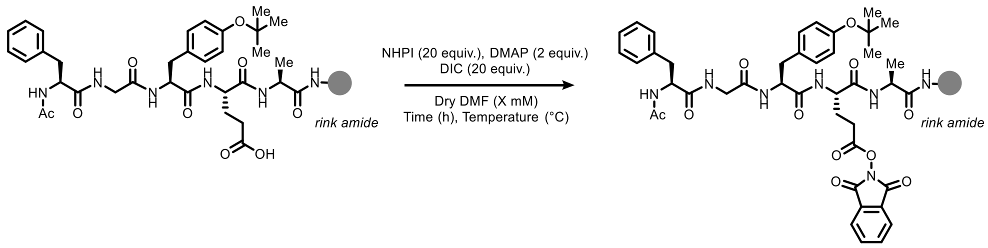

Entry	conversion	Concentration	Time (h)	Temperature (°C)	Product (peak **5**)	Aspartimide (peak **3**)
1	100%	100 mM	2h	37	40%	50%
2	100%	50 mM	2h	37	50%	42%
3	100%	33 mM	2h	37	62%	32%
4	100%	25 mM	2h	37	74%	17%
**5**	**100%**	**25 mM**	**1.5**	**37**	**84%**	**4%**
6	100%	25 mM	1.5	20	20%	8%

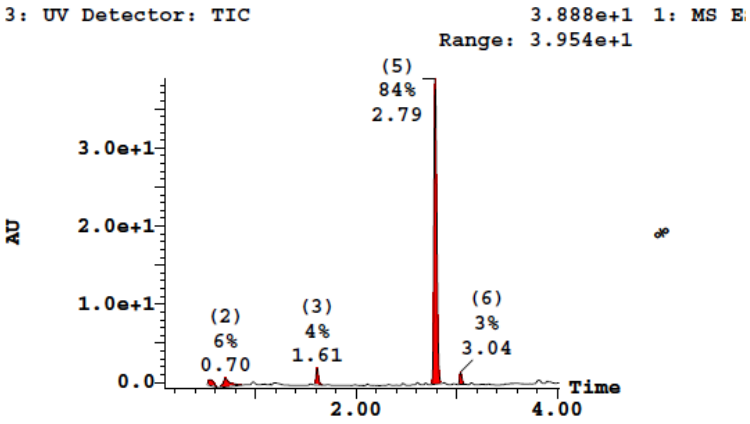

Supplementary Figure 13: UPLC-MS Trace of Entry 5. Analyzed by cleavage of a fraction of resin beads by treatment with TFA/DODT/Water/TIS (90: 2.5: 2.5: 5, v/v) for 1 h at rt. Conditions: (10 to 50 % B over 4 min).

Second round of optimization of the on-resin photochemical alkylation for peptide P0

Supplementary Table 11. Second round of reaction screening.

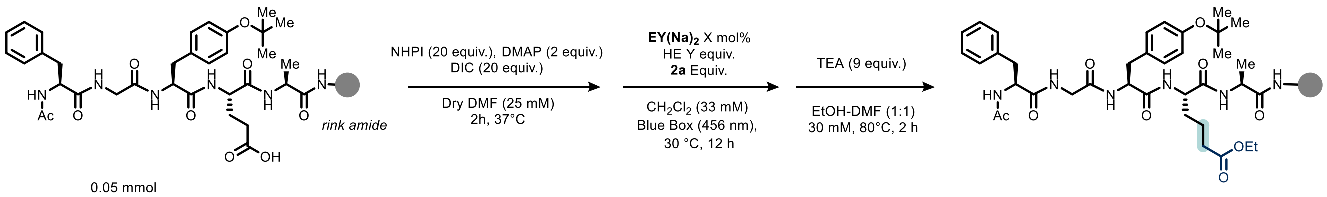

Entry	conversion **1**	**EYNa$_2$ %**	HE equiv.	**2a equiv.**	Product (peak **5**)	Aspartimide (peak **2**)
1	100%	10	1.5	1	4%	60%
2	100%	20	3	2	18%	35%
**3**	**100%**	**30**	**4.5**	**3**	**26%**	**20%**
4	100%	40	7.5	4	22%	27%

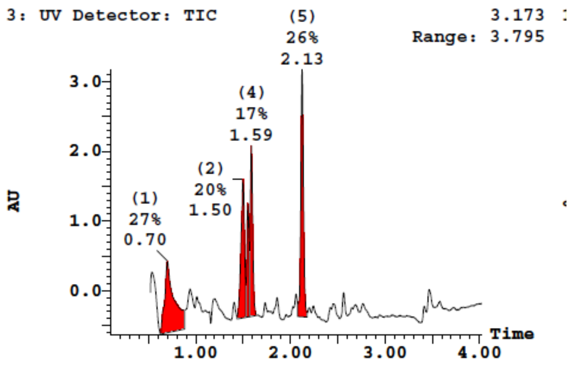

Supplementary Figure 14: UPLC-MS Trace of Entry 3. Analyzed by cleavage of a fraction of resin beads by treatment with TFA/DODT/Water/TIS (90: 2.5: 2.5: 5, v/v) for 1 h at rt. Conditions: (10 to 50 % B over 4 min).

Despite the improved conditions for the activation step (formation of the redox active ester), the reaction yield could not be further improved and beside aspartimide (Supplementary Figure 14, peak 2) and the desired product (Supplementary Figure 14, peak 5), the major side product observed was the decarboxylative reduction of peptide P0 (Supplementary Figure 14, peak 4) in 17%.

Attempt of the on-resin benzylation of peptide P0

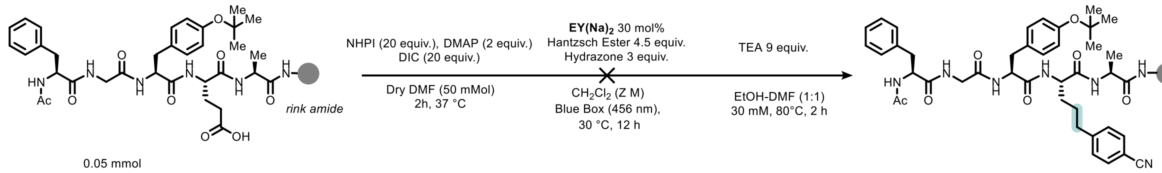

Supplementary Figure 15: Failed on-resin benzylation of peptide **P0**.

In line with what observed during the scope of the reaction in solution, where only α-amino acids derived RAE are competent substrate in the benzylation protocol, peptide P0 RAE did not afford any desired product and only aspartimide formation and reduced product were observed.

Optimization of the on-resin activation and photochemical alkylation for peptide P1

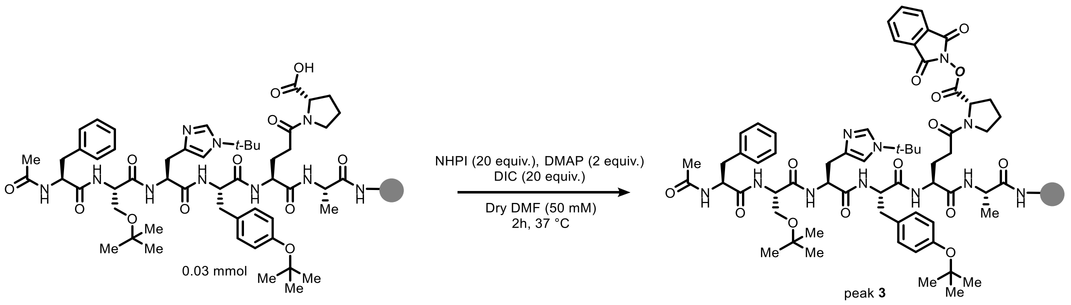

Supplementary Figure 16: On-resin activation of **P1**.

For the proline-derived containing peptide P1, clean redox active ester formation was observed (peak **3**, Supplementary Figure 17) and no aspartimide was detected, even at higher concentration (50 mM).

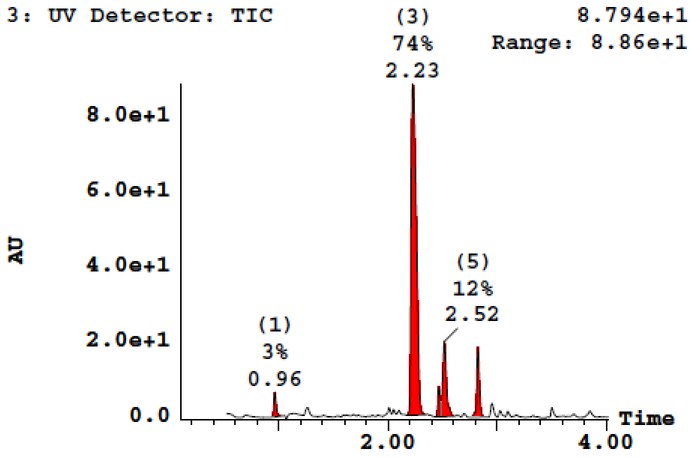

Supplementary Figure 17: UPLC-MS Trace of crude RAE synthesis for peptide P1 on resin. Analyzed by cleavage of a fraction of resin beads by treatment with TFA/DODT/Water/TIS (90: 2.5: 2.5: 5, v/v) for 1 h at rt. Conditions: (10 to 50 % B over 4 min).

Optimization of the on-resin photochemical alkylation for peptide P1

Supplementary Table 12. Optimization for the alkylation of **P1**.

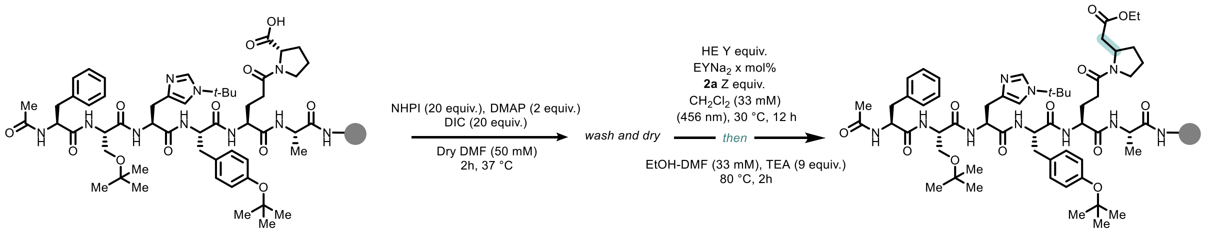

Entry	conversion	**$EYNa_2$ %**	HE equiv.	**2a equiv.**	Yield
1	100%	10	1.5	1	11%
2	100%	20	3	2	32%
**3**	**100%**	**30**	**4.5**	**3**	**63%**
4	100%	10	4.5	3	42%
5	100%	30	1.5	3	29%

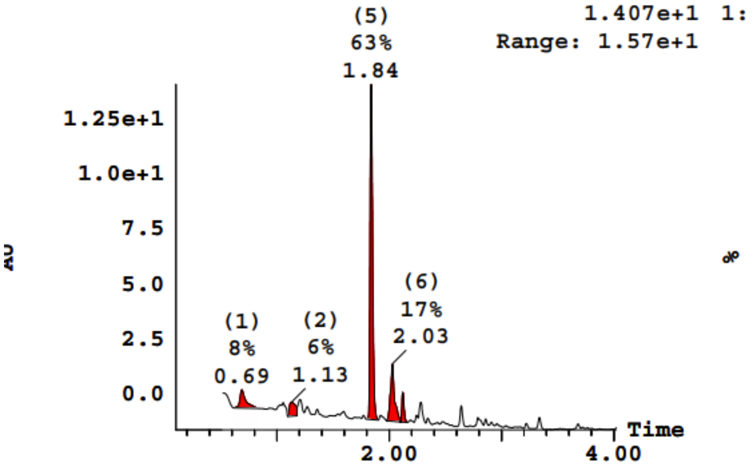

Supplementary Figure 18: UPLC-MS Trace of Entry 3. Analyzed by cleavage of a fraction of resin beads by treatment with TFA/DODT/Water/TIS (90: 2.5: 2.5: 5, v/v) for 1 h at rt. Conditions: (10 to 50 % B over 4 min).

7.5 Late Stage Functionalization of Peptides on Solid Phase: Characterization Data

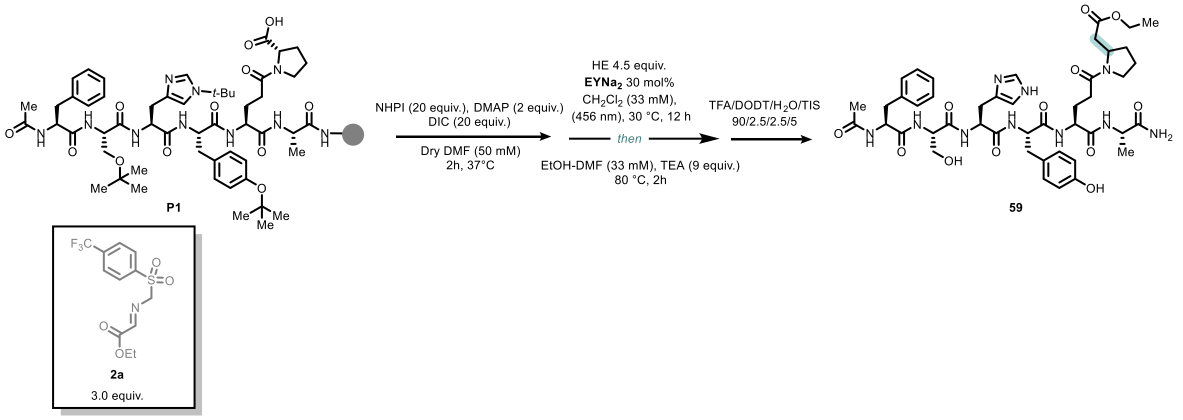

Supplementary Figure 19: Synthesis of **59**.

Peptide **59** was prepared on a 30 µmol scale from resin-bound substrate P1 following general procedure GP4 for the synthesis of the corresponding RAE on resin, GP5 for the photocatalytic alkylation step and general procedure GP6 for the thermal cleavage of the alkylated sulfonylhydrazide. After cleavage from the resin and ether precipitation, the crude peptide was purified by reverse phase HPLC (Shimadzu Preparative LCMS Nexera LC-40), (solvent system: A: MeCN and B: 5% TFA in H_2O), (20% B for 5 min, 20% to 60% B gradient over 16 min) and lyophilized to afford peptide **59** as a fluffy white solid (7.8 mg, 28% yield calculated from the original resin loading).

HRMS (ESI+) (m/z): $[M+2H]^{2+}$ calcd. for $C_{45}H_{62}N_{10}O_{12}$, 467.2274; found: 467.2274.

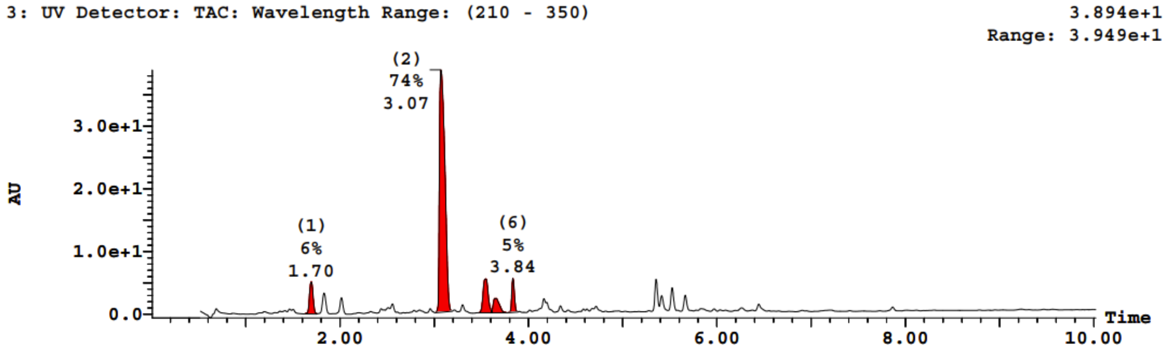

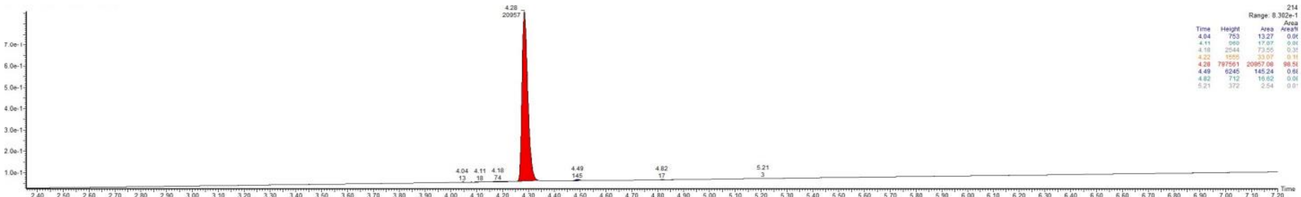

Supplementary Figure 20: **Up)** UPLC-MS Trace of the crude reaction mixture (product **59**) after full cleavage. Conditions: (10 to 50 % B over 10 min). **Down)** UPLC-MS Trace of the (product **59**) after purification. Conditions: (10% MeCN 1 min; 10-60% 1-9 min; 60-95% 9-10 min).

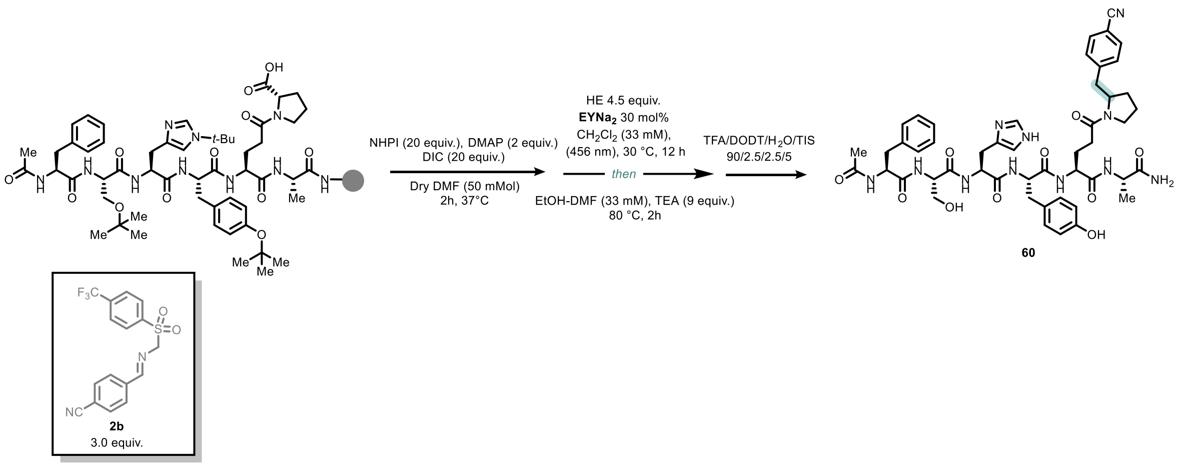

Supplementary Figure 21: Synthesis of **60**.

Peptide **60** was prepared on a 30 µmol scale from resin-bound substrate P1 following general procedure GP4 for the synthesis of the corresponding RAE on resin, GP5 for the photocatalytic alkylation step and general procedure GP6 for the thermal cleavage of the alkylated sulfonylhydrazide. After cleavage from the resin and ether precipitation, the crude peptide was purified by reverse phase HPLC (Shimadzu Preparative LCMS Nexera LC-40), (solvent system: A: MeCN and B: 5% TFA in H_2O), (25% B for 5 min, 25% to 65% B gradient over 16 min) and lyophilized to afford peptide **60** as a fluffy white solid (6.1 mg, 21% yield calculated from the original resin loading).

HRMS (ESI+) (m/z): $[M+2H]^{2+}$ calcd. for $C_{49}H_{59}N_{11}O_{10}$, 481.7301; found: 481.7306.

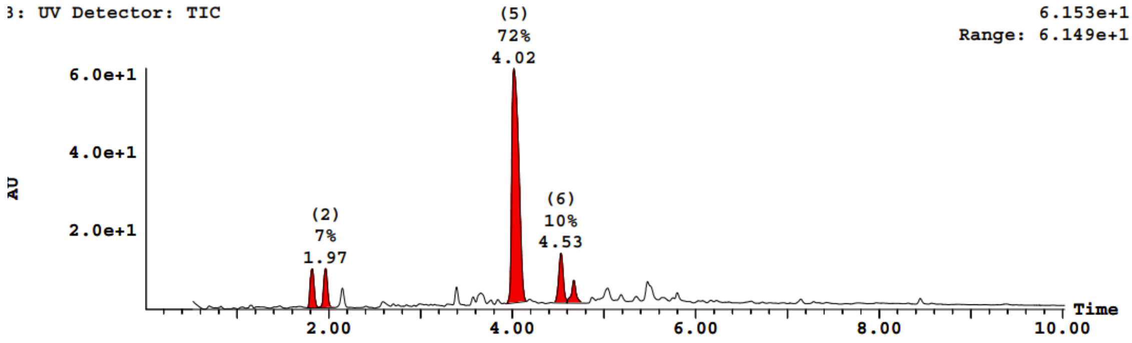

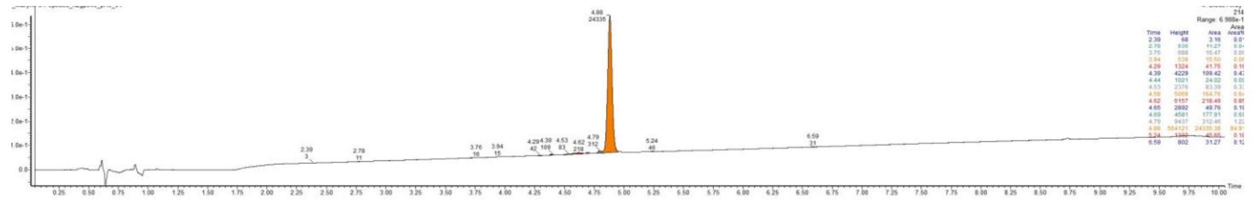

Supplementary Figure 22: **Up)** UPLC-MS Trace of the crude reaction mixture (product **60**) after full cleavage. Conditions: (10 to 50 % B over 10 min). **Down)** UPLC-MS Trace of the (product **60**) after purification. Conditions: (10% MeCN 1 min; 10-60% 1-9 min; 60-95% 9-10 min).

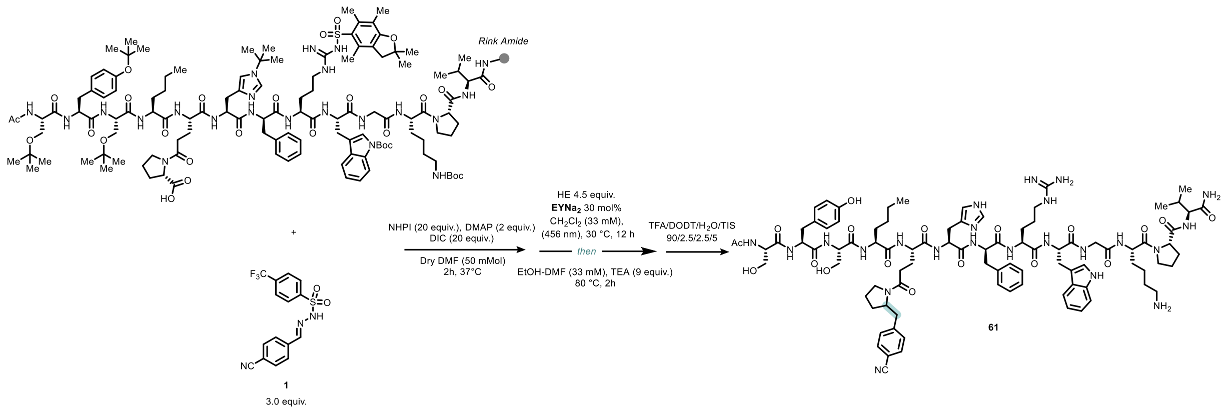

Supplementary Figure 23: Synthesis of **61**.

Peptide **61** was prepared on a 30 µmol scale from resin-bound substrate P2 following general procedure GP4 for the synthesis of the corresponding RAE on resin, GP5 for the photocatalytic alkylation step and general procedure GP6 for the thermal cleavage of the alkylated sulfonylhydrazide. After cleavage from the resin and ether precipitation, the crude peptide was purified by reverse phase HPLC (Shimadzu Preparative LCMS Nexera LC-40), (solvent system: A: MeCN and B: 5% TFA in H_2O), (25% B for 5 min, 25% to 75% B gradient over 16 min) and lyophilized to afford peptide **61** as a fluffy white solid (4.9 mg, 9% yield calculated from the original resin loading).

HRMS (ESI+) (m/z): $[M+3H]^{3+}$ calcd. for $C_{90}H_{123}N_{23}O_{18}$, 605.6550; found: 605.6567.

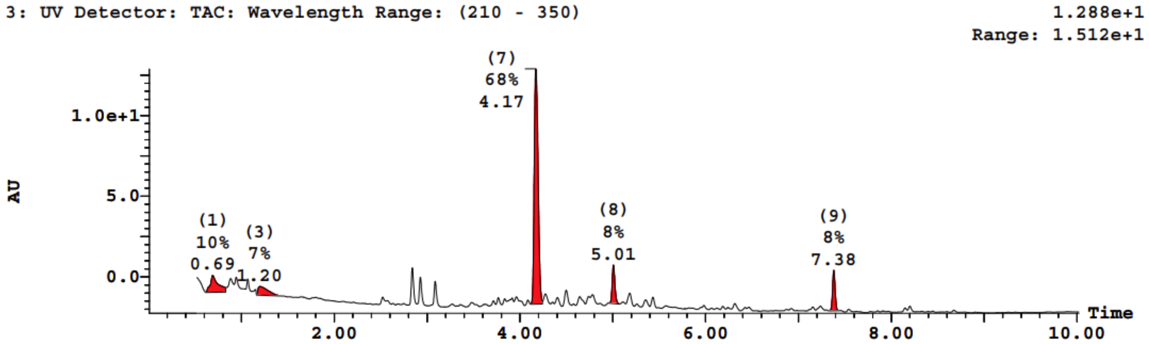

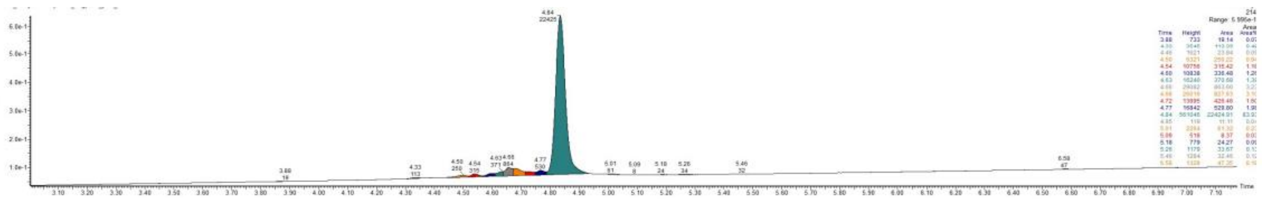

Supplementary Figure 24: **Up)** UPLC-MS Trace of the crude reaction mixture (product **61**) after full cleavage. Conditions: (10 to 50 % B over 10 min). **Down)** UPLC-MS Trace of the (product **61**) after purification. Conditions: (10% MeCN 1min; 10-60% 1-9 min; 60-95% 9-10 min).

8. Mechanistic investigation

8.1 UV-Vis Characterization

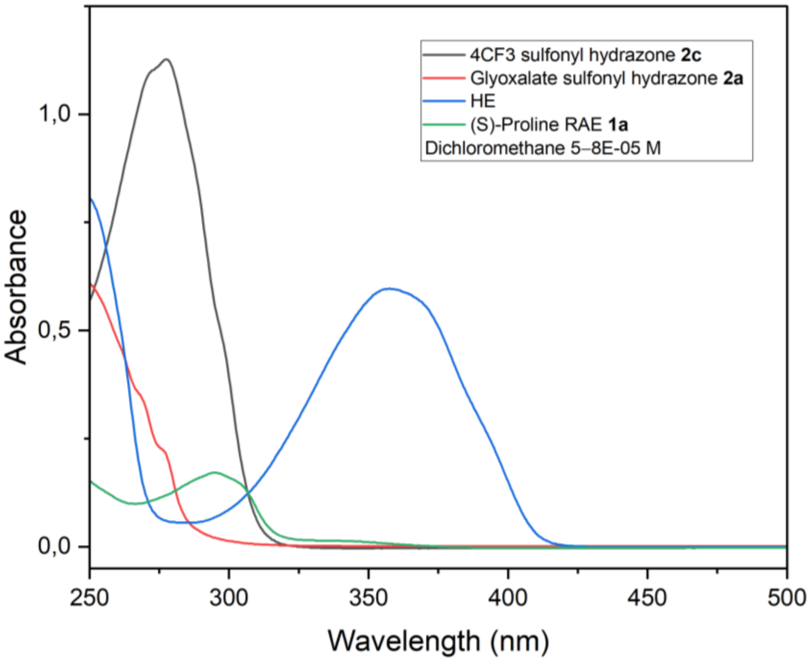

Supplementary Figure 25: Absorption spectra of each reaction component. The spectra were recorded $5 \cdot 10^{-5}$–$8 \cdot 10^{-5}$ M in dichlomethane in quartz cuvettes (optical path: 1 cm) with a bandwidth of 5 nm and a data pitch of 1 nm. Scan rate: medium.

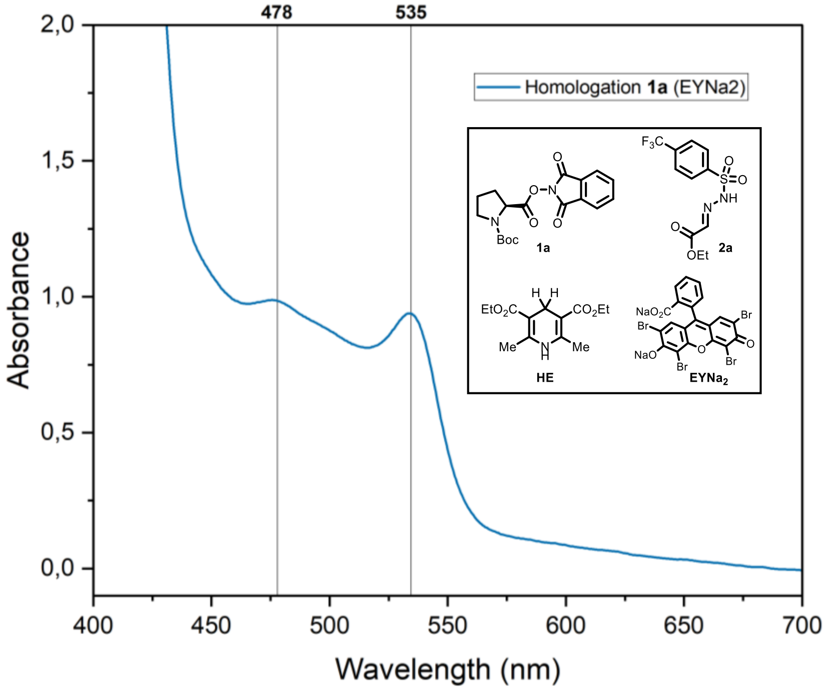

Supplementary Figure 26: Absorption spectrum of the optimized reaction condition for the C1 homologation of **1a** with glyoxalate-derived sulfonyl hydrazone **2a** using **EYNa₂** as PC. The spectrum was recorded in dichloromethane (after filtration of the unsoluble components) in quartz cuvettes (optical path: 1 cm) with a bandwidth of 5 nm and a data pitch of 1 nm. Scan rate: medium.

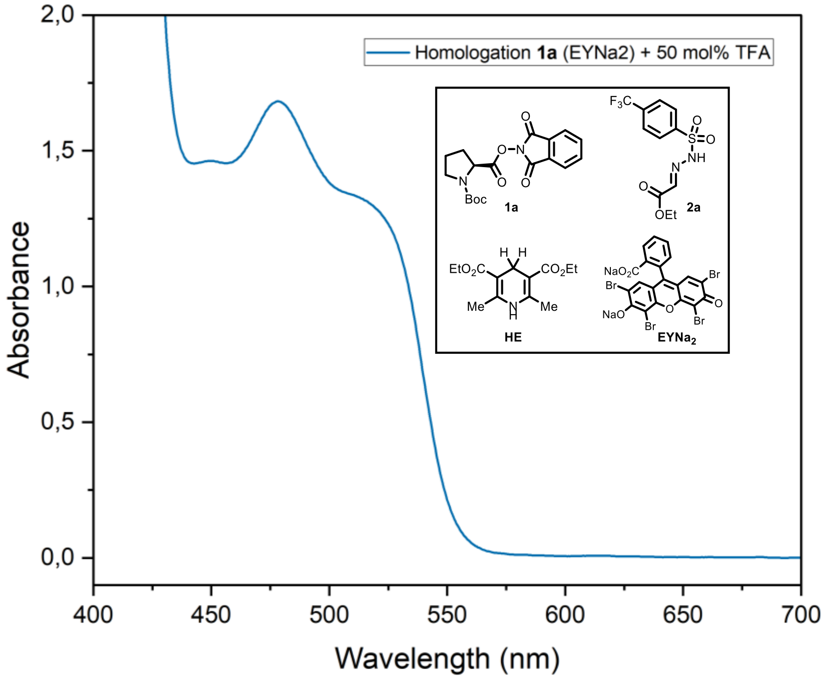

Supplementary Figure 27: Absorption spectrum of the reaction mixture for the C1 homologation of **1a** with glyoxalate-derived sulfonyl hydrazone **2a** using **EYNa**$_2$ as PC in presence of 50 mol% TFA. The spectrum was recorded in dichloromethane (after filtration of the unsoluble components) in quartz cuvettes (optical path: 1 cm) with a bandwidth of 5 nm and a data pitch of 1 nm. Scan rate: medium.

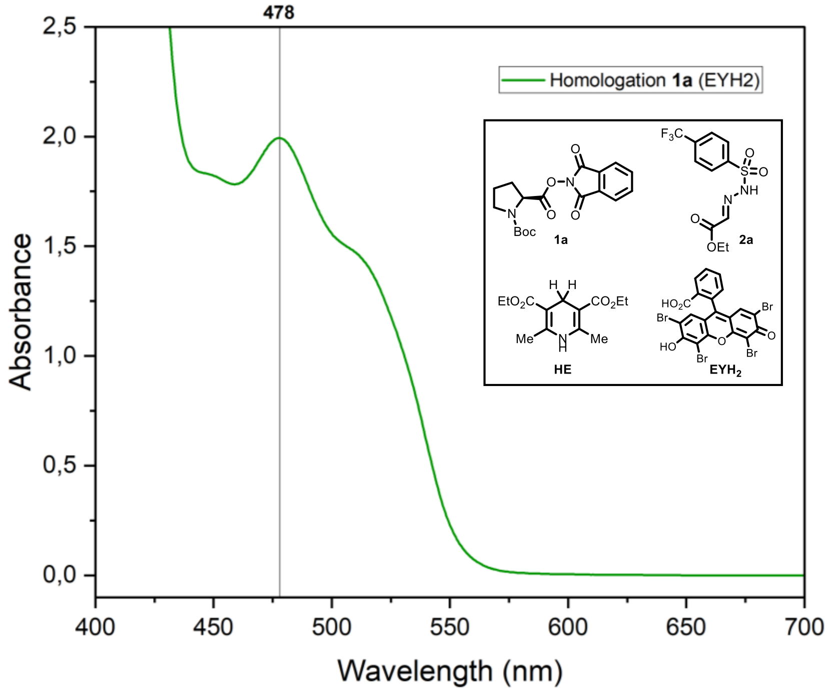

Supplementary Figure 28: Absorption spectrum of the reaction mixture for the C1 homologation of **1a** with glyoxalate-derived sulfonyl hydrazone **2a** using **EYH**$_2$ as PC. The spectrum was recorded in dichloromethane (after filtration of the unsoluble components) in quartz cuvettes (optical path: 1 cm) with a bandwidth of 5 nm and a data pitch of 1 nm. Scan rate: medium.

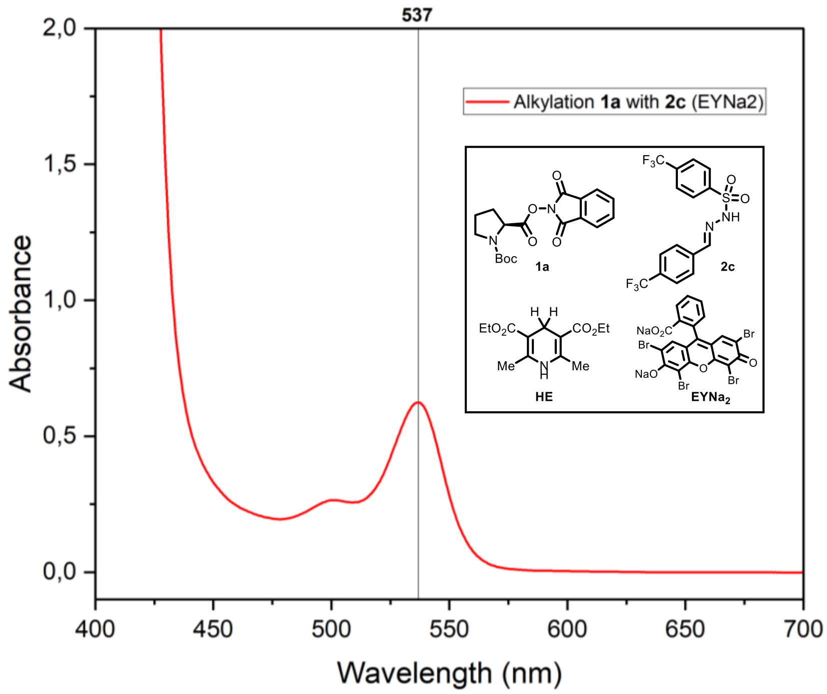

Supplementary Figure 29: Absorption spectrum of the reaction mixture for the alkylation of **1a** with arylsulfonyl hydrazone **2c** using **EYNa2** as PC. The spectrum was recorded in dichloromethane (after filtration of the unsoluble components) in quartz cuvettes (optical path: 1 cm) with a bandwidth of 5 nm and a data pitch of 1 nm. Scan rate: medium.

8.2 TEMPO Radical Trapping

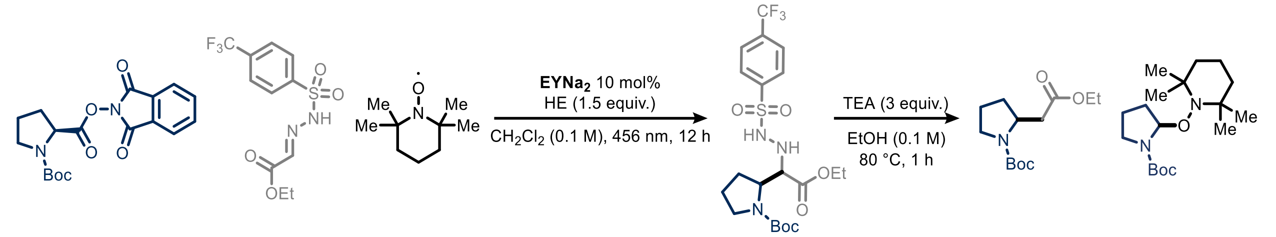

1a (1.0 equiv.) **2a** (2.0 equiv.) (3.0 equiv.) **int-a** **3** (n.d.) **61** (327.2659 $[M+H]^+$ found in ESI^+)

To an oven-dried 7 mL vial equipped with a stirring bar were added **1a** (0.300 mmol, 1.0 equiv.), **2a** (0.300 mmol, 1.0 equiv.), Hantzsch Ester (1.5 equiv.), **$EYNa_2$** (10 mol%) and TEMPO (3.0 equiv.) and the vial was sealed with a rubber septum. Subsequently, dry and degassed dichloromethane (3 mL) was added under N_2 atmosphere (0.1 M). The vial was stirred and irradiated in the UFO photochemical reactor (**See section 4**) for 12 h. The temperature was maintained at 30 °C during the course of the reaction. Then, the vial was removed from the photochemical reactor and the solvent was evaporated under reduced pressure. The obtained crude mixture was then dissolved in 3 mL of ethanol (0.1 M), TEA was added (3.0 equiv.) and the vial was placed in an oil bath at 80 °C for 1 h. The reaction mixture was cooled to rt and the solvent was removed under reduced pressure. The obtained crude was diluted with diethyl ether and washed with 1 M HCl. The combined organic layers were dried over Na_2SO_4, filtered and the solvent was removed under reduced pressure. The final crude reaction mixture was analyzed via ^{1}H-NMR using trichloroethylene as external standard and ESI-HRMS.

8.3 Radical Clock Experiment

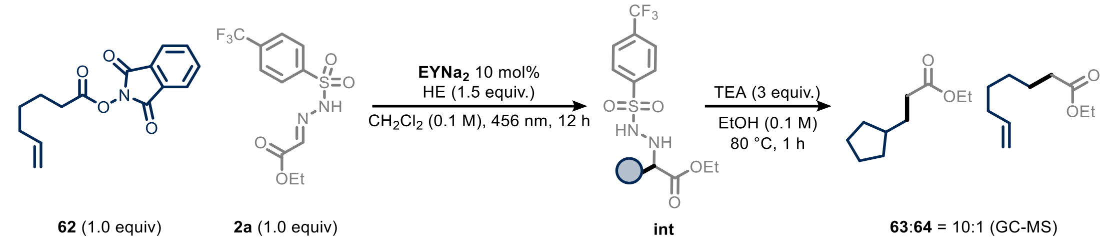

To an oven-dried 7 mL vial equipped with a stirring bar were added **62** (0.300 mmol, 1.0 equiv.), **2a** (0.300 mmol, 1.0 equiv.), Hantzsch Ester (1.5 equiv.), **EYNa₂** (10 mol%) and the vial was sealed with a rubber septum. Subsequently, dry and degassed dichloromethane (3 mL) was added under N_2 atmosphere (0.1 M). The vial was stirred and irradiated in the UFO photochemical reactor (**See section 4**) for 12 h. The temperature was maintained at 30 °C during the course of the reaction. Then, the vial was removed from the photochemical reactor and the solvent was evaporated under reduced pressure. The obtained crude mixture was then dissolved in 3 mL of ethanol (0.1 M), TEA was added (3.0 equiv.) and the vial was placed in an oil bath at 80°C for 1 h. The reaction mixture was cooled to rt and the solvent was removed under reduced pressure. The obtained crude was diluted with diethyl ether and washed with 1 M HCl. The combined organic layers were dried over Na_2SO_4, filtered and the solvent was removed under reduced pressure. The final crude reaction mixture was analyzed via ^{1}H-NMR using trichloroethylene as external standard and GC-MS.

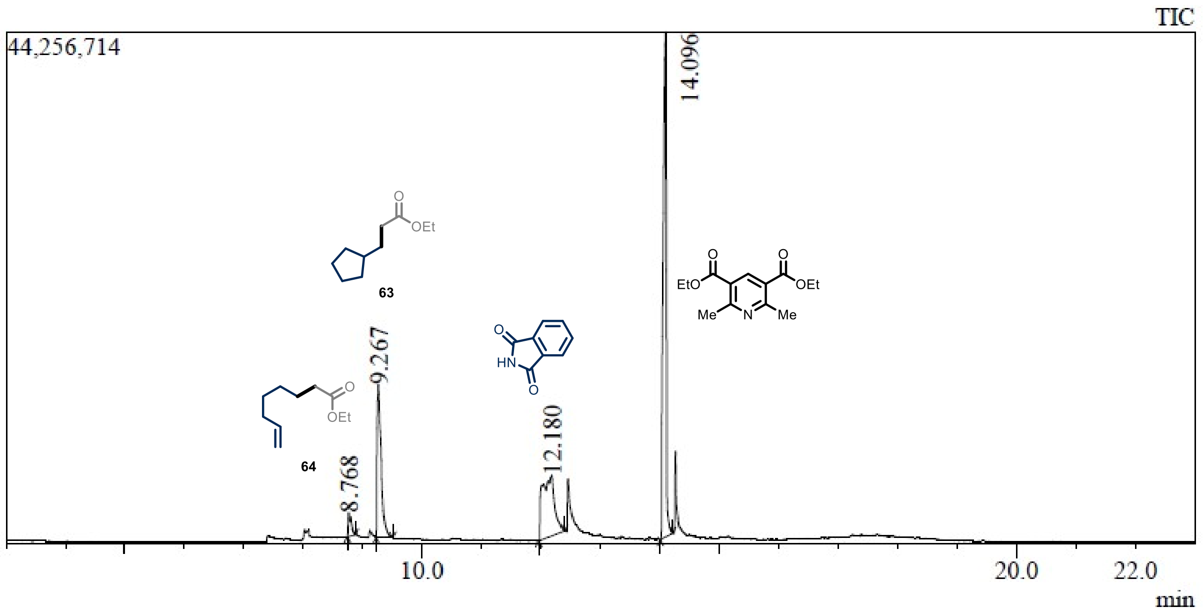

Supplementary Figure 30: GC trace of the crude reaction (formation of **63** and **64**).

9. Scale-up procedure for compounds 3 and 28

9.1 Scale-up in Batch

Supplementary Table 13. Set-ups for scale up in batch.

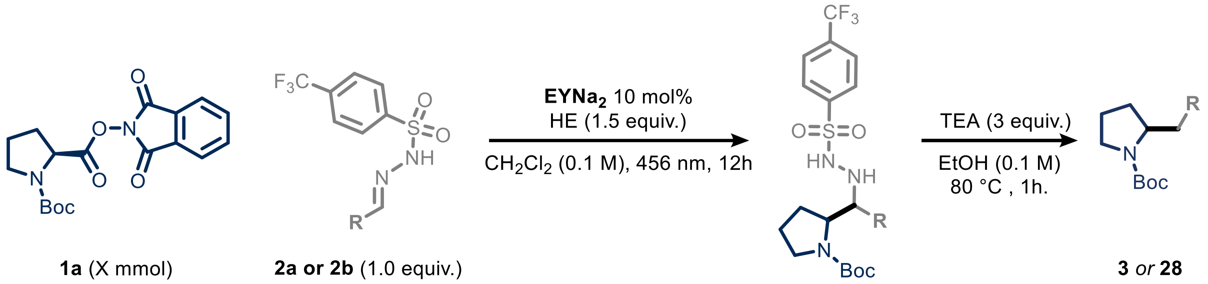

1a (X mmol) **2a or 2b** (1.0 equiv.) **3** *or* **28**

R	Set-Up	Temperature	Yield	Scale
-CO_2Et	A	35 °C	65%	1 mmol
-CO_2Et	B	30 °C	65%	1 mmol
-CO_2Et	B	35 °C	40%[b]	4 mmol[b]
-4CN(Ph)	A	35 °C	67%	1 mmol

[a]Determined via ^{1}H NMR using trichloroethylene as external standard.
[b]Isolated after column chromatography

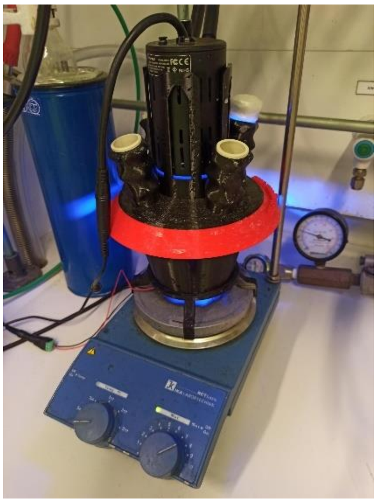

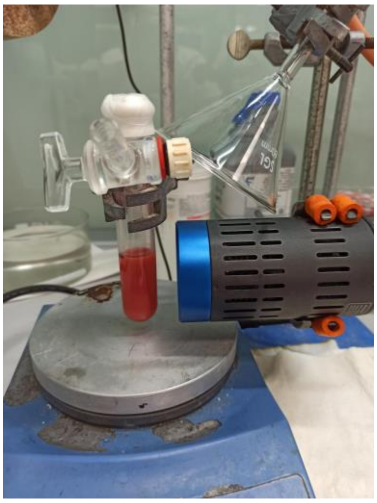

Supplementary Figure 31: Photochemical set-ups used for the scale up in batch. **(left)** Set-up A**. (right)** Set-up B.

9.2 Scale-up in Continuous Flow

Selected Entries of Optimization of the C1 Homologation in Flow

In a typical experiment, to an oven-dried 7 mL vial equipped with a stirring bar were added 4-(trifluoromethyl)sulfonyl hydrazone **2a** (65 mg, 0.20 mmol, 1 equiv.), **1a** (72 mg, 0.20 mmol, 1.0 equiv.), Hantzsch Ester (76 mg, 0.30 mmol, 1.5 equiv.) and the indicated **PC** (10 mol% or 1 mol%).and the vial was sealed with a rubber septum. Subsequently, dry and degassed solvent was added under N_2 atmosphere and the corresponding solution was taken up with a syringe. Finally, the syringe was mounted on a syringe pump and pushed into a Vapourtec UV-150 equipped blue LEDs (λ =450 nm, 60 W) for the required residence time. The outflow was collected in a 10 mL round-bottom flask. Then, the solvent was evaporated under reduced pressure. The obtained crude mixture was dissolved in 2 mL of ethanol (0.1 M), TEA was added (3.0 equiv.) and the vial was placed in an oil bath at 80°C for 1 h. The reaction mixture was cooled to rt and the solvent was removed under reduced pressure. The obtained crude was diluted with diethyl ether and washed with 1 M HCl. The combined organic layers were dried over Na_2SO_4, filtered and the solvent was removed under reduced pressure. The final crude reaction mixture was analyzed via ^{1}H-NMR using trichloroethylene as external standard.

Supplementary Table 14. Optimization of the C1 Homologation in flow.

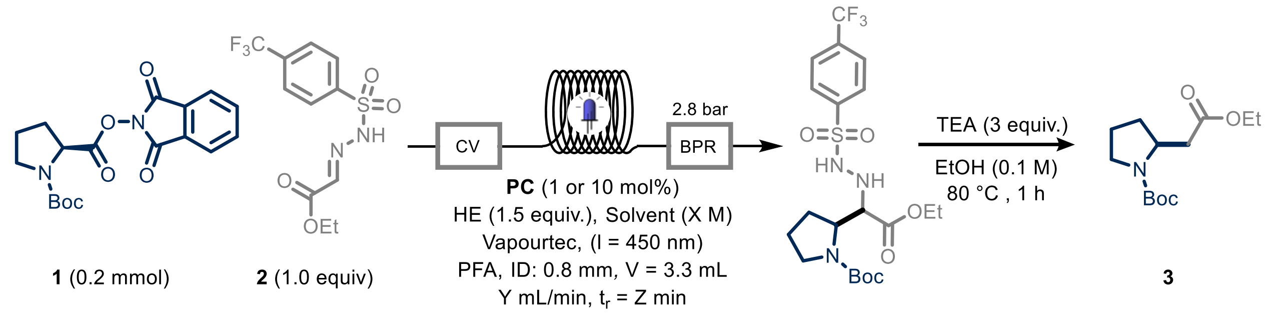

PC	Solvent	Concentration	τ_r	Yield of **3**[a]
$EYNa_2$	DCM	0.1 M	10 min	not soluble
$EYNa_2$	DCM	0.05 M	10 min	not soluble
EYH_2	DCM	0.05 M	10 min	not soluble
EYH_2	DCM:DMSO (3:1)	0.1 M	10 min	messy crude
EYH_2	DCM:THF (1:1)	0.1 M	10 min	messy crude
EYH_2	DCM:Acetone (8:1)	0.05 M	10 min	45%
EYH_2	DCM:Acetone (1:1)	0.05 M	10 min	messy crude
$Ru(bpy)_3(PF_6)_2$ 1 mol%	DMF	0.1 M	2.5 min	<10%[b]
$Ru(bpy)_3(PF_6)_2$ 1 mol%	DMF	0.1 M	8 min	50%[b]
$Ru(bpy)_3(PF_6)_2$ 1 mol%	DMF	0.1 M	15 min	<10%[b]
$Ru(bpy)_3(PF_6)_2$ 1 mol%	DMF:EtOH (1:1)	0.1 M	8 min	40%[c]
$Ru(bpy)_3(PF_6)_2$ 1 mol%	DCM:Acetone (1:1)	0.1 M	8 min	not soluble
$Ru(bpy)_3(PF_6)_2$ 1 mol%	DCM:Acetone (1:1)	0.05 M	8 min	65%

Reaction conditions: **1a**, **2c**, HE and **$EYNa_2$** in 2 mL of indicated solvent (0.1 M). [a]Determined via ^{1}H NMR using trichloroethylene as external standard. *Cleavage conditions:* Solvent switch to Ethanol (0.1 M), TEA (3 equiv.), 80 °C. [b]*Cleavage conditions*: DMF:EtOH (0.05 M), TEA (3 equiv.), 80 °C. [c]*Cleavage conditions*: DMF:EtOH (0.1 M), TEA (3 equiv.), 80 °C.

Scale-up (4 mmol) of Compound 3 in Flow

An oven dried 250 mL Schlenk flask, equipped with a magnetic stirring bar, was charged 4-(trifluoromethyl)sulfonyl hydrazone **2a** (1.30 g, 4.0 mmol, 1 equiv.), **1a** (1.44 g, 4.0 mmol, 1.0 equiv.), hantzsch ester (1.52 g, 6.0 mmol, 1.5 equiv.) and $Ru(bpy)_3(PF_6)_2$ (34 mg, 0.04 mmol, 1 mol%) and sealed with a rubber septum. Subsequently, dry and degassed CH_2Cl_2 (40 mL) and acetone (40 mL) were added under nitrogen atmosphere to prepare a 0.05 M solution (both the solvents were sparged with nitrogen for 20 min before the addition). The solution was taken up with a 60 mL syringe, mounted on a syringe pump and pushed into a Vapourtec UV-150 equipped blue LEDs (λ =450 nm, 60 W) for the required residence time. The outflow was collected in a 250 mL round-bottom flask.
Then, the solvent was evaporated under reduced pressure. The obtained crude mixture was dissolved in 40 mL of ethanol (0.1 M) in a flask equipped with a reflux condenser. TEA was added (3.0 equiv.) and the vessel was placed under stirring in an oil bath at 80 °C for 1 h (set-up connected to the schlenk line, see **Supplementary Figure 32 (right)**). The reaction mixture was cooled to rt and the solvent was removed under reduced pressure. The obtained crude was diluted with diethyl ether and washed with 1 M HCl. The combined organic layers were dried over Na_2SO_4, filtered and the solvent was removed under reduced pressure. The residue was purified via flash column chromatography on silica gel (Pentane:Ethyl Acetate 30:1) to afford the product as a colorless oil (620 mg, 60% yield).

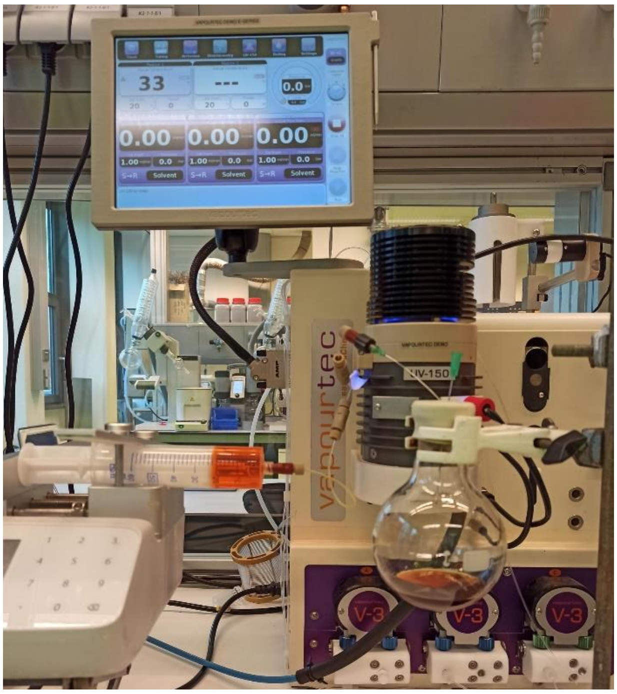

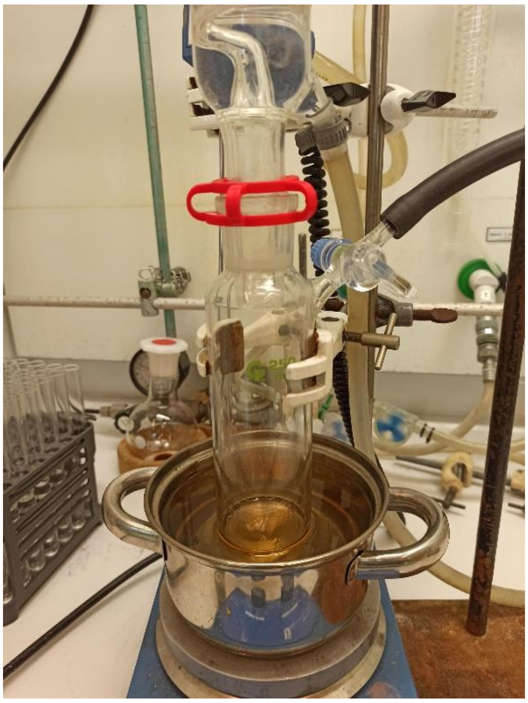

Supplementary Figure 32 (left). Photochemical step of the C1 homologation in Flow (450 nm, Vapourtec reactor). **(right).** Thermal cleavage step in batch (80 °C).

10. Characterization data of synthesized compound

10.1 Characterization of sulfonyl hydrazones 2d-2f, 2k-2m

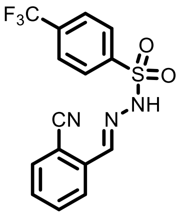

N'-(2-cyanobenzylidene)-4-(trifluoromethyl)benzenesulfonohydrazide (**2d**). Prepared according to GP1 using 4-(trifluoromethyl)benzenesulfonohydrazide (480 mg, 2.0 mmol, 1.0 equiv.) and 2-formylbenzonitrile (262 mg, 2.0 mmol, 1.0 equiv.). Isolated as a pale brown solid after filtration (1.53 mmol, 77% yield).

^{1}H NMR (400 MHz, DMSO-d_6) δ 12.29 (s, 1H), 8.21 (s, 1H), 8.13 (d, *J* = 8.2 Hz, 2H), 8.01 (d, *J* = 8.2 Hz, 2H), 7.87 (dd, *J* = 7.9, 4.1 Hz, 2H), 7.73 (t, *J* = 7.7 Hz, 1H), 7.58 (t, *J* = 7.6 Hz, 1H).
^{13}C NMR (101 MHz, DMSO-d_6) δ 143.3, 142.6, 135.6, 133.7, 133.5, 132.9 (q, *J* = 32.3 Hz), 130.6, 128.3, 126.6 (q, *J* = 3.7 Hz), 126.4, 123.4 (q, *J* = 273.0 Hz), 116.9, 110.3.
^{19}F NMR (282 MHz, DMSO-d_6) δ -56.98.
HRMS (ESI+) (m/z): $[M+H]^+$ calcd. for $C_{15}H_{10}F_3N_3O_2S$, 354.0524; found: 354.0515.

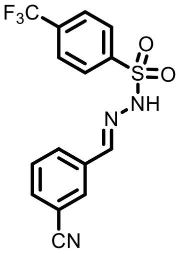

N'-(3-cyanobenzylidene)-4-(trifluoromethyl)benzenesulfonohydrazide (**2e**). Prepared according to GP1 using 4-(trifluoromethyl)benzenesulfonohydrazide (480 mg, 2.0 mmol, 1.0 equiv.) and 3-formylbenzonitrile (262 mg, 2.0 mmol, 1.0 equiv.). Isolated as a white solid after filtration (600 mg, 85% yield).

^{1}H NMR (400 MHz, DMSO-d_6) δ 12.06 (s, 1H), 8.13 (d, *J* = 8.2 Hz, 2H), 8.00 (dd, *J* = 5.6, 2.8 Hz, 4H), 7.92 (dt, *J* = 8.0, 1.4 Hz, 1H), 7.84 (dt, *J* = 7.9, 1.4 Hz, 1H), 7.59 (t, *J* = 7.8 Hz, 1H).
^{13}C NMR (101 MHz, DMSO-d_6) δ 145.7, 142.7, 134.7, 133.5, 132.8 (q, *J* = 32.4 Hz), 130.8, 130.6, 130.1, 128.2, 126.6 (q, *J* = 3.9 Hz), 123.4 (q, *J* = 272.9 Hz), 118.3, 112.0.
^{19}F NMR (282 MHz, DMSO-d_6) δ -61.76.
HRMS (FD+) (m/z): $[M]^+$ calcd. for $C_{15}H_{10}F_3N_3O_2S$, 353.0446; found: 353.0436.

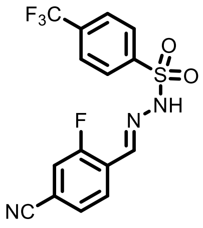

N'-(4-cyano-2-fluorobenzylidene)-4-(trifluoromethyl)benzenesulfonohydrazide (**2f**). Prepared according to GP1 using 4-(trifluoromethyl)benzenesulfonohydrazide (480 mg, 2.0 mmol, 1.0 equiv.) and 3-fluoro-4-formylbenzonitrile (298 g, 2.0 mmol, 1.0 equiv.). Isolated as a white solid after filtration (620 g, 84% yield).

^{1}H NMR (300 MHz, DMSO-d_6) δ 12.05 (s, 1H), 8.17 – 8.07 (m, 3H), 8.05 – 7.94 (m, 4H), 7.55 (t, *J* = 9.0 Hz, 1H).

^{13}C NMR (101 MHz, DMSO-d_6) δ 163.1 (d, J = 259.2 Hz), 144.8, 142.7, 133.6 (d, J = 9.1 Hz), 132.8 (q, J = 32.3 Hz), 132.4, 131.2 (d, J = 3.4 Hz), 128.3, 126.6 (q, J = 3.8 Hz), 123.4 (q, J = 272.9 Hz), 117.3 (d, J = 20.2 Hz), 113.5, 100.9 (d, J = 16.0 Hz).
^{19}F NMR (282 MHz, DMSO-d_6) δ -61.73, -106.35.
HRMS (ESI+) (m/z): $[M+H]^+$ calcd. for $C_{15}H_9F_4N_3O_2S$, 372.0430; found: 372.0426.

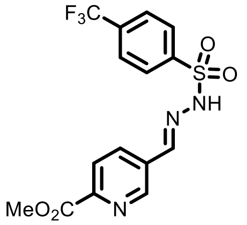

methyl (E)-5-((2-((4-(trifluoromethyl)phenyl)sulfonyl)hydrazineylidene)methyl)picolinate (**2k**). Prepared according to GP1 using 4-(trifluoromethyl)benzenesulfonohydrazide (360 mg, 1.5 mmol, 1.0 equiv.) and methyl 5-formylpicolinate (248 mg, 1.5 mmol, 1.0 equiv.). Isolated as a white solid after filtration (535 mg, 92% yield).

^{1}H NMR (500 MHz, DMSO-d_6) δ 12.25 (bs, 1H), 8.86 (d, J = 2.1 Hz, 1H), 8.21 – 7.93 (m, 7H), 3.87 (s, 3H).
^{13}C NMR (126 MHz, DMSO-d_6) δ 164.7, 148.4, 147.9, 144.1, 142.6, 134.6, 132.8 (q, J = 32.5 Hz), 132.3, 128.2, 126.6 (q, J = 3.8 Hz), 124.9, 123.4 (d, J = 272.9 Hz), 52.5.
^{19}F NMR (470 MHz, DMSO-d_6) δ -61.78.
HRMS (ESI+) (m/z): $[M+H]^+$ calcd. for $C_{15}H_{12}F_3N_3O_4S$, 388.0579; found: 388.0571.

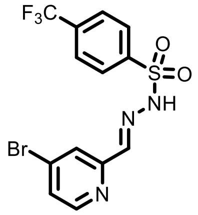

N'-((4-bromopyridin-2-yl)methylene)-4-(trifluoromethyl)benzenesulfonohydrazide (**2l**). Prepared according to GP1 using 4-(trifluoromethyl)benzenesulfonohydrazide (480 mg, 2.0 mmol, 1.0 equiv.) and 4-bromopicolinaldehyde (372 mg, 2.0 mmol, 1.0 equiv.). Isolated as a pale pink solid after filtration (432 mg, 53% yield).

^{1}H NMR (400 MHz, DMSO-d_6) δ 12.31 (s, 1H), 8.44 (d, J = 5.3 Hz, 1H), 8.12 (d, J = 8.2 Hz, 2H), 8.02 (d, J = 8.3 Hz, 2H), 7.93 (s, 1H), 7.88 (d, J = 1.9 Hz, 1H), 7.67 (dd, J = 5.3, 2.0 Hz, 1H).
^{13}C NMR (101 MHz, DMSO-d_6) δ 153.6, 150.8, 146.3, 142.6, 132.9 (q, J = 32.4 Hz), 132.8, 128.2, 127.5, 126.71 (q, J = 3.8 Hz), 123.3 (q, J = 273.0 Hz) 122.5.
^{19}F NMR (282 MHz, DMSO-d_6) δ -61.76.
HRMS (ESI+) (m/z): $[M+H]^+$ calcd. for $C_{13}H_9BrF_3N_3O_2S$, 409.9609; found: 409.9593.

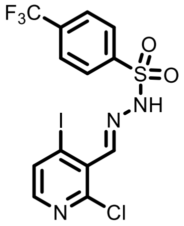

(E)-N'-((2-chloro-4-iodopyridin-3-yl)methylene)-4-(trifluoromethyl)benzenesulfonohydrazide (**2m**). Prepared according to GP1 using 4-(trifluoromethyl)benzenesulfonohydrazide (360 mg, 1.5 mmol, 1.0 equiv.) and 2-chloro-4-iodonicotinaldehyde (401 mg, 1.5 mmol, 1.0 equiv.). Isolated as a white solid after filtration (624 mg, 85% yield).

^{1}**H NMR** (400 MHz, DMSO-d_6) δ 12.33 (s, 1H), 8.10 (d, *J* = 8.3 Hz, 2H), 8.02 (d, *J* = 7.9 Hz, 2H), 8.00 – 7.93 (m, 3H).
^{13}**C NMR** (101 MHz, DMSO-d_6) δ 149.5, 148.3, 145.9, 142.9, 134.4, 132.9 (q, *J* = 32 Hz), 131.5, 128.4, 126.6 (q, *J* = 4 Hz), 123.4 (q, *J* = 273 Hz). 111.6.
^{19}**F NMR** (282 MHz, DMSO-d_6) δ -61.70.
HRMS (ESI+) (m/z): $[M+H]^+$ calcd. for $C_{13}H_8ClF_3IN_3O_2S$, 489.9101; found: 489.9109.

10.2 Characterization of NHPI esters 1c, 1e, 1f, 1t, 1u, 1ab-1af

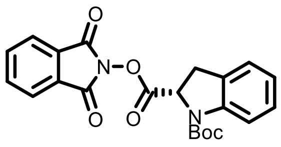

1-(tert-butyl) 2-(1,3-dioxoisoindolin-2-yl) (S)-indoline-1,2-dicarboxylate **(1c).** Prepared according to GP2 from (S)-1-(tert-butoxycarbonyl)indoline-2-carboxylic acid (526 mg, 2.0 mmol, 1.0 equiv.), 2-hydroxyisoindoline-1,3-dione (326 mg, 2 mmol, 1.0 equiv.), N,N-dimethylpyridin-4-amine (24.4 mg, 0.2 mmol, 0.1 equiv.) and diisopropylmethanediimine (313 µL, 2.0 mmol, 1.0 equiv.). Purified via flash column chromatography on silica gel (from Heptane to Heptane:Ethyl Acetate 3:1) to afford the product as a white solid (768 mg, 94% yield).

^{1}**H NMR** (500 MHz, $CDCl_3$) δ 7.98 – 7.78 (m, 5H), 7.32 – 7.19 (m, 2H), 7.03 (t, *J* = 7.5 Hz, 1H), 5.26 (dd, *J* = 11.8, 4.5 Hz, 1H), 3.77 (dd, *J* = 16.8, 11.8 Hz, 1H), 3.57 (dd, *J* = 16.9, 4.6 Hz, 1H), 1.65 (s, 9H).
^{13}**C NMR** (126 MHz, $CDCl_3$) δ 168.5, 161.7, 151.2, 142.2, 135.0, 128.9, 128.2, 127.3, 124.7, 124.1, 123.0, 114.8, 82.8, 58.5, 33.1, 28.1.
HRMS (ESI+) (m/z): $[M+H]^+$ calcd. for $C_{22}H_{20}N_2O_6$, 409.1400; found: 409.1401

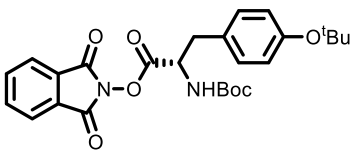

1, 3-dioxoisoindolin-2-yl (S)-2-((tert-butoxycarbonyl)amino)-3-(thiophen-2-yl)propanoate **(1e).** Prepared according to GP2 from 3-(4-(tert-butoxy)phenyl)-2-((tert-butoxycarbonyl)amino)propanoic acid (1.69 g, 5.0 mmol, 1.0 equiv.), 2-hydroxyisoindoline-1,3-dione (816 mg, 5.0 mmol, 1.0 equiv.), N,N-dimethylpyridin-4-amine (61.1 mg, 0.5 mmol, 0.1 equiv.) and diisopropylmethanediimine (783 µL, 5.0 mmol, 1.0 equiv.). Purified via flash column chromatography on silica gel (from Pentane:Ethyl Acetate 20:1 to 3:1) to afford the product as a white solid (1.95 g, 81% yield).

^{1}**H NMR** (400 MHz, $CDCl_3$) δ 7.88 (dd, *J* = 5.5, 3.1 Hz, 2H), 7.78 (dd, *J* = 5.5, 3.1 Hz, 2H), 7.20 (d, *J* = 8.4 Hz, 2H), 6.95 (d, *J* = 8.5 Hz, 2H), 5.08 – 4.57 (m, 2H), 3.38 – 3.03 (m, 2H), 1.41 (s, 9H), 1.32 (s, 9H).
^{13}**C NMR** (101 MHz, $CDCl_3$) δ 168.8, 161.5, 154.8, 134.9, 130.3, 129.6, 128.9, 124.3, 124.1, 80.5, 78.5, 52.8, 37.7, 28.9, 28.3.
HRMS (ESI+) (m/z): $[M+H]^+$ calcd. for $C_{26}H_{30}N_2O_7$, 483.2131; found: 483.2134.

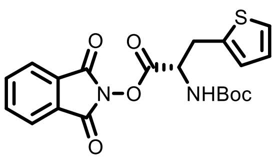

1, 3-dioxoisoindolin-2-yl (S)-2-((tert-butoxycarbonyl)amino)-3-(thiophen-2-yl)propanoate **(1f).** Prepared according to GP2 from (S)-2-((tert-butoxycarbonyl)amino)-3-(thiophen-2-yl)propanoic acid (543 mg, 2.0 mmol, 1.0 equiv.), 2-hydroxyisoindoline-1,3-dione (326 mg, 2.0 mmol, 1.0 equiv.), N,N-dimethylpyridin-4-amine (24.4 mg, 0.2 mmol, 0.1 equiv.) and diisopropylmethanediimine (313 µL, 2.0 mmol, 1.0 equiv.). Purified via flash column

chromatography on silica gel (from Pentane:Ethyl Acetate 10:1 to 2:1) to afford the product as a white solid (566 mg, 68% yield).

^{1}H NMR (400 MHz, $CDCl_3$) δ 7.87 (dd, *J* = 5.5, 3.1 Hz, 2H), 7.78 (dd, *J* = 5.6, 3.1 Hz, 2H), 7.20 (d, *J* = 5.1 Hz, 1H), 7.07 (d, *J* = 3.5 Hz, 1H), 6.98 (dd, *J* = 5.2, 3.5 Hz, 1H), 5.21 – 4.61 (m, 2H), 3.65 – 3.23 (m, 2H), 1.44 (s, 9H).
^{13}C NMR (101 MHz, $CDCl_3$) δ 168.3, 161.5, 154.7, 136.1, 135.0, 128.8, 127.7, 127.4, 125.3, 124.1, 80.7, 52.8, 32.4, 28.3.
HRMS Failed to find the mass due to instability of the NHPI redox active ester.

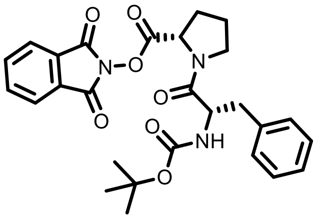

***1,3-dioxoisoindolin-2-yl (tert-butoxycarbonyl)-L-methionyl-L-prolinate* (1t).** Prepared according to GP2 from (tert-butoxycarbonyl)-L-phenylalanyl-L-prolineproline (1.14 g, 1.0 mmol, 1.0 equiv.), 2-hydroxyisoindoline-1,3-dione (163 mg, 1.0 mmol, 1.0 equiv.), N,N-dimethylpyridin-4-amine (12.2 mg, 0.1 mmol, 0.1 equiv.) and diisopropylmethanediimine (157 µL, 1.0 mmol, 1.0 equiv.). Purified via flash column chromatography on silica gel (Dichloromethane:MeOH 95:5 to 90:10) to afford the product as a white solid (238 mg, 47% yield).

^{1}H NMR (400 MHz, $CDCl_3$) δ 7.35 (dd, *J* = 5.5, 3.1 Hz, 2H), 7.26 – 7.20 (m, 2H), 6.72 – 6.57 (m, 5H), 4.84 (d, *J* = 8.9 Hz, 1H), 4.30 (dd, *J* = 8.6, 4.6 Hz, 1H), 4.08 (t, *J* = 7.8 Hz, 1H), 3.05 (dt, *J* = 9.8, 7.3 Hz, 1H), 2.57 – 2.45 (m, 2H), 2.39 (dd, *J* = 13.6, 6.3 Hz, 1H), 1.85 – 1.63 (m, 2H), 1.50 – 1.38 (m, 2H), 0.84 (s, 9H).
^{13}C NMR (101 MHz, $CDCl_3$) δ 171.2, 168.4, 161.7, 155.4, 136.3, 134.9, 129.8, 129.1, 128.6, 126.9, 124.2, 79.9, 57.1, 53.6, 46.8, 39.5, 29.5, 28.5, 25.1.
HRMS (ESI+) (m/z): $[M+H]^+$ calcd. for $C_{27}H_{29}N_3O_7$, 508.2084; found: 508.2081

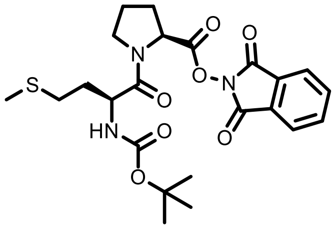

***1,3-dioxoisoindolin-2-yl (tert-butoxycarbonyl)-L-methionyl-L-prolinate* (1u).** Prepared according to GP2 from (tert-butoxycarbonyl)-L-methionyl-L-proline (1.14 g, 3.3 mmol, 1.0 equiv.), 2-hydroxyisoindoline-1,3-dione (537 mg, 3.3 mmol, 1.0 equiv.), N,N-dimethylpyridin-4-amine (40.2 mg, 0.33 mmol, 0.1 equiv.) and diisopropylmethanediimine (510 µL, 3.3 mmol, 1.0 equiv.). Purified via flash column chromatography on silica gel (gradient from Dichloromethane:MeOH 100:0 to 98:2) to afford the product as a yellow solid (924 mg, 57% yield).

^{1}H NMR (400 MHz, $CDCl_3$) δ 7.86 (dd, *J* = 5.5, 3.1 Hz, 2H), 7.78 (dd, *J* = 5.5, 3.1 Hz, 2H), 5.41 (d, *J* = 8.9 Hz, 1H), 4.89 (dd, *J* = 8.6, 4.8 Hz, 1H), 4.69 – 4.56 (m, 1H), 3.89 – 3.71 (m, 2H), 2.56 (t, *J* = 7.2 Hz, 2H), 2.49 – 2.28 (m, 2H), 2.27 – 2.07 (m, 2H), 2.06 (s, 3H), 2.05 – 1.96 (m, 1H), 1.94 – 1.81 (m, 1H), 1.41 (s, 9H).
^{13}C NMR (101 MHz, $CDCl_3$) δ 171.3, 168.5, 161.6, 155.7, 134.9, 129.0, 124.1, 80.0, 57.0, 51.0, 47.1, 32.4, 29.9, 29.4, 28.4, 25.2, 15.7.
HRMS (ESI+) (m/z): $[M+H]^+$ calcd. for $C_{23}H_{29}N_3O_7S$, 492.1804; found: 492.1794.

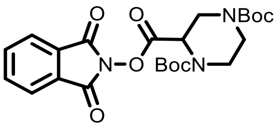

***1,4-di-tert-butyl 2-(1,3-dioxoisoindolin-2-yl) piperazine-1,2,4-tricarboxylate* (1ab).** Prepared according to GP2 from 1,4-bis(tert-butoxycarbonyl)piperazine-2-carboxylic acid (1.35 g, 4.1 mmol, 1.0 equiv.), 2-hydroxyisoindoline-1,3-dione (667 mg, 4.1 mmol, 1.0 equiv.), N,N-dimethylpyridin-4-amine (50.0 mg, 0.41 mmol,

0.1 equiv.) and diisopropylmethanediimine (633 µL, 4.1 mmol, 1.0 equiv.). Purified via flash column chromatography on silica gel (gradient Dichloromethane:Diethyl Ether) to afford the product (mixture of rotamers) as a white solid (856 mg, 44% yield).

^{1}H NMR (400 MHz, $CDCl_3$) δ 7.89 – 7.81 (m, 2H), 7.81 – 7.73 (m, 2H), 5.21 & 4.97 (rotameric m, 1H), 4.79 – 4.54 (m, 1H), 4.25 – 3.76 (m, 2H), 3.37 – 3.12 (m, 2H), 3.10 – 2.69 (m, 1H), 1.54 – 1.40 (m, 18H).
^{13}C NMR (101 MHz, $CDCl_3$) δ 167.2 & 166.9 (rotameric signals), 161.3, 155.0 & 154.7 (rotameric signals), 154.2, 134.9, 129.0, 124.0, 81.9, 80.8, 53.7 & 52.4 (rotameric signals), 44.3 & 43.4 (rotameric signals), 42.2 & 41.7 (rotameric signals), 40.4, 28.3, 28.1.
HRMS (ESI+) (m/z): $[M+H]^+$ calcd. for $C_{23}H_{29}N_3O_8$, 476.2033; found: 476.2036.

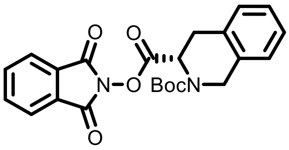

2-(tert-butyl) 3-(1,3-dioxoisoindolin-2-yl) (S)-3,4-dihydroisoquinoline-2,3(1H)-dicarboxylate **(1ac).** Prepared according to GP2 from (S)-2-(tert-butoxycarbonyl)-1,2,3,4-tetrahydroisoquinoline-3-carboxylic acid (1.14 g, 4.1 mmol, 1.0 equiv.), 2-hydroxyisoindoline-1,3-dione (667 mg, 4.1 mmol, 1.0 equiv.), N,N-dimethylpyridin-4-amine (50.0 mg, 0.41 mmol, 0.1 equiv.) and diisopropylmethanediimine (633 µL, 4.1 mmol, 1.0 equiv.). Purified via flash column chromatography on silica gel (gradient Dichloromethane:Diethyl Ether) to afford the product (mixture of rotamers) as a white solid (814 mg, 47% yield).

^{1}H NMR (400 MHz, $CDCl_3$) δ 7.88 – 7.78 (m, 2H), 7.78 – 7.69 (m, 2H), 7.33 – 7.10 (m, 4H), 5.54 & 5.10 (rotameric t, J_{minor} = 4.8 Hz, J_{major} = 5.7 Hz, 1H), 4.81 – 4.58 (m, 2H), 3.45 – 3.27 (m, 2H), 1.57 – 1.52 (m, 9H).
^{13}C NMR (101 MHz, $CDCl_3$) δ 168.9 & 168.3 (rotameric signals), 161.6 & 161.5 (rotameric signals), 155.0 & 154.5 (rotameric signals), 134.9 & 134.8 (rotameric signals), 134.4 & 132.8 (rotameric signals), 131.7 & 131.2 (rotameric signals), 129.0 & 128.8 (rotameric signals), 128.0, 127.5 & 127.2 (rotameric signals), 127.5 & 127.1 (rotameric signals), 126.4 & 126.3 (rotameric signals), 124.0, 82.0 & 81.4 (rotameric signals), 53.4 & 51.6 (rotameric signals), 44.7 & 44.1 (rotameric signals), 32.2 & 31.5 (rotameric signals), 28.5 & 28.3 (rotameric signals).
HRMS Failed to find the mass due to instability of the NHPI redox active ester.

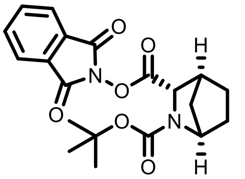

2-(tert-butyl) 3-(1,3-dioxoisoindolin-2-yl) (1R,3S,4S)-2-azabicyclo[2.2.1]heptane-2,3-dicarboxylate **(1ad).** Prepared according to GP2 from (1R,3S,4S)-2-(tert-Butoxycarbonyl)-2-azabicyclo[2.2.1]heptane-3-carboxylic acid (1.00 g, 4.1 mmol, 1.0 equiv.), 2-hydroxyisoindoline-1,3-dione (667 mg, 4.1 mmol, 1.0 equiv.), N,N-dimethylpyridin-4-amine (50.0 mg, 0.41 mmol, 0.1 equiv.) and diisopropylmethanediimine (633 µL, 4.1 mmol, 1.0 equiv.). Purified via flash column chromatography on silica gel (gradient Dichloromethane:Diethyl Ether) to afford the product (mixture of rotamers) as a pale brown solid (1.33 mg, 83% yield).

^{1}H NMR (400 MHz, $CDCl_3$) δ 7.91 – 7.83 (m, 2H), 7.82 – 7.73 (m, 2H), 4.40 & 4.24 (rotameric s, 1H), 4.22 & 4.10 (rotameric s, 1H), 3.05 – 2.97 (m, 1H), 2.05 – 1.96 (m, 1H), 1.92 – 1.74 (m, 2H), 1.74 – 1.51 (m, 2H), 1.48 & 1.46 (rotameric s, 9H), 1.44 – 1.33 (m, 1H).
^{13}C NMR (101 MHz, $CDCl_3$) δ 167.6 & 167.5 (rotameric signals), 161.8 & 161.7 (rotameric signals), 154.1 & 153.0 (rotameric signals), 134.9 & 134.8 (rotameric signals), 129.1 & 129.0 (rotameric signals), 124.1 & 124.0 (rotameric signals), 81.1 & 80.4 (rotameric signals), 62.4 & 62.2 (rotameric signals), 57.7 & 56.5 (rotameric signals), 43.6 & 42.7 (rotameric signals), 35.9 & 35.1 (rotameric signals), 30.6 & 30.3 (rotameric signals), 28.5 & 28.3 (rotameric signals), 28.0 & 27.7 (rotameric signals).
HRMS (ESI+) (m/z): $[M+H]^+$ calcd. for $C_{20}H_{22}N_2O_6$, 387.1556; found: 387.1554.

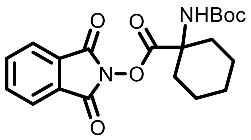

1,3-dioxoisoindolin-2-yl 1-((tert-butoxycarbonyl)amino)cyclohexane-1-carboxylate **(1ae).** Prepared according to GP2 from 1-((tert-butoxycarbonyl)amino)cyclohexane-1-carboxylic acid (1.00 g, 4.1 mmol, 1.0 equiv.), 2-hydroxyisoindoline-1,3-dione (667 mg, 4.1 mmol, 1.0 equiv.), N,N-dimethylpyridin-4-amine (50.0 mg, 0.41 mmol, 0.1 equiv.) and diisopropylmethanediimine (633 µL, 4.1 mmol, 1.0 equiv.). Purified via flash column chromatography on silica gel (gradient Dichloromethane:Diethyl Ether) to afford the product as a white solid (1.12 g, 70% yield).

^{1}H NMR (400 MHz, $CDCl_3$) δ 7.85 (dd, *J* = 5.5, 3.1 Hz, 2H), 7.76 (dd, *J* = 5.5, 3.1 Hz, 2H), 4.94 (s, 1H), 2.20 – 2.02 (m, 4H), 1.77 – 1.64 (m, 2H), 1.64 – 1.55 (m, 2H), 1.51 (s, 9H), 1.46 – 1.32 (m, 1H).
^{13}C NMR (101 MHz, $CDCl_3$) δ 170.8, 161.9, 154.5, 134.7, 129.2, 123.9, 80.8, 58.5, 33.3, 28.3, 25.2, 21.2.
HRMS (ESI+) (m/z): $[M+H]^+$ calcd. for $C_{20}H_{24}N_2O_6$, 389.1713; found: 389.1705.

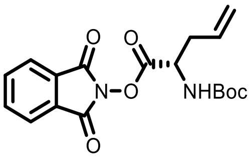

1,3-dioxoisoindolin-2-yl (S)-2-((tert-butoxycarbonyl)amino)pent-4-enoate **(1af).** Prepared according to GP2 from (S)-2-((tert-butoxycarbonyl)amino)pent-4-enoic acid (431 mg, 2.0 mmol, 1.0 equiv.), 2-hydroxyisoindoline-1,3-dione (326 mg, 2 mmol, 1.0 equiv.), N,N-dimethylpyridin-4-amine (24.4 mg, 0.2 mmol, 0.1 equiv.) and diisopropylmethanediimine (313 µL, 2.0 mmol, 1.0 equiv.). Purified via flash column chromatography on silica gel (from Heptane to Heptane:Ethyl Acetate 6:1) to afford the product (mixture of rotamers) as a white solid (504 mg, 70% yield).

^{1}H NMR (500 MHz, $CDCl_3$) δ 7.88 (dd, *J* = 5.5, 3.1 Hz, 2H), 7.79 (dd, *J* = 5.5, 3.1 Hz, 2H), 5.92 – 5.80 & 5.78 – 5.69 (rotameric m, 1H), 4.85 – 4.77 (m, 4H), 2.76 – 2.70 & 2.63 – 2.47 (rotameric m, 2H), 1.54 – 1.36 (m, 9H).
^{13}C NMR (126 MHz, $CDCl_3$) δ 168.88, 161.60, 154.93, 134.98, 131.24, 128.96, 124.15, 120.70, 82.19 & 80.72 (rotameric signals), 52.77 & 51.45 (rotameric signals), 36.98 & 36.33 (rotameric signals), 28.38 & 28.12 (rotameric signals).
HRMS Failed to find the mass due to instability of the NHPI redox active ester.

10.3 Characterization of compounds 3-26 (C1 homologation)

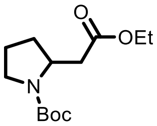

tert-butyl 2-(2-ethoxy-2-oxoethyl)pyrrolidine-1-carboxylate **(3).** Prepared according to GP3 from **1a** (108 mg, 0.3 mmol, 1.0 equiv.) and **2a** (97 mg, 0.3 mmol, 1.0 equiv.). Purified via flash column chromatography on silica gel (Pentane:Ethyl Acetate 40:1) to afford the product (mixture of rotamers) as a colorless oil (62 mg, 80% yield).

Characterization data are in accordance with literature.[4]
^{1}H NMR (400 MHz, $CDCl_3$) δ 4.11 (d, *J* = 7.2 Hz, 3H), 3.42 – 3.23 (m, 2H), 3.01 – 2.70 (m, 1H), 2.28 (dd, *J* = 15.0, 9.9 Hz, 1H), 2.11 – 1.96 (m, 1H), 1.88 – 1.67 (m, 3H), 1.45 (s, 9H), 1.24 (t, *J* = 7.1 Hz, 3H).
^{13}C NMR (101 MHz, $CDCl_3$) δ 171.7, 154.4, 79.7 & 79.4 (rotameric signals), 60.5, 54.2, 46.7 & 46.3 (rotameric signals), 39.5 & 38.7 (rotameric signals), 31.4 & 30.6 (rotameric signals), 28.6, 23.6 & 22.9 (rotameric signals), 14.3.

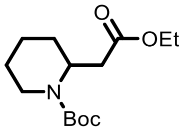

***tert-butyl 2-(2-ethoxy-2-oxoethyl)piperidine-1-carboxylate* (4).** Prepared according to GP3 from **1b** (112 mg, 0.3 mmol, 1.0 equiv.) and **2a** (97 mg, 0.3 mmol, 1.0 equiv.). Purified via flash column chromatography on silica gel (from Pentane:Ethyl Acetate 40:1 to 15:1) to afford the product as a colorless oil (63 mg, 77% yield).

Characterization data are in accordance with literature.[4]
^{1}H NMR (500 MHz, $CDCl_3$) δ 4.68 (d, *J* = 8.1 Hz, 1H), 4.10 (q, *J* = 7.1 Hz, 2H), 3.97 (d, *J* = 13.2 Hz, 1H), 2.76 (t, *J* = 13.3 Hz, 1H), 2.61 – 2.49 (m, 2H), 1.61 (d, *J* = 12.2 Hz, 4H), 1.54 – 1.35 (m, 11H), 1.23 (t, *J* = 7.1 Hz, 3H).
^{13}C NMR (126 MHz, $CDCl_3$) δ 171.5, 154.8, 79.6, 60.6, 48.0, 39.3, 35.4, 28.5, 28.3, 25.4, 19.0, 14.3.

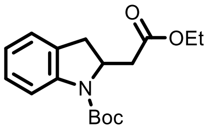

***tert-butyl 2-(2-ethoxy-2-oxoethyl)indoline-1-carboxylate* (5).** Prepared according to GP3 from **1c** (123 mg, 0.3 mmol, 1.0 equiv.) and **2a** (97 mg, 0.3 mmol, 1.0 equiv.). Purified via flash column chromatography on silica gel (from Pentane:Ethyl Acetate 20:1 to 10:1) to afford the product as a colorless oil (50 mg, 55% yield).

^{1}H NMR (500 MHz, $CDCl_3$) δ 7.87 – 7.33 (m, 1H), 7.19 – 7.10 (m, 2H), 6.94 (t, *J* = 7.5 Hz, 1H), 4.86 – 4.68 (m, 1H), 4.16 – 4.05 (m, 2H), 3.40 (dd, *J* = 16.4, 9.7 Hz, 1H), 2.94 – 2.77 (m, 2H), 2.51 (dd, *J* = 15.2, 9.9 Hz, 1H), 1.57 (s, 9H), 1.22 (t, *J* = 7.1 Hz, 3H).
^{13}C NMR (126 MHz, $CDCl_3$) δ 171.2, 152.1, 141.8, 129.8, 127.6, 125.1, 122.7, 115.4, 81.4, 60.7, 56.2, 39.3, 34.0, 28.6, 14.3.
HRMS (GC-FI+) (m/z): $[M+H]^+$ calcd. for $C_{17}H_{23}NO_4$, 305.1627; found: 305.1640.

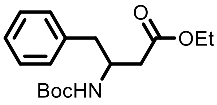

***ethyl 3-((tert-butoxycarbonyl)amino)-4-phenylbutanoate* (6).** Prepared according to GP3 from **1d** (123 mg, 0.3 mmol, 1.0 equiv.) and **2a** (97.3 mg, 0.3 mmol, 1.0 equiv.). Purified via flash column chromatography on silica gel (gradient from Pentane:Ethyl Acetate 98:2 to 90:10) to afford the product as a white solid (60 mg, 65% yield).

Characterization data are in accordance with literature.[8]
^{1}H NMR (400 MHz, $CDCl_3$) δ 7.29 (dd, *J* = 8.1, 6.6 Hz, 2H), 7.24 – 7.15 (m, 3H), 5.05 (s, 1H), 4.22 – 4.09 (m, 3H), 2.91 (d, *J* = 6.6 Hz, 1H), 2.81 (dd, *J* = 13.4, 7.6 Hz, 1H), 2.46 (qd, *J* = 15.8, 5.7 Hz, 2H), 1.40 (s, 9H), 1.26 (t, *J* = 7.2 Hz, 3H).
^{13}C NMR (101 MHz, $CDCl_3$) δ 171.8, 155.2, 137.9, 129.5, 128.6, 126.7, 79.4, 60.7, 49.0, 40.5, 37.9, 28.5, 14.3.

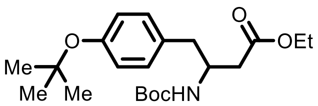

***ethyl 4-(4-(tert-butoxy)phenyl)-3-((tert-butoxycarbonyl)amino)butanoate* (7).** Prepared according to GP3 from **1e** (145 mg, 0.3 mmol, 1.0 equiv.) and **2a** (97.3 mg, 0.3 mmol, 1.0 equiv.). Purified via flash column chromatography on silica gel (gradient from Pentane:Ethyl Acetate 98:2 to 90:10)) to afford the product as a white solid (64 mg, 56% yield).

^{1}H NMR (400 MHz, $CDCl_3$) δ 7.09 – 7.02 (m, 2H), 6.94 – 6.86 (m, 2H), 5.14 – 4.84 (m, 1H), 4.18 – 4.08 (m, 3H), 2.90 – 2.69 (m, 2H), 2.48 (dd, *J* = 15.7, 5.5 Hz, 1H), 2.40 (dd, *J* = 15.7, 5.9 Hz, 1H), 1.39 (s, 9H), 1.31 (s, 9H), 1.25 (t, *J* = 7.1 Hz, 3H).
^{13}C NMR (101 MHz, $CDCl_3$) δ 171.8, 155.2, 154.1, 132.7, 129.9, 124.3, 79.4, 78.4, 60.7, 49.0, 39.8, 38.0, 28.9, 28.5, 14.3.
HRMS (ESI+) (m/z): [M+H]+ calcd. for $C_{21}H_{33}NO_5$, 380.2437; found: 380.2428.

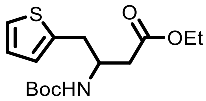

***ethyl 3-((tert-butoxycarbonyl)amino)-4-(thiophen-2-yl)butanoate* (8).** Prepared according to GP3 from **1f** (125 mg, 0.3 mmol, 1.0 equiv.) and **2a** (97.3 mg, 0.3 mmol, 1.0 equiv.). Purified via flash column chromatography on silica gel (gradient from Pentane:Ethyl Acetate 98:2 to 90:10) to afford the product as a colorless oil (32 mg, 34% yield)

^{1}H NMR (400 MHz, $CDCl_3$) δ 7.16 (dd, *J* = 5.1, 1.2 Hz, 1H), 6.93 (dd, *J* = 5.2, 3.4 Hz, 1H), 6.82 (dd, *J* = 3.5, 1.0 Hz, 1H), 5.22 – 4.85 (m, 1H), 4.21 – 4.09 (m, 3H), 3.21 – 2.96 (m, 2H), 2.58 – 2.42 (m, 3H), 1.42 (s, 9H), 1.26 (t, *J* = 7.2 Hz, 3H).
^{13}C NMR (101 MHz, $CDCl_3$) δ 171.6, 155.2, 139.7, 127.1, 126.4, 124.4, 79.6, 60.8, 48.8, 37.7, 34.3, 28.5, 14.3.
HRMS (ESI+) (m/z): [M+H]+ calcd. for $C_{15}H_{23}NO_4S$, 314.1426; found: 314.1429

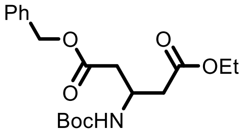

***1-benzyl 5-ethyl 3-((tert-butoxycarbonyl)amino)pentanedioate* (9).** Prepared according to GP3 from **1g** (141 mg, 0.3 mmol, 1.0 equiv.) and **2a** (97 mg, 0.3 mmol, 1.0 equiv.). Purified via flash column chromatography on silica gel (from Pentane:Ethyl Acetate 60:1 to 20:1) to afford the product as a colorless oil (66 mg, 60% yield).

^{1}H NMR (400 MHz, $CDCl_3$) δ 7.39 – 7.32 (m, 5H), 5.39 – 5.25 (m, 1H), 5.12 (s, 2H), 4.40 – 4.29 (m, 1H), 4.12 (q, *J* = 7.1 Hz, 2H), 2.80 – 2.53 (m, 4H), 1.42 (s, 9H), 1.24 (t, *J* = 7.1 Hz, 3H).
^{13}C NMR (101 MHz, $CDCl_3$) δ 171.3, 171.2, 155.1, 135.7, 128.7, 128.5, 128.4, 79.7, 66.7, 60.8, 44.6, 38.3 (2C), 28.5, 14.3.
HRMS (ESI+) (m/z): $[M+H]^+$ calcd. for $C_{19}H_{27}N_2O_6$, 366.1917; found: 366.1929

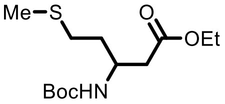

***ethyl 3-((tert-butoxycarbonyl)amino)-5-(methylthio)pentanoate* (10).** Prepared according to GP3 from **1h** (118 mg, 0.3 mmol, 1.0 equiv.) and **2a** (97.3 mg, 0.3 mmol, 1.0 equiv.). Purified via flash column chromatography on silica gel (gradient from Pentane:Ethyl Acetate 98:2 to 90:10) to afford the product as a colorless oil (42 mg, 48% yield).

^{1}H NMR (400 MHz, $CDCl_3$) δ 5.16 – 4.70 (m, 1H), 4.11 (q, *J* = 7.1 Hz, 2H), 4.06 – 3.89 (m, 1H), 2.59 – 2.41 (m, 4H), 2.06 (s, 3H), 1.86 – 1.70 (m, 2H), 1.40 (s, 9H), 1.23 (t, *J* = 7.1 Hz, 3H).
^{13}C NMR (101 MHz, $CDCl_3$) δ 171.6, 155.4, 79.4, 60.7, 47.0, 39.2, 34.2, 30.8, 28.4, 15.6, 14.3.
HRMS (ESI+) (m/z): [M+Na]+ calcd. for $C_{13}H_{25}NO_4S$, 314.1402; found: 314.1400.

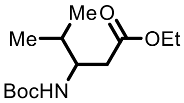

***ethyl 3-((tert-butoxycarbonyl)amino)-4-methylpentanoate* (11).** Prepared according to GP3 from **1i** (109 mg, 0.3 mmol, 1.0 equiv.) and **2a** (97.3 mg, 0.3 mmol, 1.0 equiv.). Purified via flash column chromatography on silica gel (gradient from Pentane:Ethyl Acetate 98:2 to 90:10) to afford the product as a colorless oil (44 mg, 56% yield).

^{1}H NMR (400 MHz, $CDCl_3$) δ 4.87 (d, *J* = 9.7 Hz, 1H), 4.11 (q, *J* = 7.1 Hz, 2H), 3.80 – 3.68 (m, 1H), 2.53 – 2.37 (m, 2H), 1.84 – 1.71 (m, *J* = 7.8, 7.0 Hz, 1H), 1.40 (s, 9H), 1.23 (t, *J* = 7.1 Hz, 3H), 0.89 (d, *J* = 6.8 Hz, 6H).
^{13}C NMR (101 MHz, $CDCl_3$) δ 172.0, 155.6, 79.2, 60.6, 53.1, 37.5, 32.0, 28.5, 19.4, 18.6, 14.3.
HRMS (FI+) (m/z): $[M]^+$ calcd. for $C_{13}H_{25}NO_4$, 259.1784; found: 259.1785

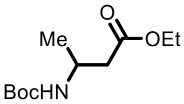

***ethyl 3-((tert-butoxycarbonyl)amino)butanoate* (12).** Prepared according to GP3 from **1j** (100 mg, 0.3 mmol, 1.0 equiv.) and **2a** (97.3 mg, 0.3 mmol, 1.0 equiv.). Purified via flash column chromatography on silica gel (gradient from Pentane:Ethyl Acetate 98:2 to 90:10) to afford the product as a colorless oil (38 mg, 55% yield).

Characterization data are in accordance with literature.[9]
^{1}H NMR (400 MHz, $CDCl_3$) δ 4.94 (s, 1H), 4.10 (q, *J* = 7.1 Hz, 2H), 4.05 (s, 1H), 2.53 – 2.37 (m, 2H), 1.40 (s, 9H), 1.22 (t, *J* = 7.1 Hz, 3H), 1.17 (d, *J* = 6.7 Hz, 3H).
^{13}C NMR (101 MHz, $CDCl_3$) δ 171.6, 155.2, 79.3, 60.5, 43.6, 40.9, 28.5, 20.5, 14.3.

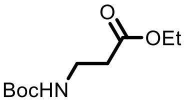

***ethyl 3-((tert-butoxycarbonyl)amino)propanoate* (13).** Prepared according to GP3 from **1k** (96 mg, 0.3 mmol, 1.0 equiv.) and **2a** (97 mg, 0.3 mmol, 1.0 equiv.). Purified via flash column chromatography on silica gel (from Pentane:Ethyl Acetate 10:1 to 5:1) to afford the product as a colorless oil (27 mg, 41% yield).

Characterization data are in accordance with literature.[10]
^{1}H NMR (400 MHz, $CDCl_3$) δ 5.03 (s, 1H), 4.13 (q, *J* = 7.1 Hz, 2H), 3.37 (t, *J* = 6.1 Hz, 2H), 2.49 (t, *J* = 6.1 Hz, 2H), 1.42 (s, 9H), 1.25 (t, *J* = 7.1 Hz, 3H).
^{13}C NMR (101 MHz, $CDCl_3$) δ 172.6, 155.9, 79.4, 60.7, 36.2, 34.8, 28.5, 14.3.

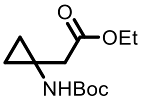

***ethyl 2-(1-((tert-butoxycarbonyl)amino)cyclopropyl)acetatecarboxylate* (14).** Prepared according to GP3 from **1l** (104 mg, 0.3 mmol, 1.0 equiv.) and **2a** (97 mg, 0.3 mmol, 1.0 equiv.). Purified via flash column chromatography on silica gel (Pentane:Ethyl Acetate 10:1) to afford the product as a colorless oil (36 mg, 50% yield).

^{1}H NMR ^{1}H NMR (500 MHz, $CDCl_3$) δ 5.23 (s, 1H), 4.14 (q, *J* = 7.1 Hz, 2H), 2.53 (s, 2H), 1.41 (s, 9H), 1.25 (t, *J* = 7.1 Hz, 3H), 0.89 – 0.80 (m, 2H), 0.74 – 0.63 (m, 2H).
^{13}C NMR (126 MHz, $CDCl_3$) δ 172.2, 155.7, 79.5, 60.5, 41.3, 30.1, 28.5, 14.4, 13.9.
HRMS (GC-FI+) (m/z): $[M+H]^+$ calcd. for $C_{12}H_{21}NO_4$, 243.1471; found: 243.1480.

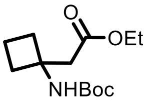

***ethyl 2-(1-((tert-butoxycarbonyl)amino)cyclobutyl)acetate* (15).** Prepared according to GP3 from **1m** (108 mg, 0.3 mmol, 1.0 equiv.) and **2a** (97.3 mg, 0.3 mmol, 1.0 equiv.). Purified via flash column chromatography on silica gel (gradient from Pentane:Ethyl Acetate 98:2 to 90:10) to afford the product as a colorless oil (45 mg, 58% yield).

^{1}H NMR (400 MHz, $CDCl_3$) δ 5.04 (s, 1H), 4.10 (q, *J* = 7.1 Hz, 2H), 2.81 (s, 2H), 2.32 – 2.20 (m, 2H), 2.17 – 2.06 (m, 2H), 1.99 – 1.84 (m, 1H), 1.84 – 1.71 (m, 1H), 1.40 (s, 9H), 1.23 (t, *J* = 7.1 Hz, 3H).
^{13}C NMR (101 MHz, $CDCl_3$) δ 171.6, 154.5, 79.2, 60.3, 54.4, 41.6, 33.0, 28.5, 14.8, 14.3.
HRMS (ESI+) (m/z): [M+Na]+ calcd. for $C_{13}H_{23}NO_4$, 280.1525; found: 280.1529.

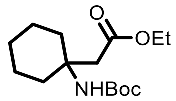

***ethyl 2-(1-((tert-butoxycarbonyl)amino)cyclohexyl)acetate* (16).** Prepared according to GP3 from **1ae** (117 mg, 0.3 mmol, 1.0 equiv.) and **2a** (97 mg, 0.3 mmol, 1.0 equiv.). Purified via flash column chromatography on silica gel (from Pentane:Ethyl Acetate 40:1 to 20:1) to afford the product as a colorless oil (33 mg, 50% yield).

^{1}H NMR (400 MHz, $CDCl_3$) δ 4.49 (s, 1H), 4.09 (q, *J* = 7.1 Hz, 2H), 2.72 (s, 2H), 2.05 (dd, *J* = 11.1, 5.2 Hz, 2H), 1.56 – 1.38 (m, 16H), 1.23 (t, *J* = 7.1 Hz, 4H).
^{13}C NMR (101 MHz, $CDCl_3$) δ 171.4, 154.7, 79.0, 60.2, 53.5, 42.8, 35.2, 28.6, 25.6, 21.6, 14.4.
HRMS (ESI+) (m/z): $[M+H]^+$ calcd. for $C_{15}H_{27}NO_4$, 286.2018; found: 286.2011

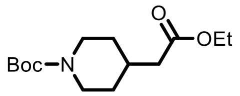

***tert-butyl 4-(2-ethoxy-2-oxoethyl)piperidine-1-carboxylate* (17).** Prepared according to GP3 from **1n** (112 mg, 0.3 mmol, 1.0 equiv.) and **2a** (97.3 mg, 0.3 mmol, 1.0 equiv.). Purified via flash column chromatography on silica gel (gradient from Pentane:Ethyl Acetate 100:0 to 90:10) to afford the product as a pale yellow oil (34 mg, 42% yield).

Characterization data are in accordance with literature.[11]
^{1}H NMR (400 MHz, $CDCl_3$) δ 4.10 (p, *J* = 7.0 Hz, 4H), 2.69 (t, *J* = 12.6 Hz, 2H), 2.20 (d, *J* = 7.1 Hz, 2H), 1.90 (ttt, *J* = 11.1, 7.2, 3.7 Hz, 1H), 1.70 – 1.61 (m, 2H), 1.42 (d, *J* = 0.9 Hz, 9H), 1.23 (t, *J* = 7.1, 3H), 1.13 (qd, *J* = 12.4, 4.3 Hz, 2H).
^{13}C NMR (101 MHz, $CDCl_3$) δ 172.5, 154.9, 79.4, 60.4, 43.8, 41.2, 33.2, 31.9, 28.5, 14.4.

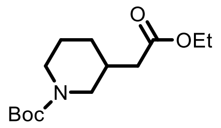

***tert-butyl 3-(2-ethoxy-2-oxoethyl)piperidine-1-carboxylate* (18).** Prepared according to GP3 from **1o** (112 mg, 0.3 mmol, 1.0 equiv.) and **2a** (97.3 mg, 0.3 mmol, 1.0 equiv.). Purified via flash column chromatography on silica gel (gradient from Pentane:Ethyl Acetate 100:0 to 90:10) to afford the product as a pale yellow oil (50 mg, 62% yield).

^{1}H NMR (400 MHz, $CDCl_3$) δ 4.11 (q, *J* = 7.2 Hz, 2H), 4.05 – 3.70 (m, 2H), 2.90 – 2.73 (m, 1H), 2.74 – 2.40 (br m, 1H), 2.23 (dd, *J* = 15.1, 7.0 Hz, 1H), 2.14 (dd, *J* = 15.1, 7.3 Hz, 1H), 2.04 – 1.88 (m, 1H), 1.87 – 1.76 (m, 1H), 1.68 – 1.56 (m, 1H), 1.42 (s, 10H), 1.24 (t, *J* = 7.1 Hz, 3H), 1.21 – 1.09 (m, 1H).
^{13}C NMR (101 MHz, $CDCl_3$) δ 172.3, 154.9, 79.5, 60.5, 49.4, 43.9, 38.4, 32.9, 30.6, 28.5, 24.6, 14.4.
HRMS (FI+) (m/z): $[M]^+$ calcd. for $C_{14}H_{25}NO_4$, 271.1784; found: 271.1781.

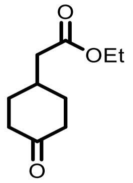

***ethyl 2-(4-oxocyclohexyl)acetate* (19) *(AP_958*).** Prepared according to GP3 from **1p** (86mg, 0.3 mmol, 1.0 equiv.) and **2a** (97 mg, 0.3 mmol, 1.0 equiv.). Purified via flash column chromatography on silica gel (from Pentane:Acetone 12:1) to afford the product as a colorless oil (27 mg, 48% yield).

^{1}H NMR (400 MHz, $CDCl_3$) δ 4.15 (q, *J* = 7.1 Hz, 2H), 2.38 (dd, *J* = 8.8, 4.6 Hz, 4H), 2.33 – 2.22 (m, 3H), 2.12 – 2.05 (m, 2H), 1.54 – 1.43 (m, 2H), 1.29 – 1.21 (m, 3H).
^{13}C NMR (101 MHz, $CDCl_3$) δ 211.4, 172.5, 60.6, 40.7, 40.4, 33.2, 32.5, 14.4.

HRMS (GC-FI+) (m/z): $[M]^+$ calcd. for $C_{10}H_{16}O_3$, 184.1099; found: 184.1099.

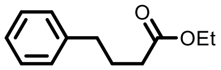

***ethyl 4-phenylbutanoate* (20).** Prepared according to GP3 from **1q** (88.6 mg, 0.3 mmol, 1.0 equiv.) and **2a** (97.3 mg, 0.3 mmol, 1.0 equiv.). Purified via flash column chromatography on silica gel (gradient from Pentane:Ethyl Acetate 100:0 to 95:5) to afford the product as a colorless oil (20 mg, 35% yield).

Characterization data are in accordance with literature.[12]
^{1}H NMR (400 MHz, $CDCl_3$) δ 7.32 – 7.26 (m, 2H), 7.24 – 7.14 (m, 3H), 4.13 (q, *J* = 7.1 Hz, 2H), 2.70 – 2.59 (m, 2H), 2.32 (t, *J* = 7.5 Hz, 2H), 1.96 (p, *J* = 7.6 Hz, 2H), 1.26 (t, *J* = 7.2 Hz, 3H).
^{13}C NMR (101 MHz, $CDCl_3$) δ 173.7, 141.6, 128.6, 128.5, 126.1, 60.4, 35.3, 33.8, 26.7, 14.4.

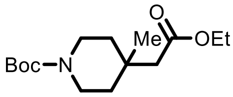

***tert-butyl 4-(2-ethoxy-2-oxoethyl)-4-methylpiperidine-1-carboxylate* (21).** Prepared according to GP3 from **1r** (117 mg, 0.3 mmol, 1.0 equiv.) and 2**a** (97.3 mg, 0.3 mmol, 1.0 equiv.). Purified via flash column chromatography on silica gel (gradient from Pentane:Ethyl Acetate 100:0 to 95:5) to afford the product as a colorless oil (26 mg, 30% yield).

^{1}H NMR (400 MHz, $CDCl_3$) δ 4.11 (q, *J* = 7.1 Hz, 2H), 3.49 (ddd, *J* = 13.7, 6.7, 4.1 Hz, 2H), 3.29 (ddd, *J* = 13.7, 8.5, 3.8 Hz, 2H), 2.25 (s, 2H), δ 1.55 – 1.47 (m, 2H), 1.44 (s, 9H), 1.43 – 1.35 (m, 2H), 1.25 (t, *J* = 7.1 Hz, 3H), 1.07 (s, 3H).
^{13}C NMR (101 MHz, $CDCl_3$) δ 171.8, 155.1, 79.5, 60.2, 45.5, 39.8, 36.9, 31.9, 28.6, 24.2, 14.4.
HRMS (ESI+) (m/z): [M+H]+ calcd. for $C_{15}H_{27}NO_4$, 286.2018; found: 286.2015.

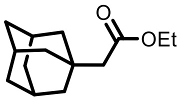

***ethyl (adamantan-1-yl)acetate* (22).** Prepared according to GP3 from **1s** (97.6 mg, 0.3 mmol, 1.0 equiv.) and **2a** (97.3 mg, 0.3 mmol, 1.0 equiv.). Purified via flash column chromatography on silica gel (gradient from Pentane:Ethyl Acetate 100:0 to 95:5) to afford the product as a colorless oil (33 mg, 50% yield).

Characterization data are in accordance with literature.[13]
^{1}H NMR (400 MHz, $CDCl_3$) δ 4.10 (q, *J* = 7.1 Hz, 2H), 2.05 (s, 2H), 1.99 – 1.92 (m, 3H), 1.74 – 1.57 (m, 12H), 1.25 (t, *J* = 7.1 Hz, 3H).
^{13}C NMR (101 MHz, $CDCl_3$) δ 172.0, 59.9, 49.1, 42.5, 36.9, 32.9, 28.8, 14.5.

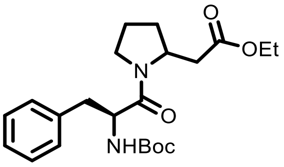

***ethyl 3-((tert-butoxycarbonyl)amino)propanoate* (23).** Prepared according to GP1 from **1t** (152 mg, 0.3 mmol, 1.0 equiv.) and **2a** (97 mg, 0.3 mmol, 1.0 equiv.). Purified via flash column chromatography on silica gel (Pentane:Ethyl Acetate 4:1) to afford the product as a white solid (57 mg, 47% yield). *d.r.* ratio of **23** was determined via ^{1}H NMR to be > 20:1.

^{1}H NMR (400 MHz, $CDCl_3$) δ 7.22 – 7.12 (m, 5H), 5.30 (d, *J* = 9.0 Hz, 1H), 4.52 – 4.41 (m, 1H), 4.20 (ddt, *J* = 10.2, 6.9, 3.2 Hz, 1H), 4.09 – 3.98 (m, 2H), 3.34 – 3.23 (m, 1H), 2.99 – 2.82 (m, 3H), 2.46 (dt, *J* = 9.8, 7.1 Hz, 1H), 2.16 (dd, *J* = 15.5, 9.9 Hz, 1H), 1.76 – 1.49 (m, 3H), 1.35 (s, 9H), 1.26 – 1.09 (m, 4H).
^{13}C NMR (101 MHz, $CDCl_3$) δ 171.4, 170.2, 155.2, 136.6, 129.5, 128.5, 127.0, 79.7, 60.5, 54.3, 53.9, 46.6, 40.2, 37.4, 29.8, 28.4, 23.4, 14.3.
HRMS (ESI+) (m/z): $[M+H]^+$ calcd. for $C_{22}H_{32}N_2O_5$, 405.2389; found: 405.2380.

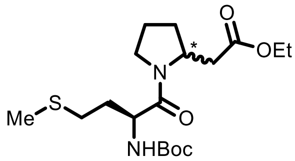

ethyl 2-(1-((tert-butoxycarbonyl)-L-methionyl)pyrrolidin-2-yl)acetate **(24).** Prepared according to GP3 from **1u** (148 mg, 0.3 mmol, 1.0 equiv.) and **2a** (97.3 mg, 0.3 mmol, 1.0 equiv.). Purified via flash column chromatography on silica gel (gradient from Pentane:Ethyl Acetate 80:20 to 60:40) to afford the two diastereomers as an inseparable mixture as a colorless oil (49 mg, 42% yield). *d.r.* ratio of **24** was determined via ^{1}H NMR to be 3:1.

^{1}H NMR (400 MHz, $CDCl_3$) δ 5.36 & 5.32 (diastereomeric d, *J* = 8.8, 1H), 4.56 – 4.46 (m, 1H), 4.45 – 4.32 (m, 1H), 4.20 – 4.03 (m, 2H), 3.73 – 3.63 (m, 1H), 3.58 – 3.41 (m, 1H), 2.99 & 2.81 (diastereomeric dd, *J* = 15.4, 3.7 Hz, 1H), 2.59 – 2.48 (m, 2H), 2.41 & 2.29 (diastereomeric dd, *J* = 15.5, 9.8 Hz, 1H), 2.09 & 2.08 (diastereomeric s, 3H), 2.08 – 1.86 (m, 4H), 1.86 – 1.74 (m, 2H), 1.42 & 1.41 (diastereomeric s, 9H), 1.23 (diastereomeric t, *J* = 7.1 Hz, 3H).
^{13}C NMR (101 MHz, $CDCl_3$) δ 171.4 & 171.3 (diastereomeric signals), 170.6 & 170.3 (diastereomeric signals), 155.6, 79.8, 60.6 & 60.6 (diastereomeric signals), 54.6 & 54.3 (diastereomeric signals), 51.4 & 51.3 (diastereomeric signals), 47.2 & 46.8 (diastereomeric signals), 37.6 & 37.5 (diastereomeric signals), 33.1 & 32.7 (diastereomeric signals), 30.3 & 30.2 (diastereomeric signals), 30.0 & 29.9 (diastereomeric signals), 28.4, 24.3 & 23.8 (diastereomeric signals), 15.8 &15.8 (diastereomeric signals), 14.3.
HRMS (ESI+) (m/z): $[M+H]^+$ calcd. for $C_{18}H_{32}N_2O_5S$, 389.2110; found: 389.2111.

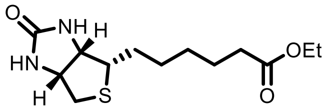

ethyl 6-((3aS,4S,6aR)-2-oxohexahydro-1H-thieno[3,4-d]imidazol-4-yl)hexanoate **(25).** Prepared according to GP3 from **1v** (117 mg, 0.3 mmol, 1.0 equiv.) and **2a** (97.3 mg, 0.3 mmol, 1.0 equiv.). Purified via flash column chromatography on silica gel (gradient from Dichloromethane:Methanol 98:2 to 95:5) to afford the product as a pale yellow oil (29 mg, 34% yield).

^{1}H NMR (400 MHz, $CDCl_3$) δ 5.94 (s, 1H), 5.63 (s, 1H), 4.49 (dd, *J* = 7.8, 5.0 Hz, 1H), 4.33 – 4.25 (m, 1H), 4.11 (q, *J* = 7.1 Hz, 2H), 3.20 – 3.08 (m, 1H), 2.95 – 2.85 (m, 1H), 2.72 (d, *J* = 12.8 Hz, 1H), 2.28 (t, *J* = 7.5 Hz, 2H), 1.77 – 1.54 (m, 4H), 1.48 – 1.30 (m, 4H), 1.24 (t, *J* = 7.1 Hz, 3H).
^{13}C NMR (101 MHz, $CDCl_3$) δ 174.0, 163.9, 62.2, 60.5, 60.3, 55.9, 40.7, 34.4, 29.2, 28.8, 28.5, 24.7, 14.4.
HRMS (ESI+) (m/z): $[M+H]^+$ calcd. for $C_{13}H_{22}N_2O_3S$, 287.1429; found: 287.1423.

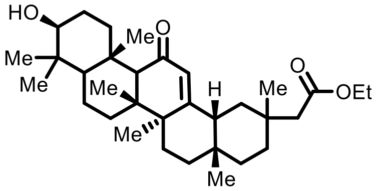

ethyl (enoxolone)acetate **(26).** Prepared according to GP3 from **1z** (123 mg, 0.2 mmol, 1.0 equiv.) and **2a** (65 mg, 0.3 mmol, 1.0 equiv.). Purified via flash column chromatography on silica gel (gradient from Pentane:Ethyl Acetate 90:10 to 80:20) to afford the product with a minor aromatic impurity. Further purification via reverse-phase column chromatrography (gradient from Acetonitrile:Water 50:50 to 80:20) followed by extraction with ethyl acetate yielded the pure product as an inseparable mixture as a white solid (31 mg, 30% yield). *d.r.* ratio of **26** was determined via ^{1}H NMR to be 2.5:1.

^{1}H NMR (400 MHz, $CDCl_3$) δ 5.58 (s, 1H), 4.10 (q, *J* = 7.2 Hz, 3H), 3.22 (dd, *J* = 10.8, 5.5 Hz, 1H), 2.77 (dt, *J* = 13.5, 3.6 Hz, 1H), 2.32 (s, 1H), 2.20 – 1.99 (m, 5H), 1.94 – 1.74 (m, 2H), 1.72 – 1.53 (m, 3H), 1.53 – 1.37 (m, 4H), 1.35 (s, 3H), 1.34 – 1.27 (m, 3H), 1.27 – 1.21 (m, 4H), 1.21 – 1.14 (m, 2H), 1.14 – 1.10 (m, 5H), 1.02 – 0.98 (m, 6H), 0.97 – 0.92 (m, 1H), 0.90 – 0.83 (m, 3H), 0.79 (s, 3H), 0.72 – 0.64 (m, 1H).
^{13}C NMR (101 MHz, $CDCl_3$) δ 200.4, 171.9, 170.0, 128.5, 78.9, 61.9, 60.2, 55.1, 49.7, 47.2, 45.6, 43.5, 42.7, 39.3, 37.2, 36.0, 34.1, 32.9, 32.5, 32.2, 29.8, 28.7, 28.2, 27.4, 26.5, 26.5, 23.5, 21.7, 18.8, 17.6, 16.5, 15.7, 14.5.

HRMS (FI+) (m/z): $[M]^+$ calcd. for $C_{33}H_{52}O_4$, 512.3866; found: 512.3857.

10.4 Characterization of compounds 27-58 (alkylation)

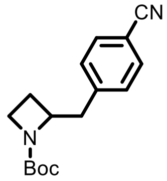

***tert*-butyl 2-(4-cyanobenzyl)azetidine-1-carboxylate (27)**. Prepared according to GP3 from **1aa** (104 mg, 0.3 mmol, 1.0 equiv.) and **2b** (106 mg, 0.3 mmol, 1.0 equiv.). Purified via flash column chromatography on silica gel (gradient from Pentane:Ethyl Acetate 98:2 to 90:10) to afford the product as a white solid (33 mg, 40% yield).

^{1}H NMR (400 MHz, $CDCl_3$) δ 7.59 (d, *J* = 8.11, 2H), 7.30 (d, *J* = 8.11, 2H), 4.48 – 4.36 (m, 1H), 3.79 (td, *J* = 8.8, 6.7 Hz, 1H), 3.61 (td, *J* = 8.8, 5.2 Hz, 1H), 3.18 (dd, *J* = 13.6, 4.2 Hz, 1H), 3.02 (dd, *J* = 13.6, 8.0 Hz, 1H), 2.23 – 2.10 (m, 1H), 1.90 – 1.77 (m, 1H), 1.44 (s, 9H).
^{13}C NMR (101 MHz, $CDCl_3$) δ 156.5, 143.3, 132.3, 130.4, 119.1, 110.5, 79.7, 61.7, 46.4, 41.1, 28.6, 21.1.
HRMS (ESI+) (m/z): $[M+H]^+$ calcd. for $C_{16}H_{20}N_2O_2$, 273.1603; found: 273.1616.

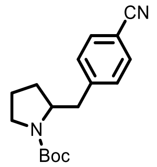

***tert*-butyl 2-(4-cyanobenzyl)pyrrolidine-1-carboxylate (28).** Prepared according to GP3 from **1a** (108 mg, 0.3 mmol, 1.0 equiv.) and **2b** (106 mg, 0.3 mmol, 1.0 equiv.). Purified via flash column chromatography on silica gel (Pentane:Ethyl Acetate 10:1) to afford the product (mixture of rotamers) as a colorless oil (56 mg, 65% yield).

Characterization data are in accordance with literature.[14]
^{1}H NMR (400 MHz, $CDCl_3$) δ 7.61 – 7.53 (m, 2H), 7.36 – 7.25 (m, 2H), 4.12 – 3.87 (m, 1H), 3.47 – 3.00 (m, 3H), 2.74 – 2.56 (m, 1H), 1.91 – 1.68 (m, 3H), 1.66 – 1.58 (m, 1H), 1.48 (s, 9H).
^{13}C NMR (101 MHz, $CDCl_3$) δ 154.7 & 154.5 (rotameric signals), 145.0 & 144.6 (rotameric signals), 132.3 & 132.2 (rotameric signals), 130.4 & 130.3 (rotameric signals), 119.1, 110.2, 79.7 & 79.5 (rotameric signals), 58.51, 46.92 & 46.43 (rotameric signals), 41.0 & 40.1 (rotameric signals), 30.0 & 29.2 (rotameric signals), 28.3, 23.6 & 22.8 (rotameric signals).

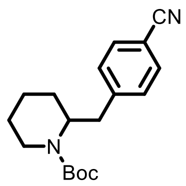

***tert*-butyl 2-(4-cyanobenzyl)piperidine-1-carboxylate (29).** Prepared according to GP3 from **1b** (112 mg, 0.3 mmol, 1.0 equiv.) and **2b** (106 mg, 0.3 mmol, 1.0 equiv.). Purified via flash column chromatography on silica gel (gradient from Pentane:Ethyl Acetate 96:4 to 90:10) to afford the product as a white solid (48 mg, 53% yield).

^{1}H NMR (400 MHz, $CDCl_3$) δ 7.55 (d, *J* = 8.0 Hz, 2H), 7.28 (d, *J* = 7.7 Hz, 2H), 4.44 (s, 1H), 4.04 (s, 1H), 2.99 (dd, *J* = 13.3, 8.2 Hz, 1H), 2.87 (td, *J* = 13.2, 2.6 Hz, 1H), 2.77 (dd, *J* = 13.4, 7.2 Hz, 1H), 1.73 – 1.36 (m, 6H), 1.29 (s, 9H).
^{13}C NMR (101 MHz, $CDCl_3$) δ 154.8, 145.3, 132.2, 130.2, 119.1, 110.1, 79.5, 52.0, 39.1, 36.5, 28.4, 27.9, 25.6, 19.1.
HRMS (ESI+) (m/z): $[M+H]^+$ calcd. for $C_{18}H_{24}N_2O_2$, 301.1916; found: 301.1928.

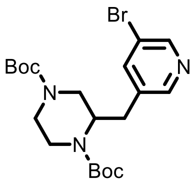

di-tert-butyl 2-((5-bromopyridin-3-yl)methyl)piperazine-1,4-dicarboxylate **(30).** Prepared according to GP3 from **1ab** (95.1 mg, 0.2 mmol, 1.0 equiv.) and **2o** (81.6 mg, 0.2 mmol, 1.0 equiv.). Purified via flash column chromatography on silica gel (gradient from Pentane:Ethyl Acetate 90:10 to 70:30) to afford the product (mixture of rotamers) as a white solid (55 mg, 40% yield).

^{1}H NMR (400 MHz, $CDCl_3$) δ 8.54 (s, 1H), 8.39 (s, 1H), 7.85 – 7.58 (m, 1H), 4.53 – 3.68 (m, 4H), 3.14 – 2.99 (m, 1H), 2.91 (dd, J = 13.6, 3.9 Hz, 1H), 2.86 – 2.68 (m, 3H), 1.48 (s, 9H), 1.40 (s, 9H).m
^{13}C NMR (101 MHz, $CDCl_3$) δ 155.1, 154.4, 149.2, 148.9, 139.5, 135.7, 120.8, 80.6 (2C), 53.1 & 51.5 (rotameric signals), 45.0 & 42.9 (rotameric signals), 39.8 & 38.5 (rotameric signals), 32.6, 29.8, 28.5, 28.4.
HRMS (ESI+) (m/z): $[M+H]^+$ calcd. for $C_{20}H_{30}BrN_3O_4$, 456.1498; found: 456.1493.

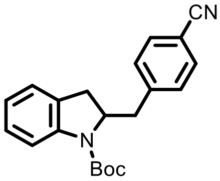

tert-butyl 2-(4-cyanobenzyl)indoline-1-carboxylate **(31).** Prepared according to GP3 from **1c** (123 mg, 0.3 mmol, 1.0 equiv.) and **2b** (106 mg, 0.3 mmol, 1.0 equiv.). Purified via flash column chromatography on silica gel (gradient from Pentane:Ethyl Acetate 98:2 to 85:15) to afford the product as a pale yellow oil (50 mg, 50% yield).

^{1}H NMR (400 MHz, $CDCl_3$) δ 7.94 – 7.40 (br s, 1H), 7.58 (d, J = 7.9 Hz, 2H), 7.32 (d, J = 7.8 Hz, 2H), 7.17 (t, J = 7.7 Hz, 1H), 7.11 (d, J = 7.4 Hz, 1H), 6.94 (t, J = 7.4 Hz, 1H), 4.65 (s, 1H), 3.23 (d, J = 13.2 Hz, 1H), 3.15 (dd, J = 16.2, 9.4 Hz, 1H), 2.77 – 2.63 (m, 2H), 1.57 (s, 9H).
^{13}C NMR (101 MHz, $CDCl_3$) δ 152.2, 143.6, 141.7, 132.3, 130.4, 129.6, 127.7, 125.1, 122.8, 119.0, 115.5, 110.5, 81.4, 60.1, 40.5, 32.7, 28.5.
HRMS (ESI+) (m/z): $[M+H]^+$ calcd. for $C_{21}H_{22}N_2O_2$, 335.1760; found: 335.1750.

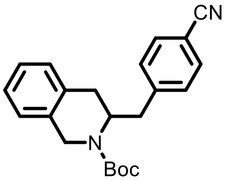

tert-butyl 3-(4-cyanobenzyl)-3,4-dihydroisoquinoline-2(1H)-carboxylate **(32).** Prepared according to GP3 from **1ac** (127 mg, 0.3 mmol, 1.0 equiv.) and **2b** (106 mg, 0.3 mmol, 1.0 equiv.). Purified via flash column chromatography on silica gel (gradient from Pentane:Ethyl Acetate 100:0 to 80:20) to afford the product (mixture of rotamers) as a white solid (56 mg, 54% yield).

^{1}H NMR (400 MHz, $CDCl_3$) δ 7.60 – 7.53 (m, 2H), 7.30 – 7.07 (m, 6H), 4.86 – 4.49 (m, 2H), 4.34 (br t, J = 13.7 Hz, 1H), 3.00 (dd, J = 15.9, 5.5 Hz, 1H), 2.83 (dd, J = 13.4, 7.3 Hz, 1H), 2.64 – 2.52 (m, 2H), 1.47 – 1.37 (m, 9H).
^{13}C NMR (101 MHz, $CDCl_3$) δ 154.7 & 154.6 (rotameric signals), 144.6, 132.8, 132.6, 132.3, 130.1, 129.3 & 129.0 (rotameric signals), 127.0, 126.7, 126.5 & 126.3 (rotameric signals), 119.0, 110.3, 80.1, 51.7 & 50.1 (rotameric signals), 43.7 & 43.1 (rotameric signals), 39.1 & 38.5 (rotameric signals), 32.8 & 32.1 (rotameric signals), 28.4.
HRMS (FD+) (m/z): $[M]^+$ calcd. for $C_{22}H_{24}N_2O_2$, 348.1838; found: 348.1835.

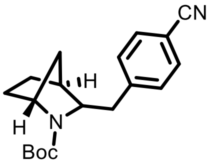

***tert*-butyl (1R,4S)-3-(4-cyanobenzyl)-2-azabicyclo[2.2.1]heptane-2-carboxylate** (**33**). Prepared according to GP3 from **1ad** (116 mg, 0.3 mmol, 1.0 equiv.) and **2b** (147 mg, 0.3 mmol, 1.0 equiv.). Purified via flash column chromatography on silica gel (from Pentane:Ethyl Acetate 20:1 to 10:1) to afford the two diastereomers as an inseparable mixture as a colorless oil (63 mg, 67% yield). *d.r.* ratio of **33** was determined via ^{1}H NMR to be 2.5:1.

^{1}H NMR (400 MHz, $CDCl_3$) δ 7.61 – 7.52 (m, 2H), 7.34 & 7.27 (diastereomeric d, *J* = 7.9 Hz, 2H), 4.21 – 4.18 & 4.11 – 4.06 (diastereomeric m, 1H), 3.39 & 3.15 (diastereomeric dd, *J* = 13.2, 3.3 Hz, 1H), 3.30 – 3.22 (m, 1H), 2.50 – 2.40 (m, 1H), 2.21 – 2.14 (m, 1H), 1.83 – 1.52 (m, 4H), 1.49 & 1.47 (diastereomeric s, 9H), 1.28 – 1.19 (m, 2H).
^{13}C NMR (101 MHz, $CDCl_3$) δ 155.2 & 154.6 (diastereomeric signals), 145.2 & 145.1 (diastereomeric signals), 132.4 & 132.3 (diastereomeric signals), 130.3 & 130.1 (diastereomeric signals), 119.2 & 119.0 (diastereomeric signals), 110.3 & 110.1 (diastereomeric signals), 79.7 & 79.4 (diastereomeric signals), 65.7 & 65.6 (diastereomeric signals), 58.0 & 57.2 (diastereomeric signals), 40.7 & 40.1 (diastereomeric signals), 40.0 & 39.4 (diastereomeric signals), 34.7 & 33.9 (diastereomeric signals), 30.4 & 29.9 (diastereomeric signals), 28.7 & 28.7 (diastereomeric signals), 27.8 & 27.7(diastereomeric signals).
HRMS (ESI+) (m/z): $[M+H]^+$ calcd. for $C_{19}H_{24}N_2O_2$, 313.1916; found: 313.1912.

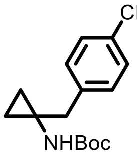

***tert*-butyl (1-(4-cyanobenzyl)cyclopropyl)carbamate** (**34**). Prepared according to GP3 from **1l** (104 mg, 0.3 mmol, 1.0 equiv.) and **2b** (106 mg, 0.3 mmol, 1.0 equiv.). Purified via flash column chromatography on silica gel (from Pentane:Ethyl Acetate 40:1 to 15:1) to afford the product as a colorless oil (33 mg, 41% yield).

^{1}H NMR (500 MHz, $CDCl_3$) δ 7.59 (d, *J* = 8.2 Hz, 2H), 7.29 (d, *J* = 8.0 Hz, 2H), 4.75 – 4.48 (m, 1H), 2.95 – 2.79 (m, 2H), 1.47 – 1.36 (m, 9H), 0.83 – 0.74 (m, 4H).
^{13}C NMR (126 MHz, $CDCl_3$) δ 155.5, 145.1, 132.2, 130.3, 119.1, 110.5, 79.7, 41.9, 34.1, 28.5, 13.8.
HRMS (GC-FI+) (m/z): $[M+H]^+$ calcd. for $C_{16}H_{20}N_2O_2$, 272.1525; found: 272.1522.

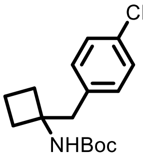

***tert*-butyl (1-(4-cyanobenzyl)cyclobutyl)carbamate** (**35**). Prepared according to GP3 from **1m** (108 mg, 0.3 mmol, 1.0 equiv.) and **2b** (106 mg, 0.3 mmol, 1.0 equiv.). Purified via flash column chromatography on silica gel (gradient from Pentane:Ethyl Acetate 100:0 to 70:30) to afford the product as a white solid (27 mg, 31% yield).

^{1}H NMR (400 MHz, $CDCl_3$) δ 7.57 (d, *J* = 8.20 Hz, 2H), 7.25 (d, *J* = 8.20 Hz, 2H), 4.46 (s, 1H), 3.29 – 3.03 (m, 2H), 2.22 – 2.09 (m, 2H), 2.09 – 1.92 (m, 3H), 1.92 – 1.77 (m, 1H), 1.46 (s, 9H).
^{13}C NMR (101 MHz, $CDCl_3$) δ 154.3, 144.1, 131.9, 130.9, 119.1, 110.3, 79.4, 56.9, 42.6, 33.1, 28.6, 15.1.
HRMS (FI+) (m/z): $[M]^+$ calcd. for $C_{17}H_{22}N_2O_2$, 286.1681; found: 286.1676.

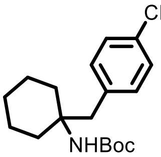

***tert*-butyl (1-(4-cyanobenzyl)cyclohexyl)carbamate** (**36**). Prepared according to GP3 from **1ae** (117 mg, 0.3 mmol, 1.0 equiv.) and **2b** (106 mg, 0.3 mmol, 1.0 equiv.). Purified via flash column chromatography on silica gel (gradient from Pentane:Ethyl Acetate 98:2 to 90:10) to afford the product as a white solid (34 mg, 36% yield).

^{1}H NMR (400 MHz, $CDCl_3$) δ 7.57 – 7.50 (d, *J* = 8.1 Hz, 2H), 7.23 (d, *J* = 8.1 Hz, 2H), 4.05 (s, 1H), 3.05 (s, 2H), 1.89 (d, *J* = 12.1 Hz, 2H), 1.64 – 1.50 (m, 3H), 1.46 (s, 9H), 1.43 – 1.17 (m, 5H).
^{13}C NMR (101 MHz, $CDCl_3$) δ 154.5, 143.8, 131.6, 131.4, 119.2, 110.1, 79.1, 54.9, 44.3, 35.2, 28.6, 25.7, 21.5.

HRMS (FD+) (m/z): $[M]^+$ calcd. for $C_{19}H_{26}N_2O_2$, 315.2073; found: 315.2065.

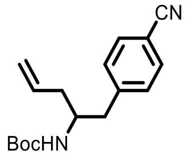

***tert*-butyl (1-(4-cyanophenyl)pent-4-en-2-yl)carbamate** **(37).** Prepared according to GP3 from **1af** (108 mg, 0.3 mmol, 1.0 equiv.) and **2b** (106 mg, 0.3 mmol, 1.0 equiv.). Purified via flash column chromatography on silica gel (from Pentane:Ethyl Acetate 30:1 to 10:1) to afford the product as a colorless oil (36 mg, 42% yield).

^{1}H NMR (500 MHz, $CDCl_3$) δ 7.57 (d, *J* = 7.8 Hz, 2H), 7.30 (d, *J* = 7.8 Hz, 2H), 5.83 – 5.71 (m, 1H), 5.16 – 5.07 (m, 2H), 4.46 – 4.32 (m, 1H), 4.01 – 3.82 (m, 1H), 2.86 – 2.81 (m, 2H), 2.24 (dt, *J* = 13.3, 6.4 Hz, 1H), 2.13 (dt, *J* = 14.4, 7.3 Hz, 1H), 1.37 (s, 9H).
^{13}C NMR (126 MHz, $CDCl_3$) δ 155.3, 144.2, 133.9, 132.2, 130.3, 119.1, 118.7, 110.4, 79.6, 51.0, 41.0, 38.5, 28.4.
HRMS (GC-FI+) (m/z): $[M+H]^+$ calcd. for $C_{17}H_{22}N_2O_2$, 286.1681; found: 286.1686.

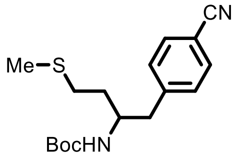

***tert*-butyl (1-(4-cyanophenyl)-4-(methylthio)butan-2-yl)carbamate** **(38).** Prepared according to GP3 from **1h** (118 mg, 0.3 mmol, 1.0 equiv.) and **2b** (106 mg, 0.3 mmol, 1.0 equiv.). Purified via flash column chromatography on silica gel (gradient from Pentane:Ethyl Acetate 100:0 to 70:30) to afford the product as a white solid (26 mg, 27% yield).

^{1}H NMR (400 MHz, $CDCl_3$) δ 7.58 (d, *J* = 8.0 Hz, 2H), 7.30 (d, *J* = 8.0 Hz, 2H), 4.44 – 4.29 (m, 1H), 4.0 – 3.84 (br s, 1H), 2.85 (d, *J* = 6.8 Hz, 2H), 2.63 – 2.44 (m, 2H), 2.06 (s, 3H), 1.84 – 1.71 (m, 1H), 1.71 – 1.57 (m, 1H), 1.38 (s, 9H).
^{13}C NMR (101 MHz, $CDCl_3$) δ 155.4, 144.0, 132.3, 130.3, 119.0, 110.5, 79.7, 51.1, 41.9, 34.2, 30.9, 28.4, 15.8.
HRMS (FD+) (m/z): $[M]^+$ calcd. for $C_{17}H_{24}N_2O_2S$, 320.1558; found: 320.1547.

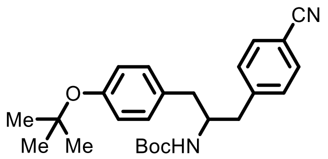

***tert*-butyl (1-(4-(*tert*-butoxy)phenyl)-3-(4-cyanophenyl)propan-2-yl)carbamate** **(39).** Prepared according to GP3 from **1e** (145 mg, 0.3 mmol, 1.0 equiv.) and **2b** (106 mg, 0.3 mmol, 1.0 equiv.). Purified via flash column chromatography on silica gel (Pentane:Ethyl Acetate 10:1) to afford the product as a colorless oil (75 mg, 61% yield).

^{1}H NMR (400 MHz, $CDCl_3$) δ 7.56 (d, *J* = 8.1 Hz, 2H), 7.27 (d, *J* = 8.3 Hz, 2H), 7.05 (d, *J* = 8.4 Hz, 2H), 6.92 (d, *J* = 8.4 Hz, 2H), 4.35 (s, 1H), 4.20 – 4.01 (m, 1H), 2.87 (dd, *J* = 13.8, 5.7 Hz, 1H), 2.73 (d, *J* = 6.4 Hz, 3H), 1.32 (s, 18H).
^{13}C NMR (101 MHz, $CDCl_3$) δ 155.2, 154.2, 144.4, 132.5, 132.2, 130.2, 129.8, 124.4, 119.1, 110.4, 79.5, 78.5, 52.6, 40.9, 40.2, 28.9, 28.4.
HRMS (ESI+) (m/z): $[M+H]^+$ calcd. for $C_{25}H_{32}N_2O_3$, 409.2491; found: 409.2490.

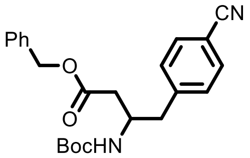

benzyl 3-((*tert*-butoxycarbonyl)amino)-4-(4-cyanophenyl)butanoate **(40).** Prepared according to GP3 from **1g** (140 mg, 0.3 mmol, 1.0 equiv.) and **2b** (106 mg, 0.3 mmol, 1.0 equiv.). Purified via flash column chromatography

on silica gel (gradient from Cyclohexane:Ethyl Acetate 100:0 to 80:20) to afford the product as a white solid (67 mg, 57% yield).

^{1}H NMR (400 MHz, $CDCl_3$) δ 7.54 (d, *J* = 8.1 Hz, 2H), 7.43 – 7.30 (m, 5H), 7.24 (d, *J* = 8.1 Hz, 2H), 5.24 – 5.00 (m, 3H), 4.24 – 4.06 (m, 1H), 2.95 (dd, *J* = 13.6, 7.4 Hz, 1H), 2.85 (dd, *J* = 13.4, 7.0 Hz, 1H), 2.58 (dd, *J* = 16.1, 5.5 Hz, 1H), 2.49 (dd, *J* = 16.1, 5.5 Hz, 1H), 1.37 (s, 9H).
^{13}C NMR (101 MHz, $CDCl_3$) δ 171.3, 155.1, 143.7, 135.6, 132.4, 130.2, 128.8, 128.7, 128.5, 119.0, 110.7, 79.8, 66.8, 48.6, 40.6, 37.9, 28.4.
HRMS (ESI+) (m/z): $[M+H]^+$ calcd. for $C_{23}H_{26}N_2O_4$, 395.1971; found: 395.1995.

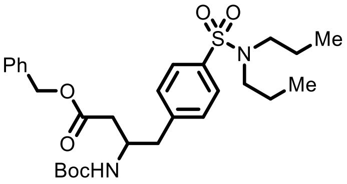

benzyl 3-((tert-butoxycarbonyl)amino)-4-(4-(N,N-dipropylsulfamoyl)phenyl)butanoate **(41).** Prepared according to GP3 from **1g** (141 mg, 0.3 mmol, 1.0 equiv.) and **2h** (147 mg, 0.3 mmol, 1.0 equiv.). Purified via flash column chromatography on silica gel (from Hexane:Ethyl Acetate 5:1 to 2:1) to afford the product as a colorless oil (72 mg, 45% yield).

^{1}H NMR (400 MHz, $CDCl_3$) δ 7.69 (d, *J* = 8.1 Hz, 2H), 7.40 – 7.34 (m, 5H), 7.27 – 7.21 (m, 2H), 5.22 – 4.99 (m, 3H), 4.22 – 3.95 (m, 1H), 3.10 – 3.01 (m, 4H), 3.01 – 2.82 (m, 2H), 2.57 (dd, *J* = 16.1, 5.4 Hz, 1H), 2.48 (dd, *J* = 16.1, 5.6 Hz, 1H), 1.55 (dt, *J* = 15.1, 7.8 Hz, 4H), 1.39 (s, 9H), 0.86 (t, *J* = 7.4 Hz, 6H).
^{13}C NMR (101 MHz, $CDCl_3$) δ 171.4, 155.1, 142.8, 138.6, 135.7, 130.0, 128.8, 128.6, 128.5, 127.4, 79.7, 66.7, 50.2, 40.2, 37.8, 29.8, 28.5, 22.2, 11.3.
HRMS (ESI+) (m/z): $[M+H]^+$ calcd. for $C_{28}H_{40}N_2O_6S$, 533.2685; found: 533.2671

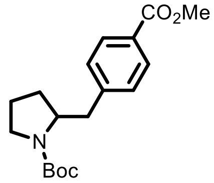

tert-butyl 2-(4-(methoxycarbonyl)benzyl)pyrrolidine-1-carboxylate **(42).** Prepared according to GP3 from **1a** (108 mg, 0.3 mmol, 1.0 equiv.) and **2g** (116 mg, 0.3 mmol, 1.0 equiv.). Purified via flash column chromatography on silica gel (gradient from Pentane:Ethyl Acetate 98:2 to 90:10) to afford the product (mixture of rotamers) as a colorless oil (45 mg, 47% yield).

Characterization data are in accordance with literature.[15]
^{1}H NMR (400 MHz, $CDCl_3$) δ 7.95 (d, *J* = 7.7 Hz, 2H), 7.25 (q, *J* = 7.5 Hz, 2H), 4.11 – 3.92 (m, 1H), 3.89 (s, 3H), 3.43 – 3.22 (m, 2H), 3.22 – 3.03 (m, 1H), 2.69 – 2.55 (m, 1H), 1.85 – 1.59 (m, 4H), 1.49 (s, 9H).
^{13}C NMR (101 MHz, $CDCl_3$) δ 167.2, 154.6, 144.8, 129.8 & 129.7 (rotameric signals), 129.7 & 129.5 (rotameric signals), 128.4 & 128.2 (rotameric signals), 79.6 & 79.3 (rotameric signals), 58.7 & 58.5 (rotameric signals), 52.1, 46.9 & 46.4 (rotameric signals), 40.8 & 39.8 (rotameric signals), 29.8, 28.7, 23.6 & 22.8 (rotameric signals).

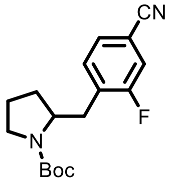

tert-butyl 2-(4-cyano-2-fluorobenzyl)pyrrolidine-1-carboxylate **(43).** Prepared according to GP3 from **1a** (108 mg, 0.3 mmol, 1.0 equiv.) and **2f** (111 mg, 0.3 mmol, 1.0 equiv.). Purified via flash column chromatography on silica gel (gradient from Pentane:Ethyl Acetate 98:2 to 90:10) to afford the product (mixture of rotamers) as a white solid (50 mg, 55% yield).

^{1}H NMR (300 MHz, $CDCl_3$) δ 7.56 – 7.30 (m, 2H), 7.18 – 7.06 (m, 1H), 4.07 – 3.80 (m, 1H), 3.53 – 3.16 (m, 2H), 3.04 (t, *J* = 15.8 Hz, 1H), 2.74 – 2.45 (m, 1H), 1.93 – 1.53 (m, 4H), 1.47 (s, 9H).
^{13}C NMR (75 MHz, $CDCl_3$) δ 162.0 (d, *J* = 257.7 Hz), 154.7, 136.4 (d, *J* = 3.8 Hz), 136.2, 134.0, 116.5, 114.1, 101.4, 79.8 &79.6 (rotameric signals), 58.5 & 58.4 (rotameric signals), 46.9 & 46.5 (rotameric signals), 39.6 & 38.7 (rotameric signals), 29.9 & 29.3 (rotameric signals), 28.7, 23.6 & 22.8 (rotameric signals).
^{19}F NMR (282 MHz, $CDCl_3$) δ -110.1 & -110.5 (rotameric signals).
HRMS (ESI+) (m/z): $[M+H]^+$ calcd. for $C_{17}H_{21}FN_2O_2$, 305.1665; found: 305.1661.

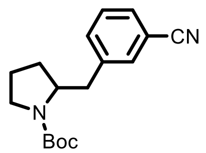

***tert*-butyl 2-(3-cyanobenzyl)pyrrolidine-1-carboxylate (44).** Prepared according to GP3 from **1a** (108 mg, 0.3 mmol, 1.0 equiv.) and **2e** (106 mg, 0.3 mmol, 1.0 equiv.). Purified via flash column chromatography on silica gel (gradient from Pentane:Ethyl Acetate 98:2 to 90:10) to afford the product (mixture of rotamers) as a pale yellow oil (43 mg, 50% yield).

Characterization data are in accordance with literature.[16]
^{1}H NMR (400 MHz, $CDCl_3$) δ 7.58 – 7.30 (m, 4H), 4.05 – 3.83 (m, 1H), 3.47 – 3.18 (m, 2H), 3.17 – 2.95 (m, 1H), 2.72 – 2.50 (m, 1H), 1.86 – 1.53 (m, 4H), 1.47 (s, 9H).
^{13}C NMR (101 MHz, $CDCl_3$) δ 154.7 & 154.5 (rotameric signals), 140.8, 134.2 & 134.0 (rotameric signals), 133.1 & 132.9 (rotameric signals), 130.1, 129.4 & 129.2 (rotameric signals), 119.0, 112.6 & 112.4 (rotameric signals), 79.7 & 79.5 (rotameric signals), 58.6 & 58.3 (rotameric signals), 46.9 & 46.4 (rotameric signals), 40.4 & 39.4 (rotameric signals), 29.9 & 29.2 (rotameric signals), 28.7, 23.6 & 22.8 (rotameric signals).

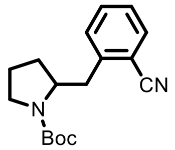

***tert*-butyl 2-(2-cyanobenzyl)pyrrolidine-1-carboxylateacetate (45).** Prepared according to GP3 from **1a** (108 mg, 0.3 mmol, 1.0 equiv.) and **2d** (106 mg, 0.3 mmol, 1.0 equiv.). Purified via flash column chromatography on silica gel (gradient from Pentane:Ethyl Acetate 98:2 to 90:10) to afford the product (mixture of rotamers) as a white solid (30 mg, 35% yield).

^{1}H NMR (400 MHz, $CDCl_3$) δ 7.61 (t, *J* = 9.4 Hz, 1H), 7.52 (td, *J* = 7.6, 1.3 Hz, 1H), 7.48 – 7.27 (m, 2H), 4.16 – 4.06 (m, 1H), 3.49 – 3.05 (m, 3H), 2.94 (dd, *J* = 13.3, 8.3 Hz, 1H), 1.97 – 1.67 (m, 4H), 1.42 (rotameric s, 9H).
^{13}C NMR (101 MHz, $CDCl_3$) δ 154.7 & 154.6 (rotameric signals), 143.5 & 143.4 (rotameric signals), 132.9, 132.6, 130.8, 127.0 & 126.8 (rotameric signals), 118.4, 113.4 & 113.3 (rotameric signals), 79.6 & 79.3 (rotameric signals), 58.7 & 58.4 (rotameric signals), 46.8 & 46.3 (rotameric signals), 38.9 & 38.1 (rotameric signals), 30.3 & 29.4 (rotameric signals), 28.6, 23.7 & 22.8. (rotameric signals)
HRMS (ESI+) (m/z): $[M+H]^+$ calcd. for $C_{17}H_{22}N_2O_2$, 287.1760; found: 287.1752.

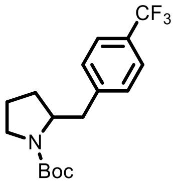

***tert*-butyl 2-(4-(trifluoromethyl)benzyl)pyrrolidine-1-carboxylate (46).** Prepared according to GP3 from **1a** (108 mg, 0.3 mmol, 1.0 equiv.) and **2c** (119 mg, 0.3 mmol, 1.0 equiv.). Purified via flash column chromatography on silica gel (Pentane:Ethyl Acetate 30:1) to afford the product as a colorless oil (46 mg, 47% yield).

Characterization data are in accordance with literature.[17]
^{1}H NMR (400 MHz, $CDCl_3$) δ 7.53 (d, *J* = 8.0 Hz, 2H), 7.32 – 7.26 (m, 2H), 4.02 – 3.98 (m, 1H), 3.40 – 3.24 (m, 2H), 3.18 – 3.07 (m, 1H), 2.63 (dd, *J* = 13.0, 9.1 Hz, 1H), 1.87 – 1.60 (m, 4H), 1.49 (s, 9H).

^{13}C NMR (101 MHz, $CDCl_3$) δ 154.6, 143.5 (q, *J* = 2 Hz), 129.9, 128.7 (q, *J* = 33 Hz), 125.4 (q, *J* = 4 Hz), 124.4 (q, *J* = 273 Hz), 79.5, 58.6, 46.7, 40.4, 29.6, 28.7, 23.2.
^{19}F NMR (282 MHz, $CDCl_3$) δ -62.4.

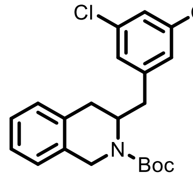

tert-butyl 3-(3,5-dichlorobenzyl)-3,4-dihydroisoquinoline-2(1H)-carboxylate **(47).** Prepared according to GP3 from **1ac** (127 mg, 0.3 mmol, 1.0 equiv.) and **2j** (119 mg, 0.3 mmol, 1.0 equiv.). Purified via flash column chromatography on silica gel (Pentane:Ethyl Acetate 4:1) to afford the product with a minor impurity. Further purification via a second flash column chromatography on silica gel (Dichloromethane) yielded the pure product (mixture of rotamers) as a colorless oil (47 mg, 40% yield).

^{1}H NMR (400 MHz, $CDCl_3$) δ 7.37 (s, 1H), 7.25 – 7.10 (m, 5H), 7.03 – 6.83 (m, 1H), 4.98 – 4.68 (m, 2H), 4.38 (d, *J* = 17.3 Hz, 1H), 3.07 (dd, *J* = 16.2, 5.6 Hz, 1H), 2.84 – 2.70 (m, 2H), 2.66 (dd, *J* = 15.9, 2.1 Hz, 1H), 1.47 – 1.26 (m, 9H).
^{13}C NMR (101 MHz, $CDCl_3$) δ 154.8, 135.4, 135.3, 133.1, 132.7, 132.3, 129.3, 127.1, 126.8, 126.6, 126.4, 80.0, 49.3 & 49.0 (rotameric signals), 43.6 & 42.9 (rotameric signals), 36.1 & 35.3 (rotameric signals), 33.5 & 32.6 (rotameric signals), 28.3.
HRMS (ESI+) (m/z): $[M+H]^+$ calcd. for $C_{21}H_{23}Cl_2NO_2$, 392.1184; found: 392.1179.

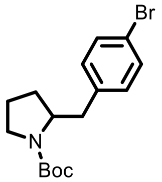

tert-butyl 2-(4-bromobenzyl)pyrrolidine-1-carboxylate **(48).** Prepared according to GP3 from **1a** (108 mg, 0.3 mmol, 1.0 equiv.) and **2i** (119 mg, 0.3 mmol, 1.0 equiv.). Purified via flash column chromatography on silica gel (Pentane:Ethyl Acetate 40:1) to afford the product (mixture of rotamers) as a colorless oil (36 mg, 35% yield).

Characterization data are in accordance with literature.[16]
^{1}H NMR (400 MHz, $CDCl_3$) δ 7.39 (d, *J* = 7.9 Hz, 2H), 7.10 – 6.99 (m, 2H), 4.03 – 3.88 (m, 1H), 3.43 – 3.22 (m, 2H), 3.15 – 2.91 (m, 1H), 2.52 (d, *J* = 12.3 Hz, 1H), 1.84 – 1.61 (m, 4H), 1.49 (s, 9H).
^{13}C NMR (101 MHz, $CDCl_3$) δ 154.6, 138.3, 131.6 & 131.4 (rotameric signals), 131.2, 120.2, 79.5 & 79.3 (rotameric signals), 58.7, 46.9 & 46.5 (rotameric signals), 40.1 & 39.1 (rotameric signals), 29.8, 28.7, 23.6 & 22.8 (rotameric signals).

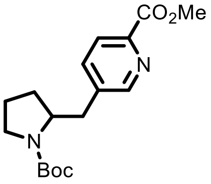

methyl 6-((1-(tert-butoxycarbonyl)pyrrolidin-2-yl)methyl)nicotinate **(49).** Prepared according to GP3 from **1a** (108 mg, 0.3 mmol, 1.0 equiv.) and **2k** (116 mg, 0.3 mmol, 1.0 equiv.). Purified via flash column chromatography on silica gel (Pentane:Ethyl Acetate 5:1) to afford the product (mixture of rotamers) as a colorless oil (60 mg, 62% yield).

^{1}H NMR (500 MHz, $CDCl_3$) δ 8.51 (d, *J* = 9.7 Hz, 1H), 8.02 (d, *J* = 8.0 Hz, 1H), 7.68 – 7.53 (m, 1H), 4.05 – 3.86 (m, 4H), 3.41 – 3.13 (m, 2H), 3.13 – 3.00 (m, 1H), 2.85 – 2.58 (m, 1H), 1.84 – 1.50 (m, 4H), 1.44 (s, 9H).
^{13}C NMR (126 MHz, $CDCl_3$) δ 165.8, 154.7 & 154.4 (*rotameric signals*), 150.7, 146.1 & 146.0 (*rotameric signals*), 138.7, 138.1 & 137.7 (*rotameric signals*), 124.9, 79.7 & 79.5 (*rotameric signals*), 58.4 & 57.9 (*rotameric signals*), 52.9, 46.9 & 46.4 (*rotameric signals*), 38.0 & 36.7 (*rotameric signals*), 30.0 & 29.2 (*rotameric signals*), 28.6, 23.6 & 22.8 (*rotameric signals*).
HRMS (GC-FI+) (m/z): $[M+H]^+$ calcd. for $C_{17}H_{24}N_2O_4$, 320.1736; found: 320.1734.

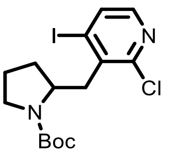

***tert*-butyl 2-((2-chloro-4-iodopyridin-3-yl)methyl)pyrrolidine-1-carboxylate** **(50).** Prepared according to GP3 from **1a** (108 mg, 0.3 mmol, 1.0 equiv.) and **2m** (147 mg, 0.3 mmol, 1.0 equiv.). Purified via flash column chromatography on silica gel (Pentane:Ethyl Acetate 3:1) to afford the product (mixture of rotamers) as a colorless oil (40 mg, 32% yield).

^{1}H NMR (400 MHz, $CDCl_3$) δ 7.86 – 7.59 (m,2H), 4.58 – 4.42 (m, 1H), 3.54 – 3.17 (m, 3H), 3.05 – 2.90 (m, 1H), 2.19 – 2.02 (m, 1H), 1.99 – 1.81 (m, 2H), 1.73 – 1.63 (m, 1H), 1.32 – 1.15 (m, 9H).
^{13}C NMR (101 MHz, $CDCl_3$) δ 154.5, 151.1, 147.1, 137.4 & 137.0 (rotameric signals), 134.1, 114.1 & 113.7 (rotameric signals), 79.4, 55.2 & 54.8 (rotameric signals), 46.5, 42.9 & 42.5 (rotameric signals), 31.3, 28.3, 23.7 & 22.9 (rotameric signals).
HRMS (ESI+) (m/z): $[M+H]^+$ calcd. for $C_{15}H_{20}ClIN_2O_2$, 422.0336; found: 422.0327.

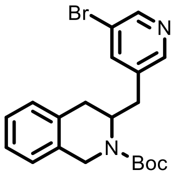

***tert*-butyl 3-((5-bromopyridin-3-yl)methyl)-3,4-dihydroisoquinoline-2(1H)-carboxylate** **(51).** Prepared according to GP3 from **1ac** (127 mg, 0.3 mmol, 1.0 equiv.) and **2o** (122 mg, 0.3 mmol, 1.0 equiv.). Purified via flash column chromatography on silica gel (gradient from Pentane:Ethyl Acetate 100:0 to 70:30) to afford the product (mixture of rotamers) as a white solid (50 mg, 41% yield).

^{1}H NMR (400 MHz, $CDCl_3$) δ 8.52 (s, 1H), 8.29 – 8.21 (m, 1H), 7.77 – 7.50 (m, 1H), 7.25 – 7.19 (m, 2H), 7.19 – 7.08 (m, 2H), 4.88 – 4.49 (m, 2H), 4.32 (d, *J* = 17.0 Hz, 1H), 3.02 (d, *J* = 15.7 Hz, 1H), 2.73 (dd, *J* = 13.8, 7.4 Hz, 1H), 2.62 (dd, *J* = 15.9, 2.2 Hz, 1H), 2.56 – 2.46 (m, 1H), 1.46 – 1.39 (m, 9H).
^{13}C NMR (101 MHz, $CDCl_3$) δ 154.8 & 154.5 (rotameric signals), 149.0, 148.7, 139.3, 136.2 & 136.1 (rotameric signals), 132.8, 132.5 & 132.2 (rotameric signals), 129.3 & 129.0 (rotameric signals), 127.1, 126.8, 126.5 & 126.3 (rotameric signals), 120.7, 80.3, 51.5 & 49.9 (rotameric signals), 43.8 & 43.0 (rotameric signals), 35.8 & 35.1 (rotameric signals), 32.9 & 32.1 (rotameric signals), 28.5.
HRMS (ESI+) (m/z): $[M+H]^+$ calcd. for $C_{20}H_{23}BrN_2O_2$, 405.1003; found: 405.1003.

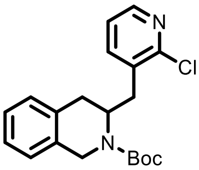

***tert*-butyl 3-((2-chloropyridin-3-yl)methyl)-3,4-dihydroisoquinoline-2(1H)-carboxylate** **(52).** Prepared according to GP3 from **1ac** (127 mg, 0.3 mmol, 1.0 equiv.) and **2h** (109 mg, 0.3 mmol, 1.0 equiv.). Purified via flash column chromatography on silica gel (Pentane:Ethyl Acetate 3:1) to afford the product (mixture of rotamers) as a colorless oil (54 mg, 50% yield).

^{1}H NMR (400 MHz, $CDCl_3$) δ 8.35 – 8.21 (m, 1H), 7.57 – 7.28 (m, 1H), 7.25 – 7.10 (m, 5H), 4.98 – 4.64 (m, 2H), 4.37 (d, *J* = 17.3 Hz, 1H), 3.21 – 2.99 (m, 1H), 2.86 – 2.59 (m, 3H), 1.42 – 1.16 (m, 9H).
^{13}C NMR (101 MHz, $CDCl_3$) δ 154.8 & 154.5 (rotameric signals), 149.0, 148.7, 139.3, 136.2 & 136.1 (rotameric signals), 132.8, 132.5 & 132.2 (rotameric signals), 129.3 & 129.0 (rotameric signals), 127.1, 126.8, 126.5 & 126.3 (rotameric signals), 120.7, 80.3, 51.5 & 49.9 (rotameric signals), 43.8 & 43.0 (rotameric signals), 35.8 & 35.1 (rotameric signals), 32.9 & 32.1 (rotameric signals), 28.5.
HRMS (ESI+) (m/z): $[M+H]^+$ calcd. for $C_{20}H_{23}ClN_2O_2$, 359.1521; found: 359.1516.

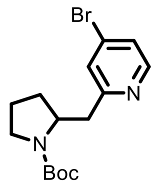

***tert*-butyl 2-((4-bromopyridin-2-yl)methyl)pyrrolidine-1-carboxylate** **(53).** Prepared according to GP3 from **1a** (108 mg, 0.3 mmol, 1.0 equiv.) and **2l** (122 mg, 0.3 mmol, 1.0 equiv.). Purified via flash column chromatography on silica gel (from Pentane:Ethyl Acetate 10:1 to 2:1) to afford the product (mixture of rotamers) as a colorless oil (49 mg, 48% yield).

^{1}H NMR (400 MHz, $CDCl_3$) δ 8.31 (d, *J* = 5.4 Hz, 1H), 7.47 – 7.27 (m, 1H), 4.12 (dd, *J* = 9.1, 4.7 Hz, 1H), 3.53 – 3.12 (m, 4H), 2.90 – 2.67 (m, 1H), 1.86 – 1.70 (m, 4H), 1.46 (s, 9H).
^{13}C NMR (101 MHz, $CDCl_3$) δ 161.3, 154.6, 150.0, 133.0, 127.2, 124.8, 79.4, 57.8, 46.9, 42.9 & 41.8 (*rotameric signals*), 30.3 & 29.4 (*rotameric signals*), 28.7, 23.6 & 22.9 (*rotameric signals*).
HRMS (ESI+) (m/z): $[M+H]^+$ calcd. for $C_{15}H_{21}BrN_2O_2$, 265.1916; found: 165.1914.

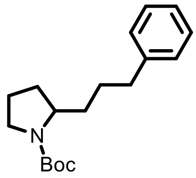

***tert*-butyl 2-(3-phenylpropyl)pyrrolidine-1-carboxylate** **(54).** Prepared according to GP3 from **1a** (216 mg, 0.6 mmol, 2.0 equiv.) and **2p** (107 mg, 0.3 mmol, 1.0 equiv.). Purified via flash column chromatography on silica gel (Pentane:Ethyl Acetate10:1) to afford the product (mixture of rotamers) as a colorless oil (43 mg, 50% yield).

Characterization data are in accordance with literature.[18]
^{1}H NMR (400 MHz, $CDCl_3$) δ 7.31 – 7.23 (m, 2H), 7.21 – 7.13 (m, 3H), 3.70 (s, 1H), 3.48 – 3.17 (m, 2H), 2.62 (q, *J* = 9.8 Hz, 2H), 1.93 – 1.70 (m, 4H), 1.66 – 1.52 (m, 3H), 1.52 – 1.31 (m, 10H).
^{13}C NMR (101 MHz, $CDCl_3$) δ 154.8, 142.8 & 142.5 (rotameric signals), 128.5, 128.4, 125.8, 79.0, 57.3, 46.6 & 46.2 (rotameric signals), 36.1, 34.5 & 34.1 (rotameric signals), 30.8 & 29.8 (rotameric signals), 28.7, 28.2, 23.9 & 23.2(rotameric signals).

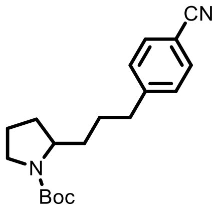

***tert*-butyl 2-(3-(4-cyanophenyl)propyl)pyrrolidine-1-carboxylate** **(55).** Prepared according to GP3 from **1a** (216 mg, 0.6 mmol, 2.0 equiv.) and **2q** (114 mg, 0.3 mmol, 1.0 equiv.). Purified via flash column chromatography on silica gel (gradient from Pentane:Ethyl Acetate 90:10 to 70:30) to afford the product (mixture of rotamers) as a colorless oil (38 mg, 40% yield).

^{1}H NMR (300 MHz, $CDCl_3$) δ 7.55 (d, *J* = 7.9 Hz, 2H), 7.26 (d, *J* = 8.0 Hz, 2H), 3.89 – 3.58 (m, 1H), 3.48 – 3.18 (m, 2H), 2.76 – 2.60 (m, 2H), 1.99 – 1.71 (m, 4H), 1.65 – 1.50 (m, 3H), 1.49 – 1.29 (m, 10H).
^{13}C NMR (101 MHz, $CDCl_3$) δ 154.8, 148.5 &148.2 (rotameric signals), 132.2, 129.3, 119.2, 109.7, 79.1, 57.1, 46.5 & 46.2 (rotameric signals), 36.2, 34.3 & 34.0 (rotameric signals), 30.9 & 30.1 (rotameric signals), 28.6, 28.0 & 27.7 (rotameric signals), 23.9 & 23.2 (rotameric signals).
HRMS (ESI+) (m/z): $[M+H]^+$ calcd. for $C_{19}H_{26}N_2O_2$, 314.1994; found: 314.2003.

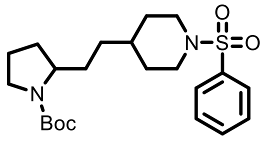

***tert*-butyl 2-(2-(1-(phenylsulfonyl)piperidin-4-yl)ethyl)pyrrolidine-1-carboxylate** **(56).** Prepared according to GP3 from **1a** (216 mg, 0.6 mmol, 2.0 equiv.) and **2r** (147 mg, 0.3 mmol, 1.0 equiv.). Purified via flash column chromatography on silica gel (gradient from Pentane:Ethyl Acetate 10:1 to 4:1) to afford the product as a pale yellow solid (48 mg, 38% yield).

^{1}H NMR (400 MHz, $CDCl_3$) δ 7.78 – 7.70 (m, 2H), 7.61 – 7.55 (m, 1H), 7.51 (dd, *J* = 8.3, 6.6 Hz, 2H), 3.83 – 3.55 (m, 3H), 3.44 – 3.17 (m, 2H), 2.21 (td, *J* = 11.8, 2.6 Hz, 2H), 1.97 – 1.50 (m, 7H), 1.42 (s, 9H), 1.34 – 1.04 (m, 6H).
^{13}C NMR (101 MHz, $CDCl_3$) δ 154.7, 136.3, 132.7, 129.0, 127.7, 79.0, 57.3, 46.6, 46.2, 35.2, 32.7, 31.8 & 31.5 (rotameric signals), 31.1 & 30.7 (rotameric signals), 29.9, 28.7, 23.9 & 23.2 (rotameric signals).
HRMS (ESI+) (m/z): $[M+H]^+$ calcd. for $C_{22}H_{34}N_2O_4S$, 423.2318; found: 423.2315.

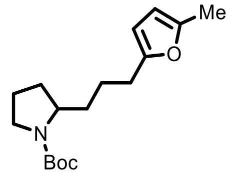

***tert*-butyl 2-(3-(5-methylfuran-2-yl)propyl)pyrrolidine-1-carboxylate** **(57).** Prepared according to GP3 from **1a** (216 mg, 0.6 mmol, 2.0 equiv.) and **2s** (108 mg, 0.3 mmol, 1.0 equiv.). Purified via flash column chromatography on silica gel (from Hexane:Ethyl Acetate 30:1 to 10:1) to afford the product as a colorless oil (26 mg, 30% yield).

^{1}H NMR (400 MHz, $CDCl_3$) δ 5.83 (s, 2H), 3.85 – 3.67 (m, 1H), 3.40 – 3.22 (m, 2H), 2.57 (q, *J* = 6.9 Hz, 2H), 2.24 (s, 3H), 1.94 – 1.72 (m, 3H), 1.68 – 1.54 (m, 4H), 1.44 (s, 9H), 1.41 – 1.24 (m, 1H).
^{13}C NMR (101 MHz, $CDCl_3$) δ 154.8, 154.5, 150.3, 105.9, 105.4, 79.0, 57.2, 46.3, 34.3, 30.8, 28.7, 28.2, 25.2, 23.3, 13.6.
HRMS (ESI+) (m/z): $[M+H]^+$ calcd. for $C_{17}H_{27}NO_3$, 294.2069; found: 294.2067

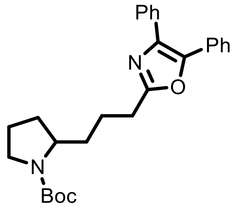

***tert*-butyl 2-(3-(4,5-diphenyloxazol-2-yl)propyl)pyrrolidine-1-carboxylate** **(58).** Prepared according to GP3 from **1a** (216 mg, 0.6 mmol, 1.0 equiv.) and **2t** (150 mg, 0.3 mmol, 1.0 equiv.). Purified via flash column chromatography on silica gel (gradient from Pentane:Ethyl Acetate 20:1 to 4:10) to afford the product (mixture of rotamers) as a colorless oil (28 mg, 22% yield).

^{1}H NMR (400 MHz, $CDCl_3$) δ 7.66 – 7.60 (m, 2H), 7.60 – 7.54 (m, 2H), 7.42 – 7.26 (m, 6H), 3.93 – 3.67 (m, 1H), 3.48 – 3.23 (m, 2H), 2.92 – 2.78 (m, 2H), 2.04 – 1.75 (m, 6H), 1.75 – 1.65 (m, 1H), 1.51 – 1.35 (m, 10H).
^{13}C NMR (101 MHz, $CDCl_3$) δ 163.5, 154.8, 145.3, 135.2, 132.7, 129.3, 128.7, 128.7, 128.5, 128.1, 128.1, 126.6, 79.2 & 79.1 (rotameric signals), 57.1, 46.4, 34.5 & 34.0 (rotameric signals), 30.9, 28.7, 28.4, 24.2, 23.0.
HRMS (ESI+) (m/z): $[M+H]^+$ calcd. for $C_{27}H_{32}N_2O_3$, 433.2491; found: 433.2493.

11. Limitation of the scope

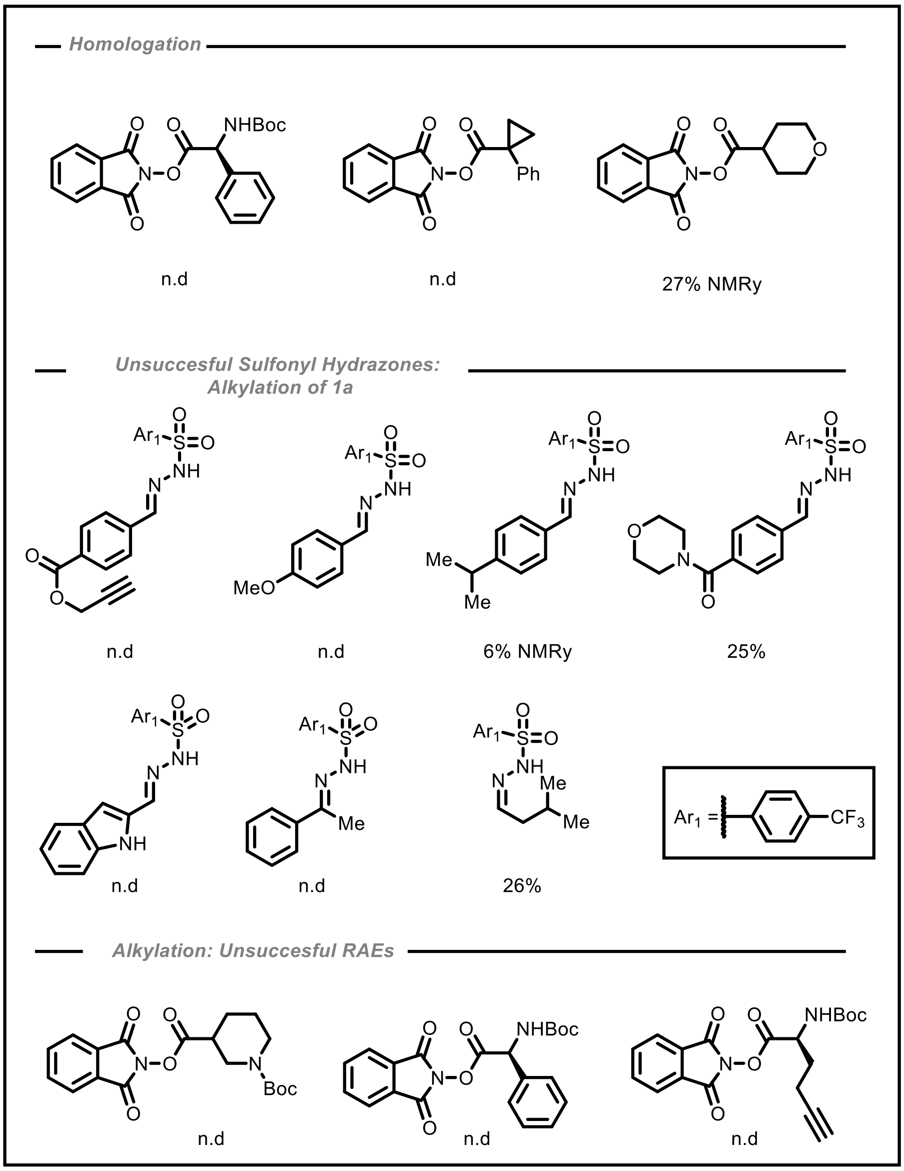

12. NMR spectra of 4-(trifluoromethyl)benzenesulfonohydrazide

^{1}H NMR (500 MHz, DMSO)

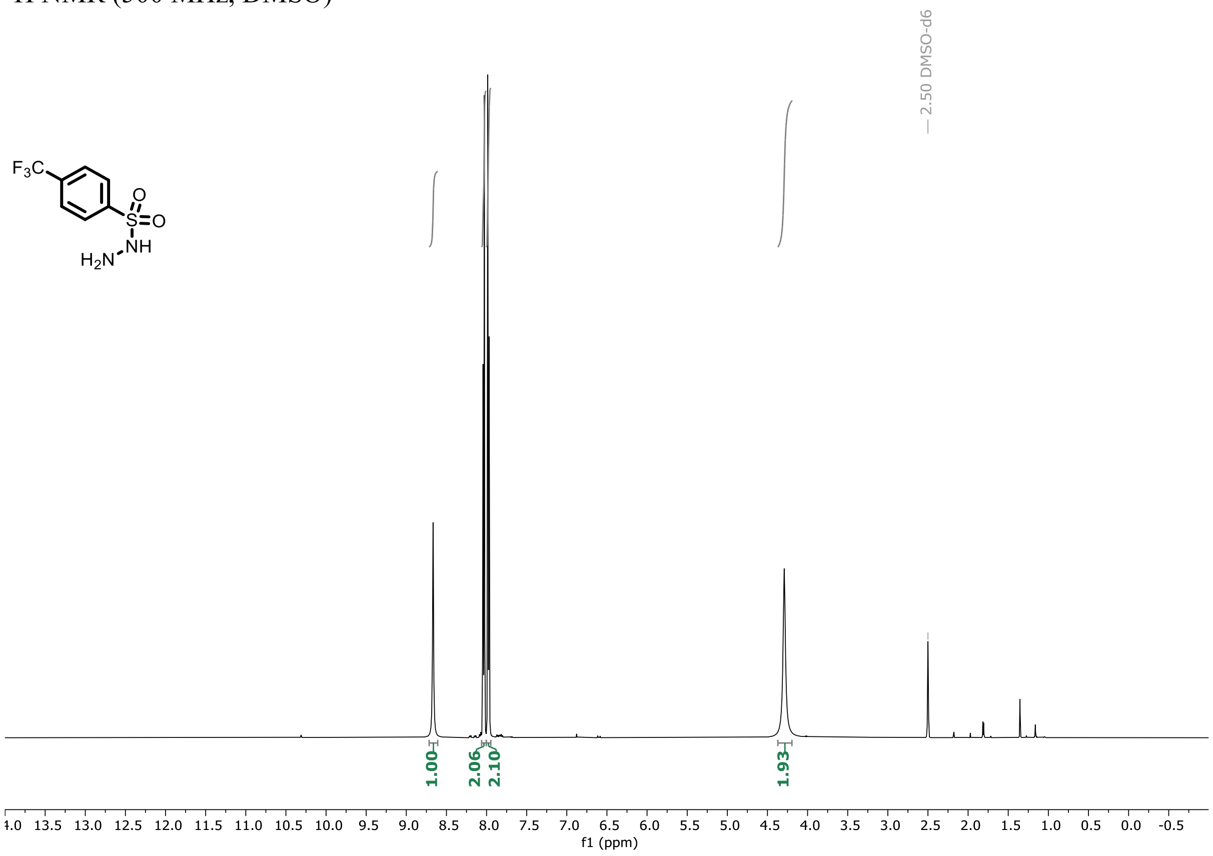

^{13}C NMR (126 MHz, DMSO) of 4-(trifluoromethyl)benzenesulfonohydrazide

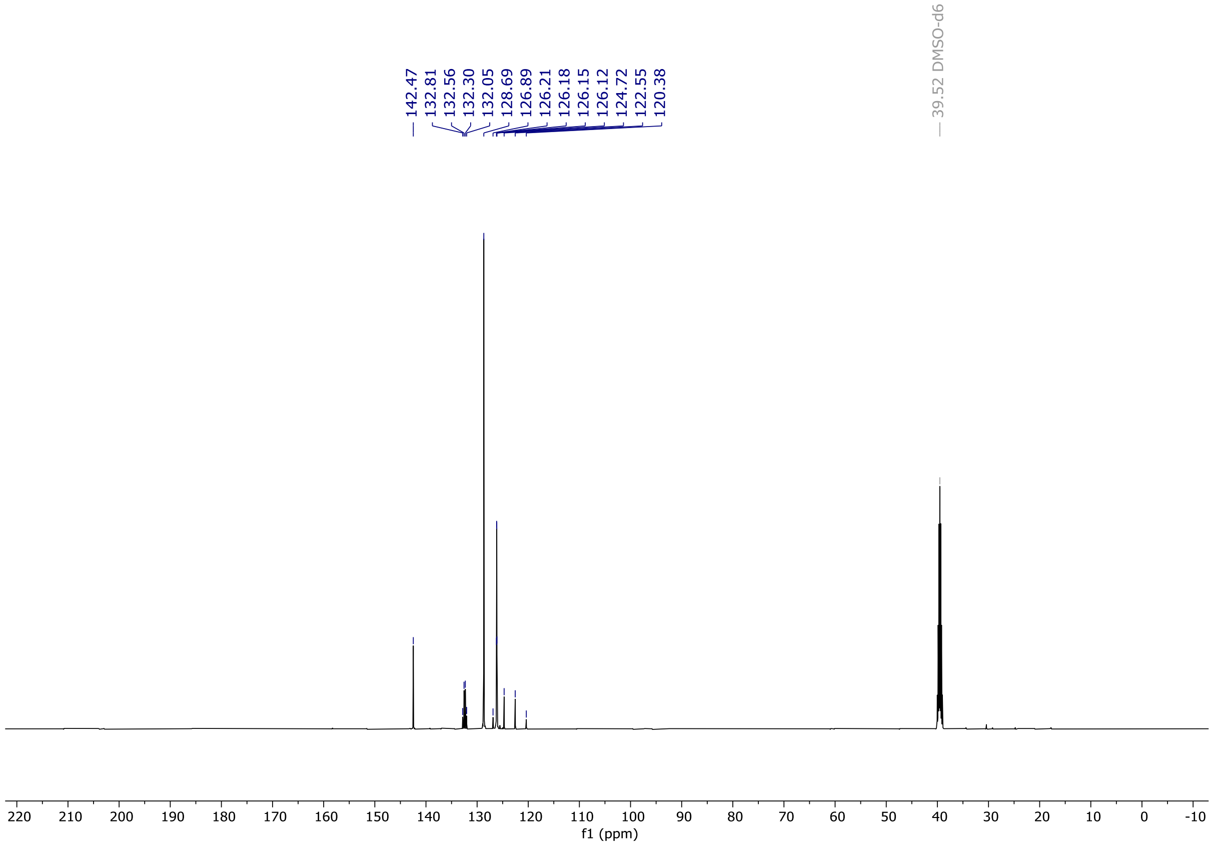
142.47
132.81
132.56
132.30
132.05
128.69
126.89
126.21
126.18
126.15
126.12
124.72
122.55
120.38
39.52 DMSO-d6
220 210 200 190 180 170 160 150 140 130 120 110 100 90 80 70 60 50 40 30 20 10 0 -10
f1 (ppm)

^{19}F NMR (470 MHz, DMSO) of 4-(trifluoromethyl)benzenesulfonohydrazide

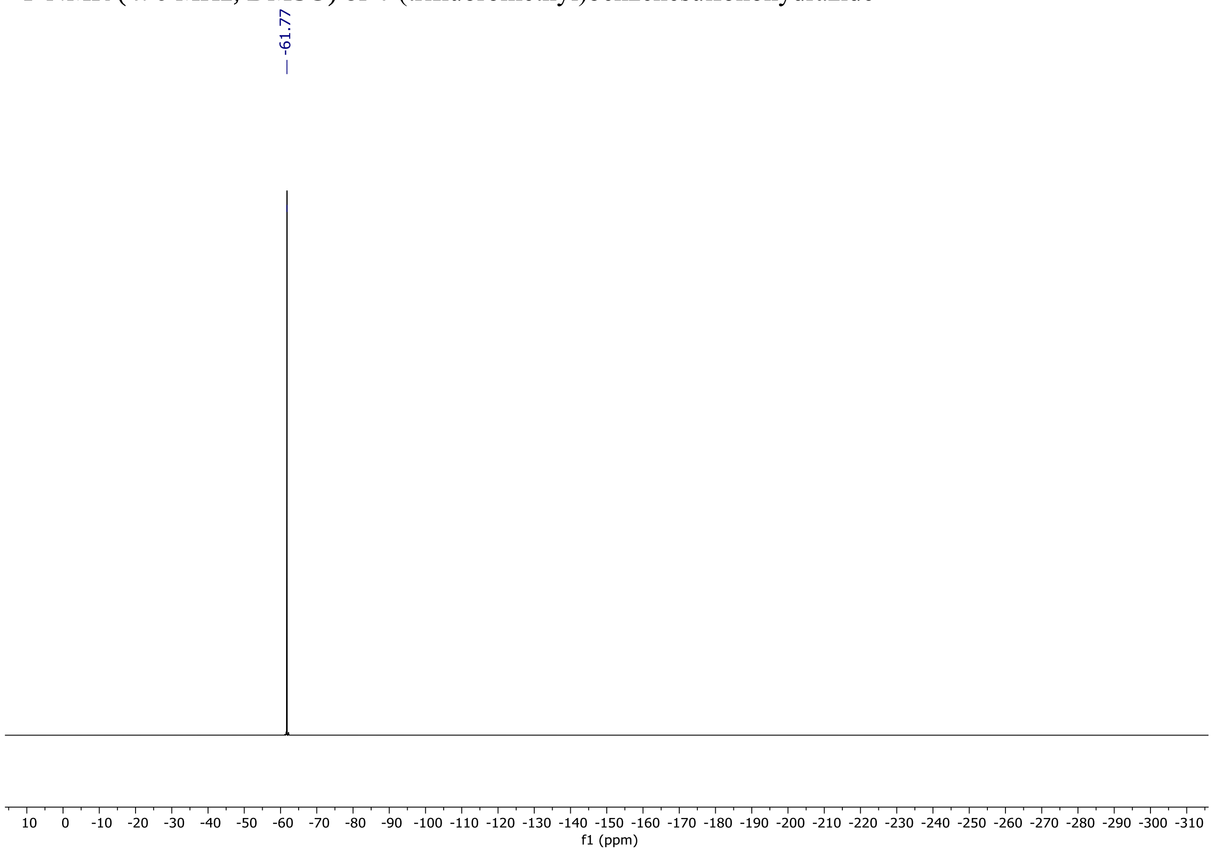

13. NMR spectra of sulfonylhydrazones 2d-2f, 2k-2m

^{1}H NMR (400 MHz, DMSO) of **2d**

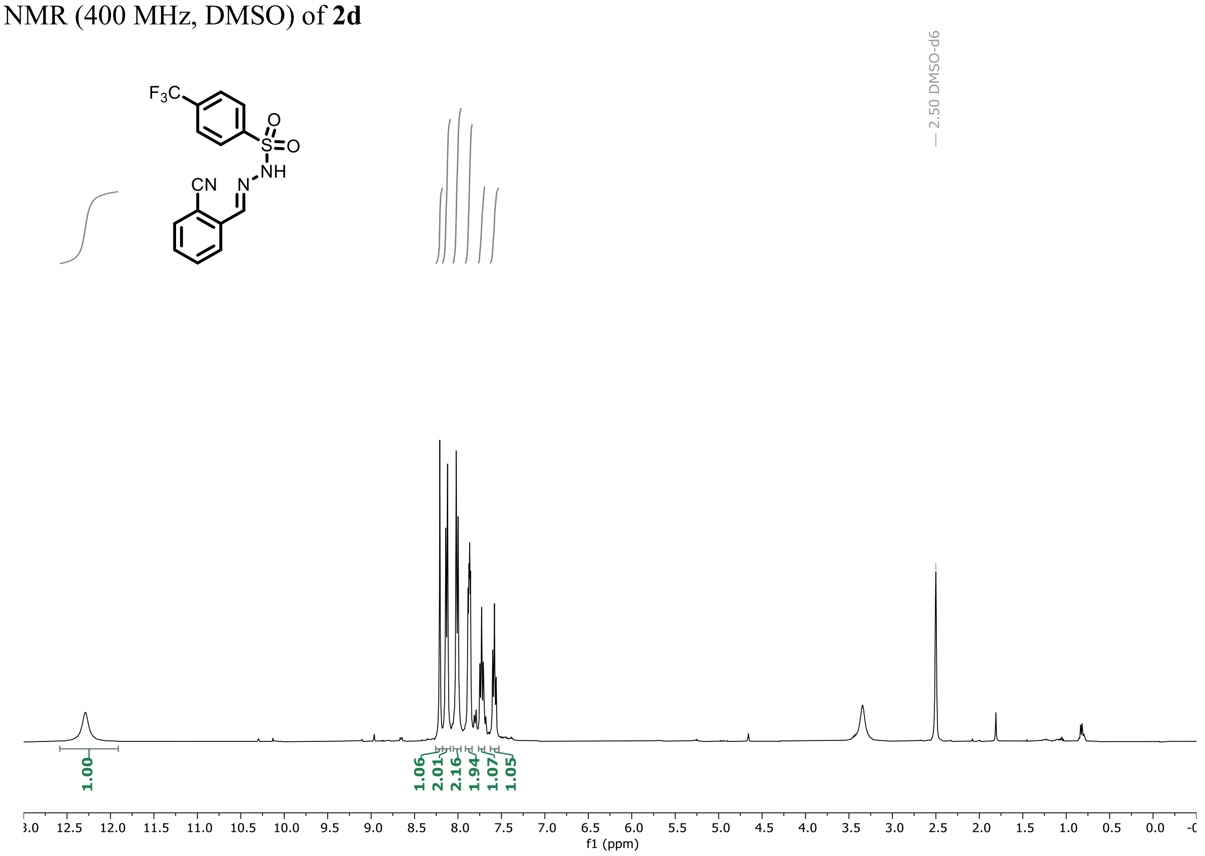

^{13}C NMR (101 MHz, DMSO) of **2d**

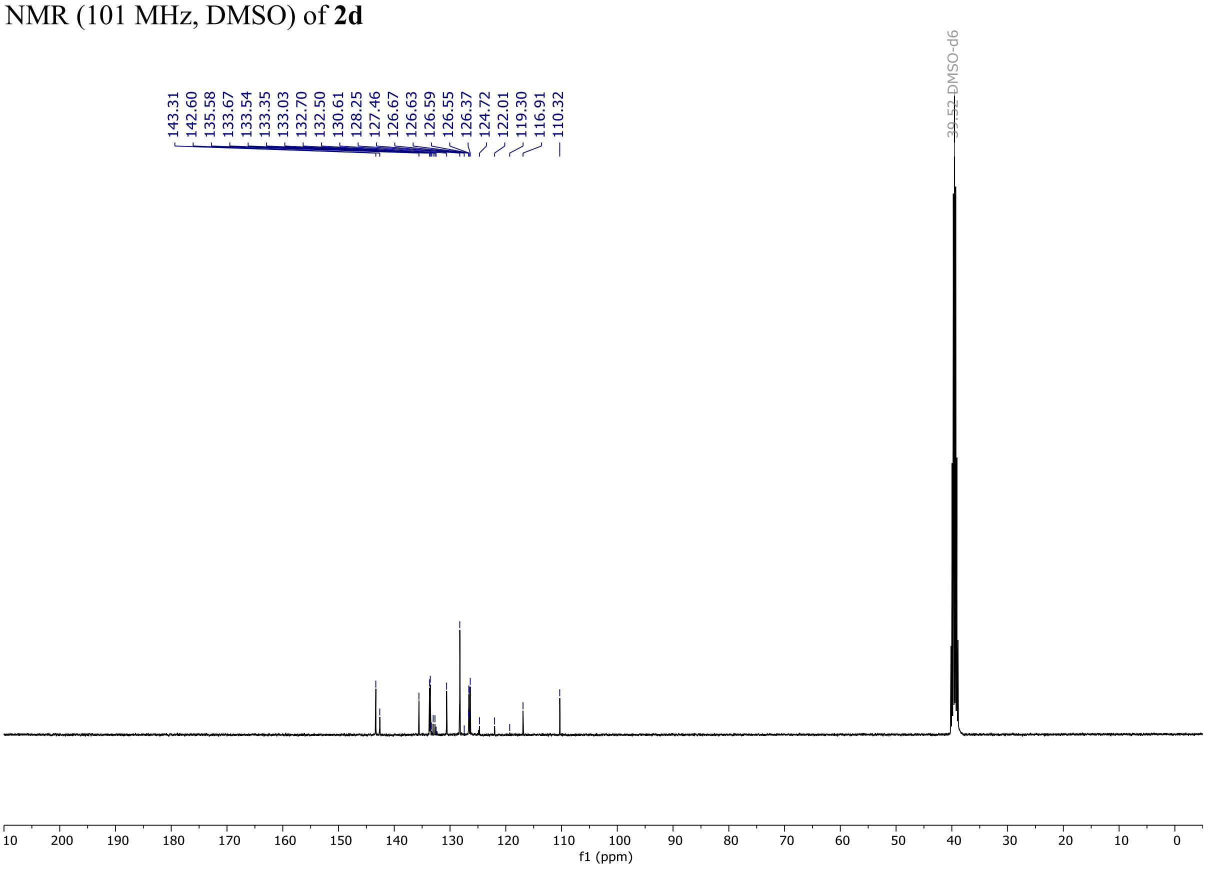

^{19}F NMR (282 MHz, DMSO) of **2d**

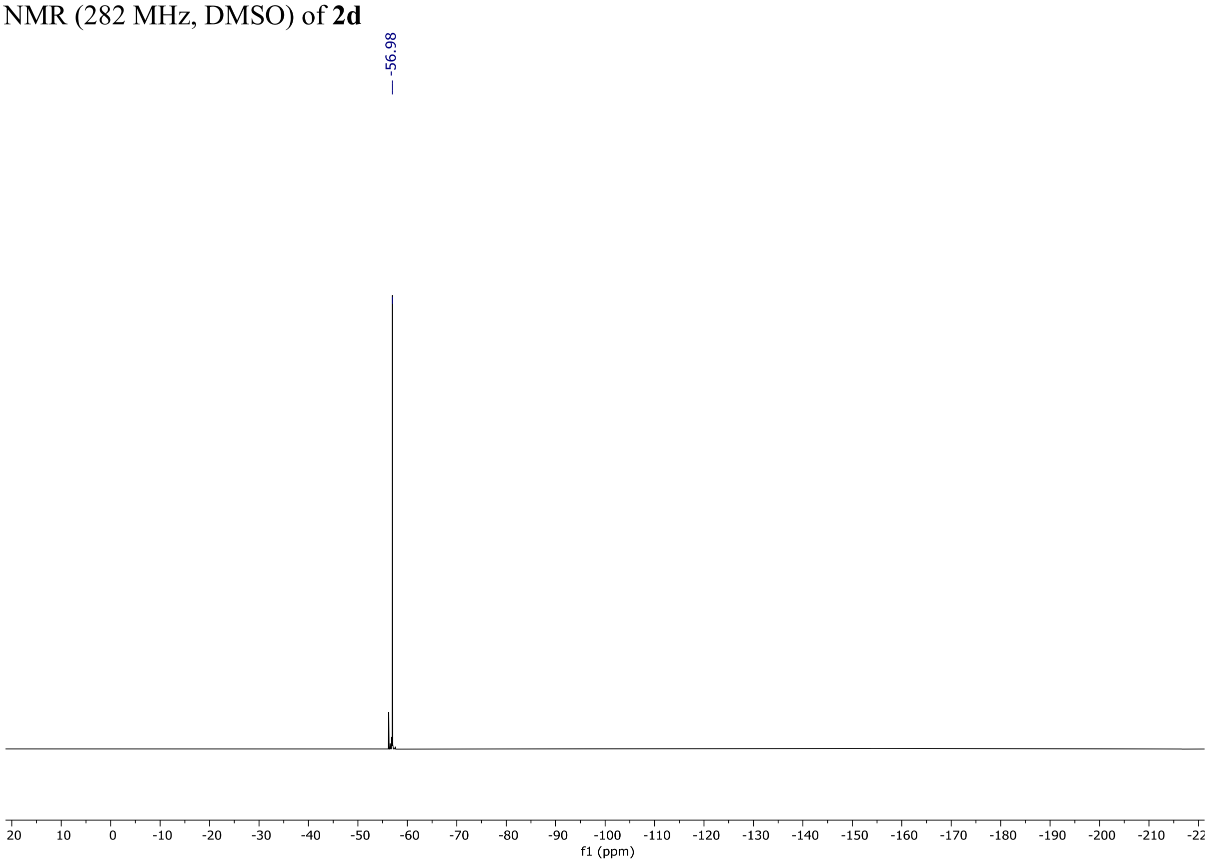

^{1}H NMR (400 MHz, DMSO) of **2e**

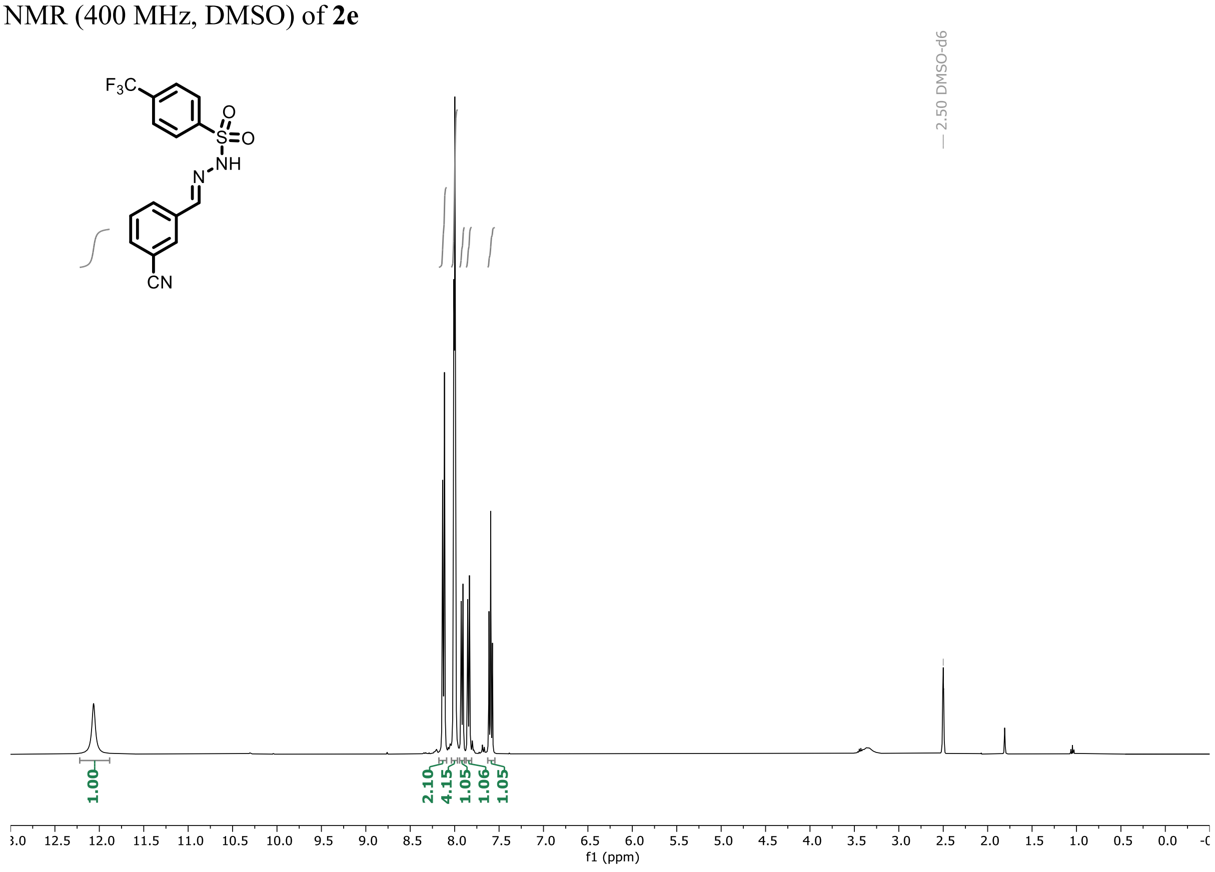

^{13}C NMR (101 MHz, DMSO) of **2e**

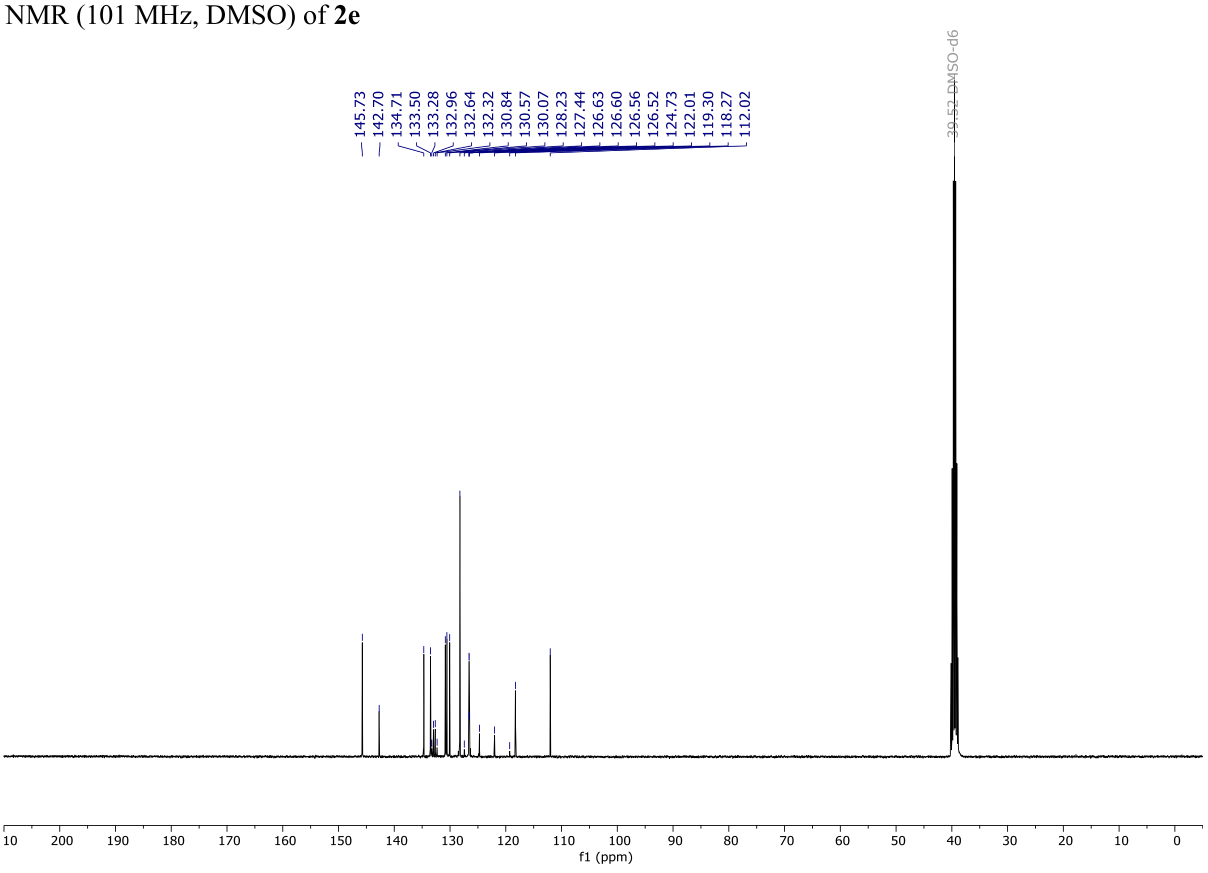

^{19}F NMR (282 MHz, DMSO) of **2e**

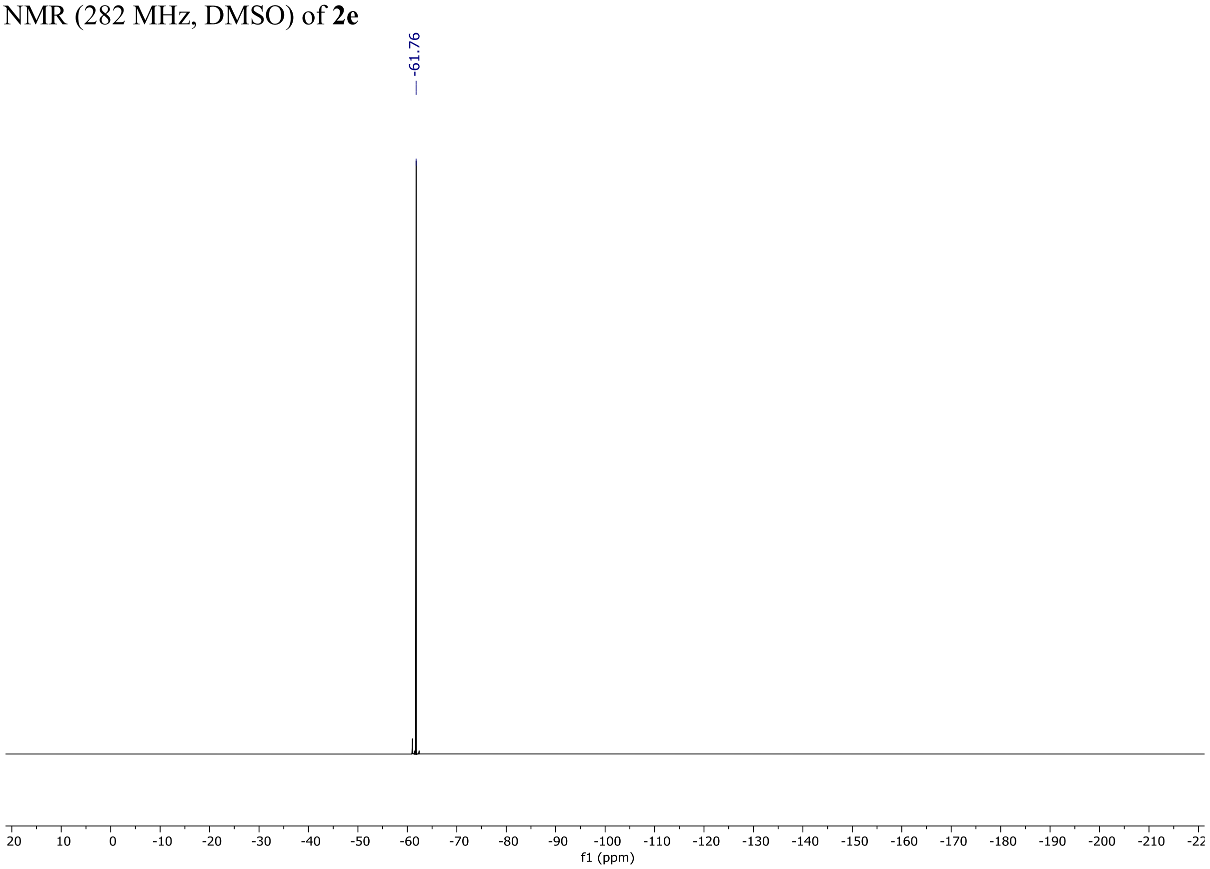

^{1}H NMR (400 MHz, DMSO) of **2f**

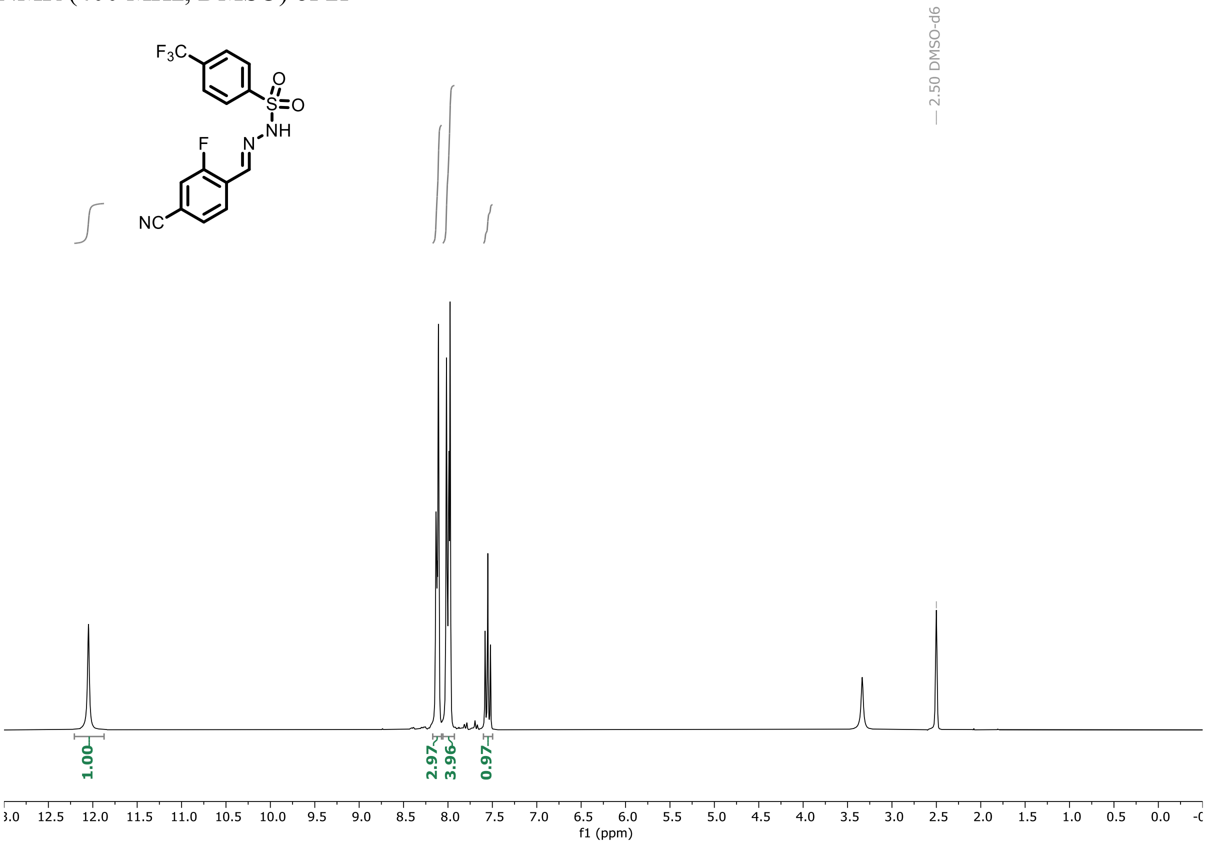

^{13}C NMR (101 MHz, DMSO) of **2f**

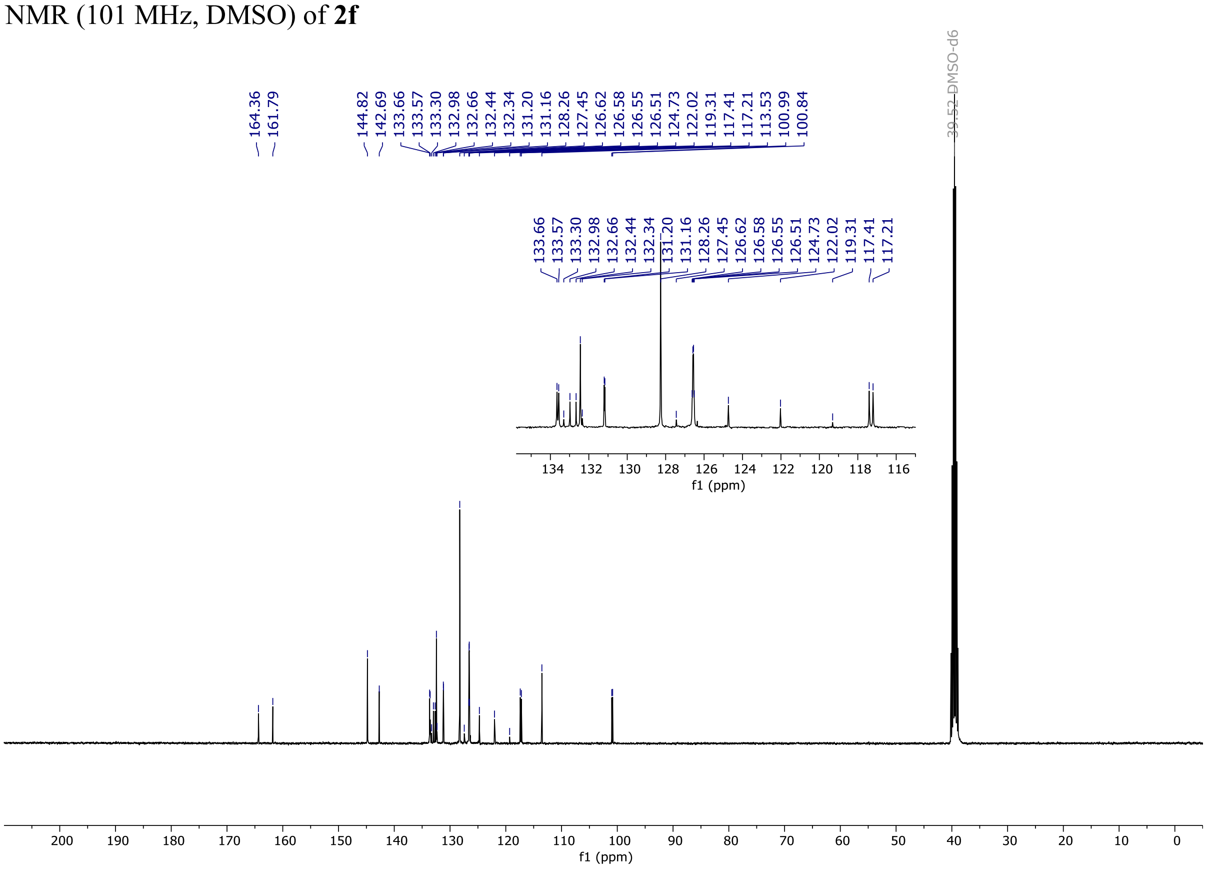

^{19}F NMR (282 MHz, DMSO) of **2f**

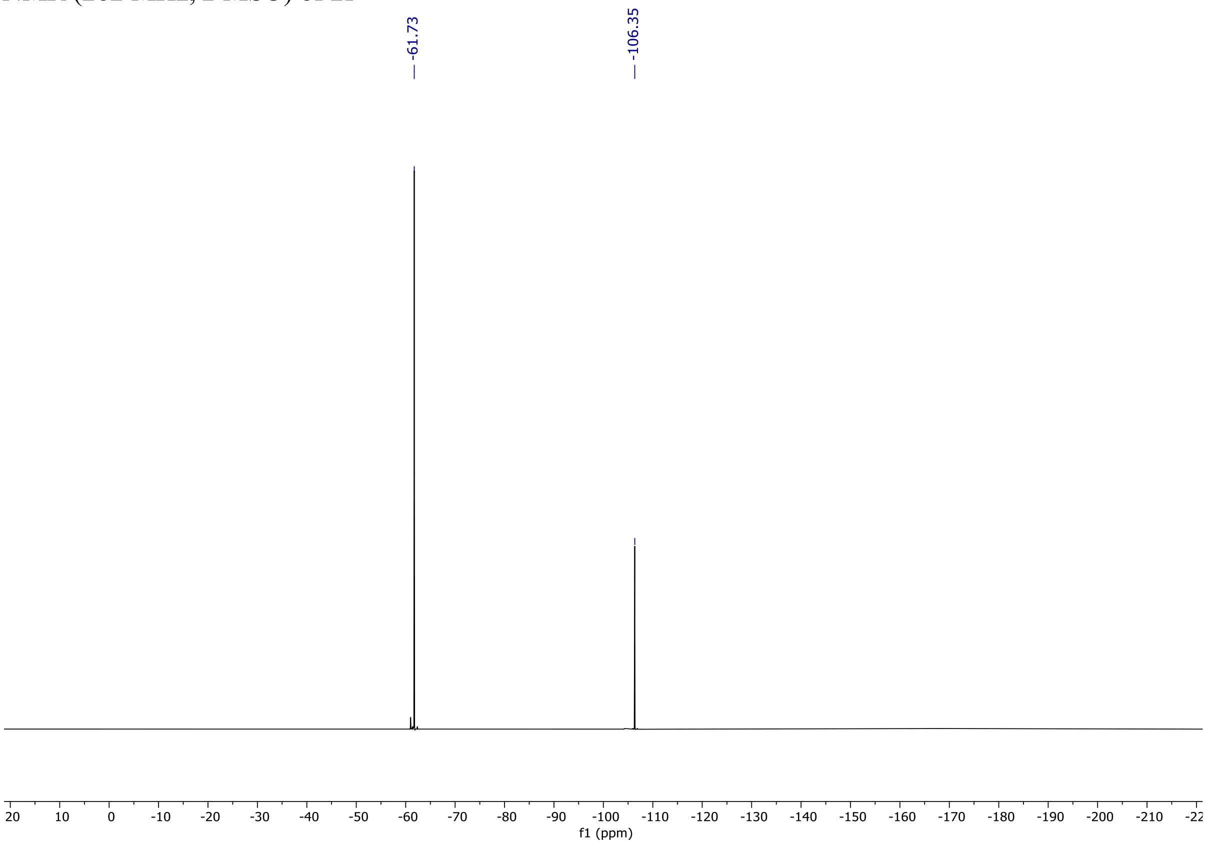

^{1}H NMR (500 MHz, DMSO) of **2k**

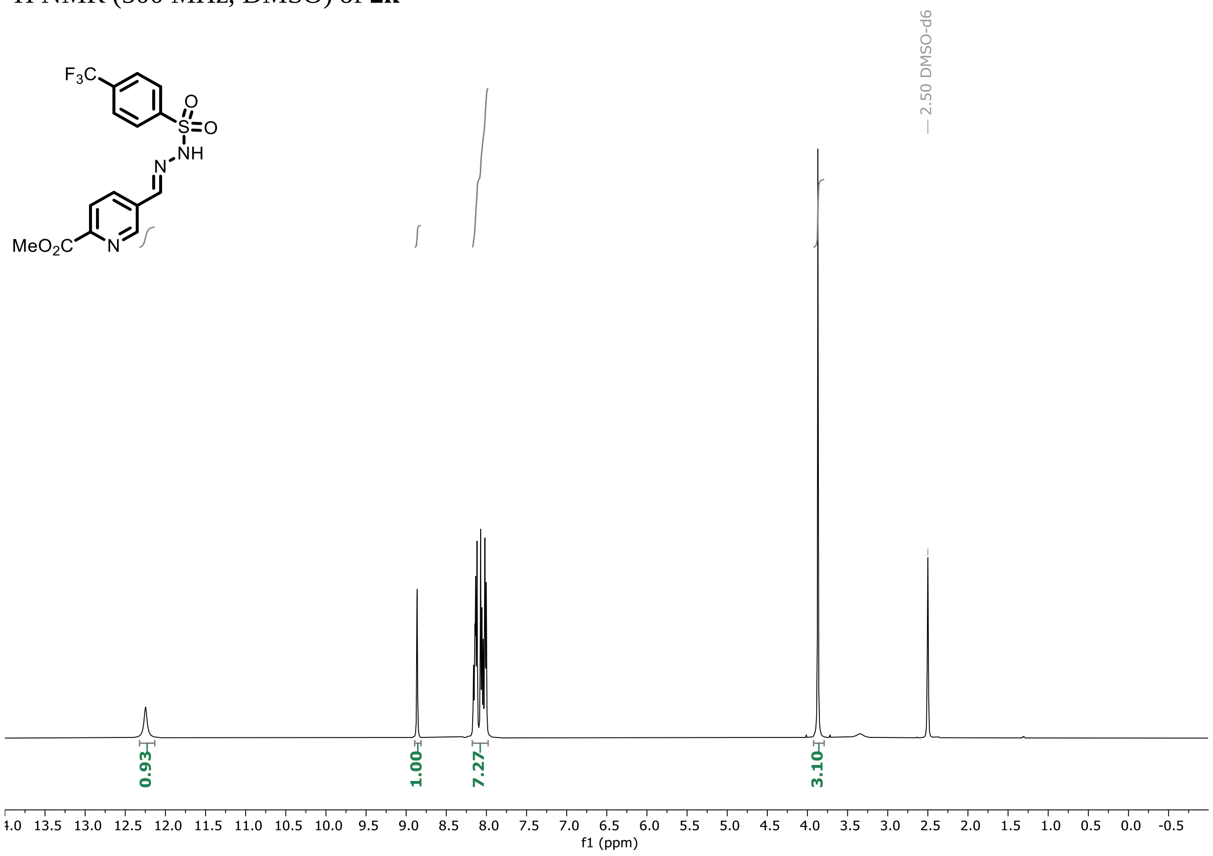

^{13}C NMR (126 MHz, DMSO) of **2k**

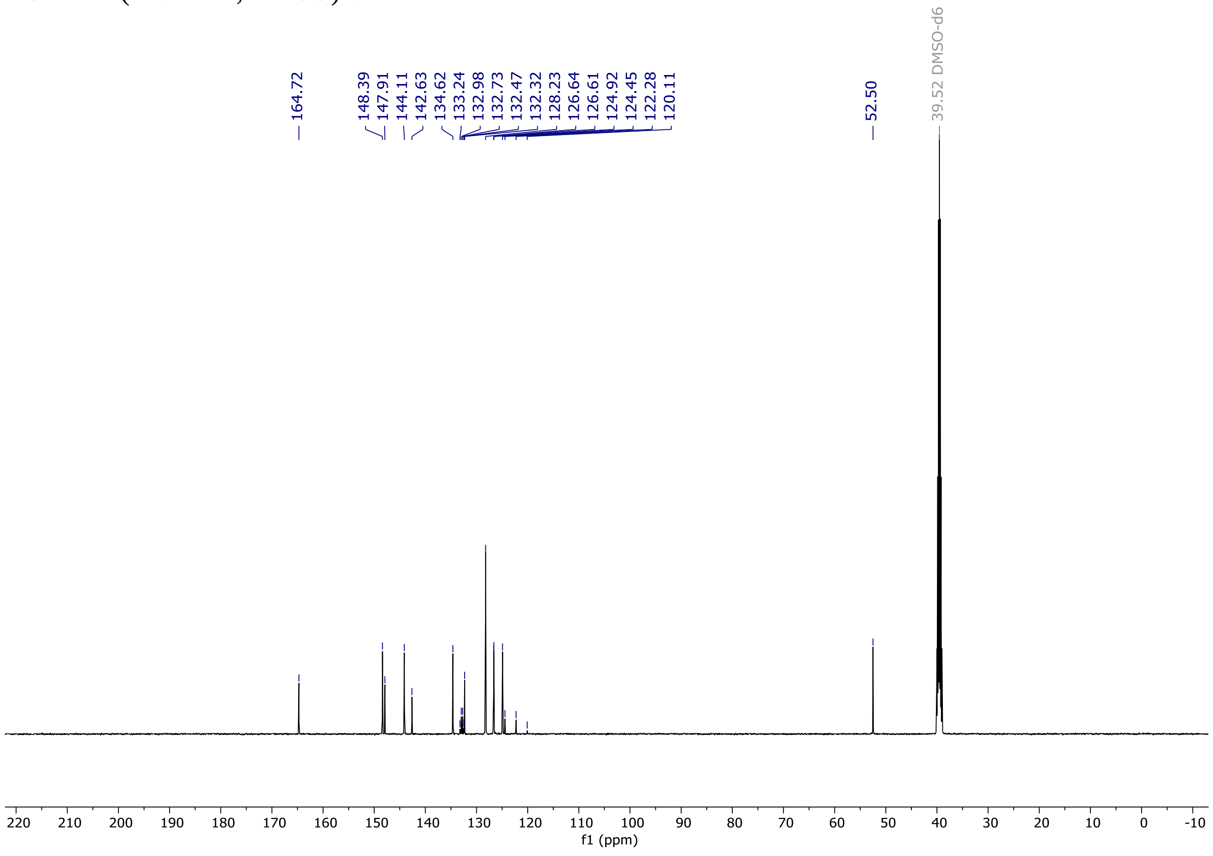

^{19}F NMR (470 MHz, DMSO) of **2k**

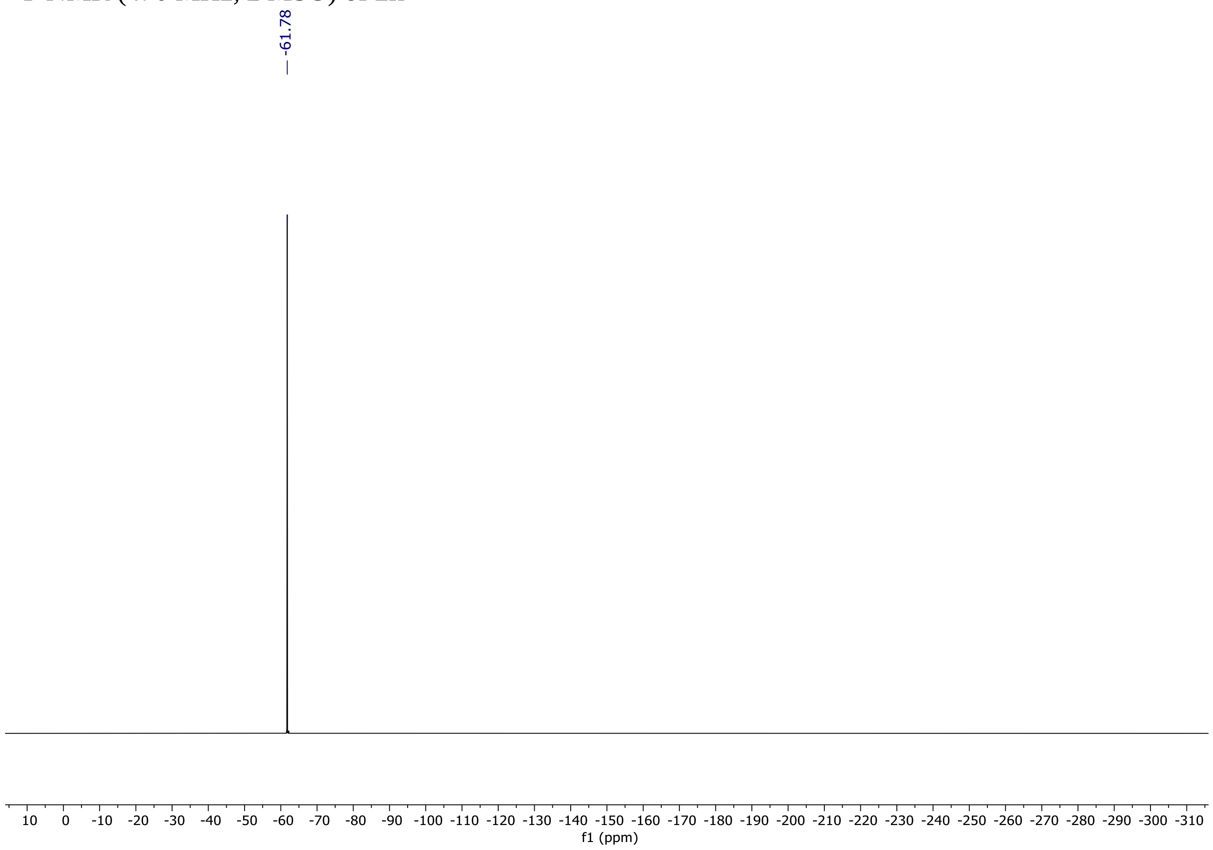

^{1}H NMR (400 MHz, DMSO) of **2l**

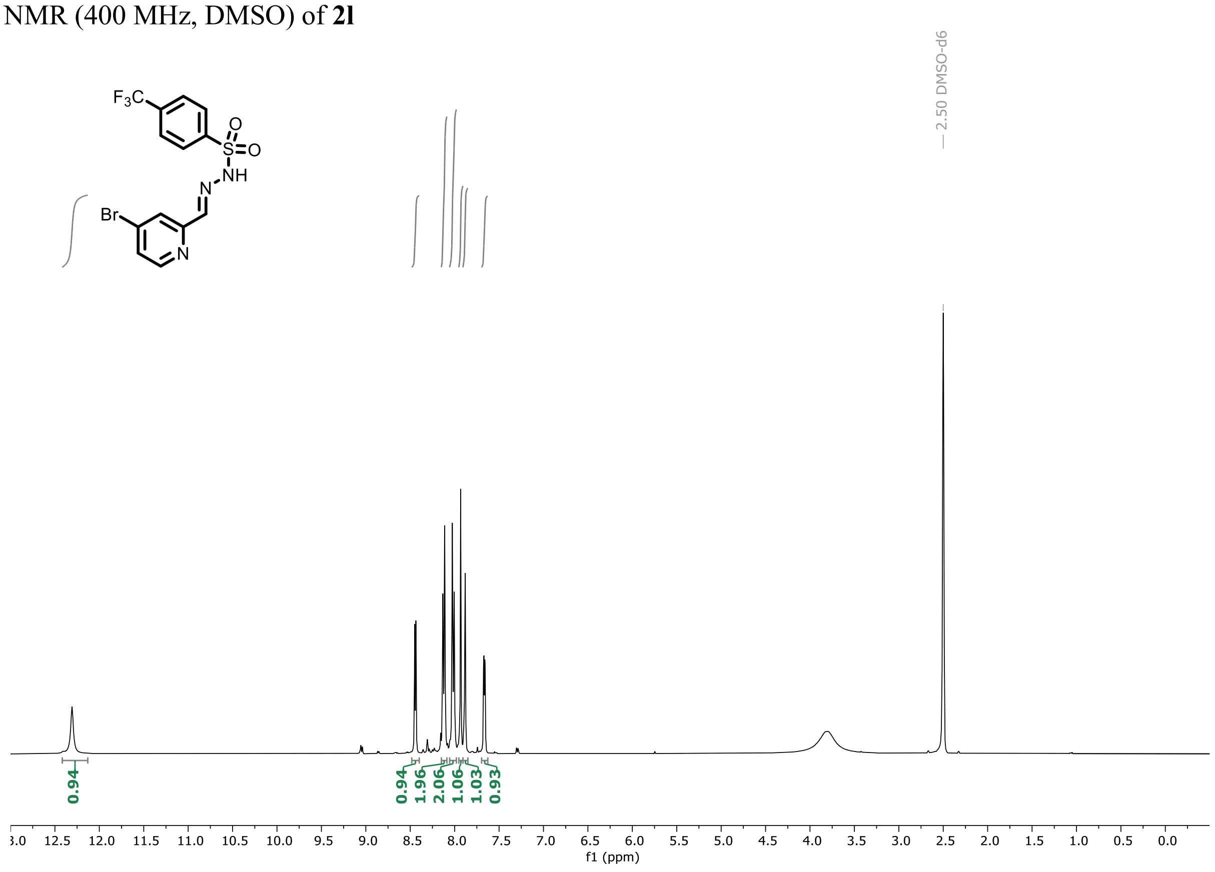

^{13}C NMR (101 MHz, DMSO) of **2l**

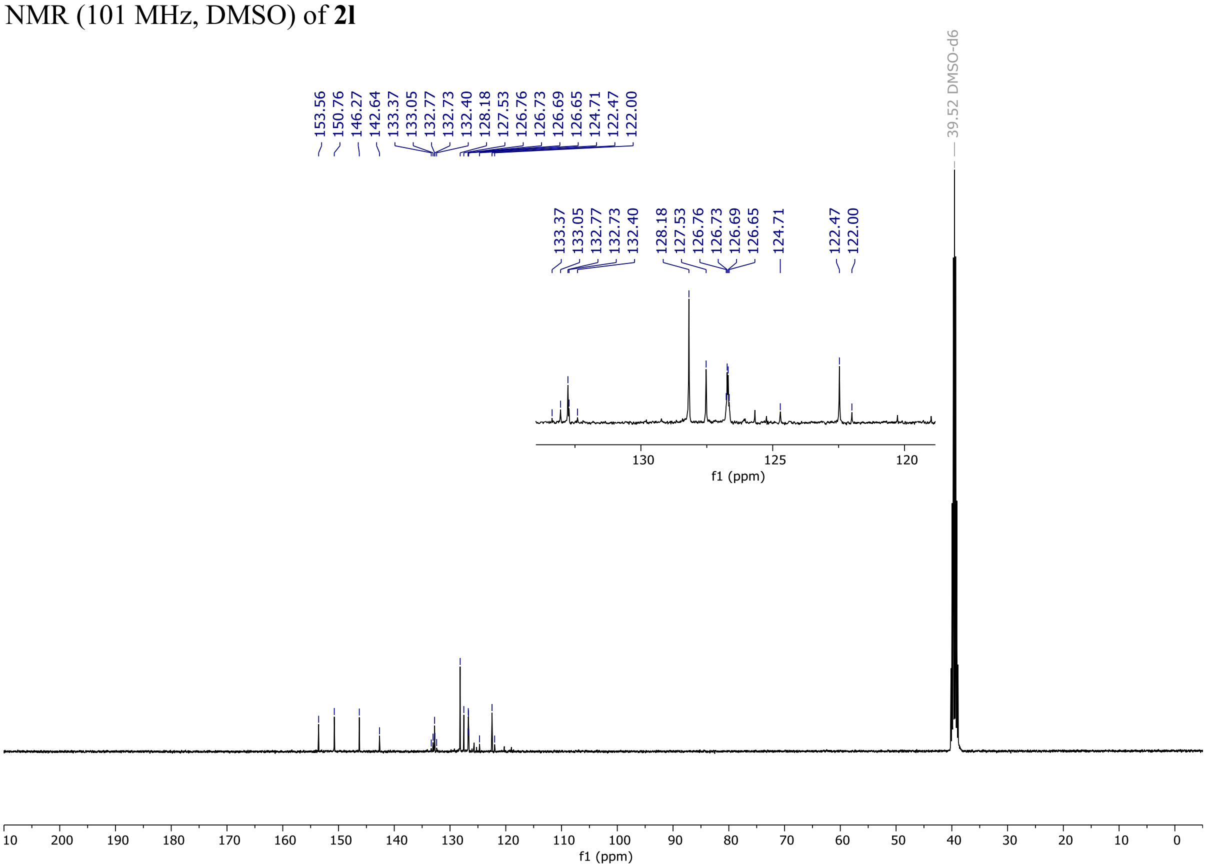

^{19}F NMR (282 MHz, DMSO) of **2l**

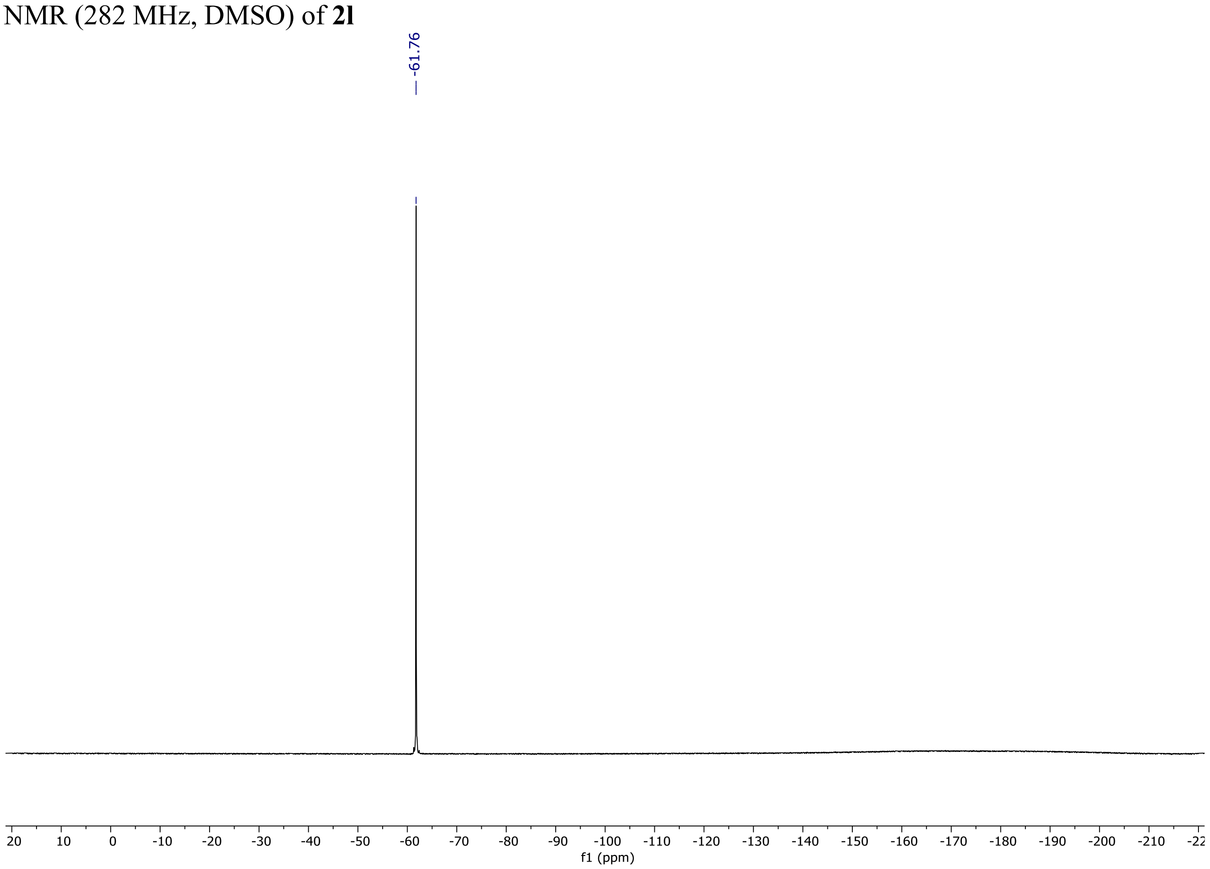

^{1}H NMR (400 MHz, DMSO) of **2m**

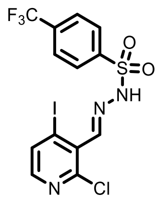

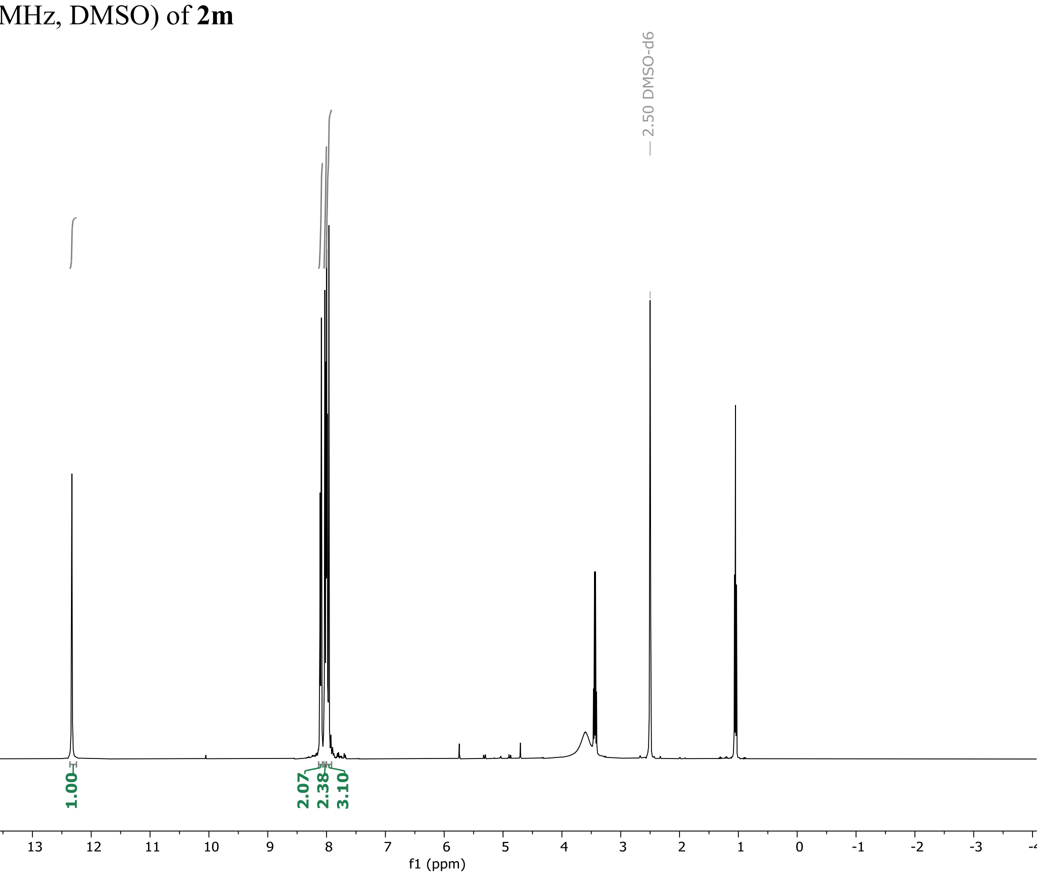

^{13}C NMR (101 MHz, DMSO) of **2m**

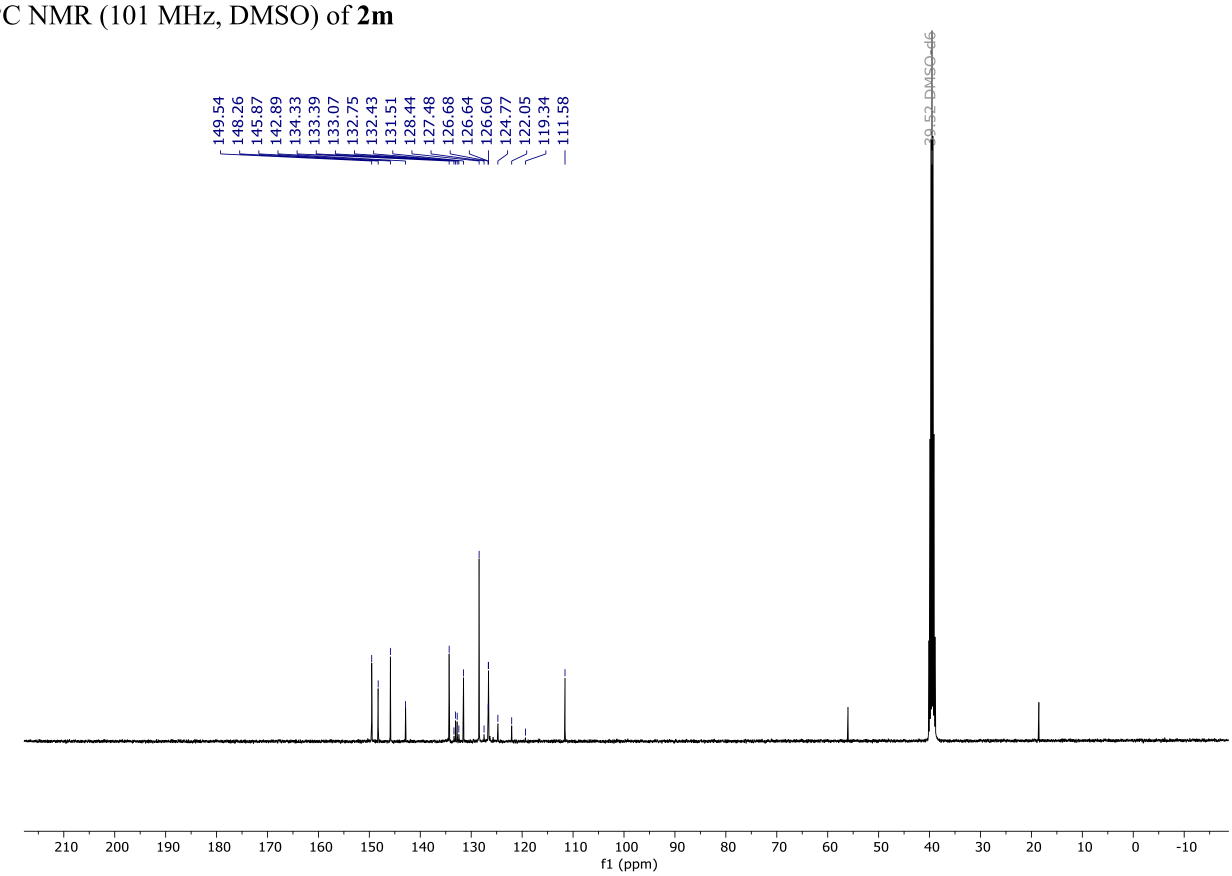

^{19}F NMR (282 MHz, $CDCl_3$) of **2m**

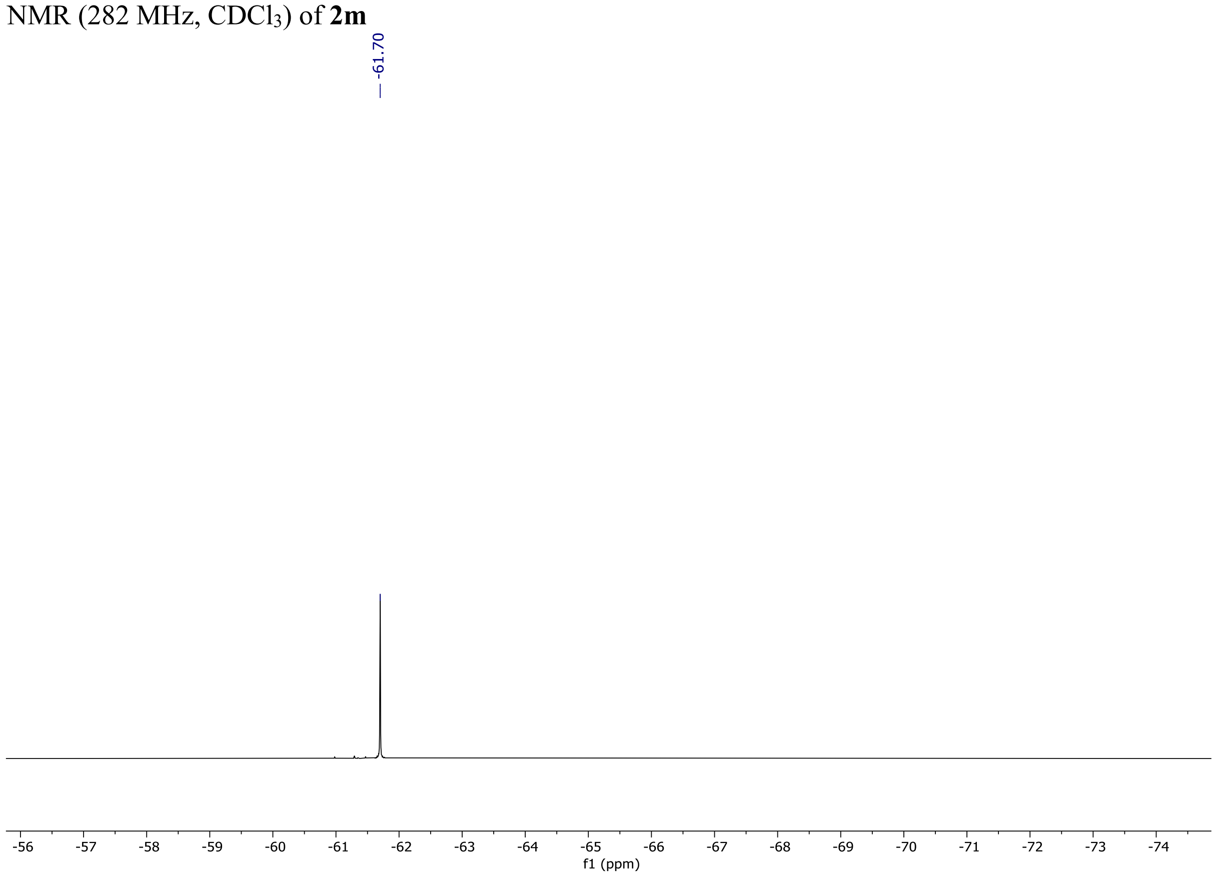

14. NMR spectra of NHPI esters 1c, 1e, 1f, 1t, 1u, 1ab-1af

^{1}H NMR (500 MHz, $CDCl_3$) of **1c**

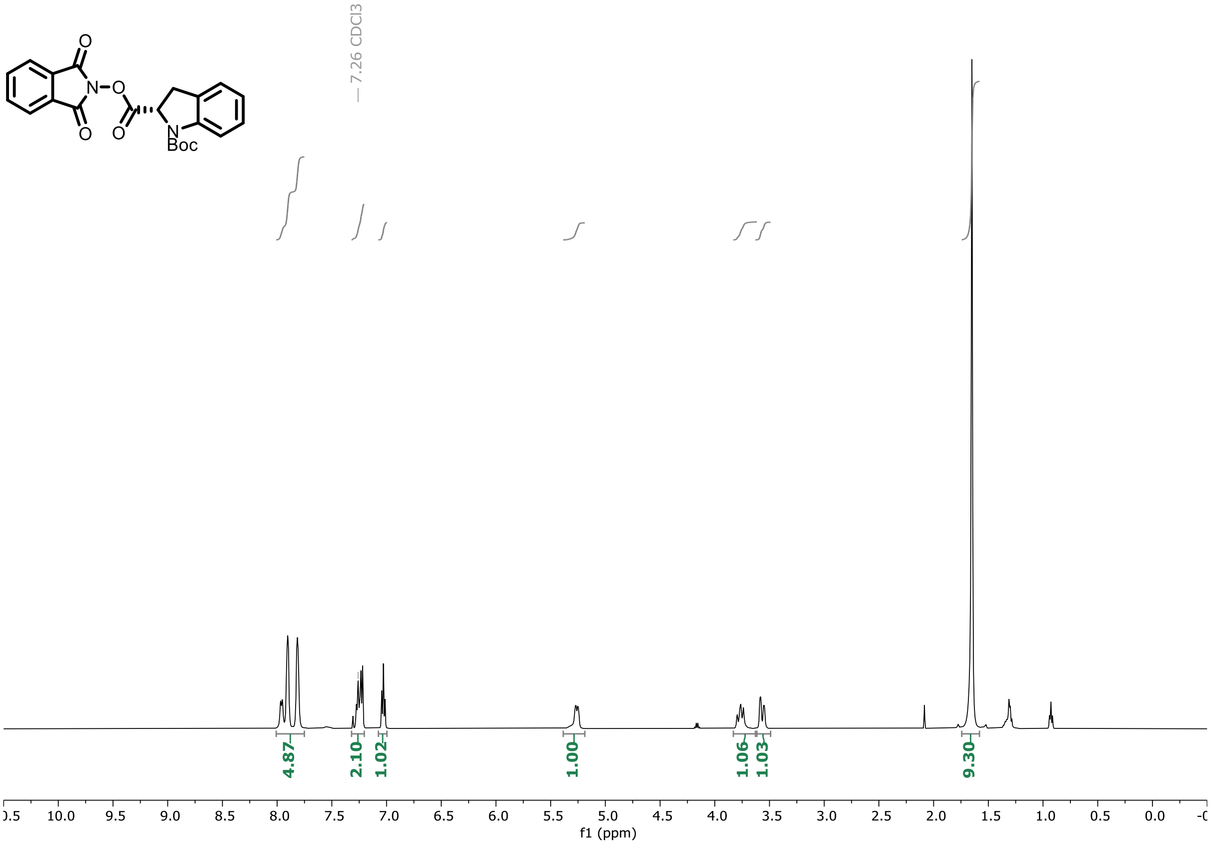

^{13}C NMR (126 MHz, $CDCl_3$) of **1c**

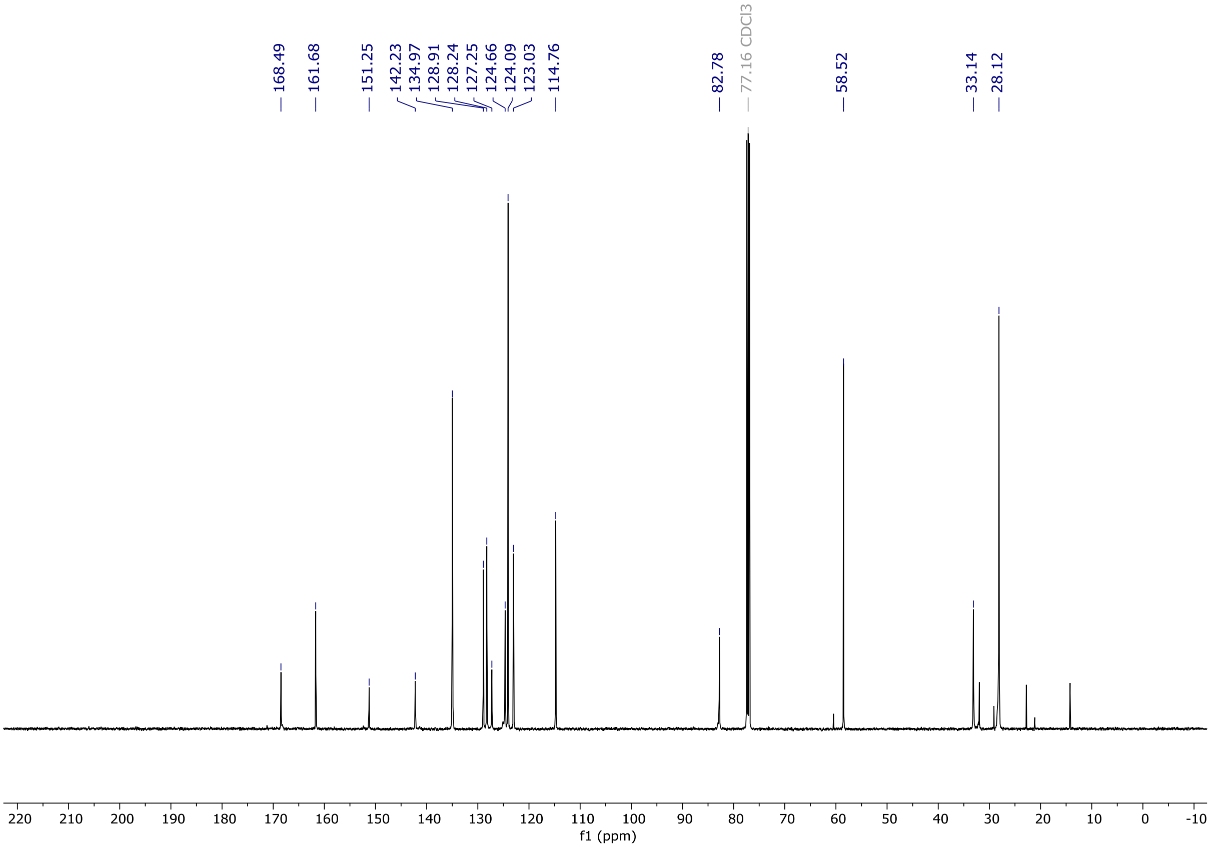

^{1}H NMR (400 MHz, $CDCl_3$) of **1e**

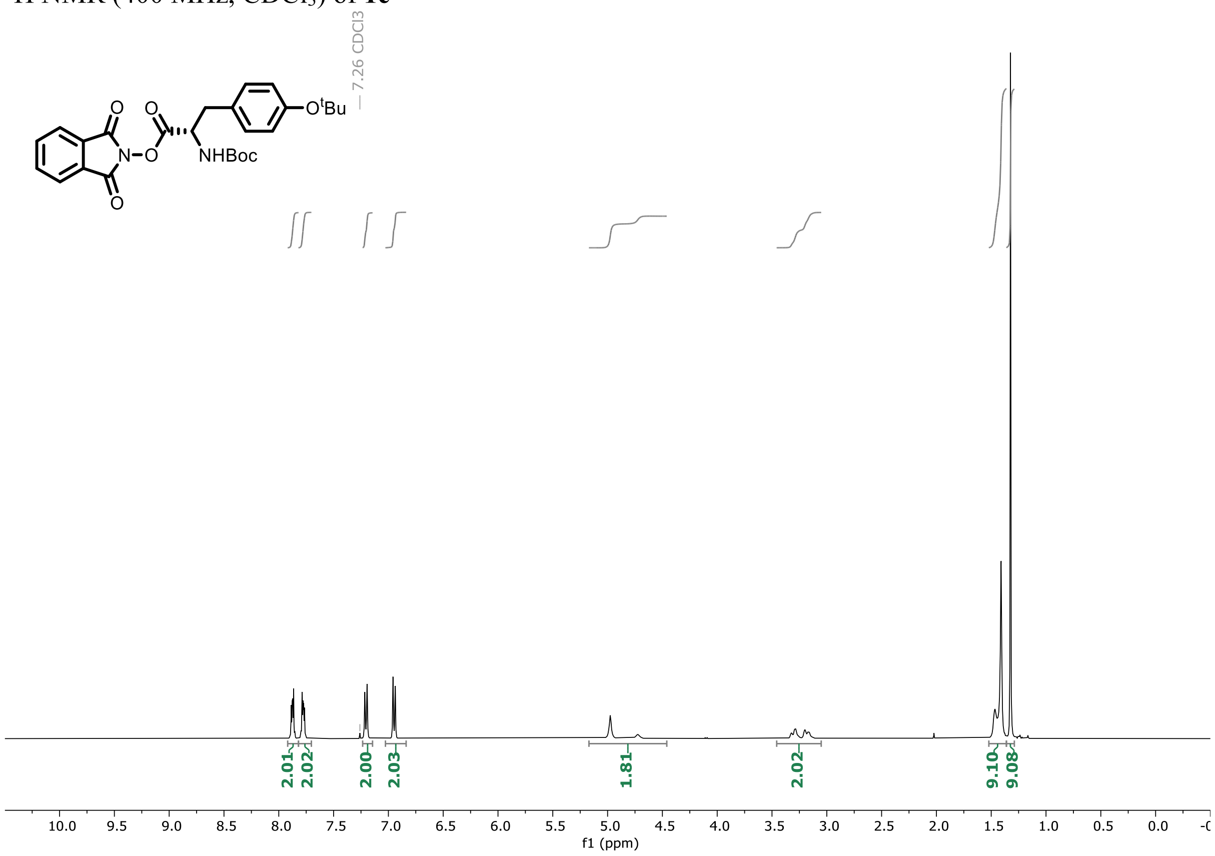

^{13}C NMR (101 MHz, $CDCl_3$) of **1e**

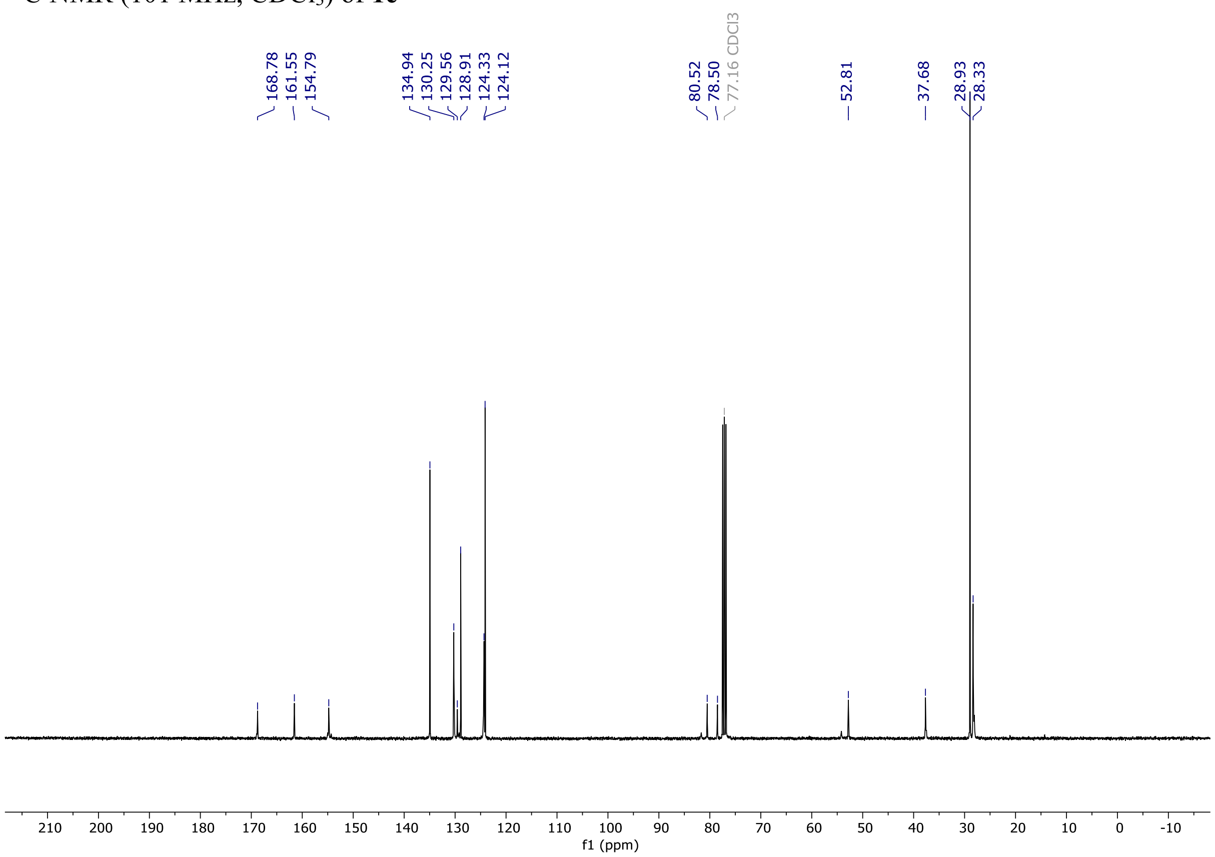

^{1}H NMR (400 MHz, $CDCl_3$) of **1f**

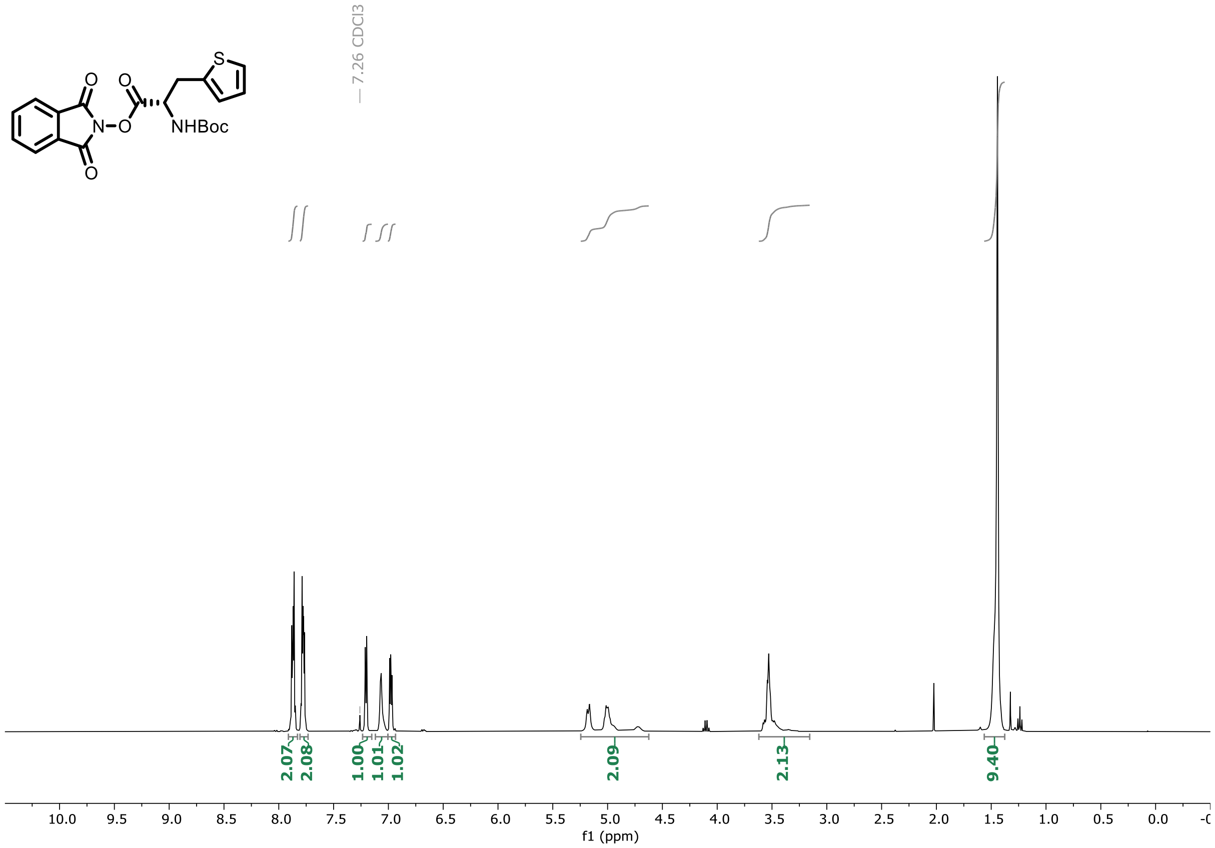

^{13}C NMR (101 MHz, $CDCl_3$) of **1f**

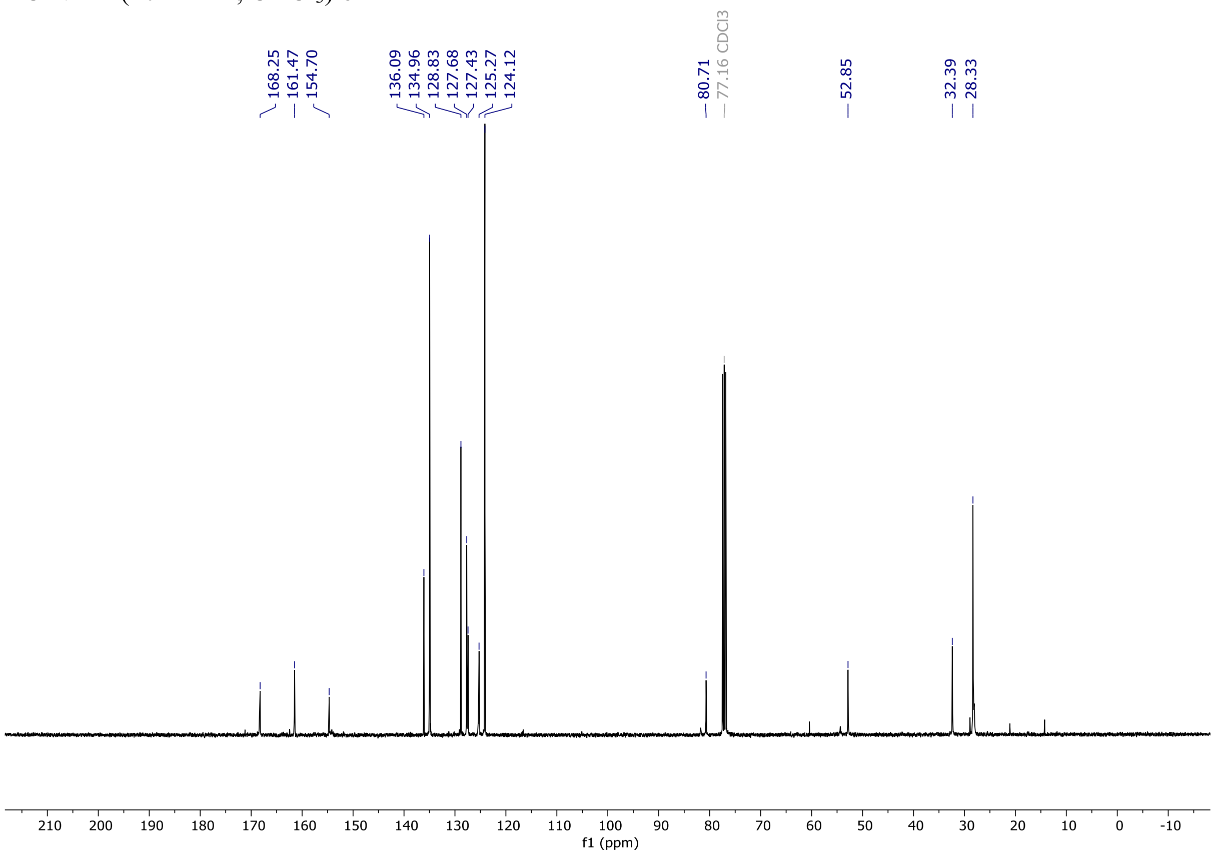

^{1}H NMR (400 MHz, $CDCl_3$) of **1t**

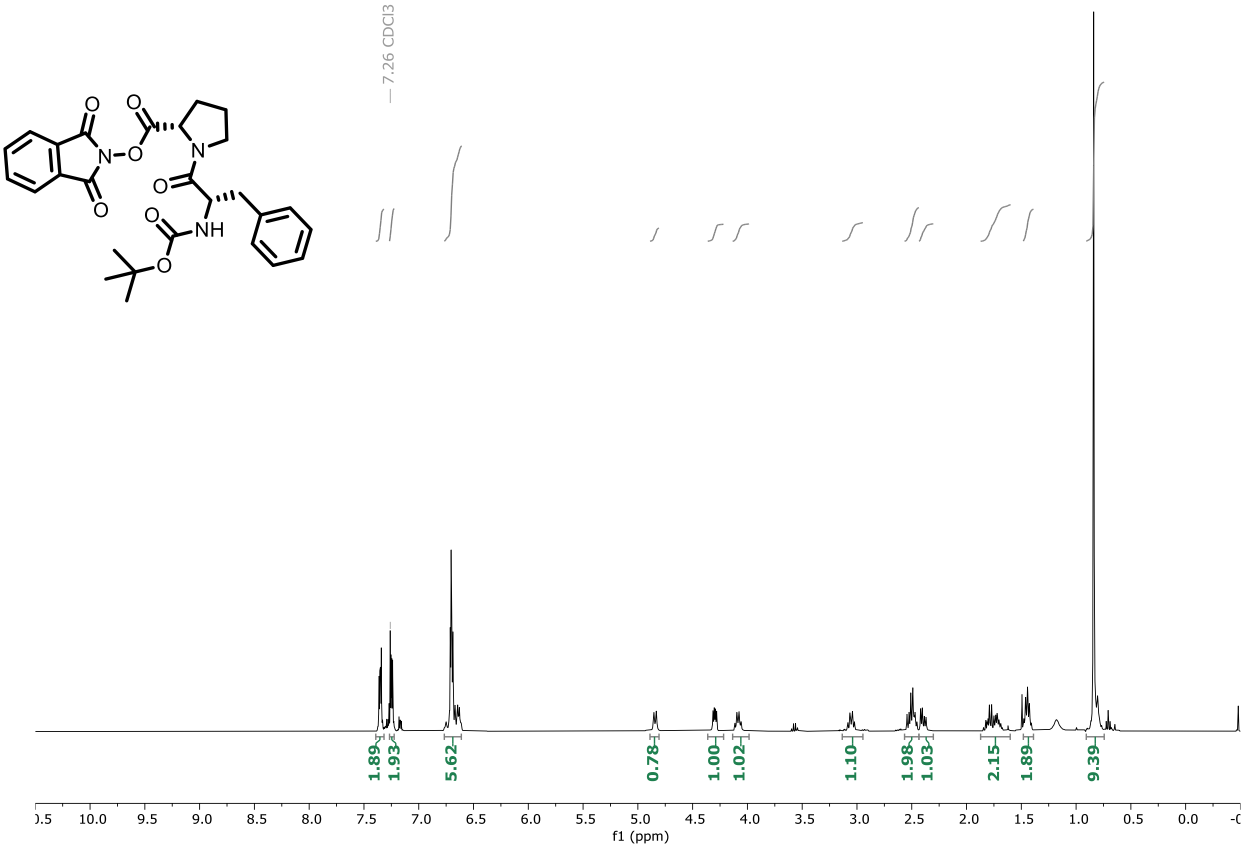

^{13}C NMR (101 MHz, $CDCl_3$) of **1t**

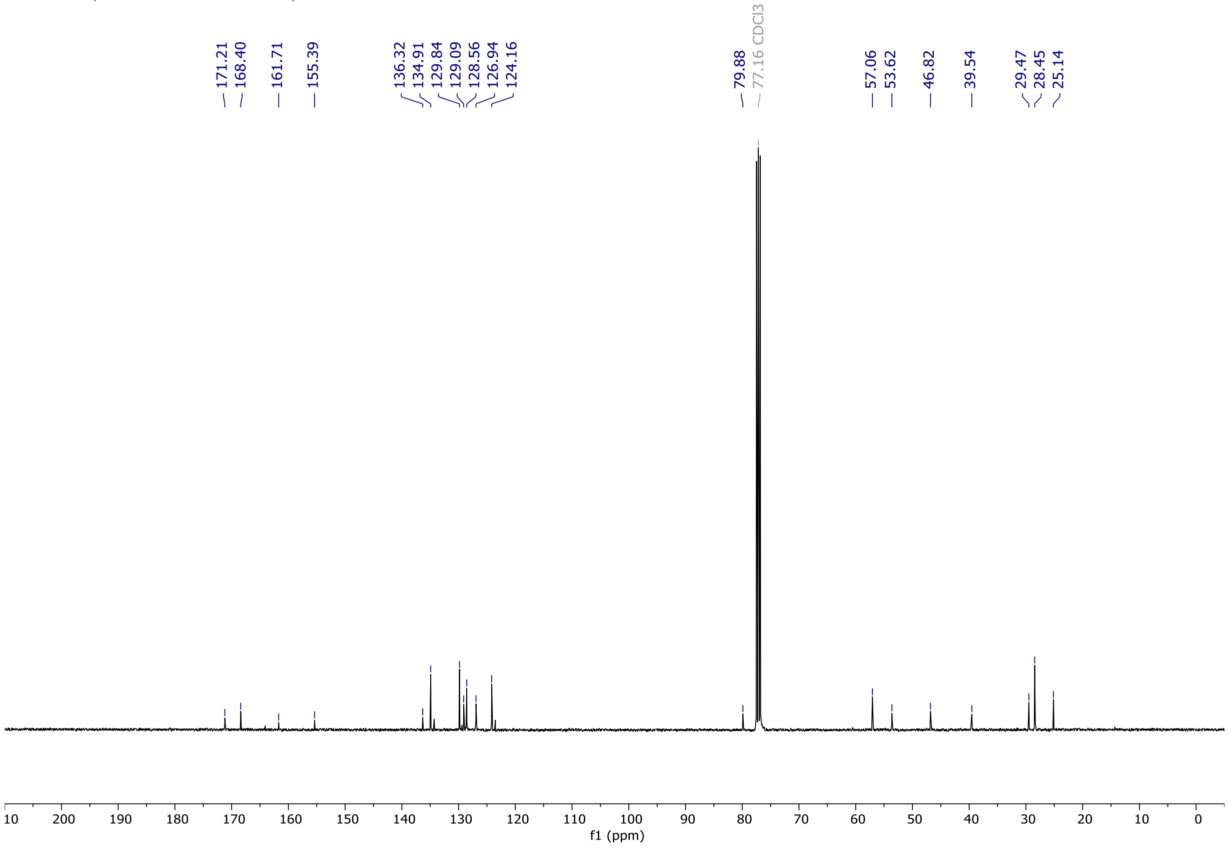

^{1}H NMR (400 MHz, $CDCl_3$) of **1u**

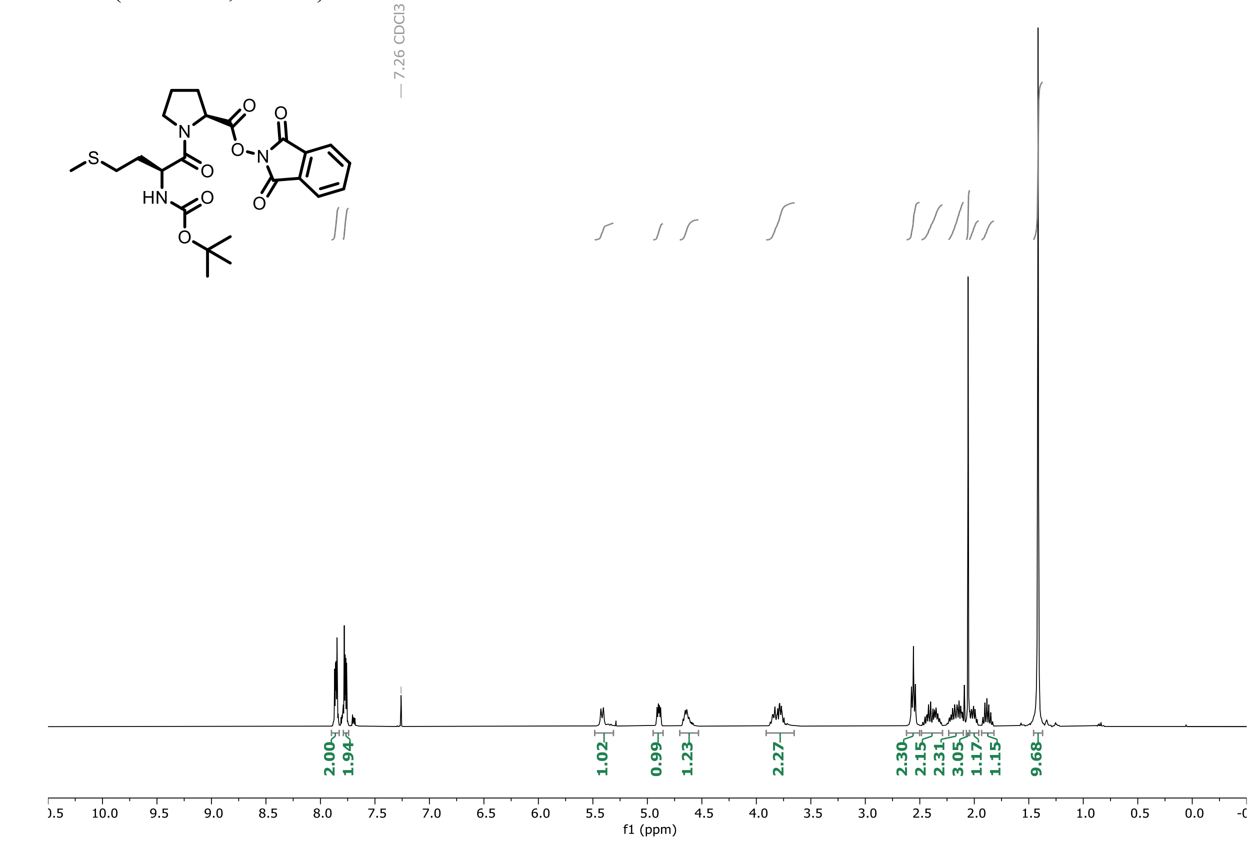

^{13}C NMR (101 MHz, $CDCl_3$) of **1u**

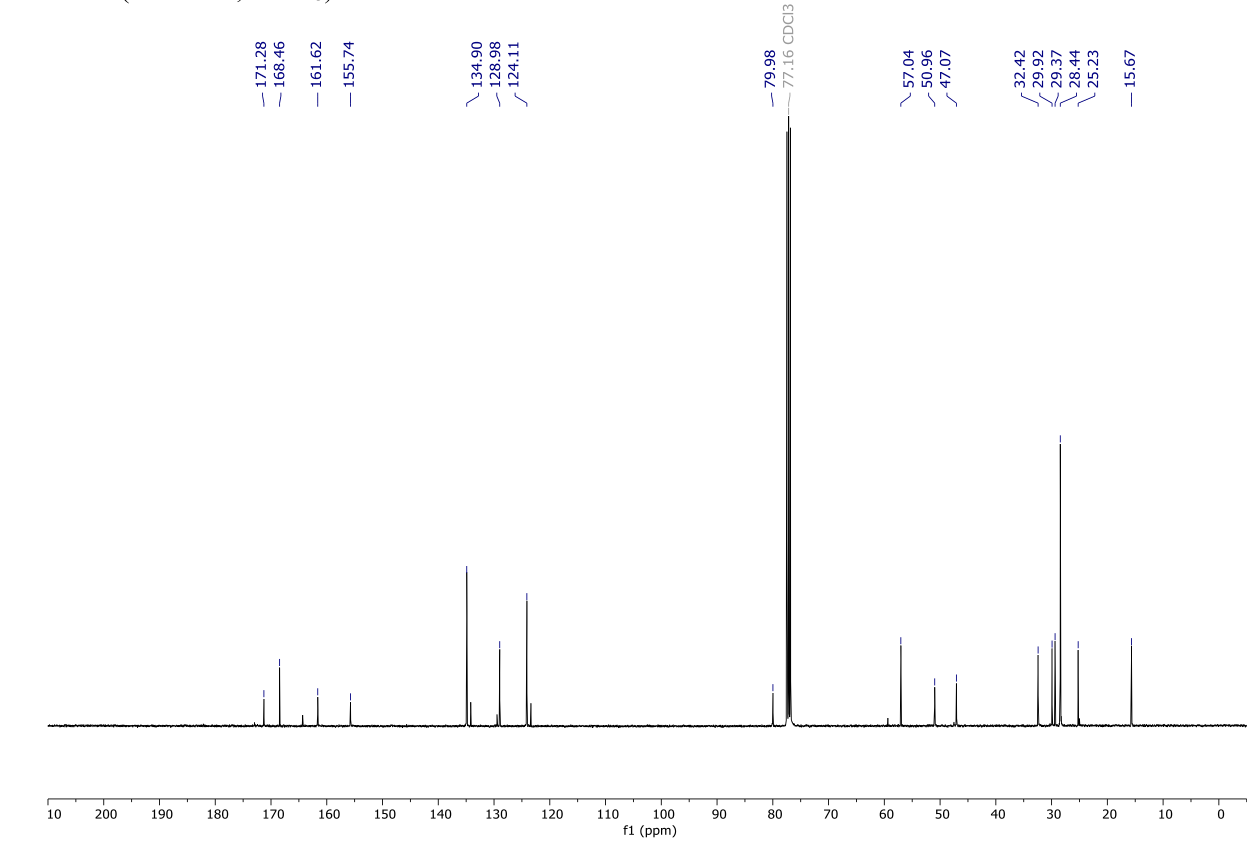

^{1}H NMR (400 MHz, $CDCl_3$) of **1ab**

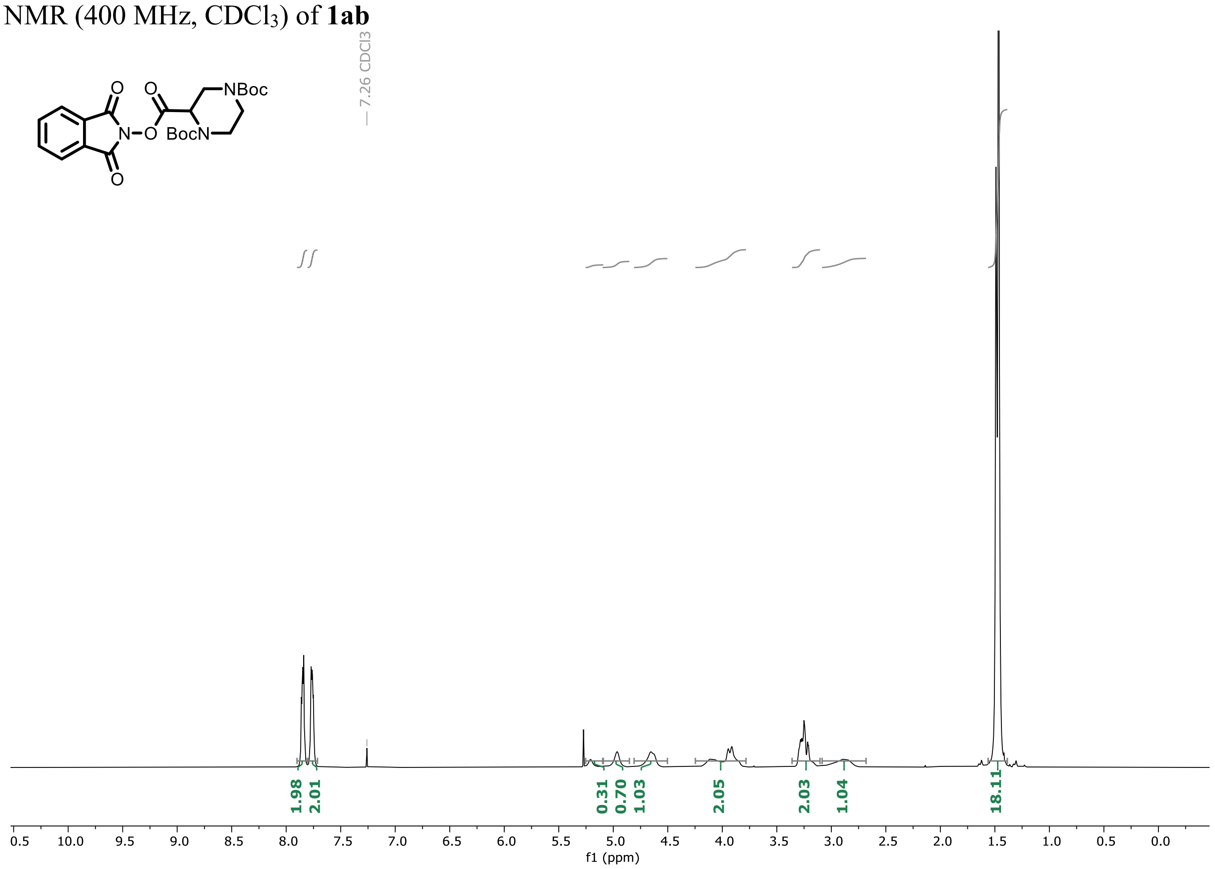

^{13}C NMR (101 MHz, $CDCl_3$) of **1ab**

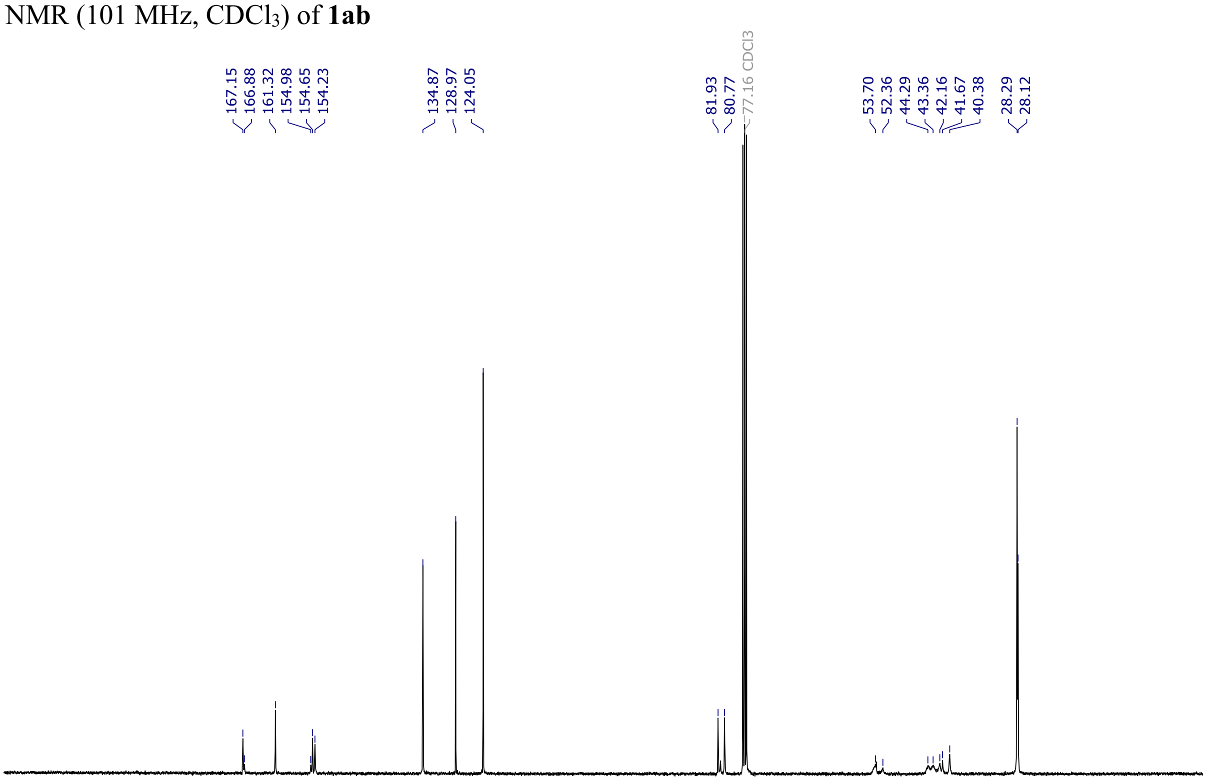

^{1}H NMR (400 MHz, $CDCl_3$) of **1ac**

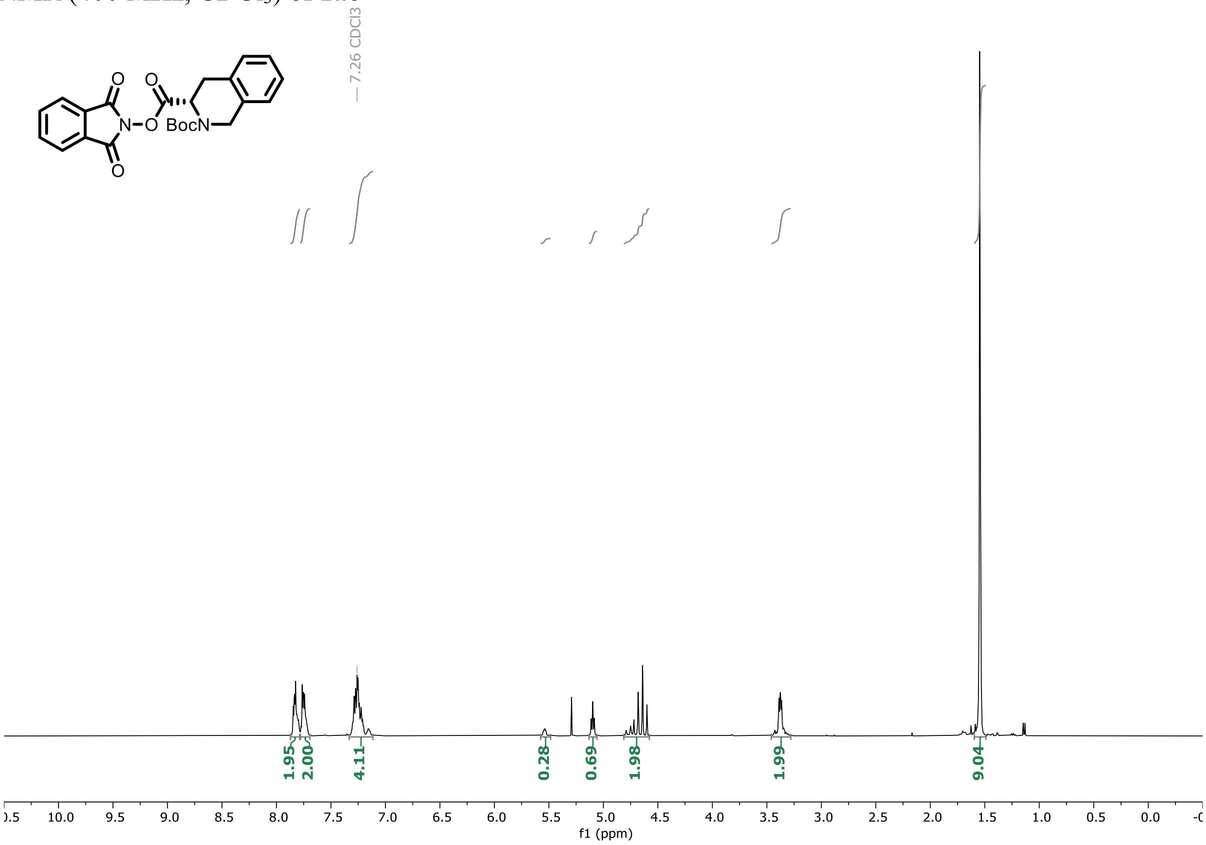

^{13}C NMR (101 MHz, $CDCl_3$) of **1ac**

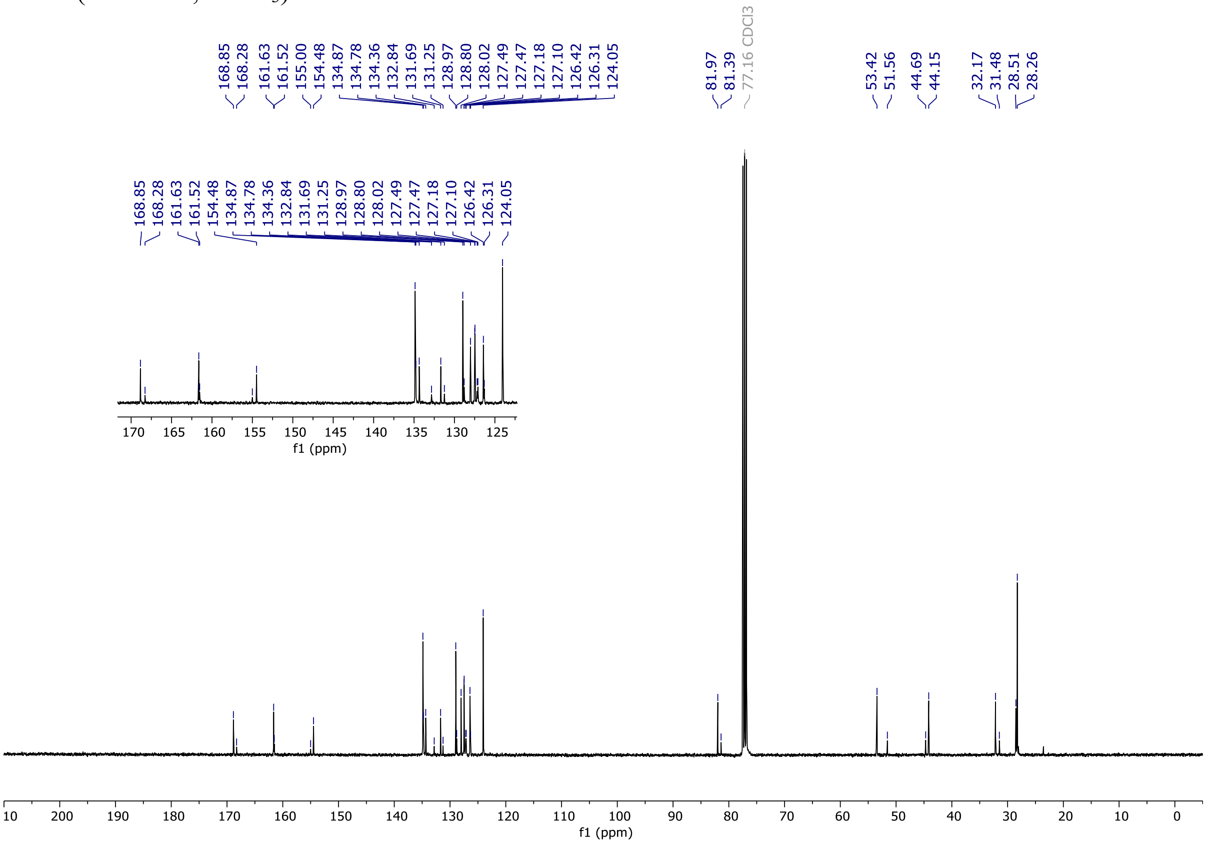

^{1}H NMR (400 MHz, $CDCl_3$) of **1ad**

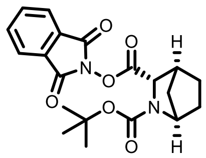

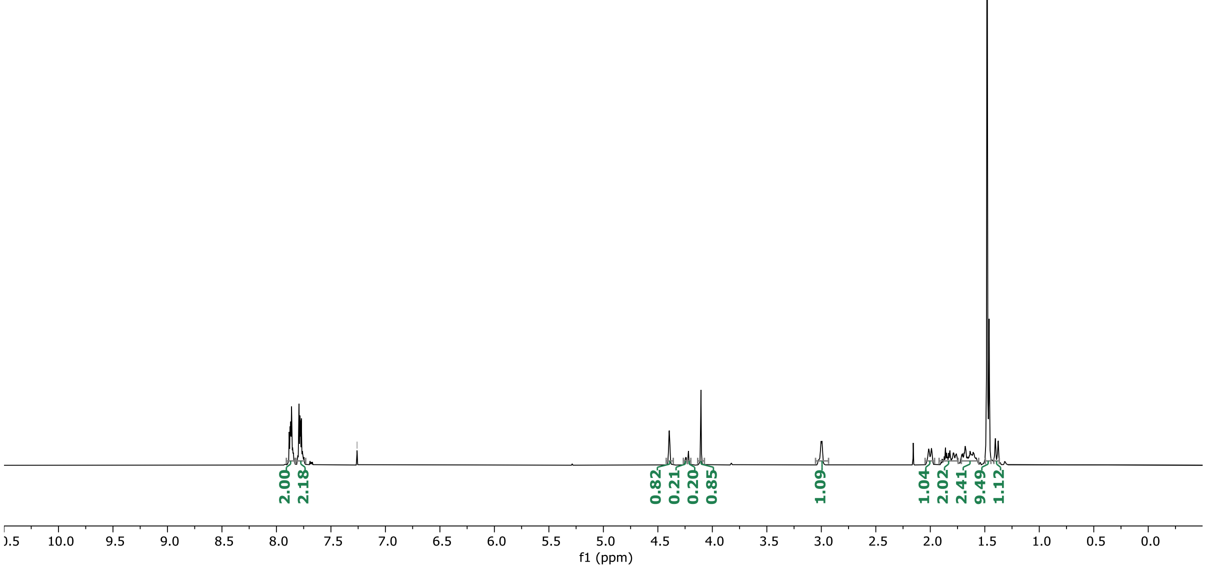

^{13}C NMR (101 MHz, $CDCl_3$) of **1ad**

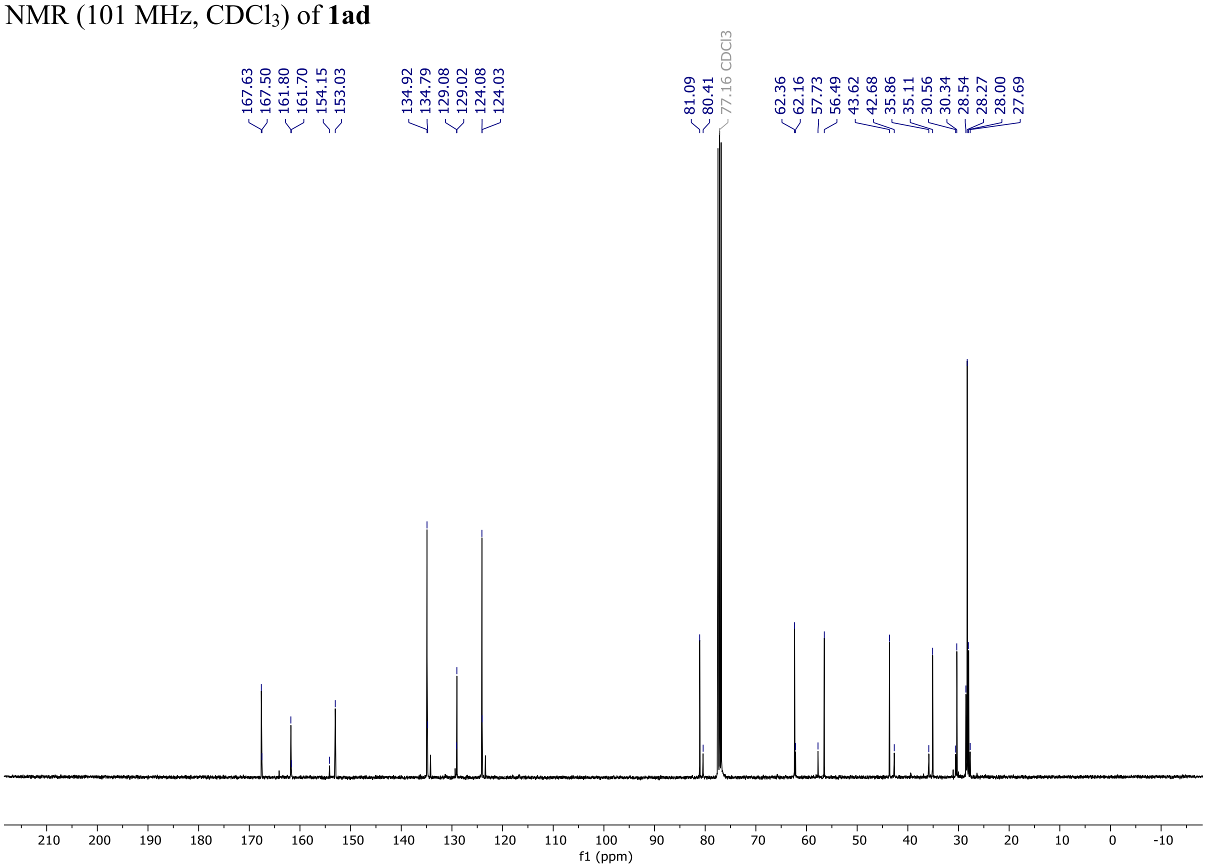

^{1}H NMR (400 MHz, $CDCl_3$) of **1ae**

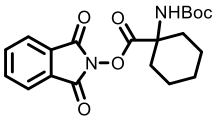

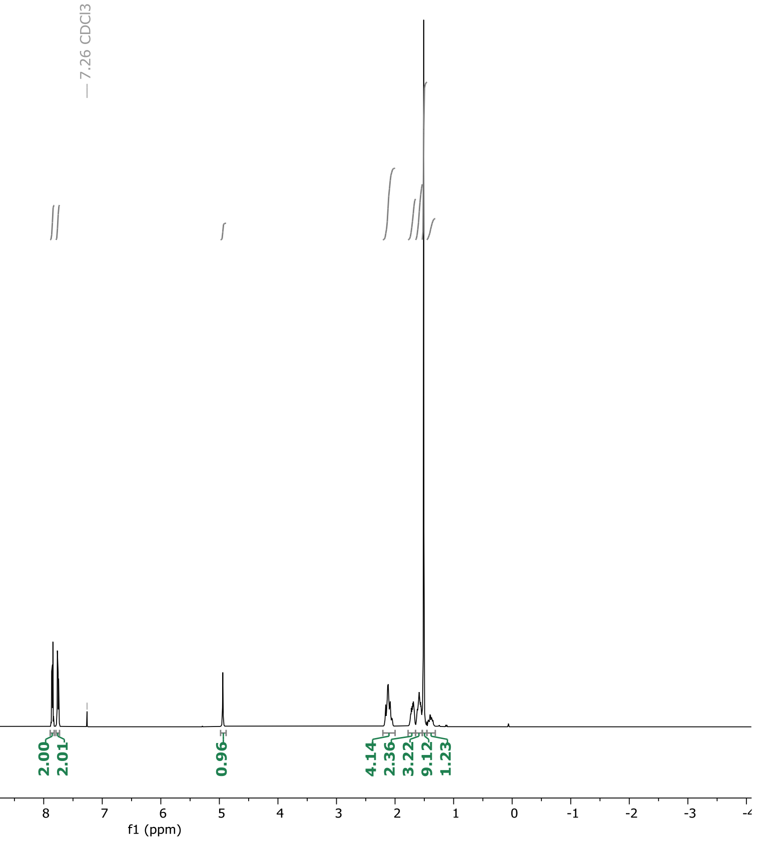

^{13}C NMR (101 MHz, $CDCl_3$) of **1ae**

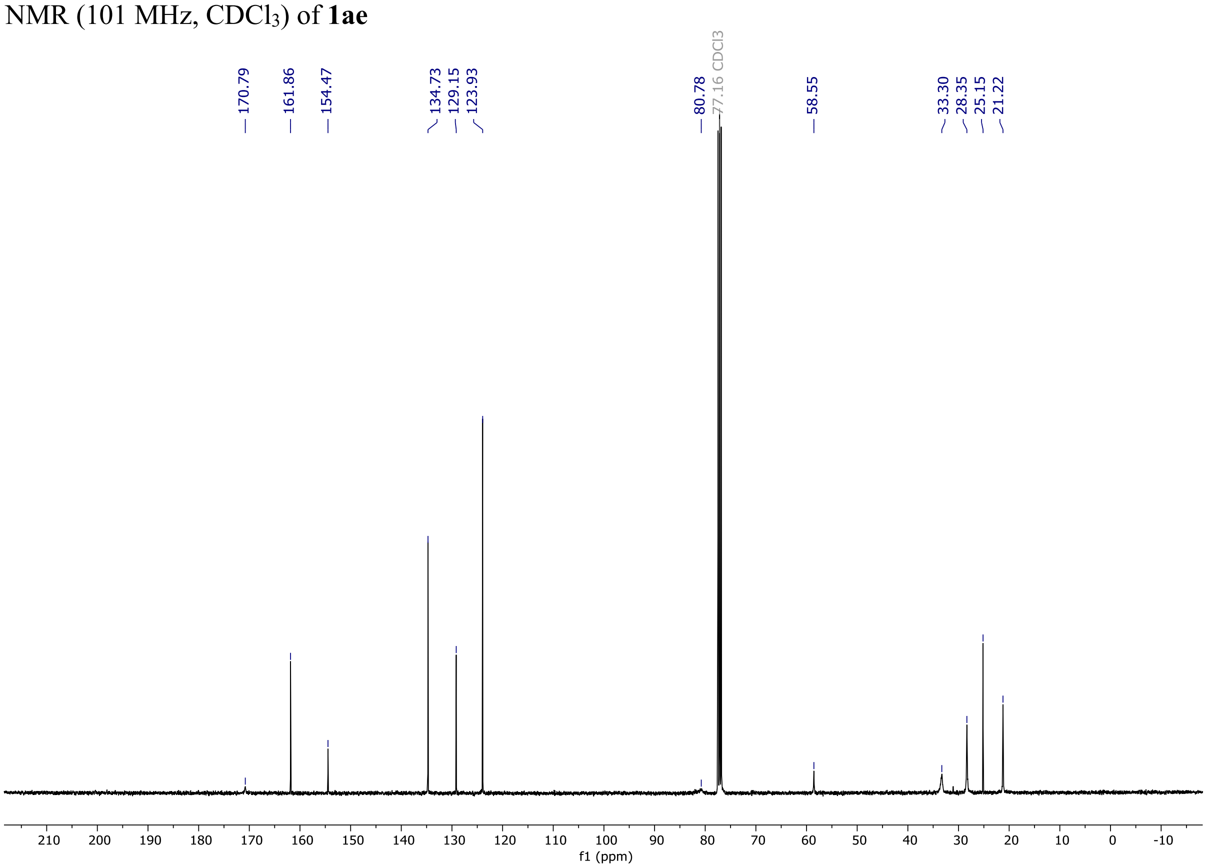

^{1}H NMR (500 MHz, $CDCl_3$) of **1af**

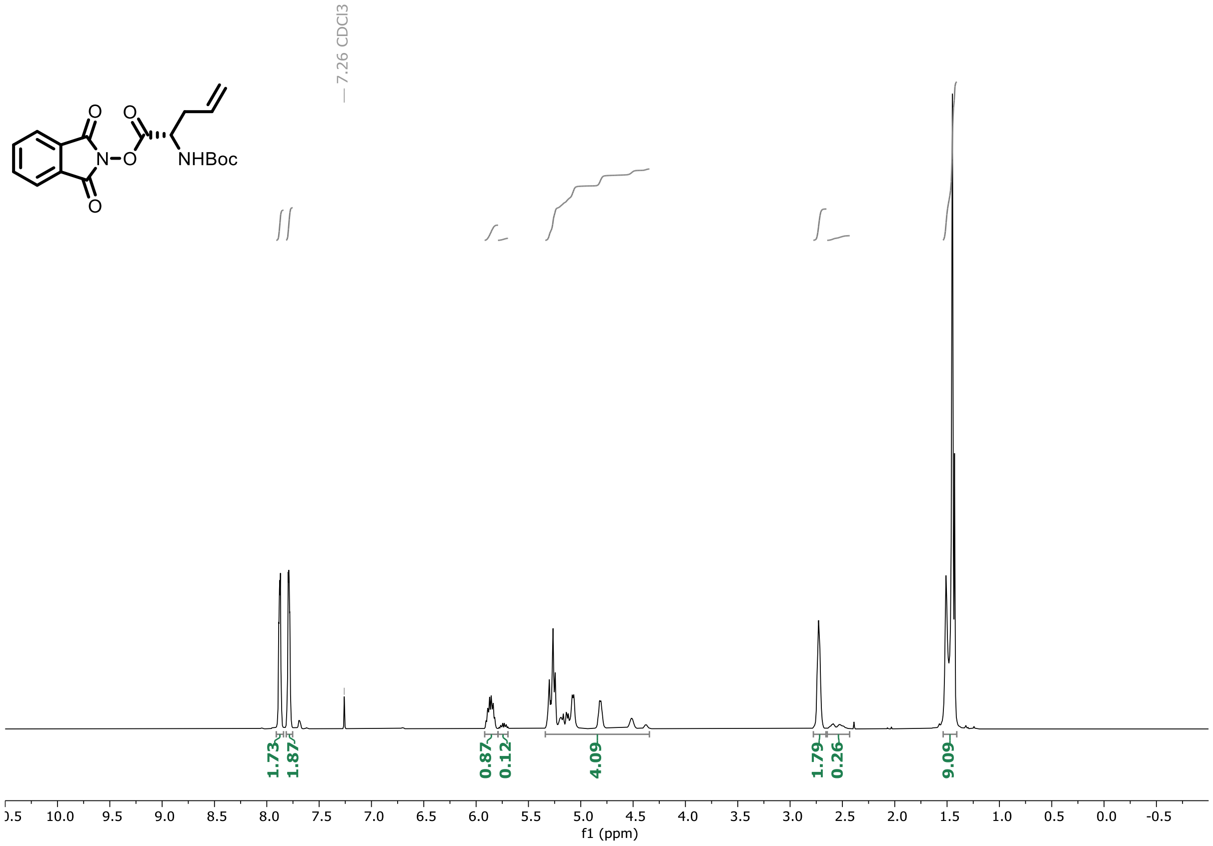

^{13}C NMR (126 MHz, $CDCl_3$)

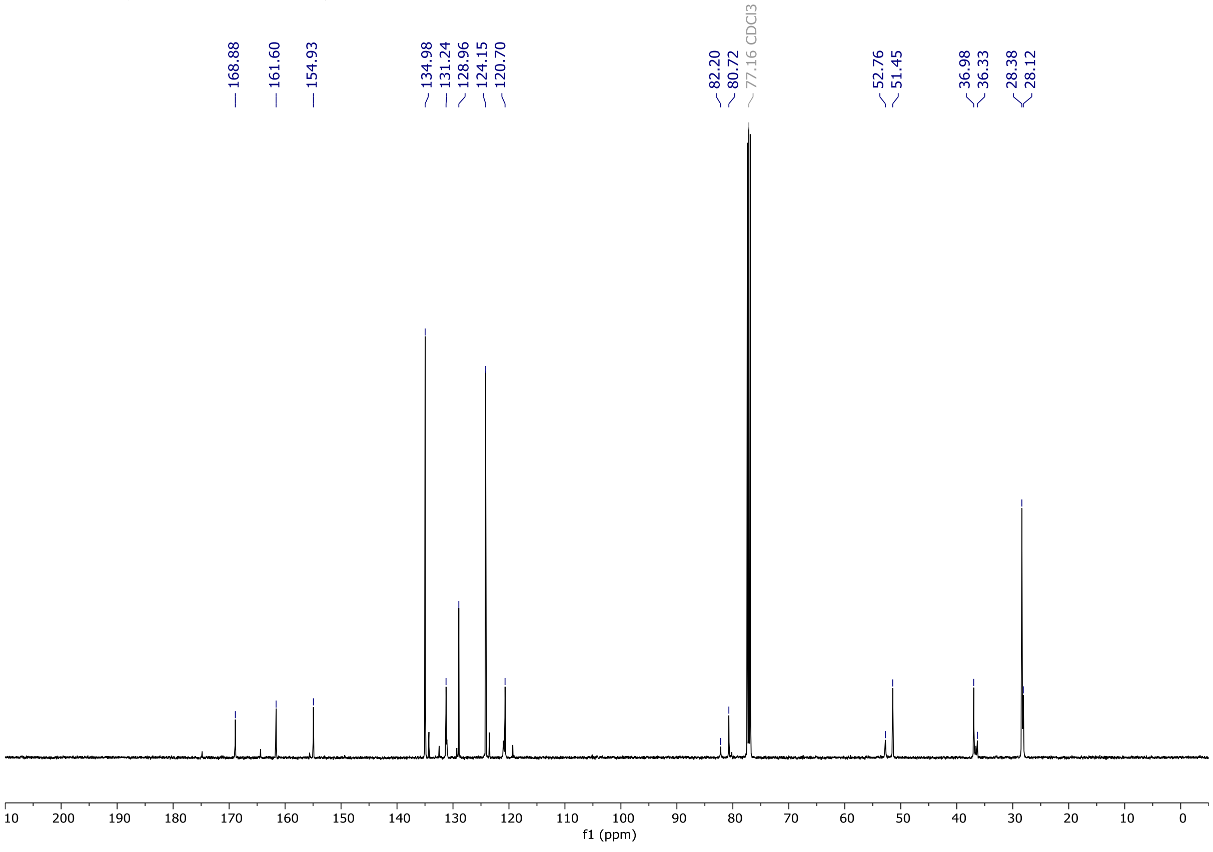

15. NMR spectra of products 3-26 (C1 homologation)

^{1}H NMR (400 MHz,CDCl$_3$) of **3**

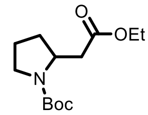

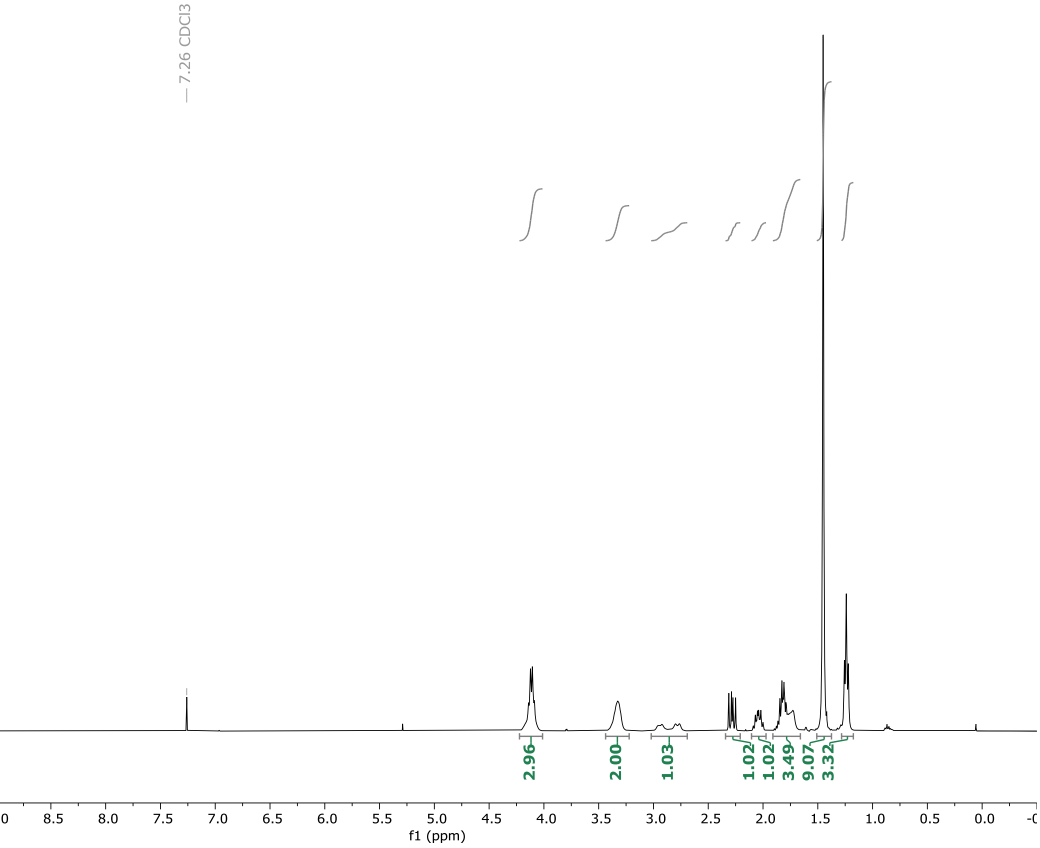

^{13}C NMR (101 MHz, CDCl$_3$) of **3**

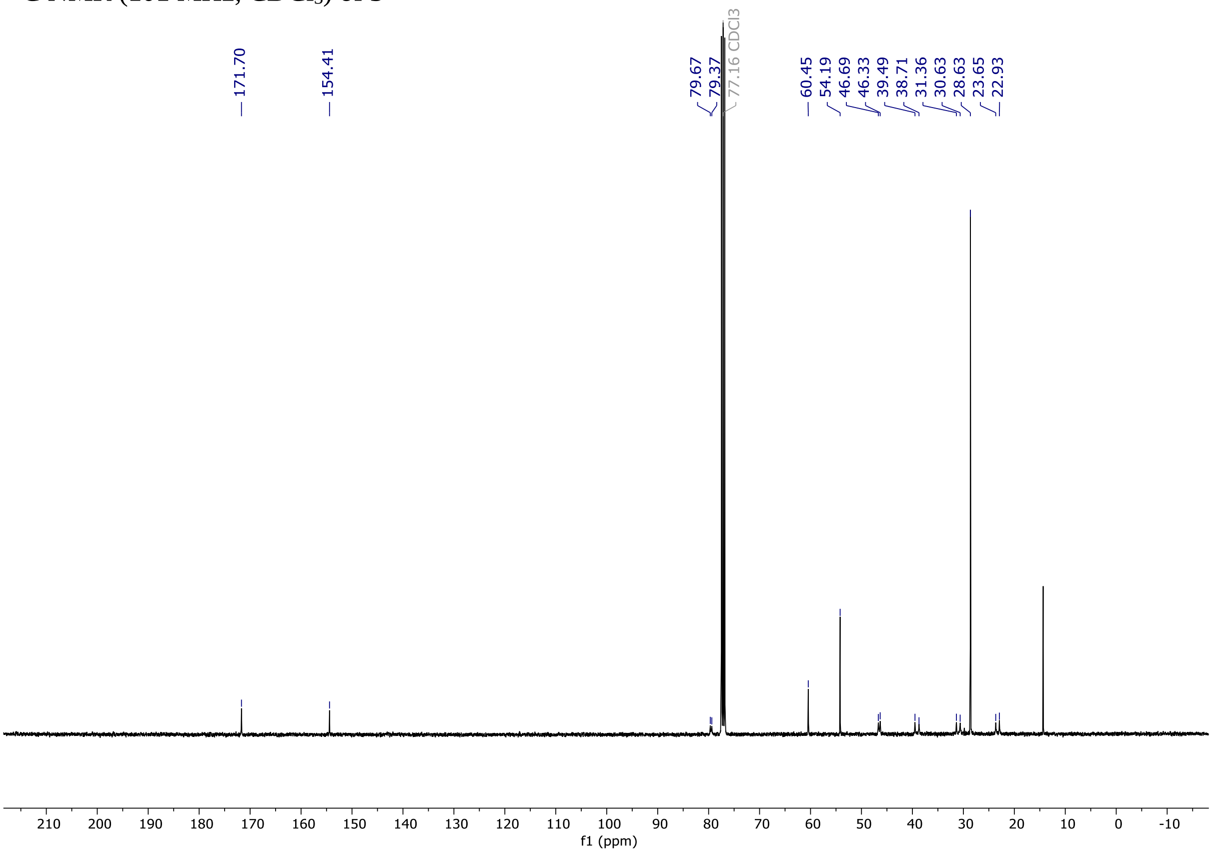

^{1}H NMR (500 MHz,$CDCl_3$) of **4**

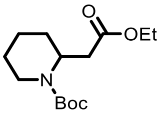

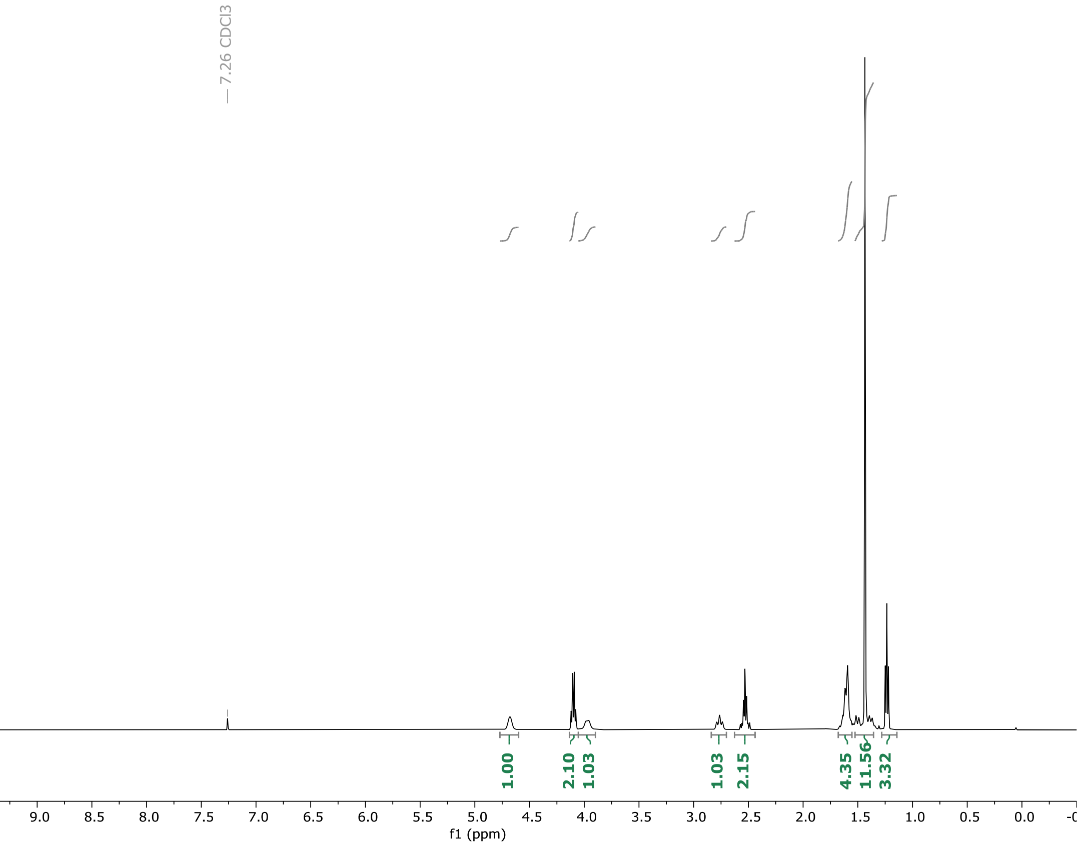

^{13}C NMR (126 MHz, $CDCl_3$) of **4**

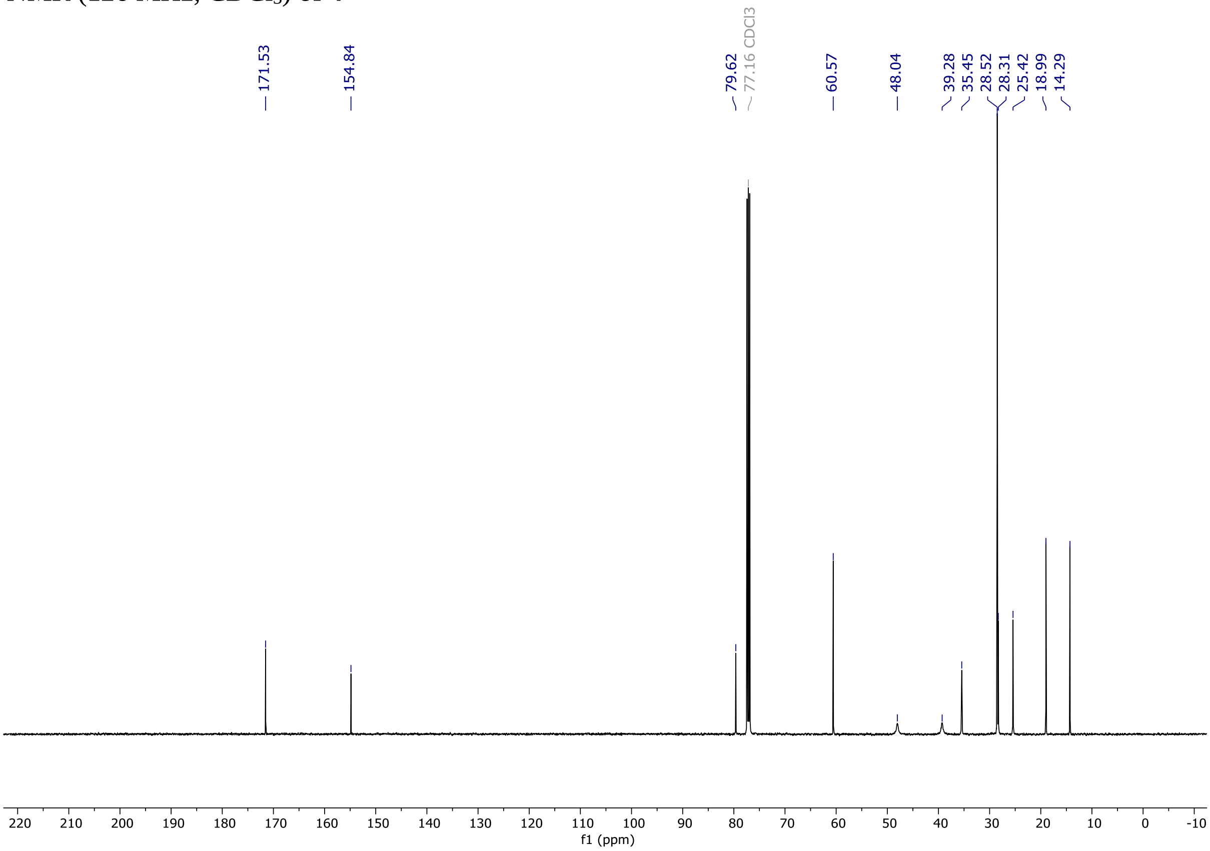

^{1}H NMR (500 MHz,$CDCl_3$) of **5**

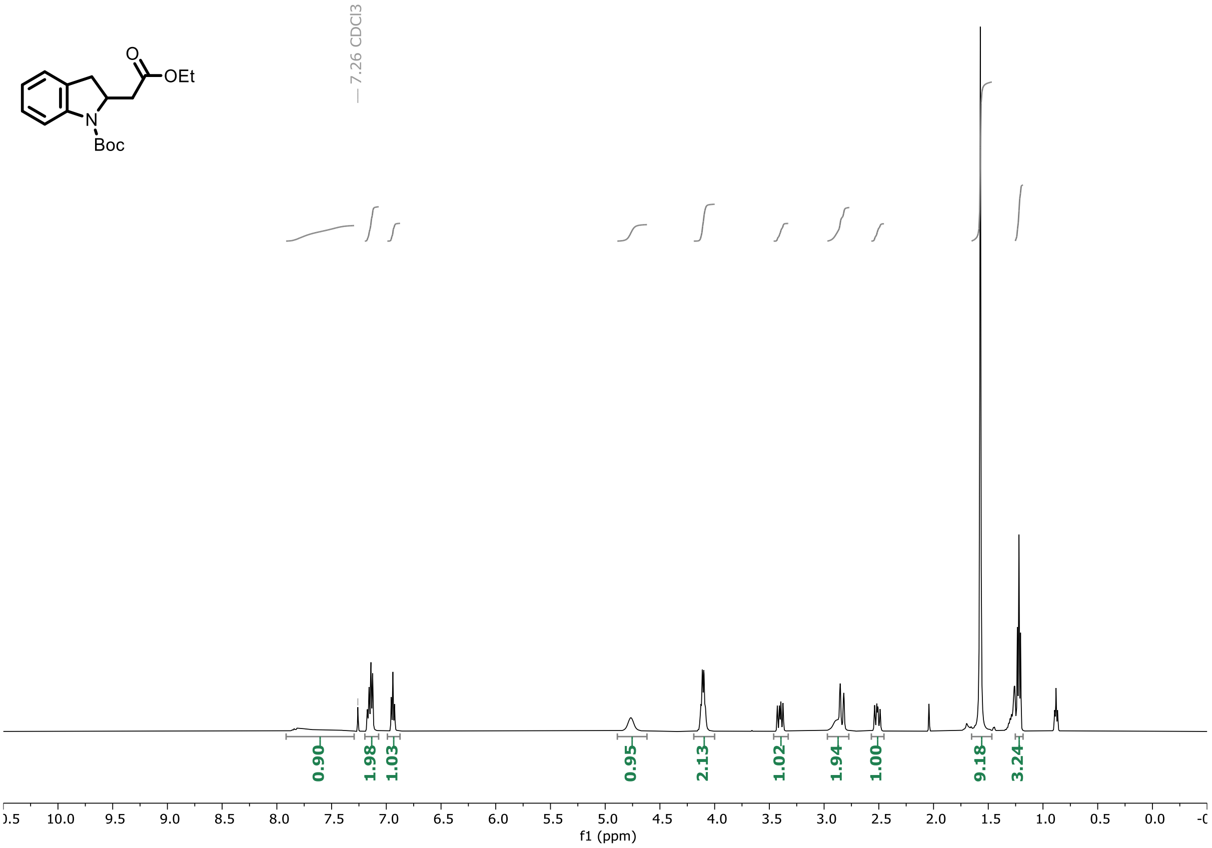

^{13}C NMR (126 MHz, $CDCl_3$) of **5**

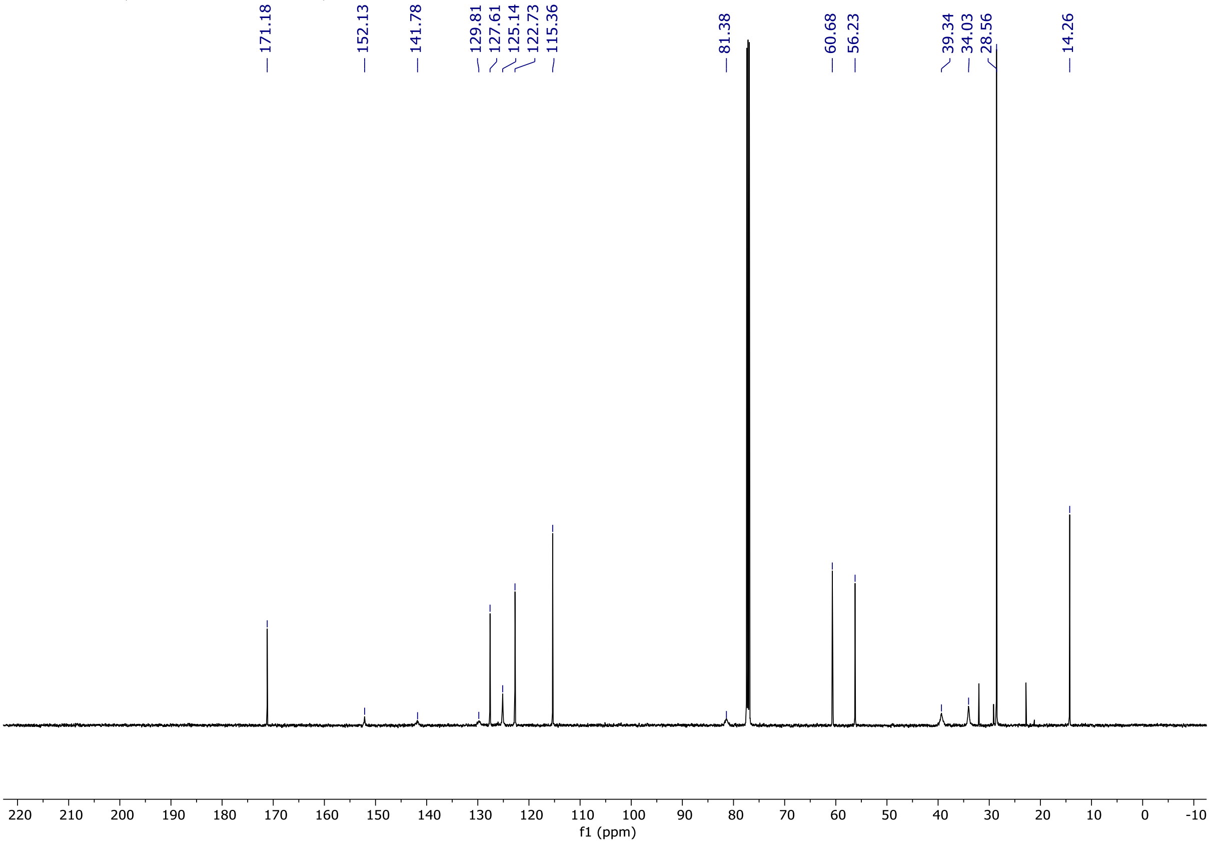

^{1}H NMR (400 MHz, $CDCl_3$) of **6**

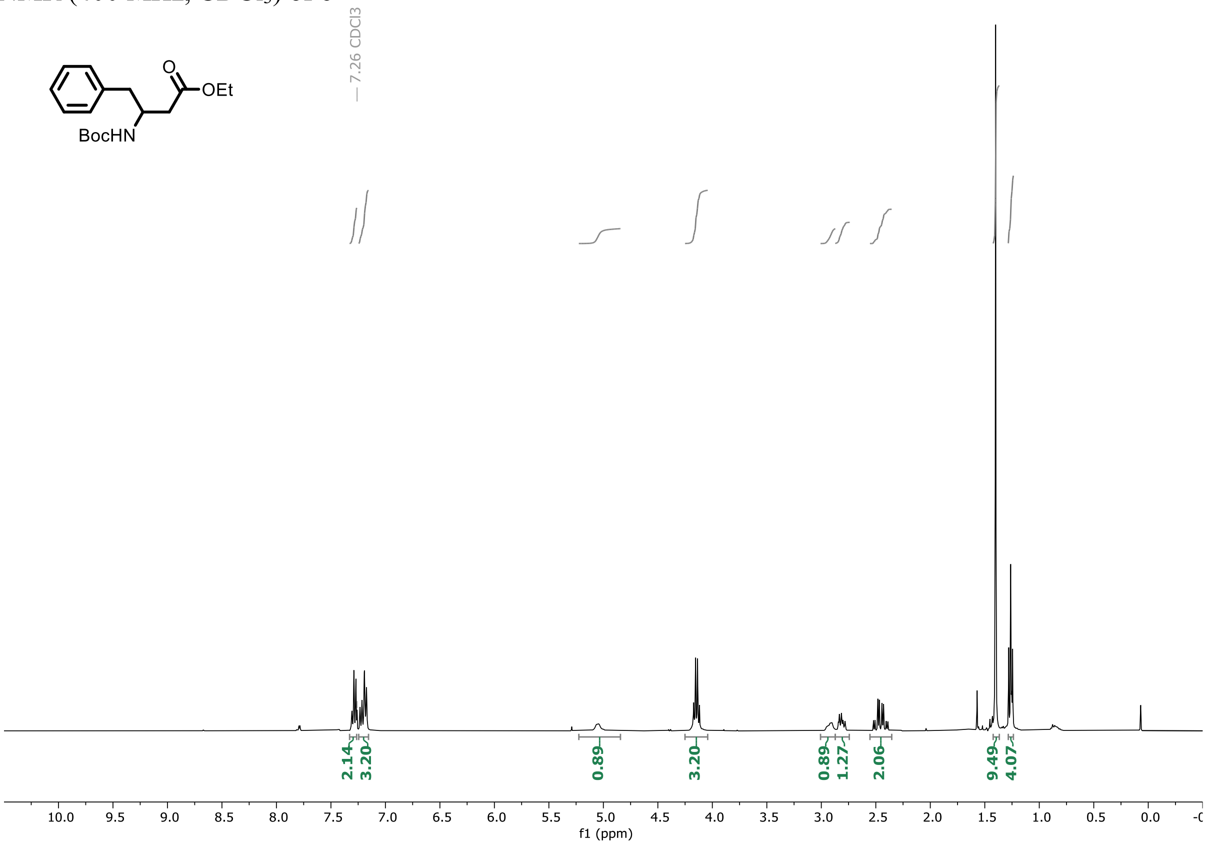

^{13}C NMR (101 MHz, $CDCl_3$) of **6**

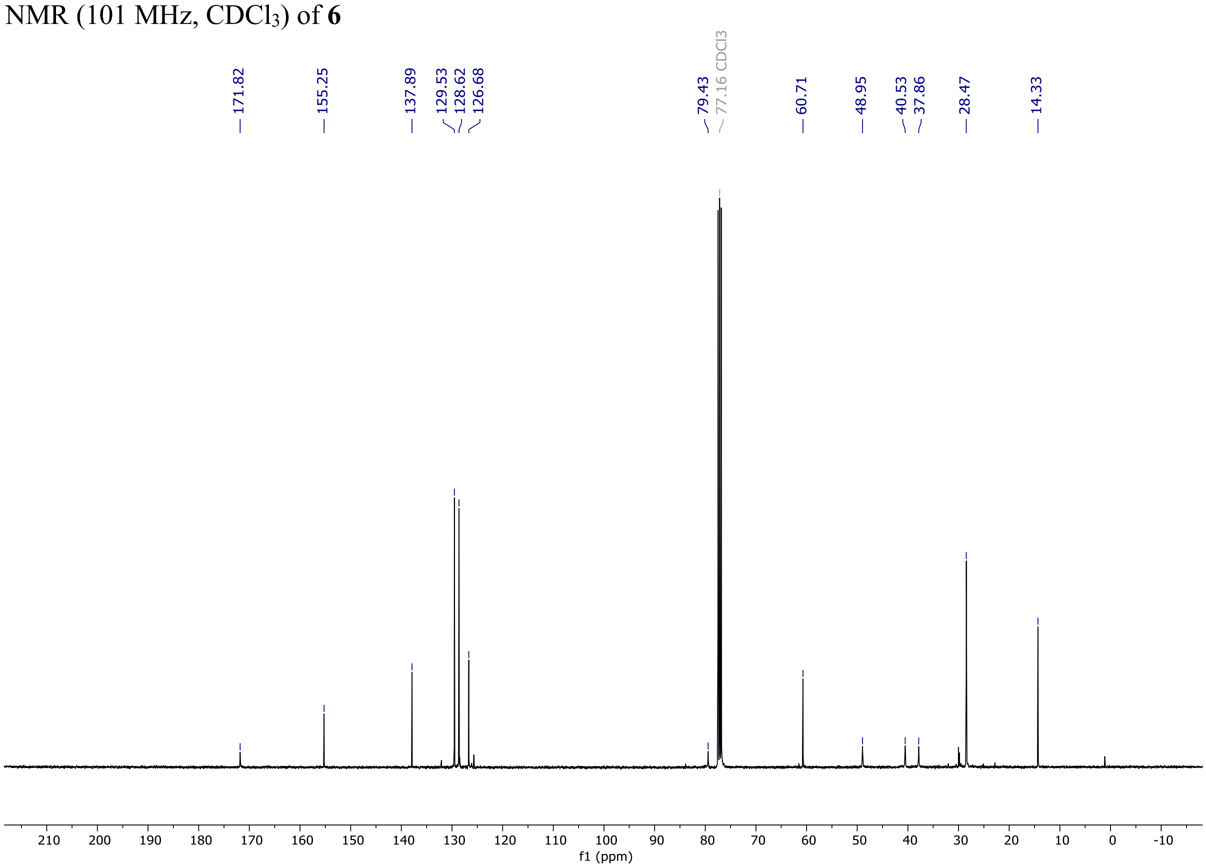

^{1}H NMR (400 MHz, $CDCl_3$) of **7**

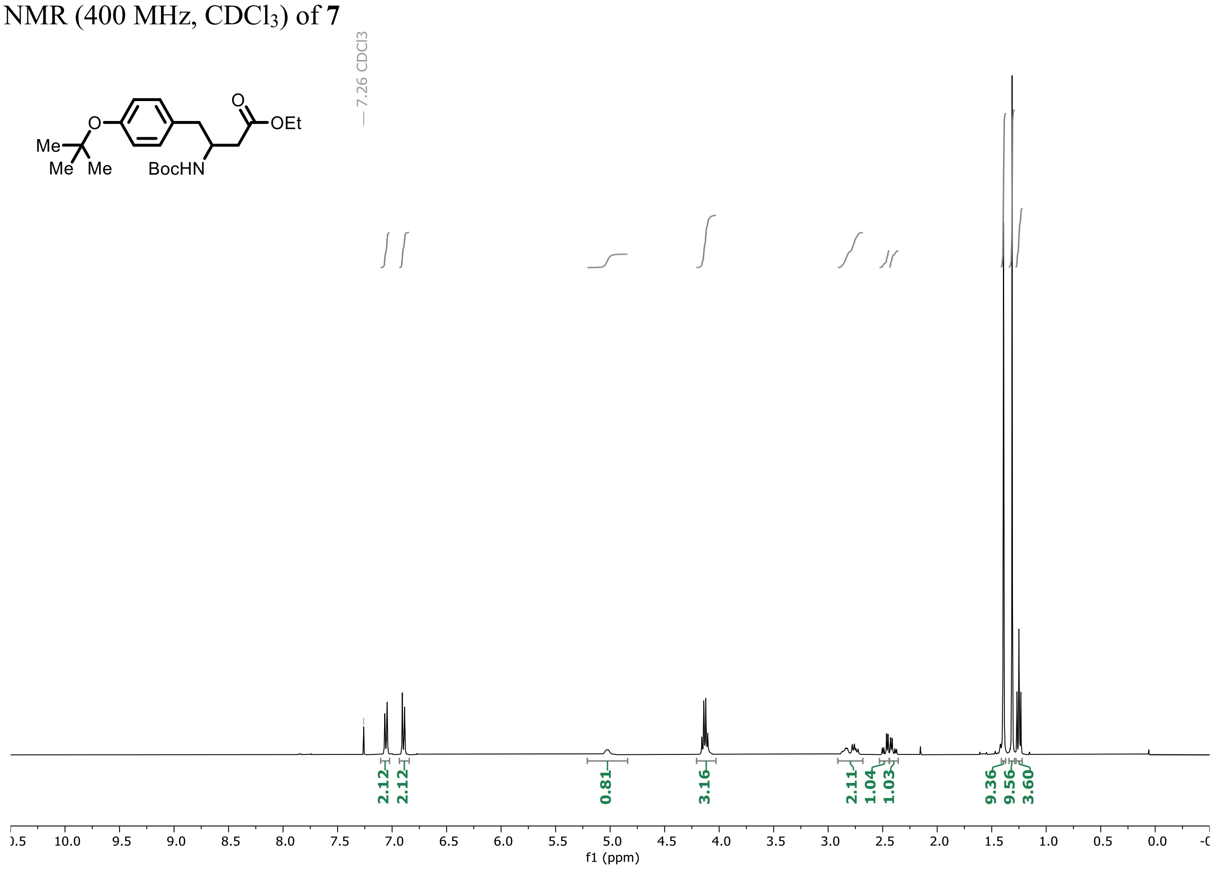

^{13}C NMR (101 MHz, $CDCl_3$) of **7**

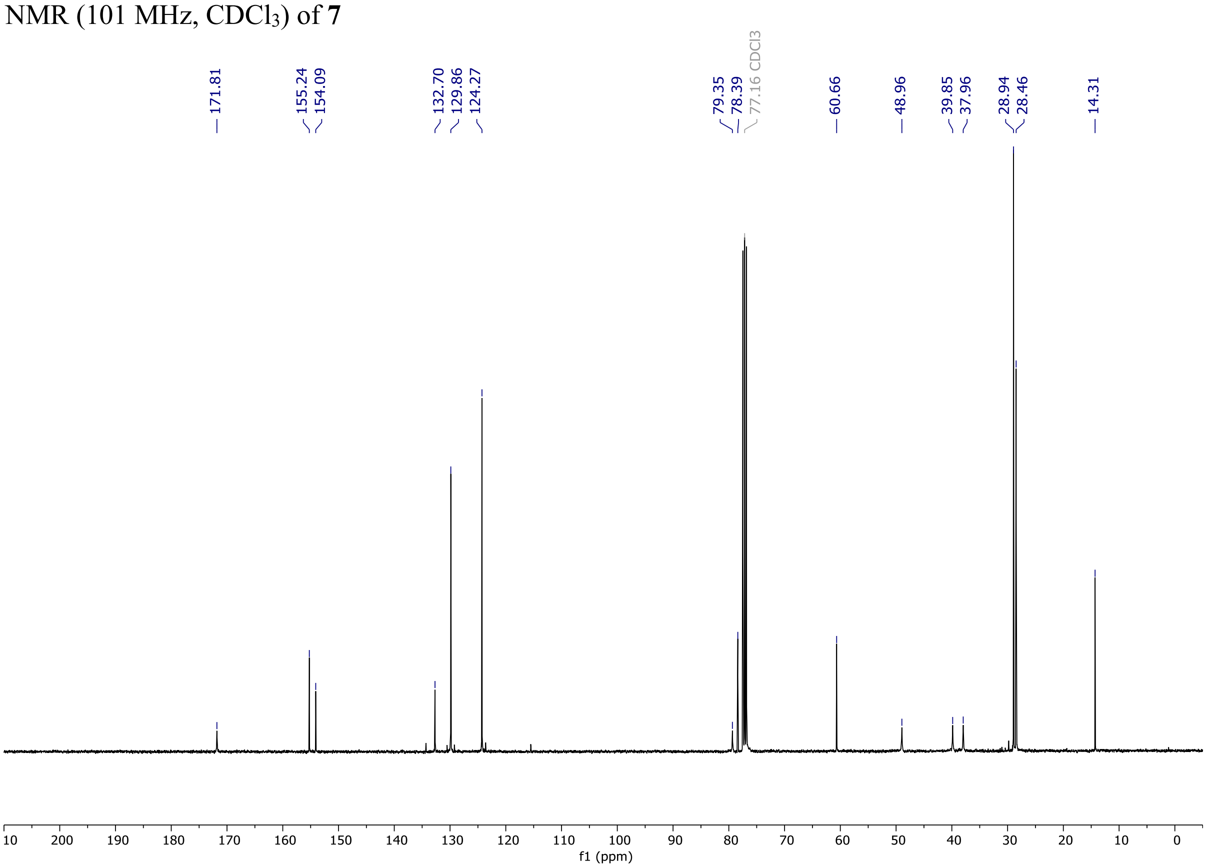

^{1}H NMR (400 MHz, $CDCl_3$) of **8**

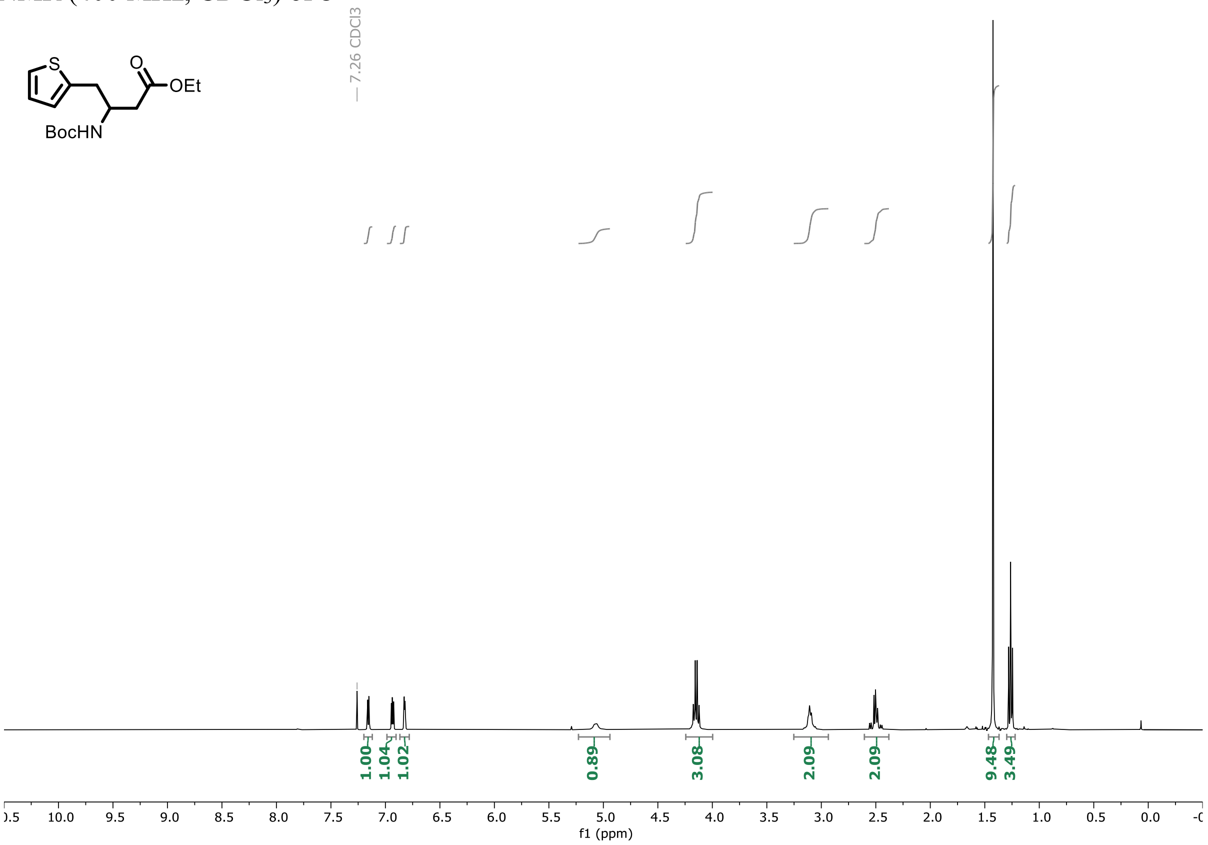

^{13}C NMR (101 MHz, $CDCl_3$) of **8**

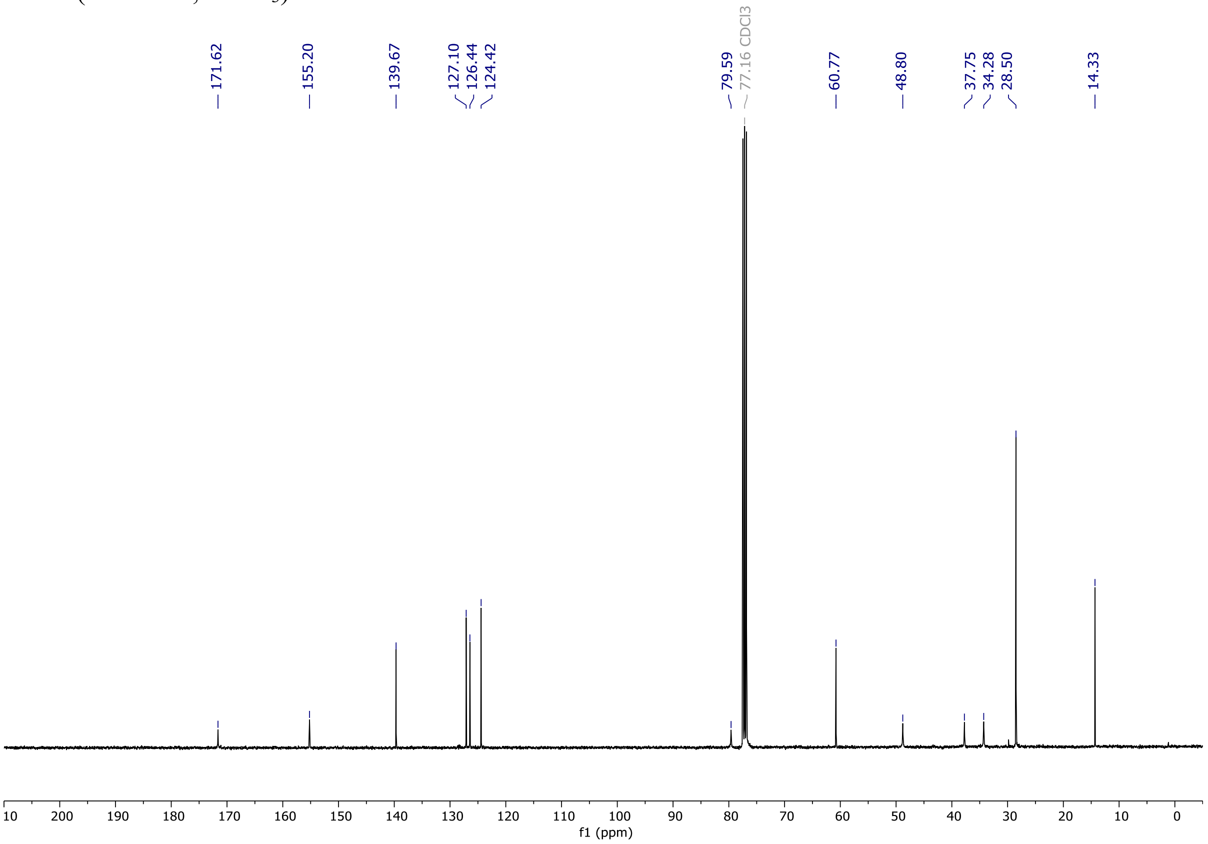

^{1}H NMR (400 MHz,$CDCl_3$) of **9**

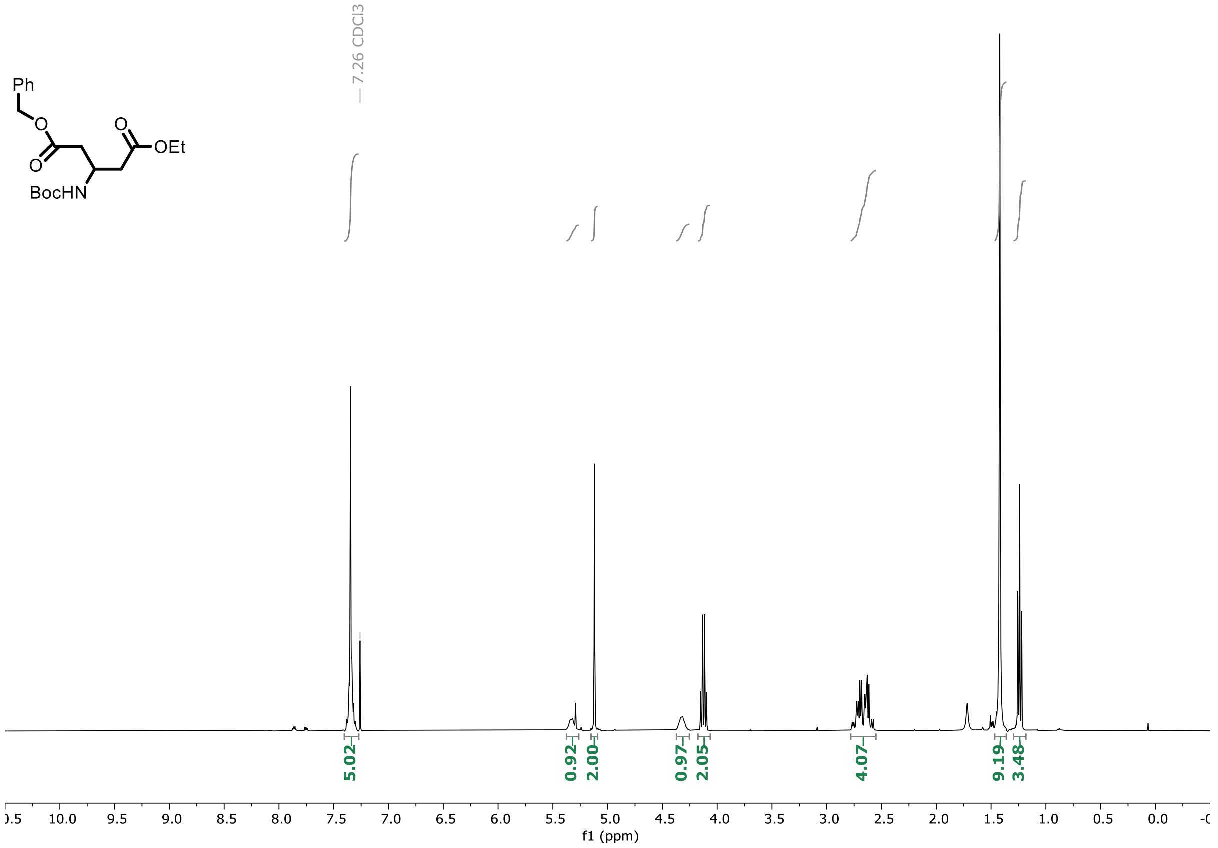

^{13}C NMR (101 MHz, $CDCl_3$) of **9**

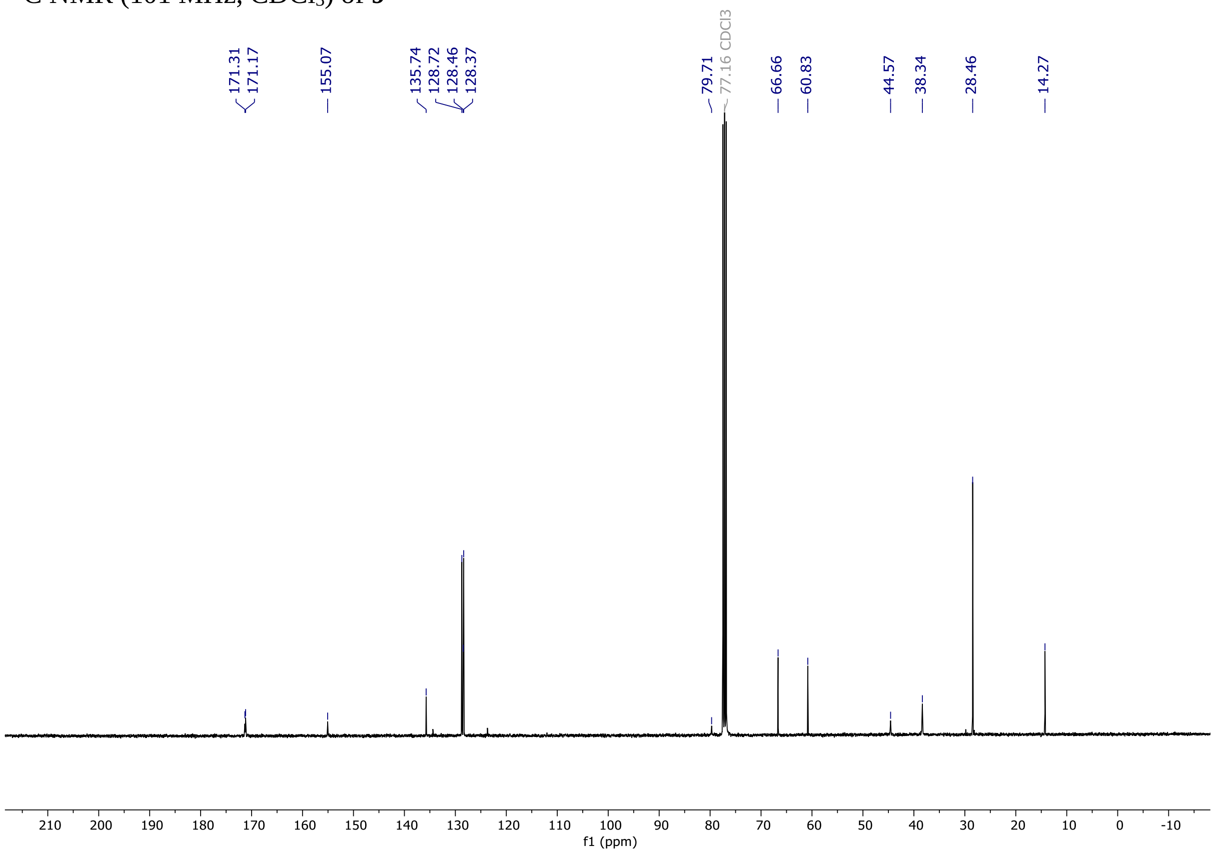

^{1}H NMR (400 MHz, $CDCl_3$) of **10**

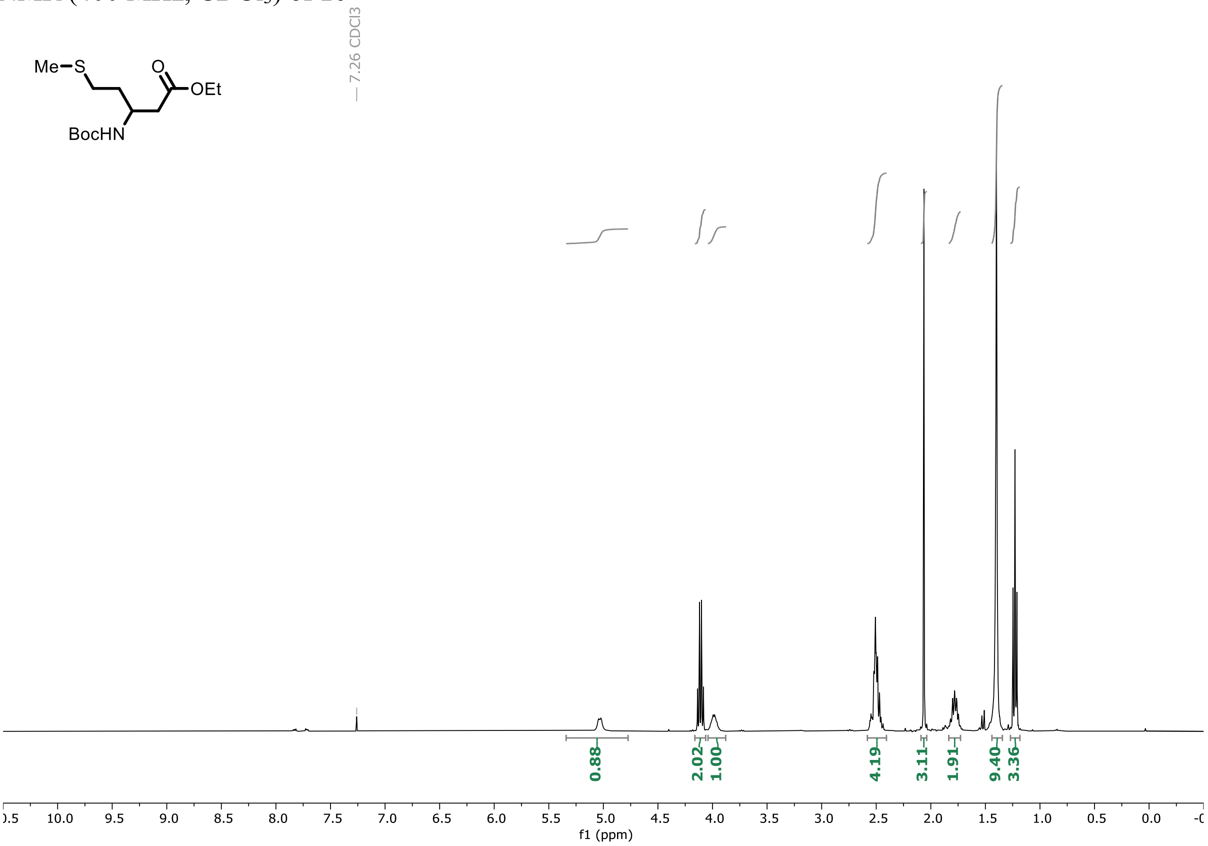

^{13}C NMR (101 MHz, $CDCl_3$) of **10**

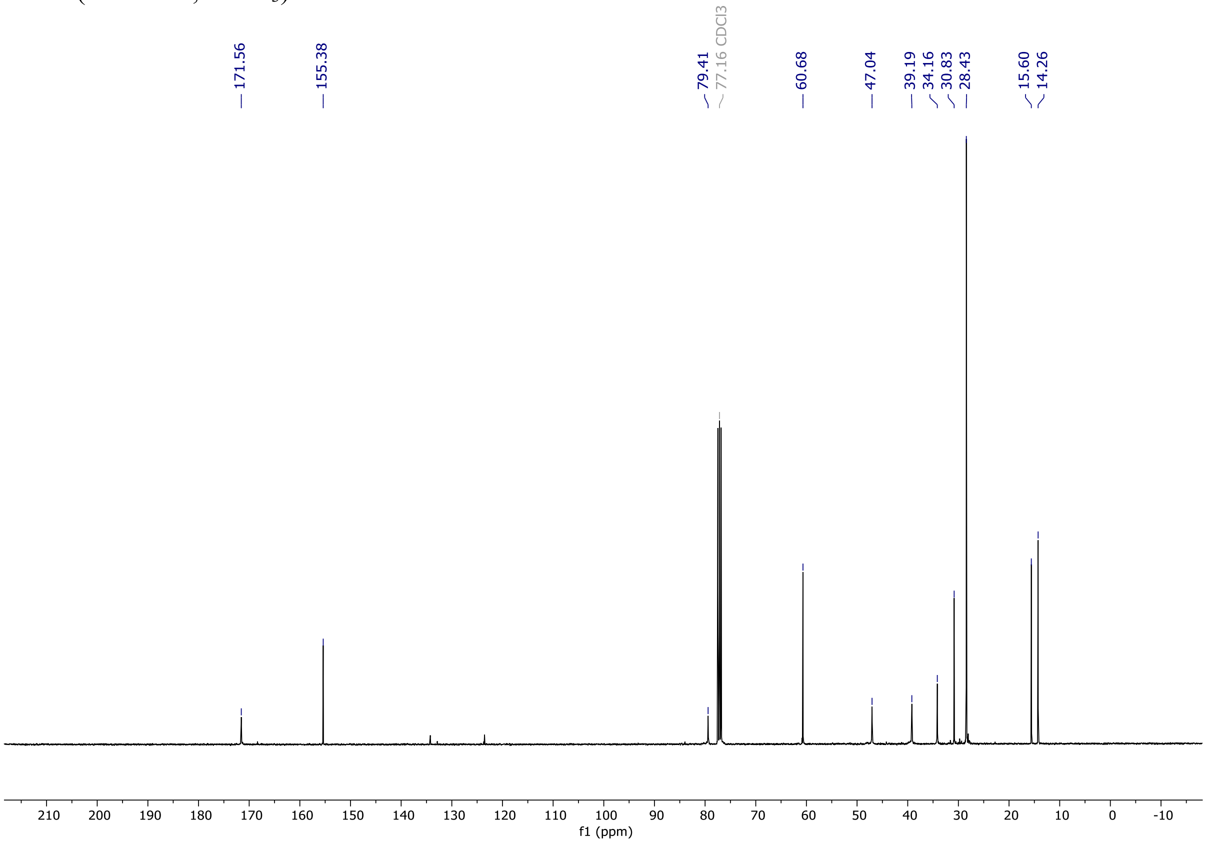

^1H NMR (400 MHz, $CDCl_3$) of **11**

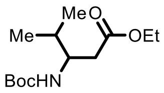

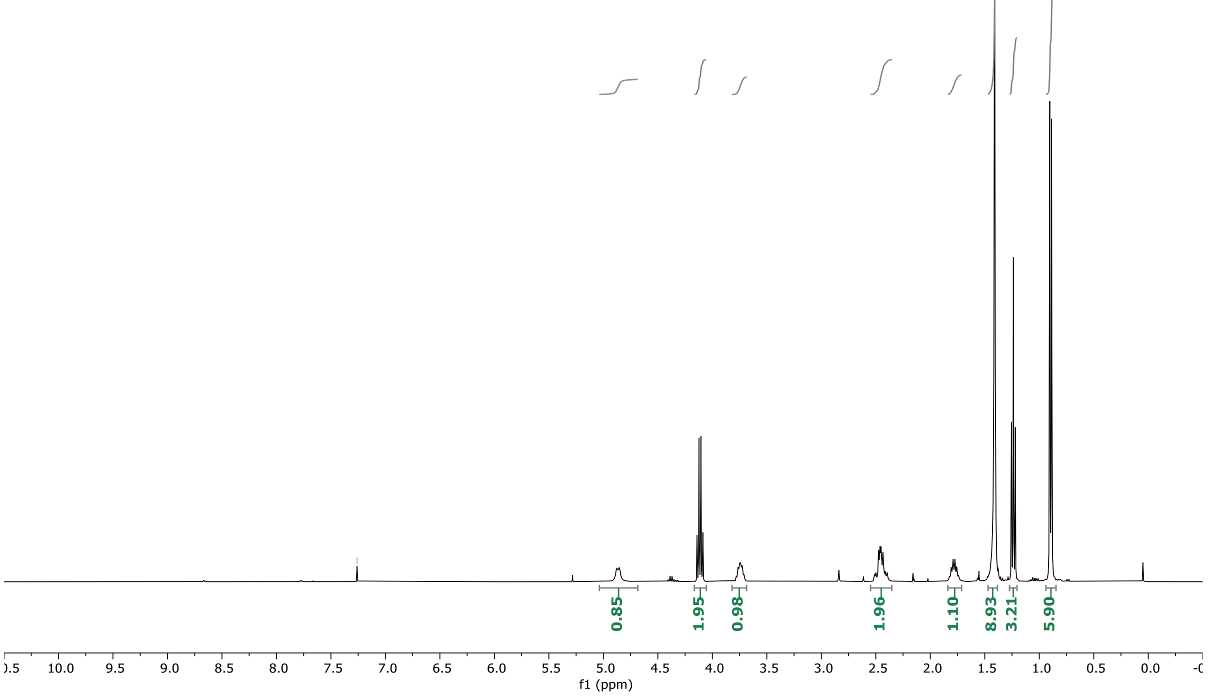

^{13}C NMR (101 MHz, $CDCl_3$) of **11**

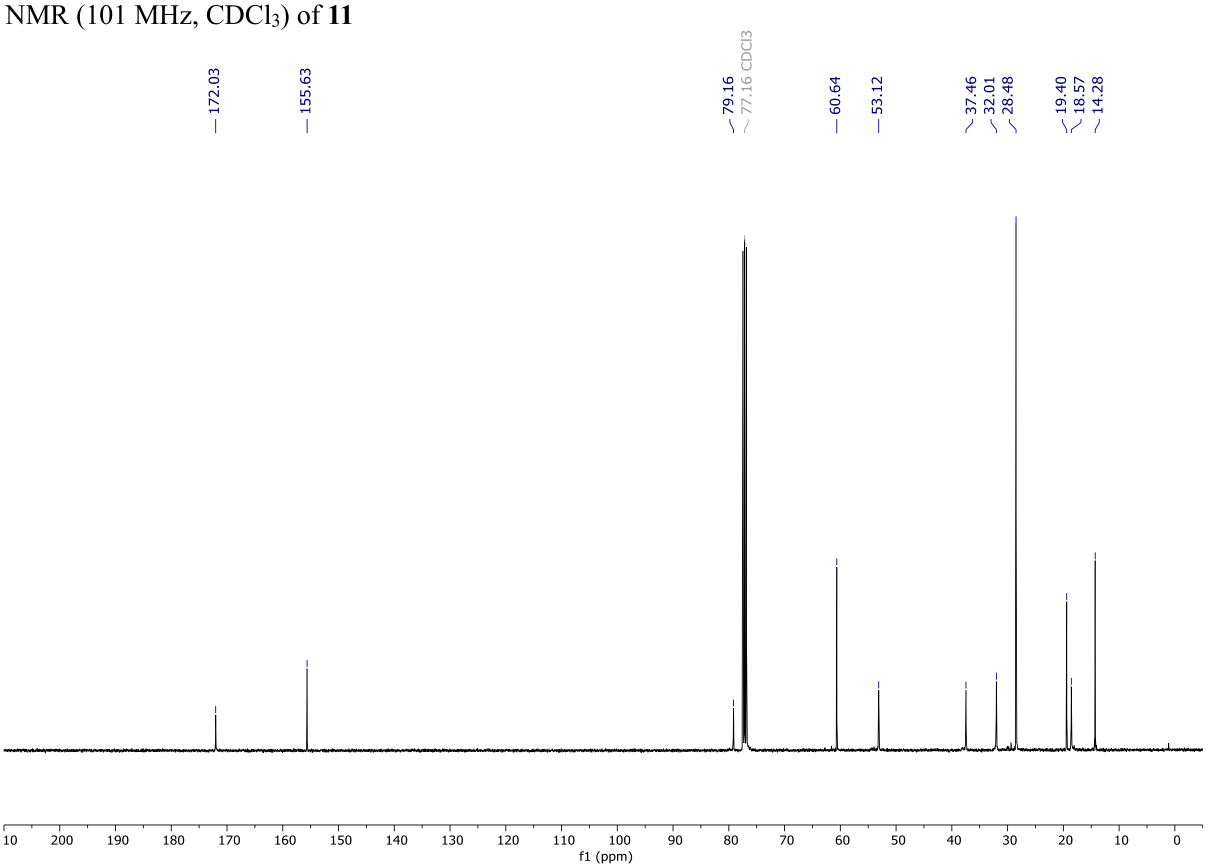

^{1}H NMR (400 MHz, $CDCl_3$) of **12**

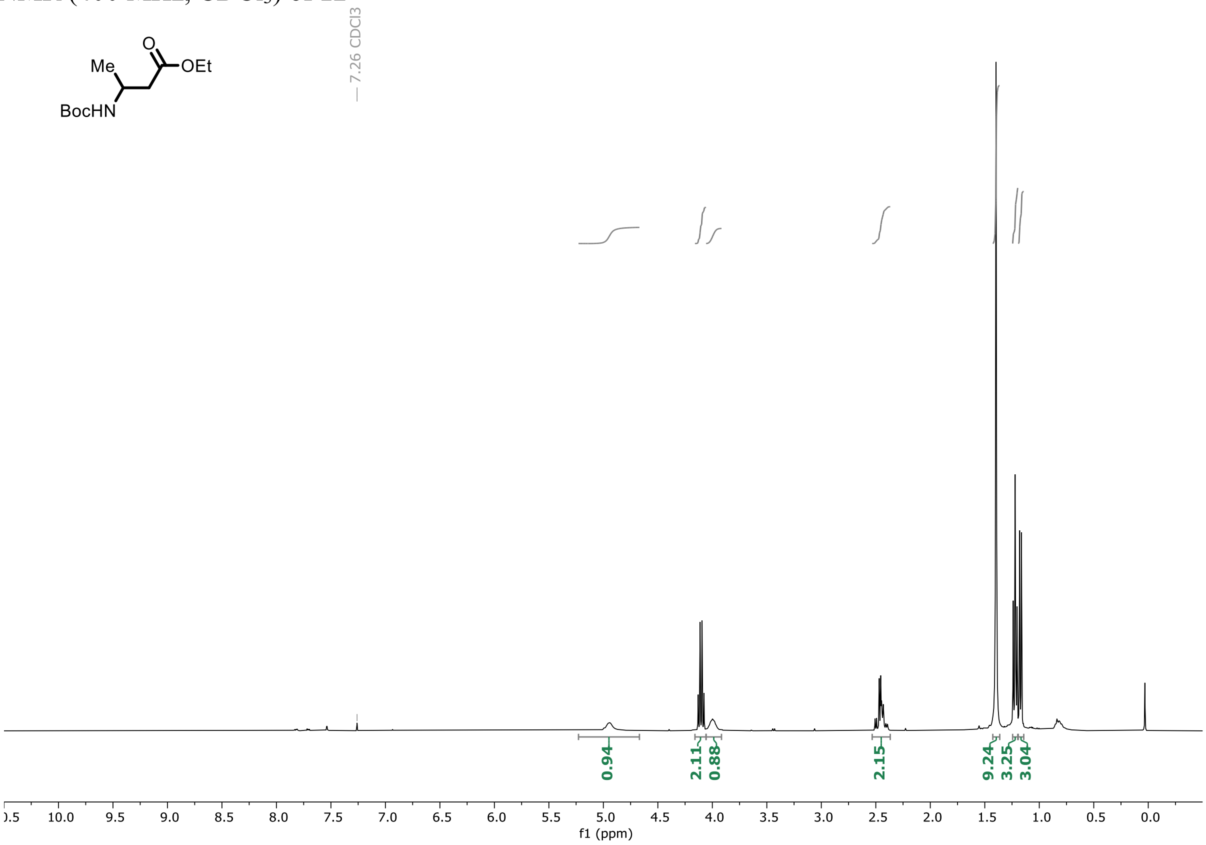

^{13}C NMR (101 MHz, $CDCl_3$) of **12**

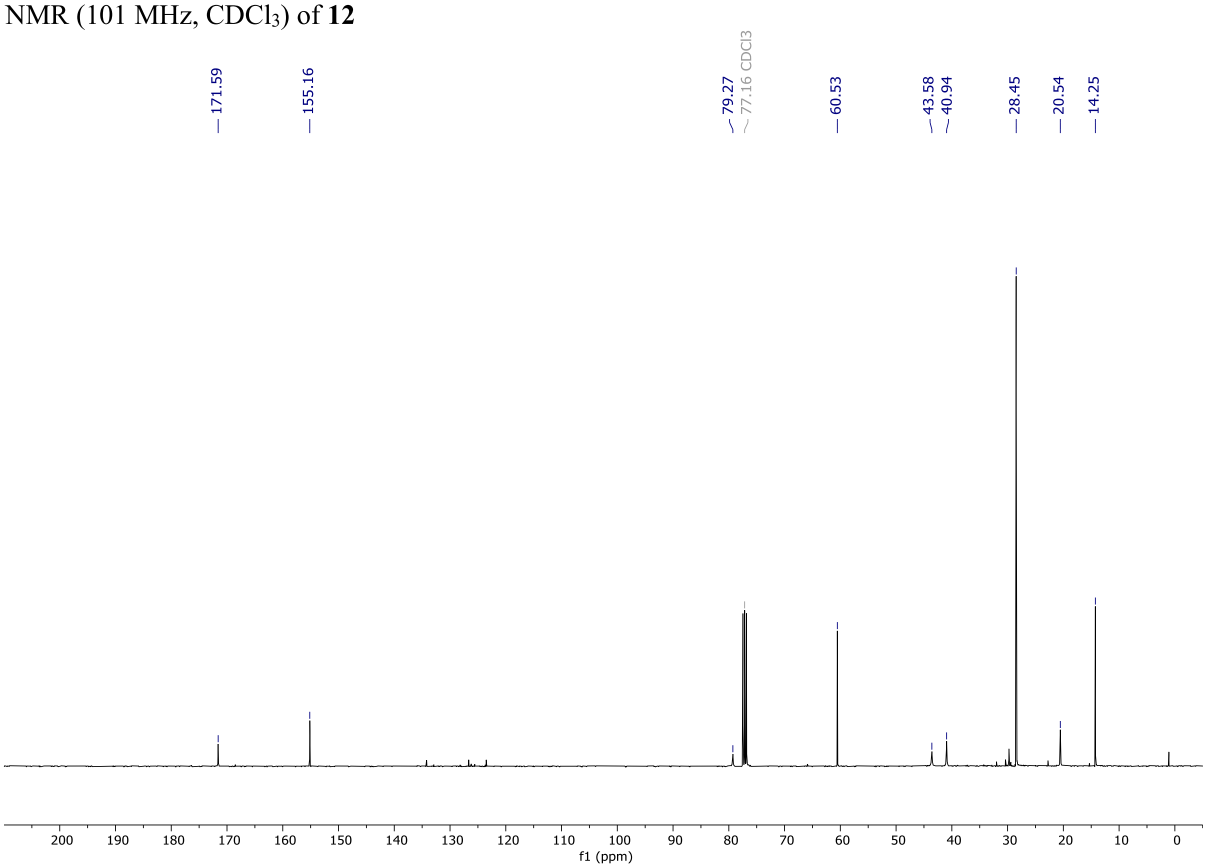

^{1}H NMR (400 MHz,$CDCl_3$) of **13**

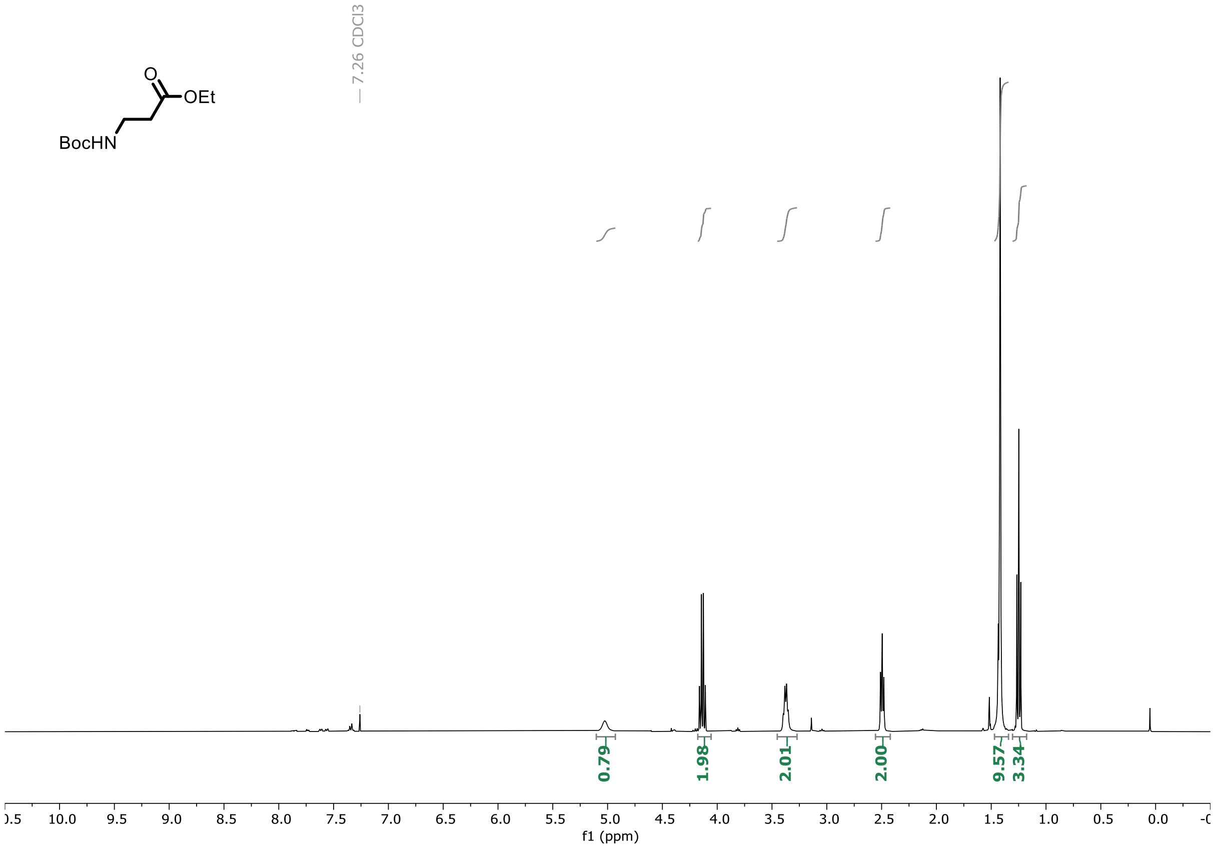

^{13}C NMR (101 MHz, $CDCl_3$) of **13**

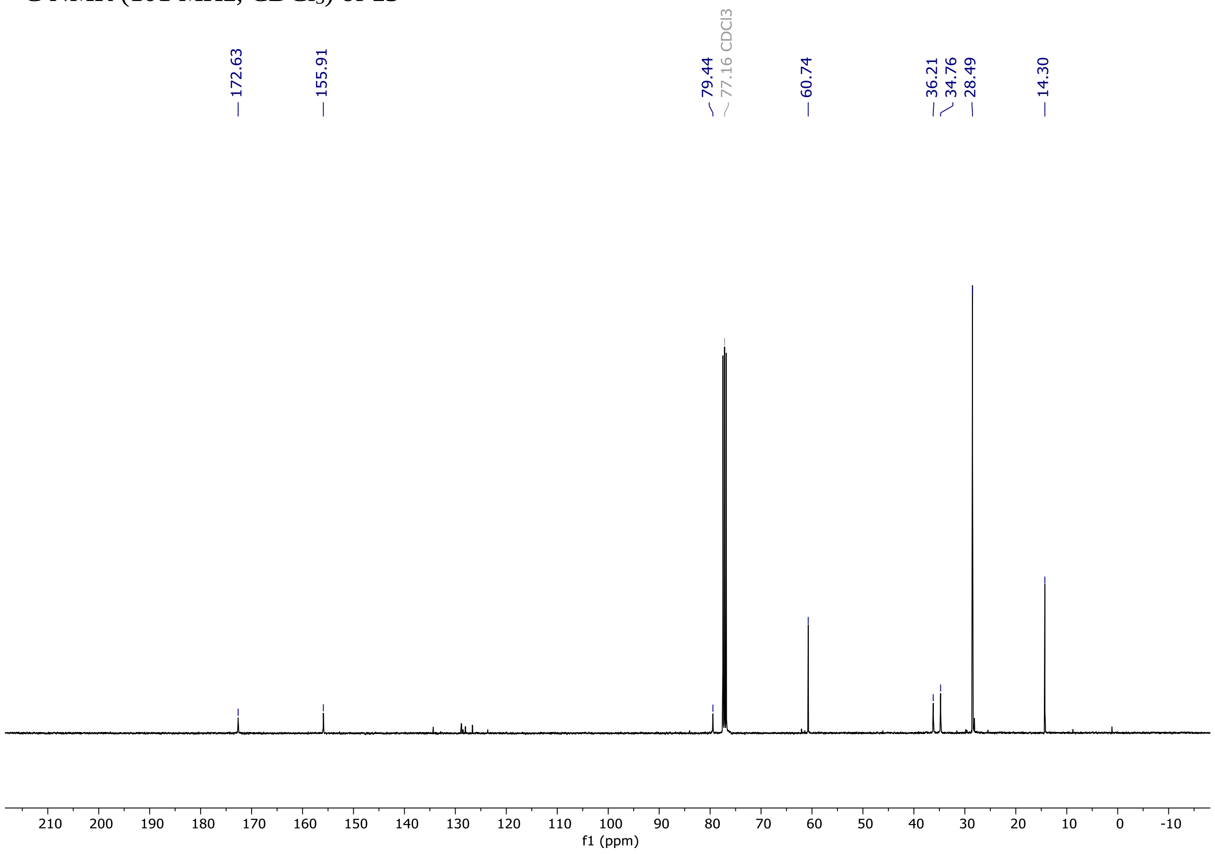

^{1}H NMR (500 MHz,CDCl3) of **14**

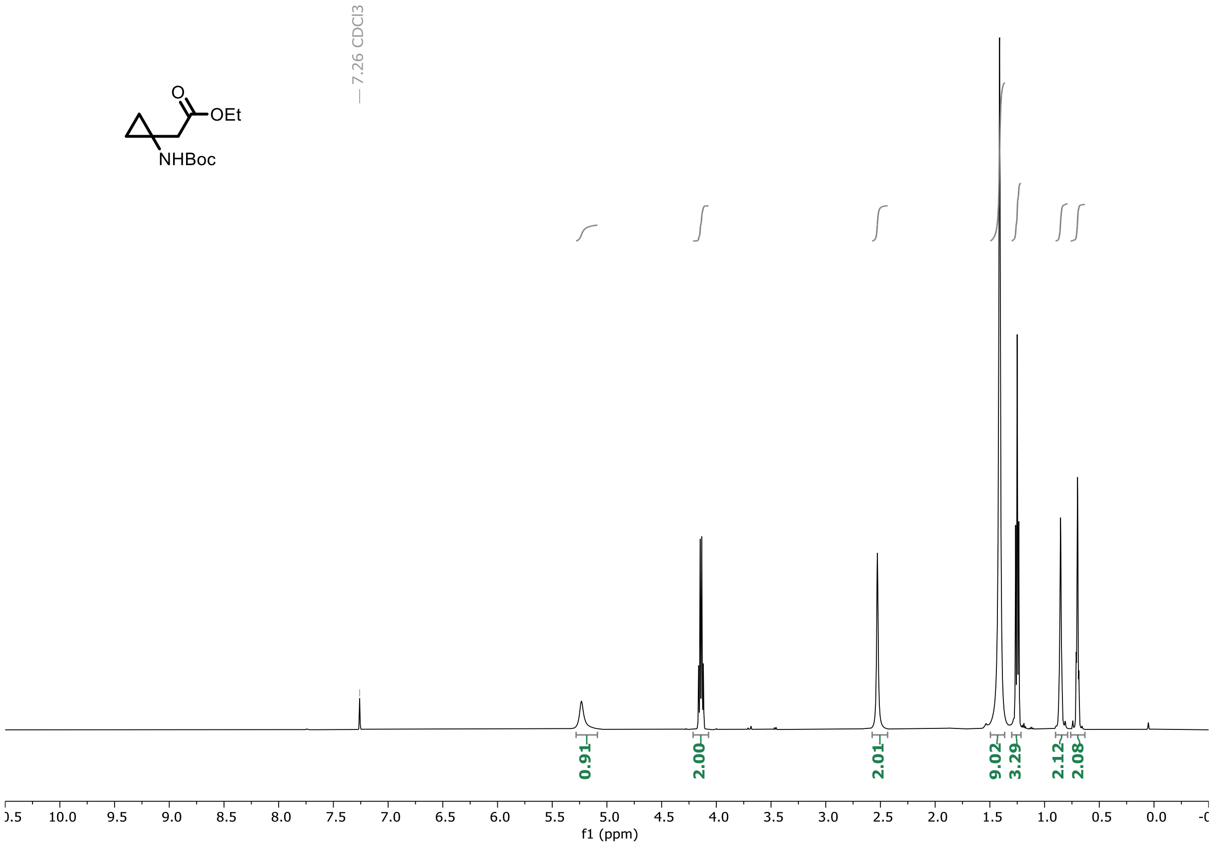

^{13}C NMR (126 MHz, CDCl3) of **14**

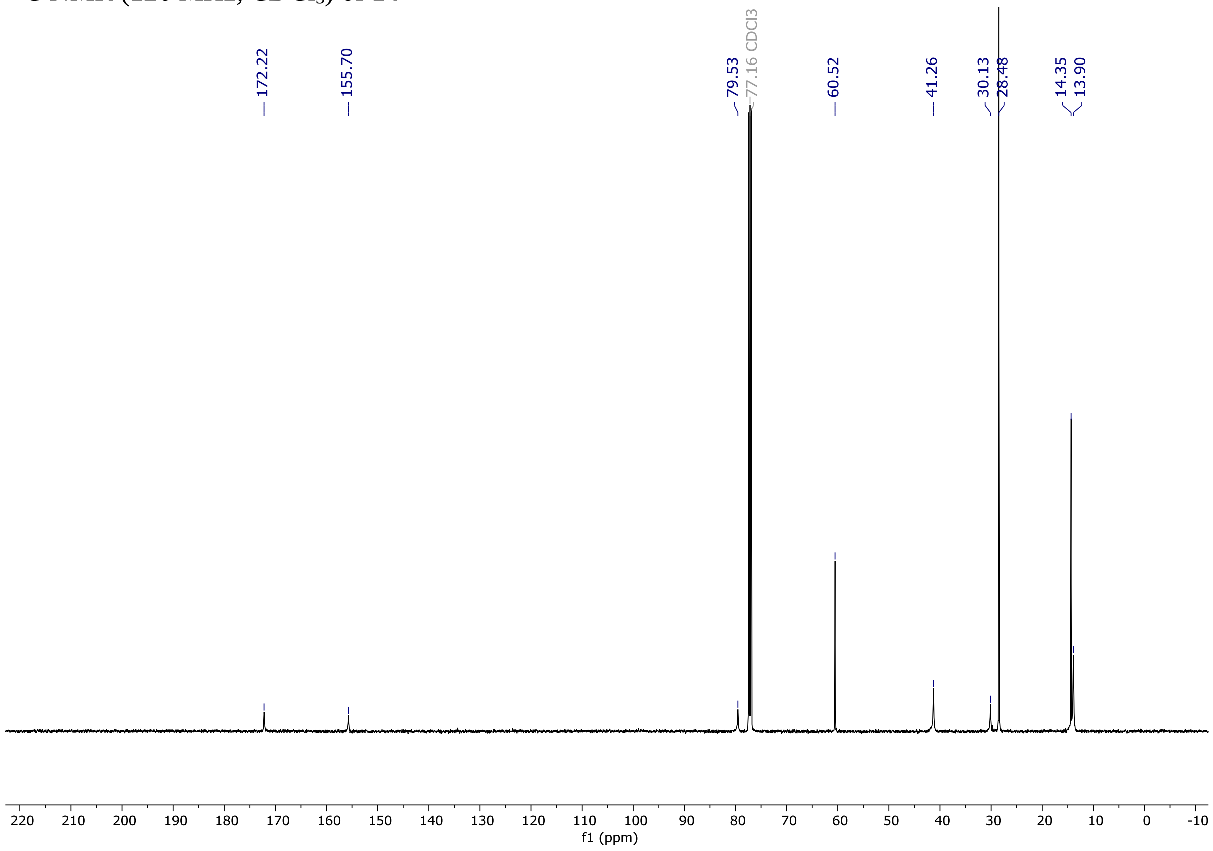

^{1}H NMR (400 MHz, $CDCl_3$) of **15**

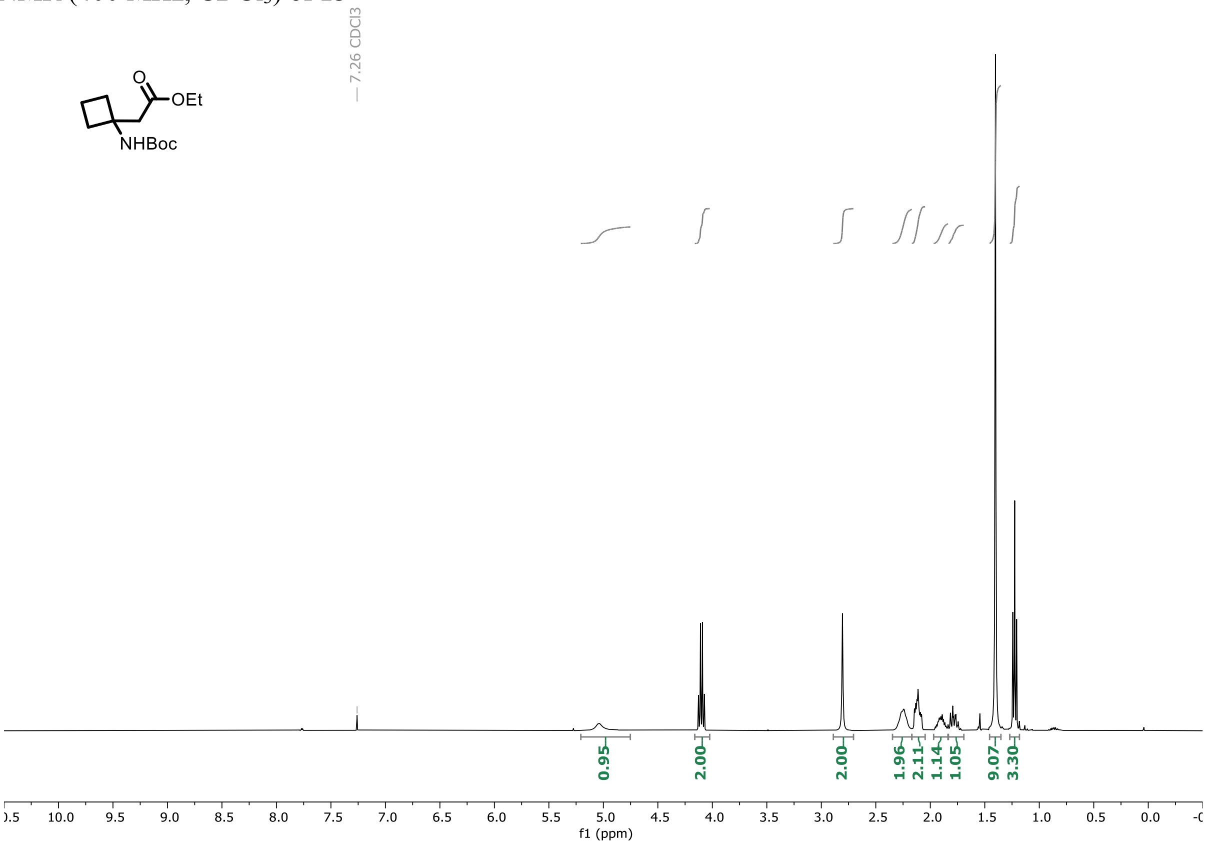

^{13}C NMR (101 MHz, $CDCl_3$) of **15**

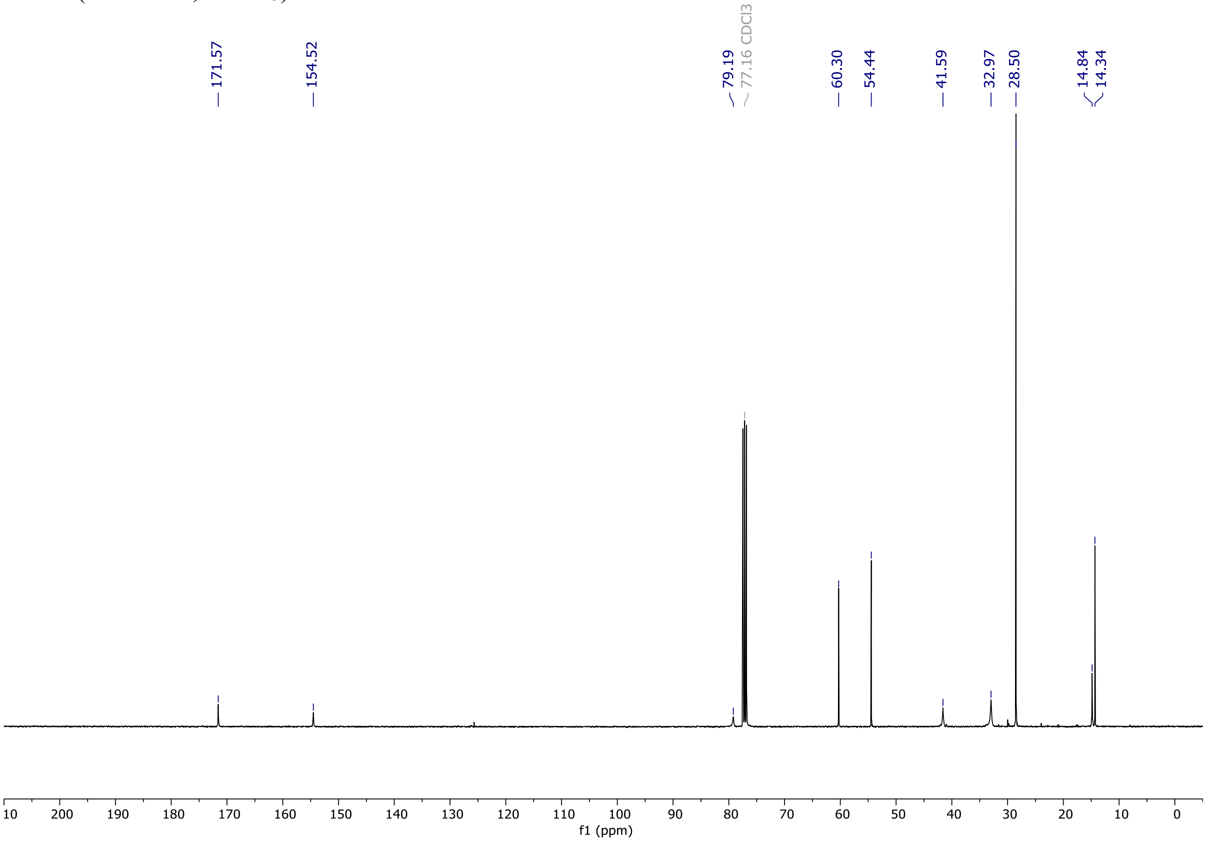

^{1}H NMR (400 MHz,$CDCl_3$) of **16**

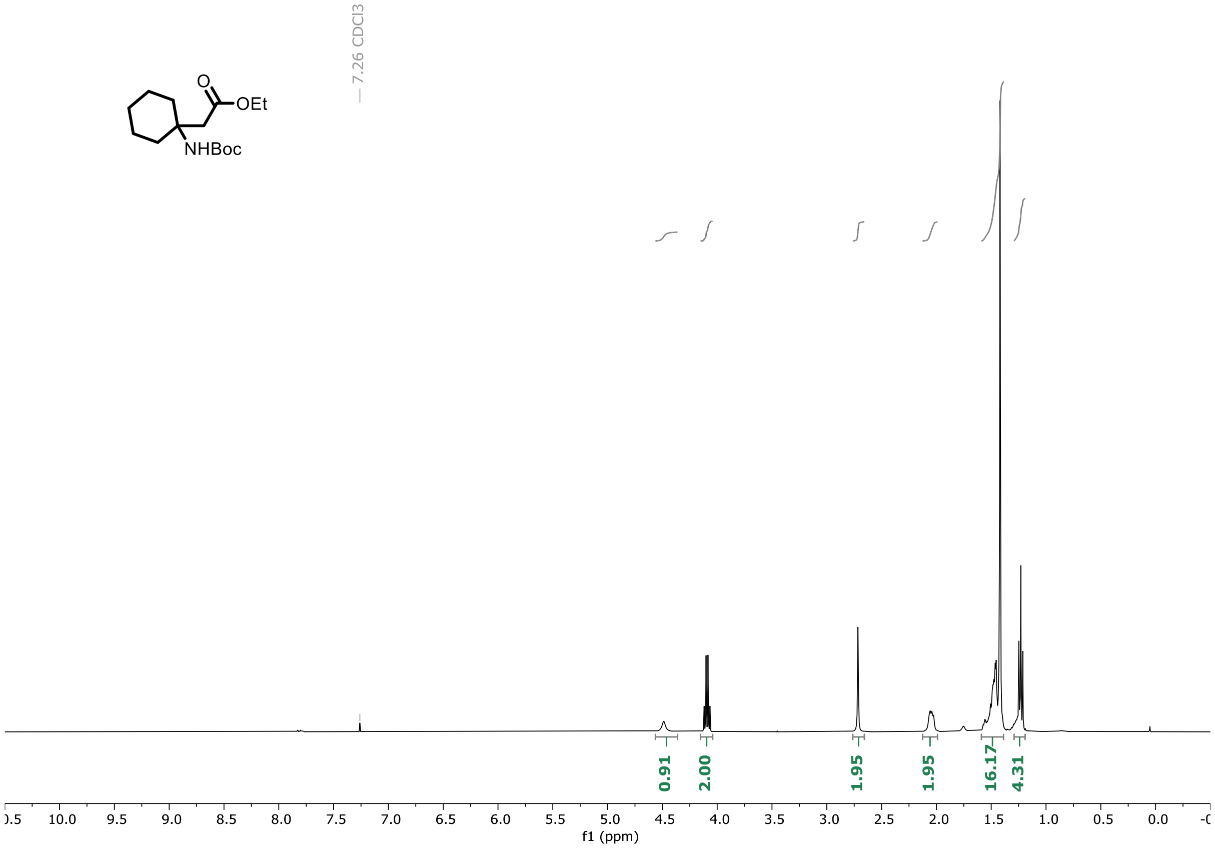

^{13}C NMR (101 MHz, $CDCl_3$) of **16**

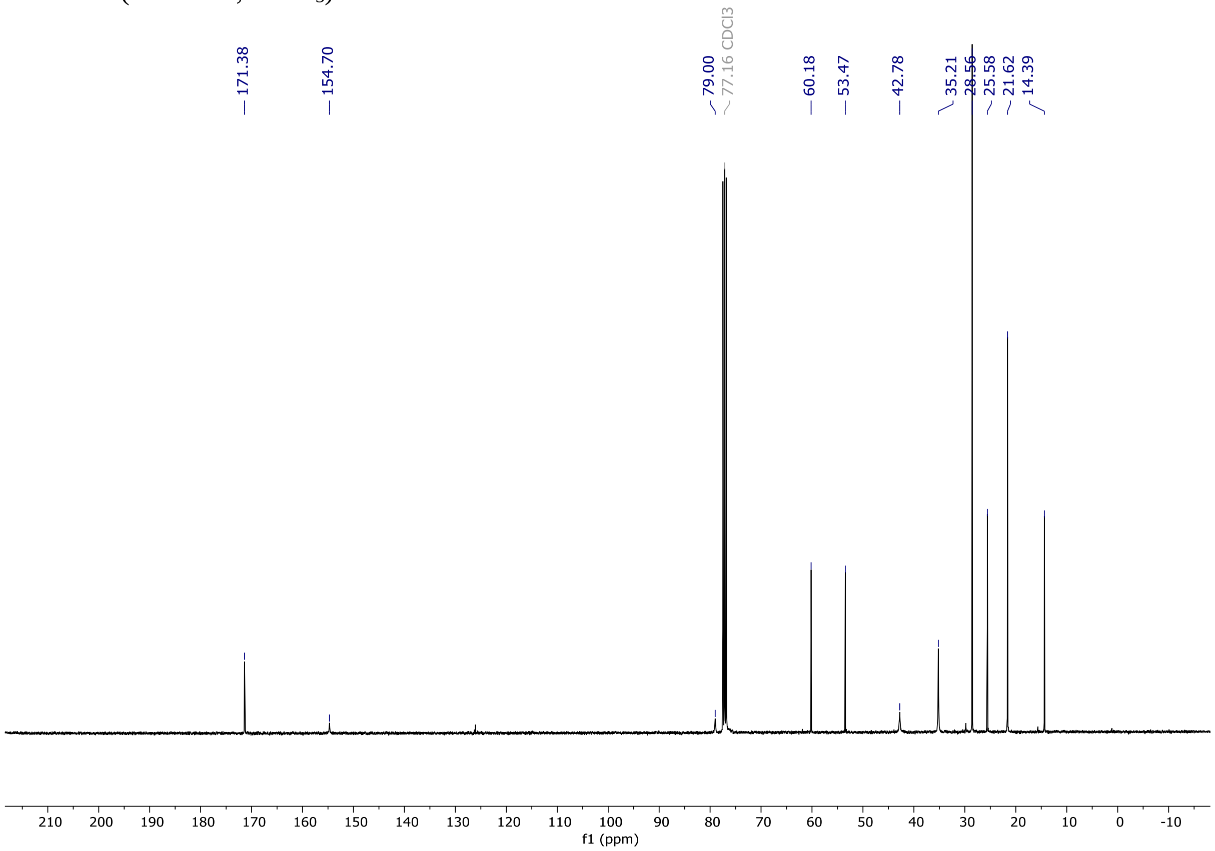

^{1}H NMR (400 MHz, $CDCl_3$) of **17**

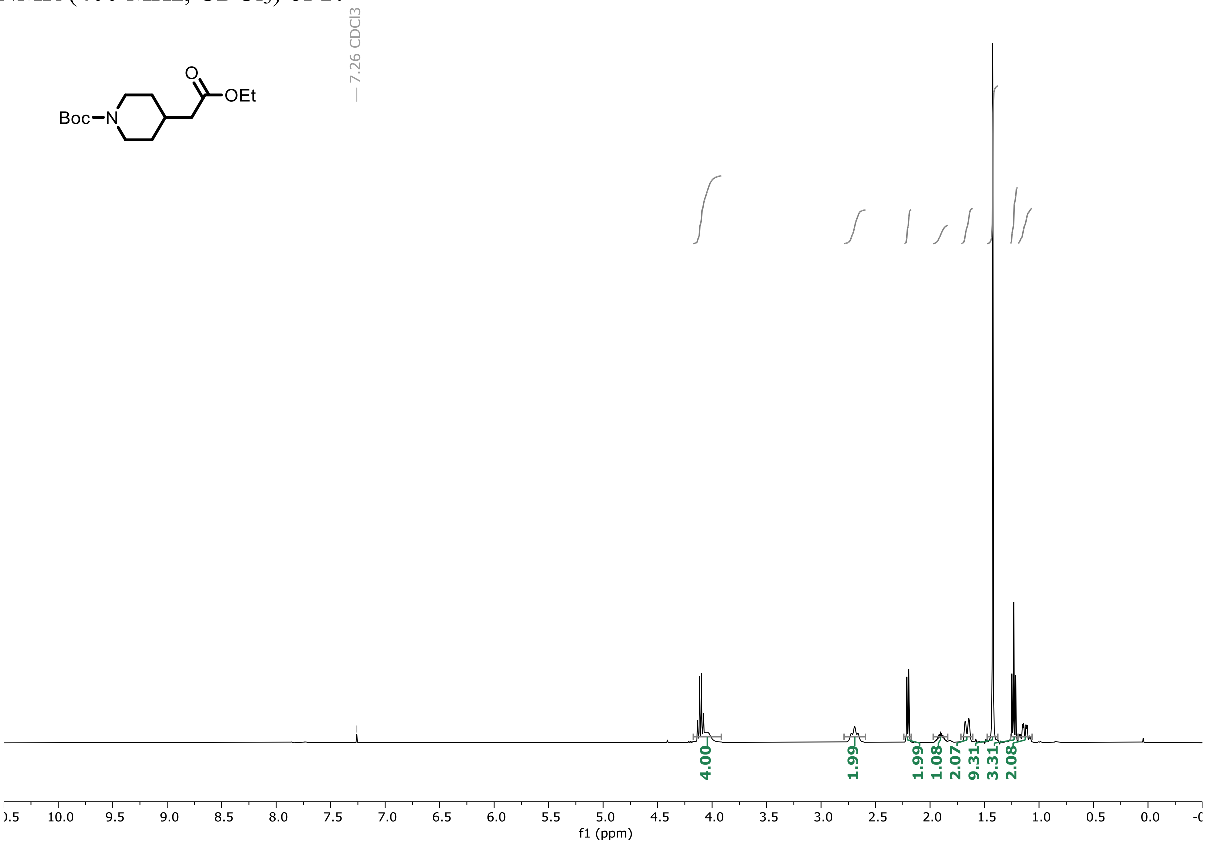

^{13}C NMR (101 MHz, $CDCl_3$) of **17**

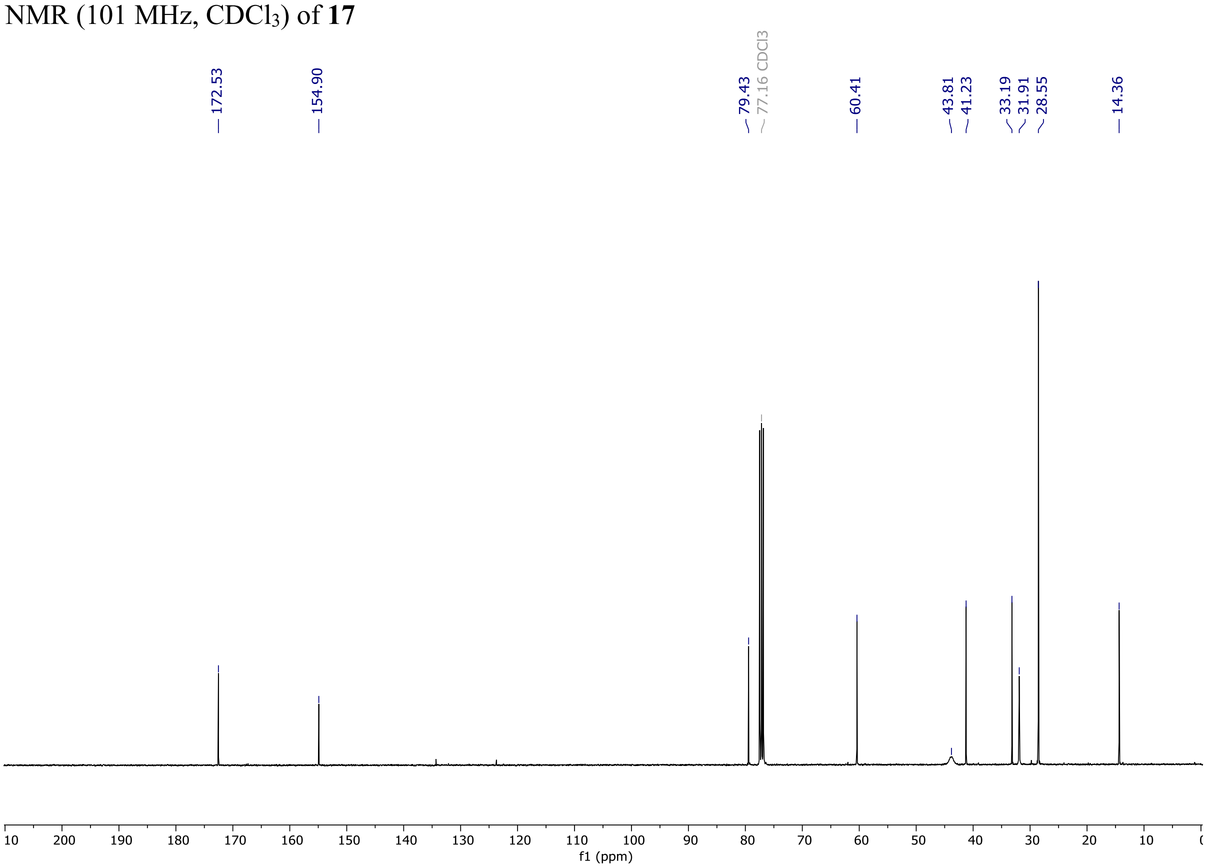

^{1}H NMR (400 MHz, $CDCl_3$) of **18**

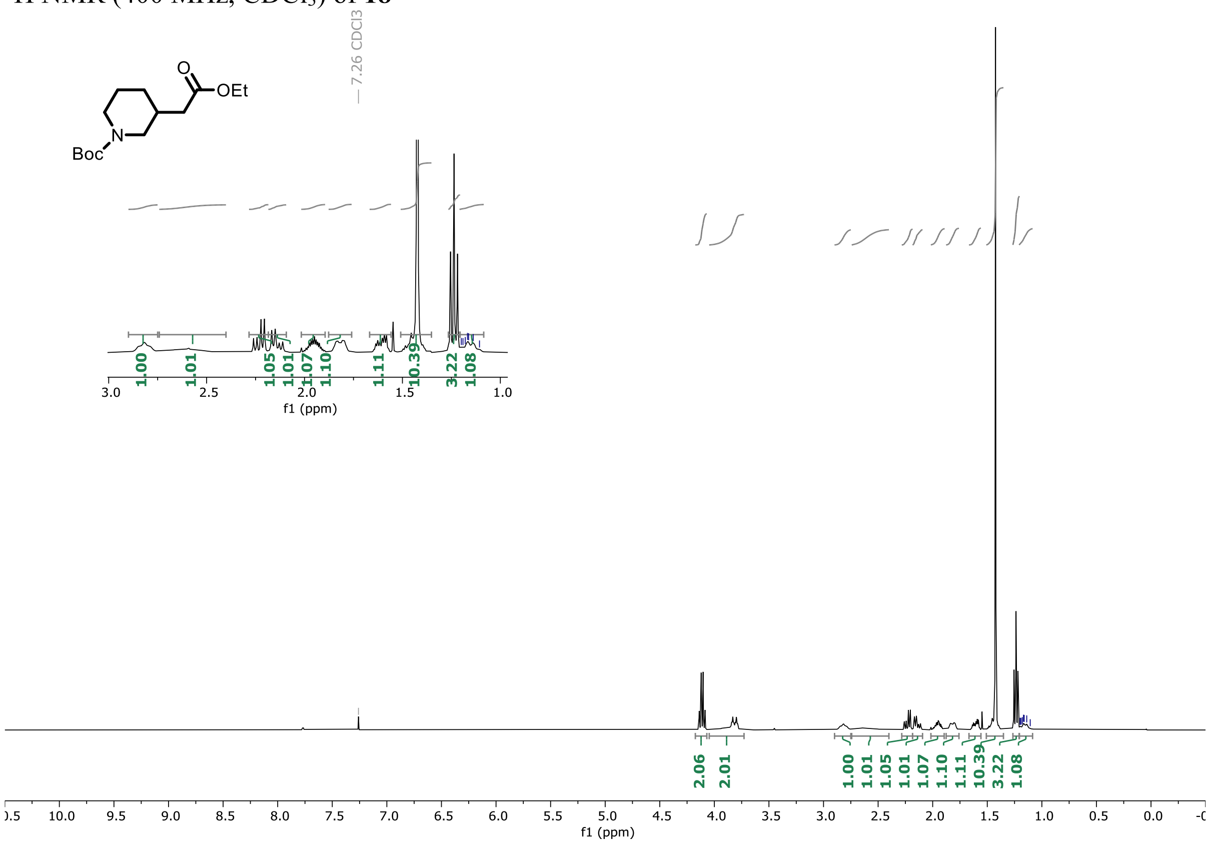

^{13}C NMR (101 MHz, $CDCl_3$) of **18**

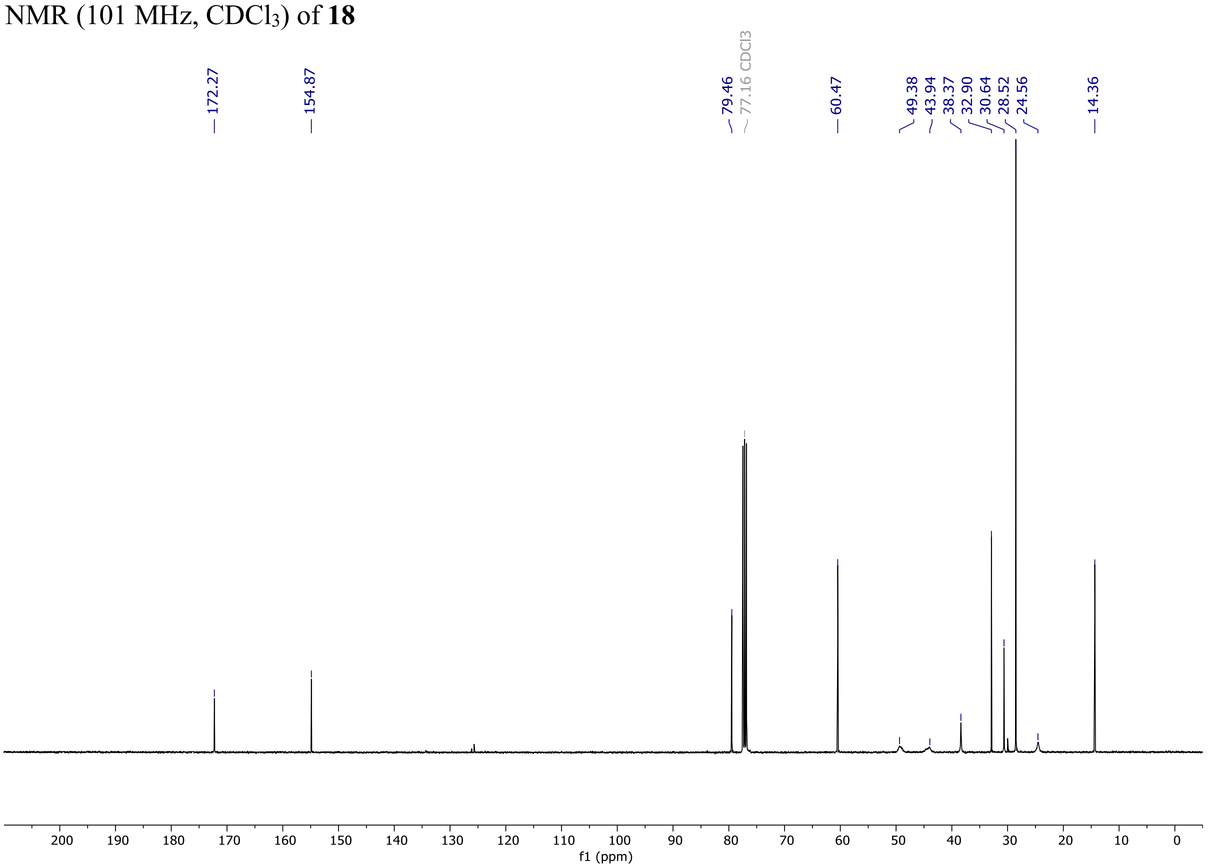

^{1}H NMR (400 MHz,CDCl$_3$) of **19**

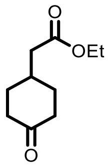

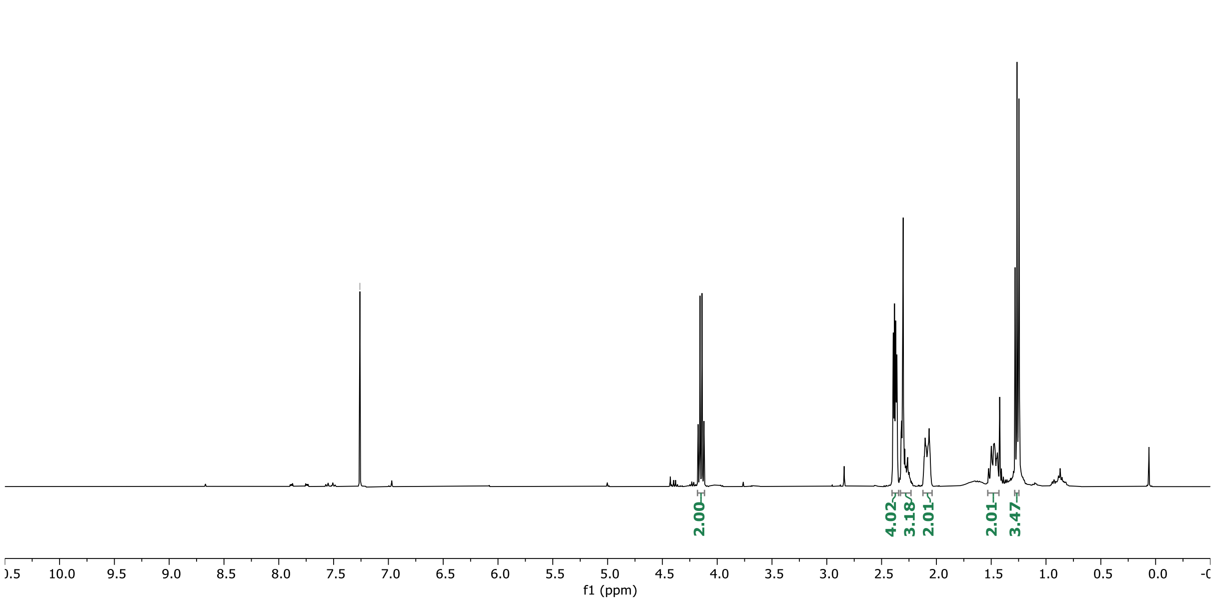

^{13}C NMR (101 MHz, CDCl$_3$) of **19**

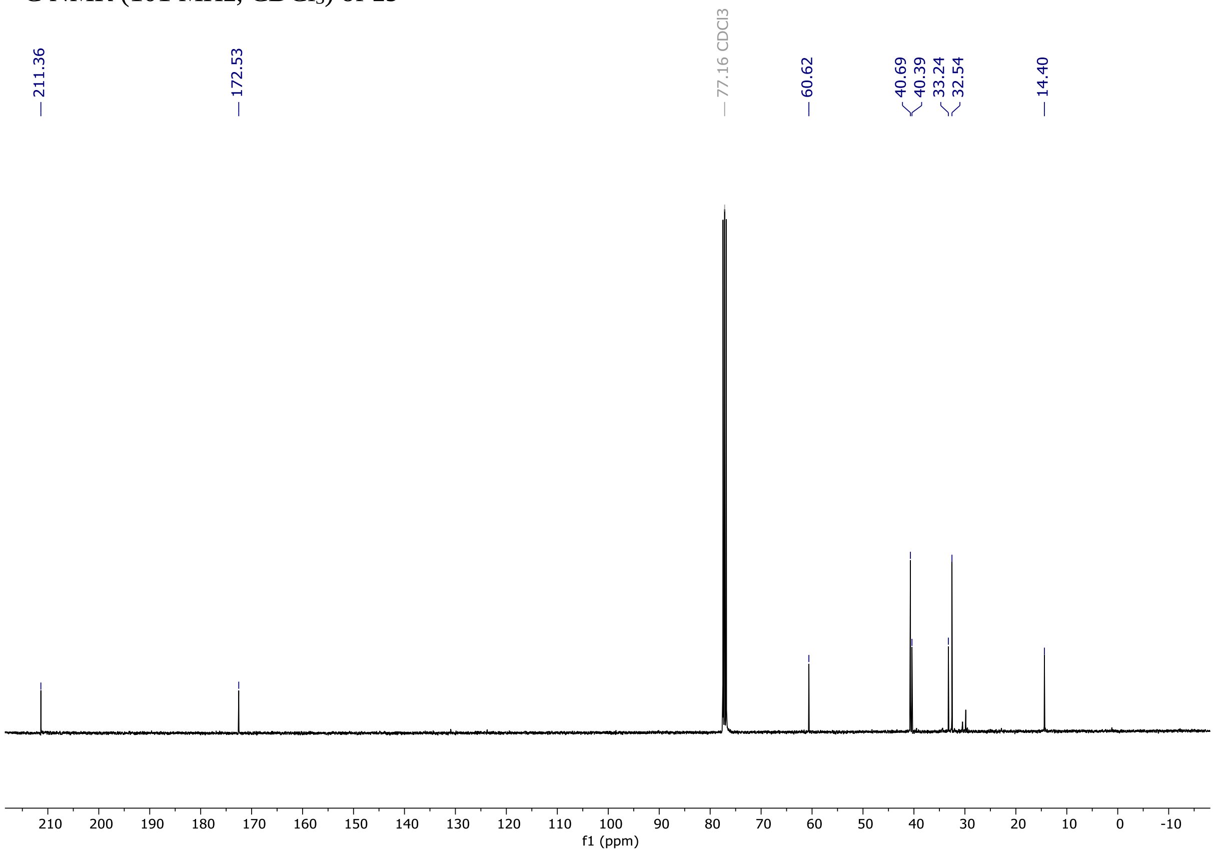

^{1}H NMR (400 MHz, $CDCl_3$) of **20**

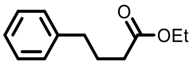

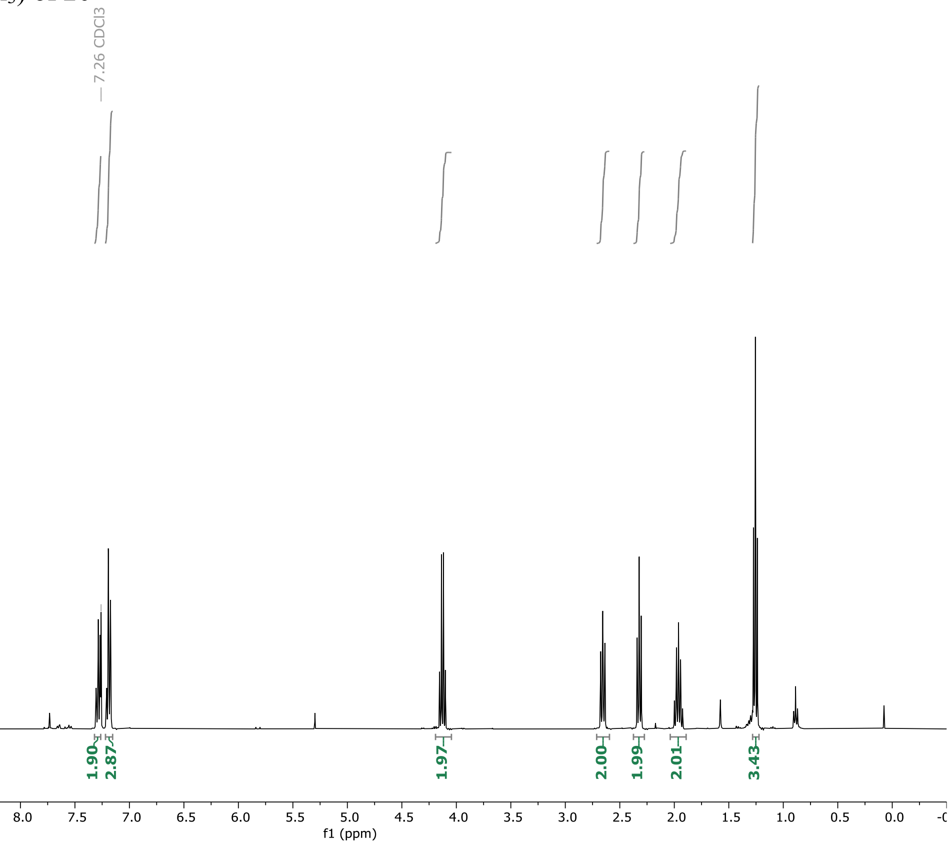

^{13}C NMR (101 MHz, $CDCl_3$) of **20**

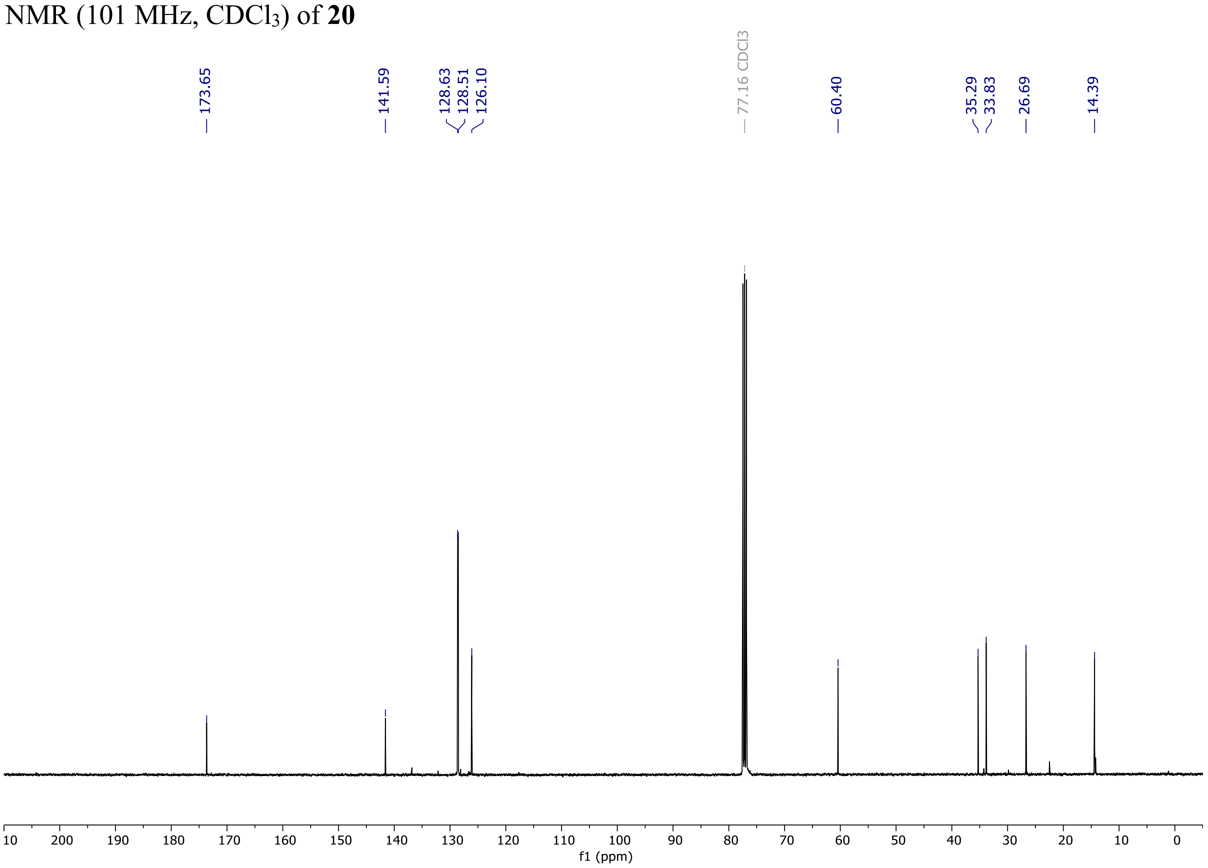

^{1}H NMR (400 MHz, $CDCl_3$) of **21**

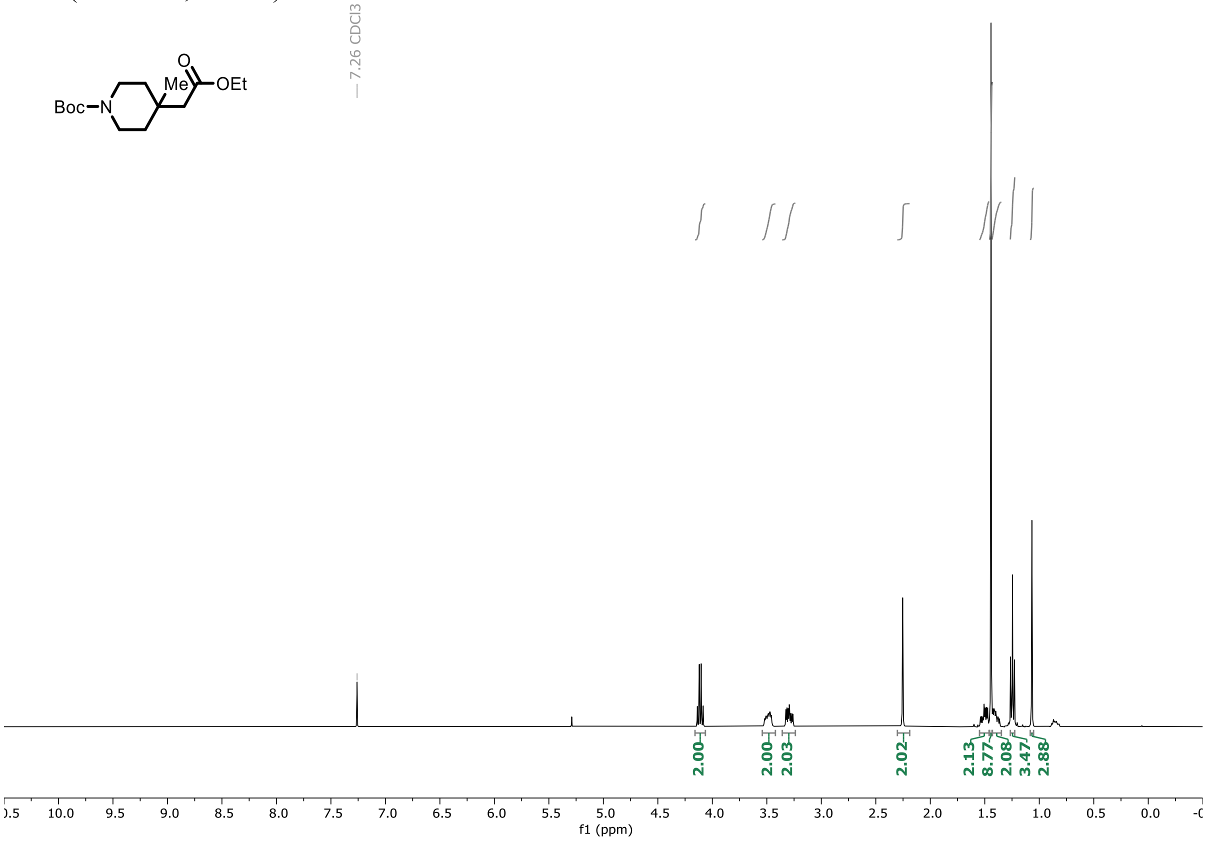

^{13}C NMR (101 MHz, $CDCl_3$) of **21**

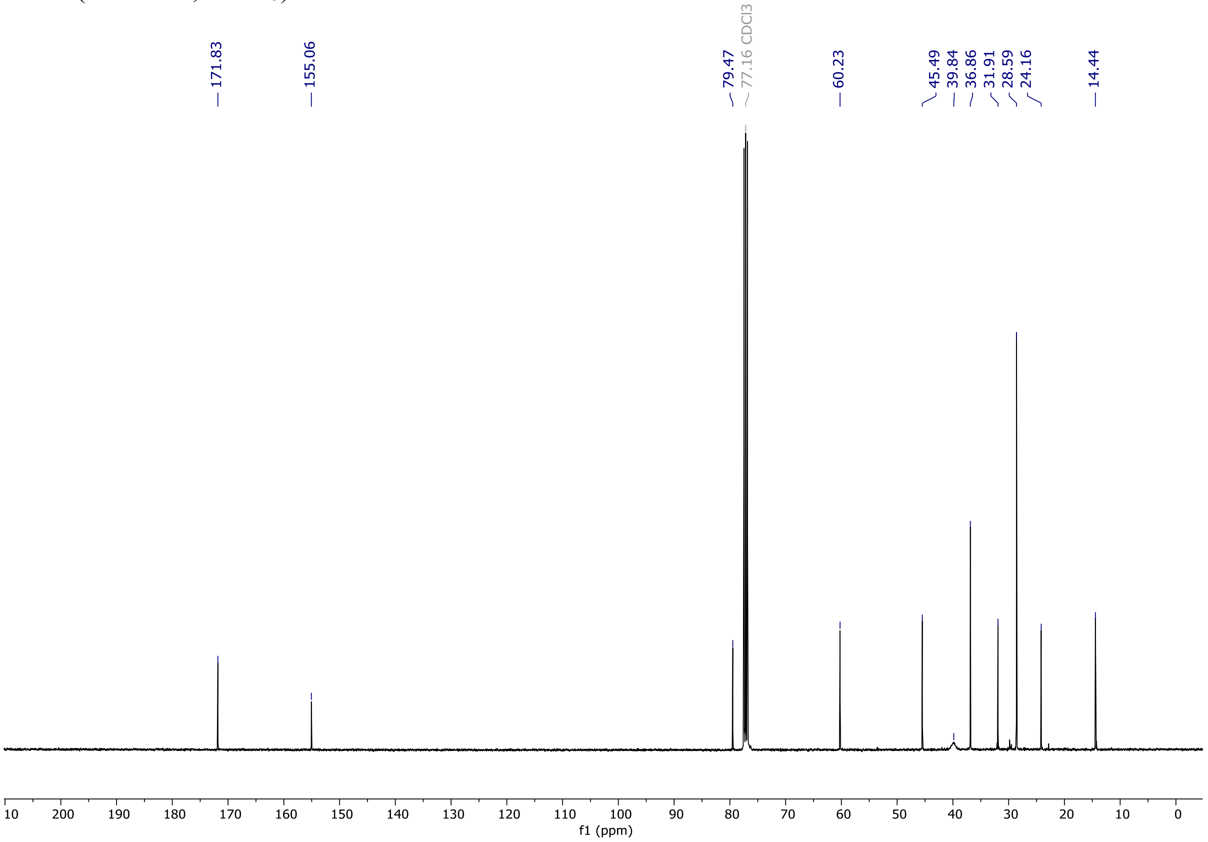

^{1}H NMR (400 MHz, $CDCl_3$) of **22**

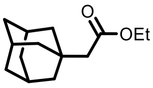

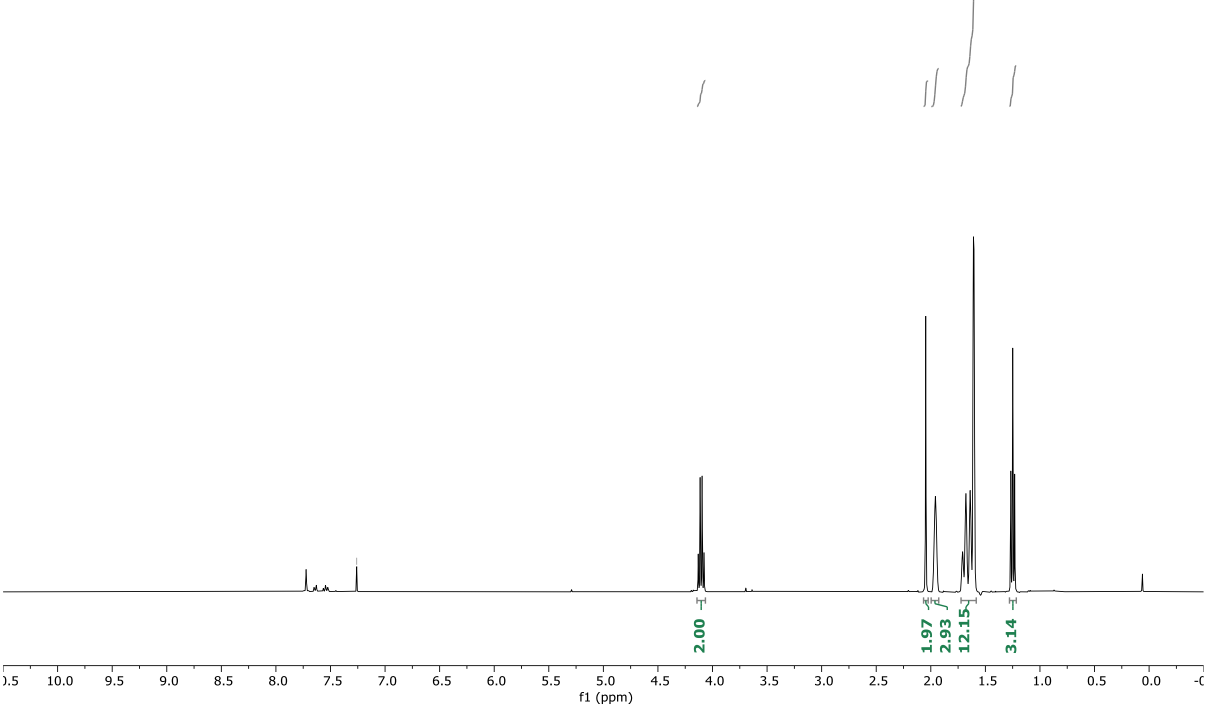

^{13}C NMR (101 MHz, $CDCl_3$) of **22**

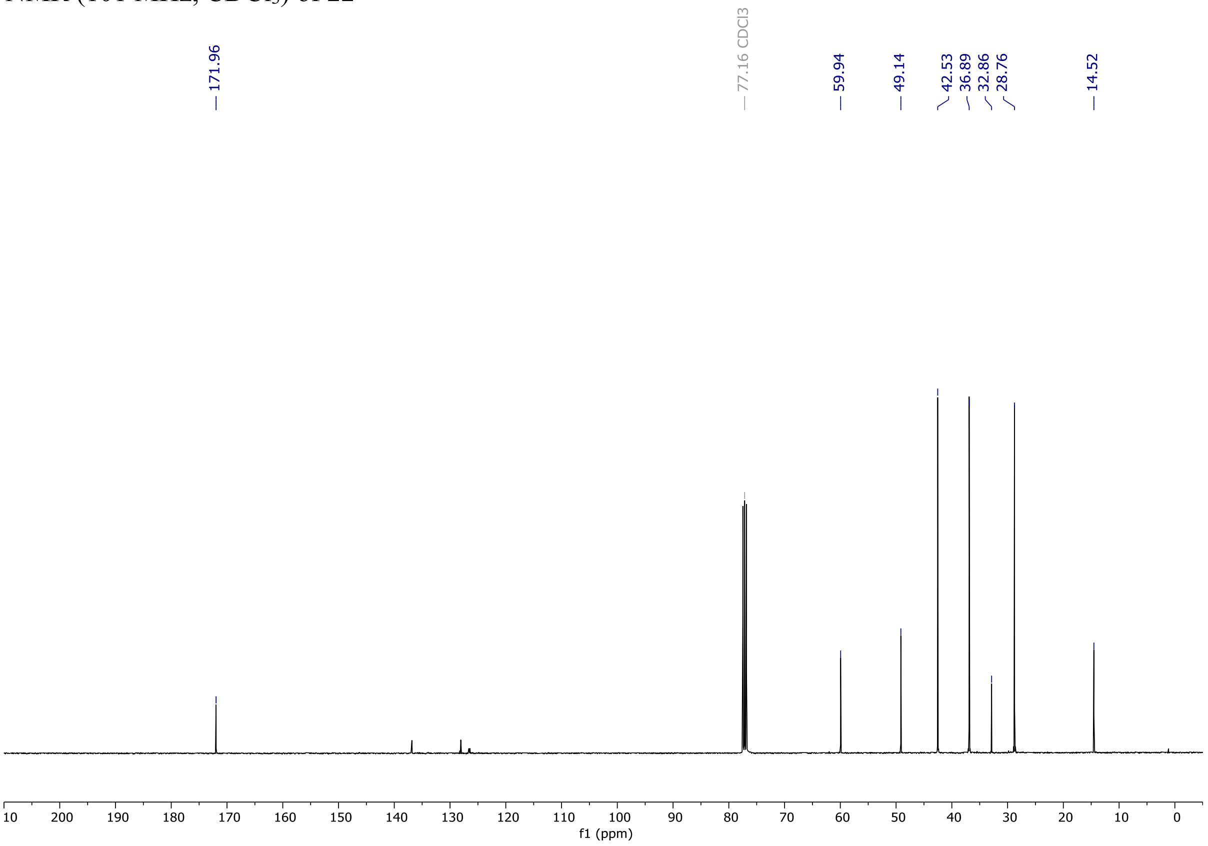

^{1}H NMR (400 MHz, $CDCl_3$) of **23**

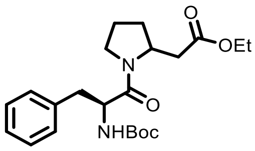

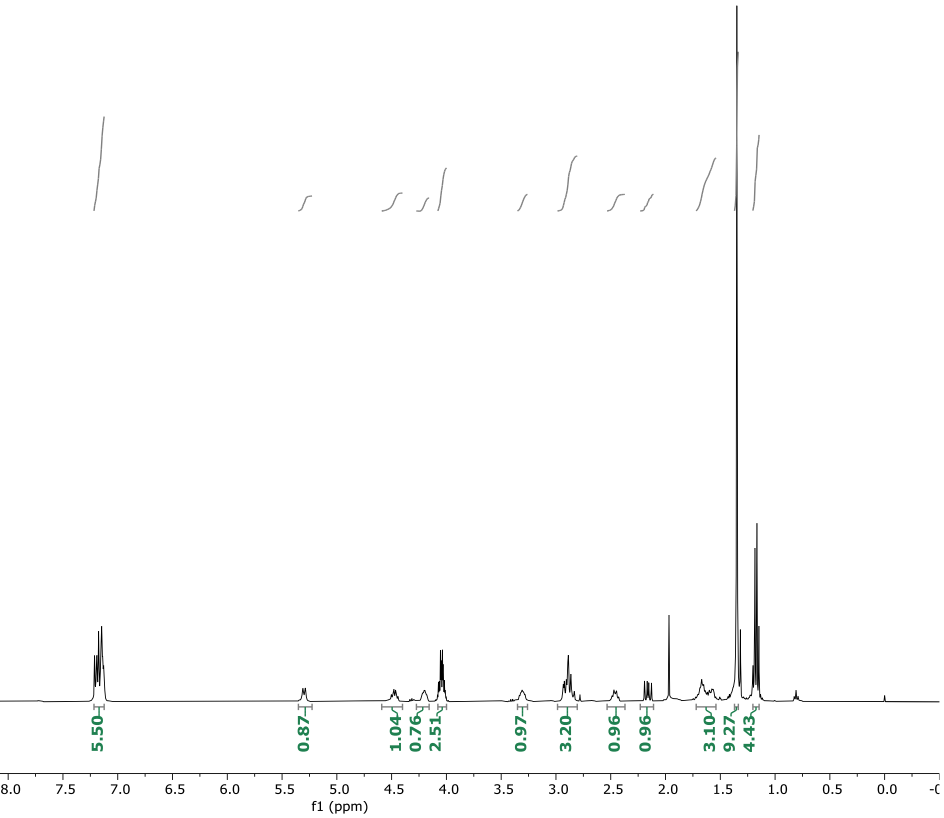

^{13}C NMR (101 MHz, $CDCl_3$) of **23**

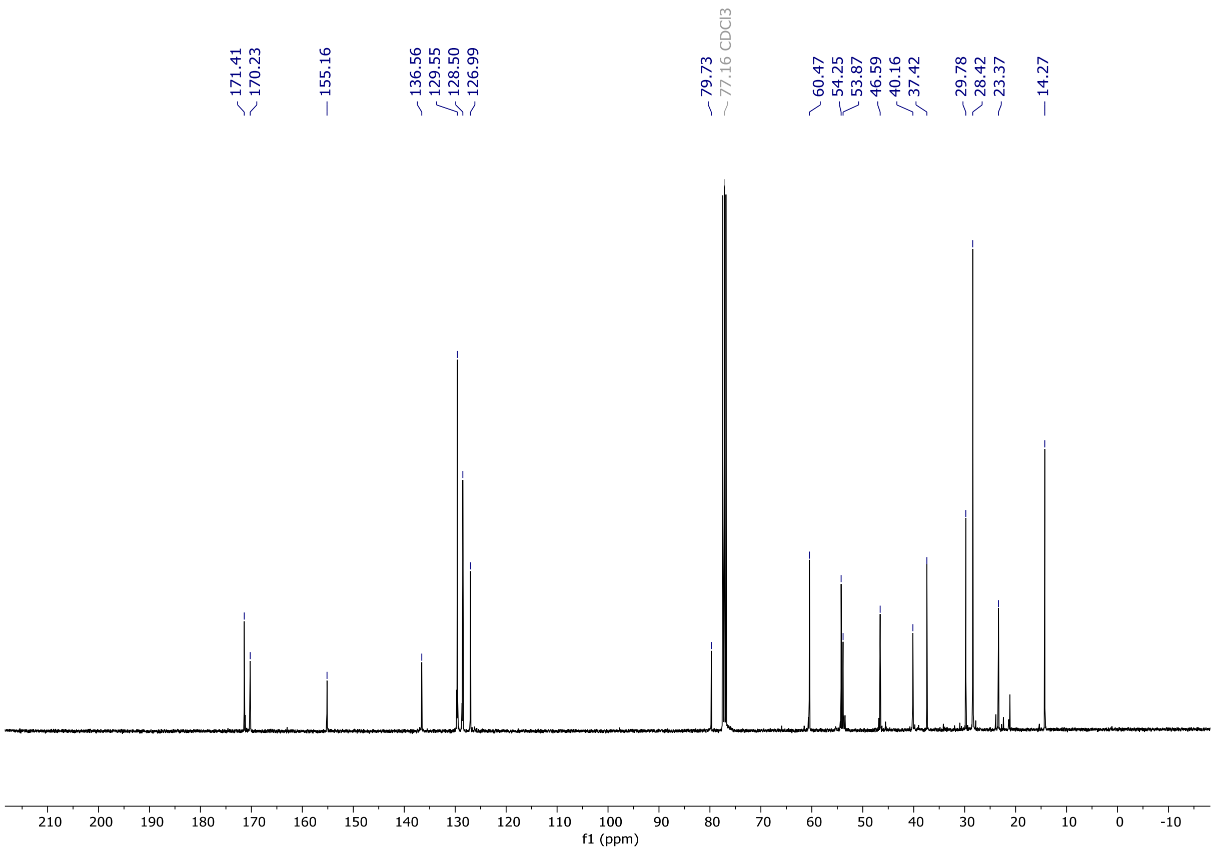

^{1}H NMR (400 MHz, $CDCl_3$) of **24**

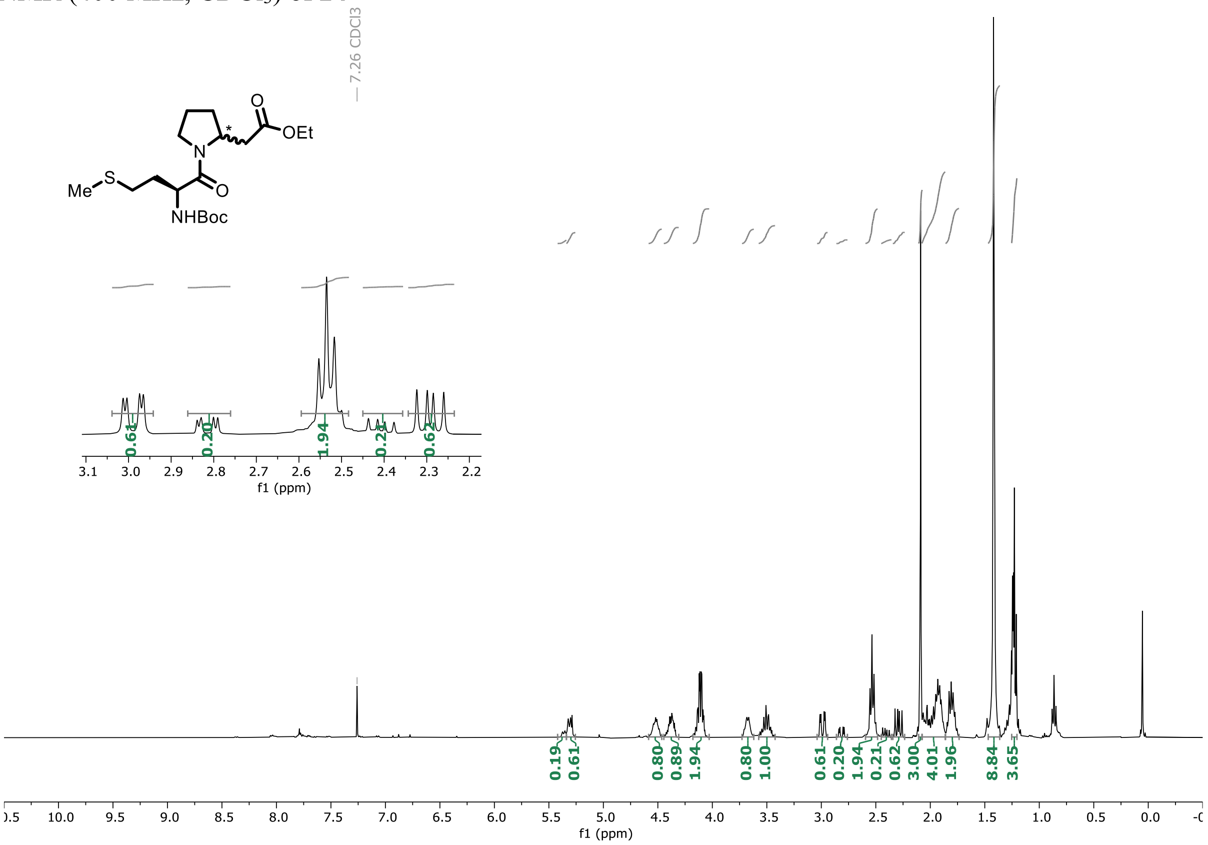

^{13}C NMR (101 MHz, $CDCl_3$) of **24**

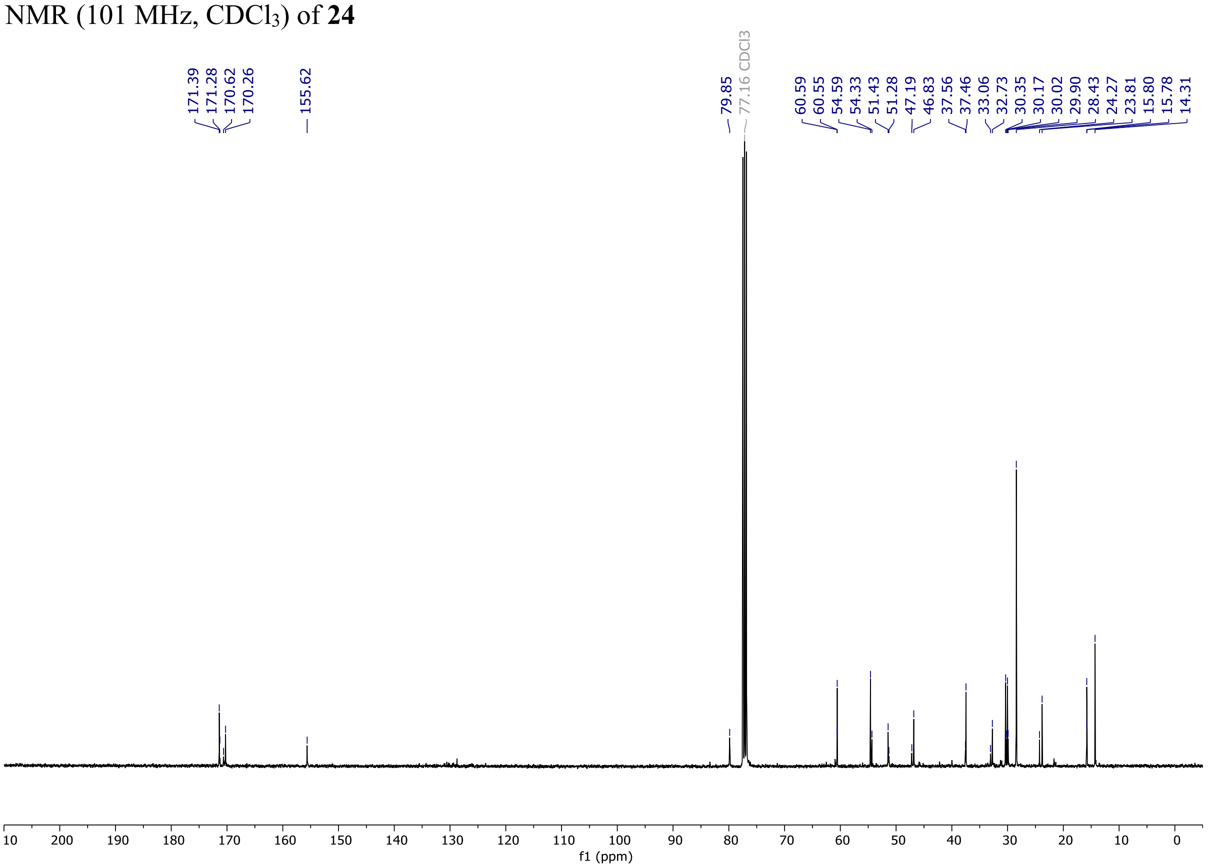

^{1}H NMR (400 MHz, $CDCl_3$) of **25**

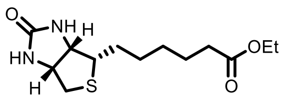

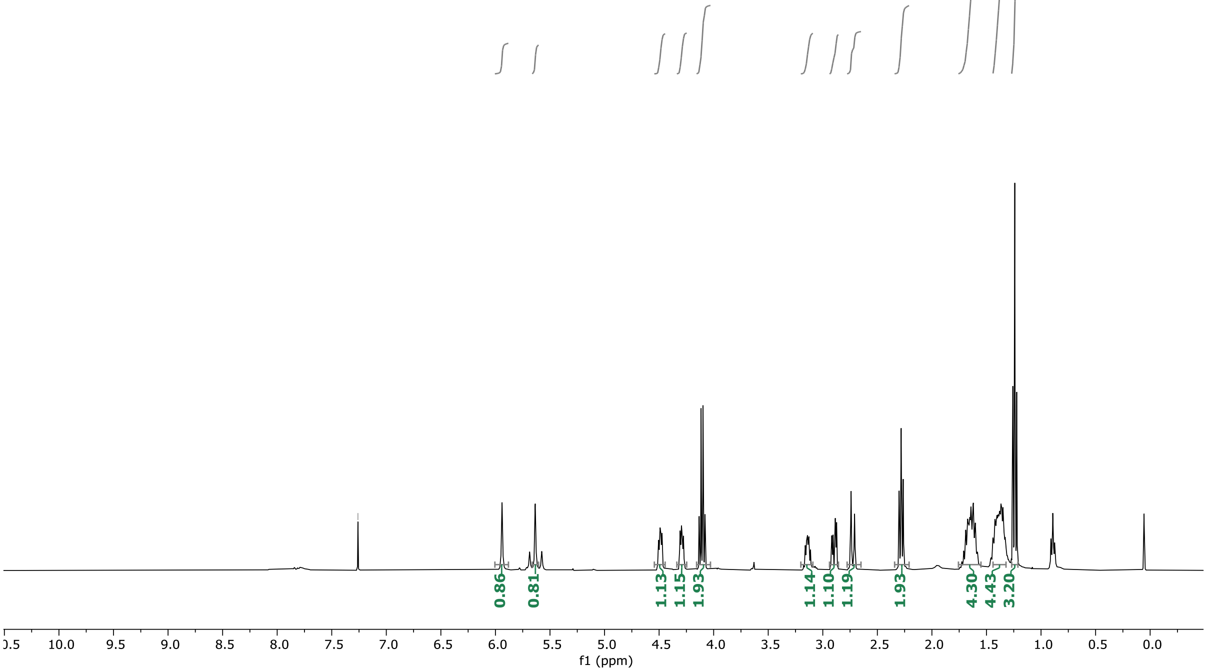

^{13}C NMR (101 MHz, $CDCl_3$) of **25**

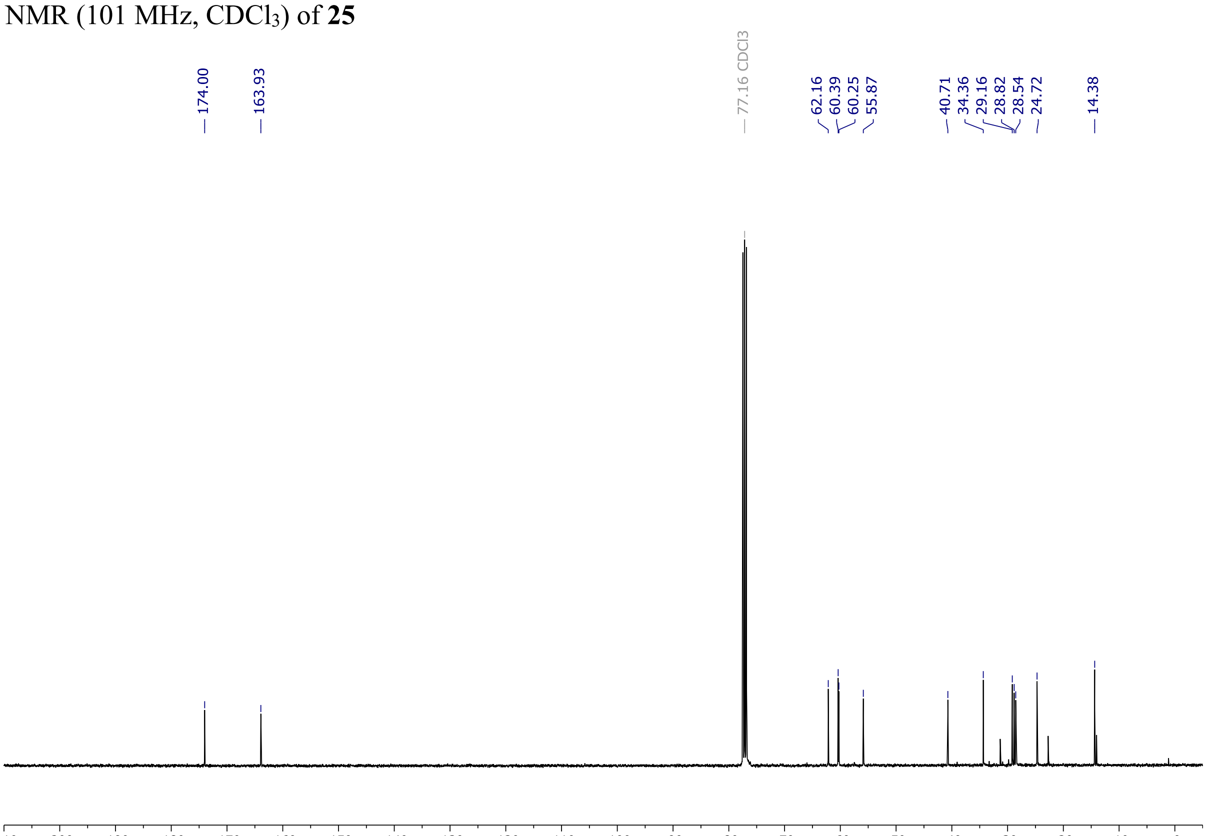

^{1}H NMR (400 MHz, $CDCl_3$) of **26**

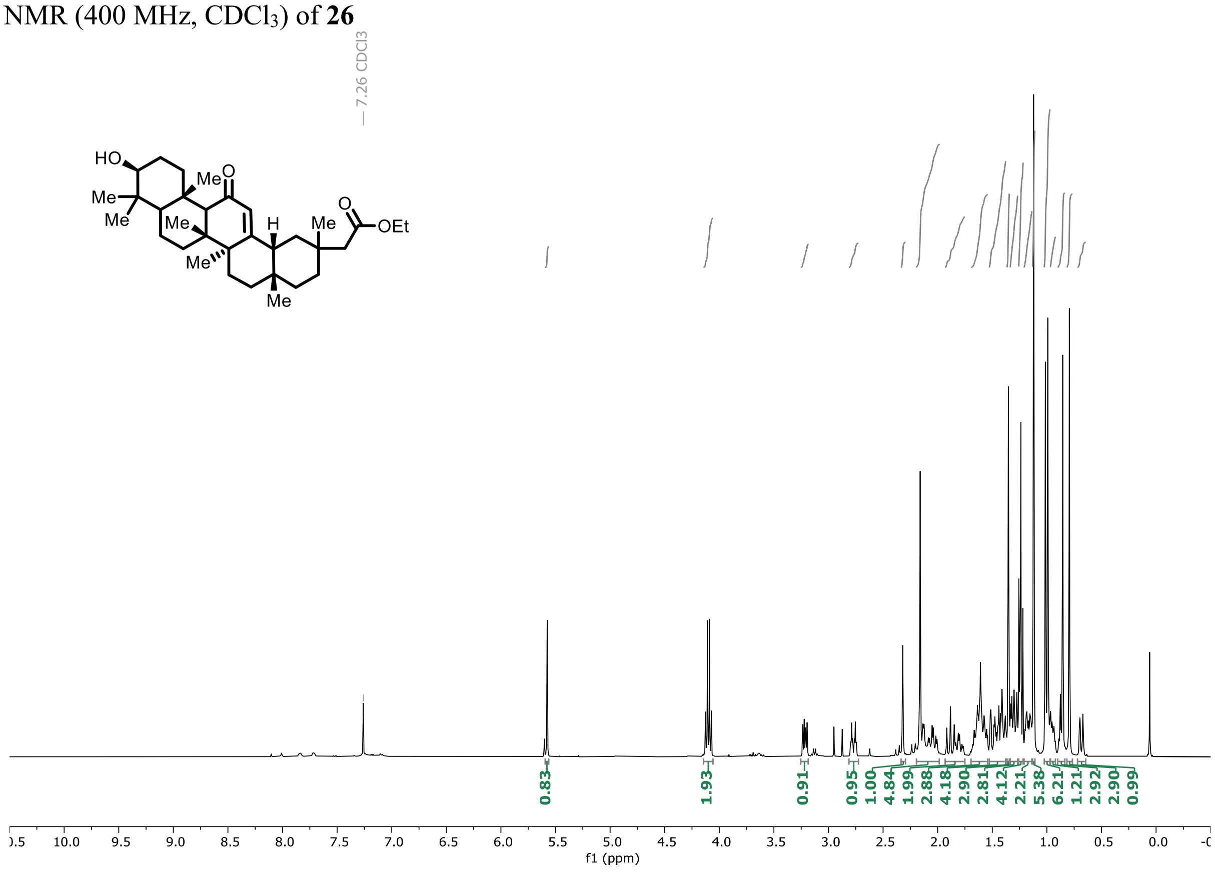

^{13}C NMR (101 MHz, $CDCl_3$) of **26**

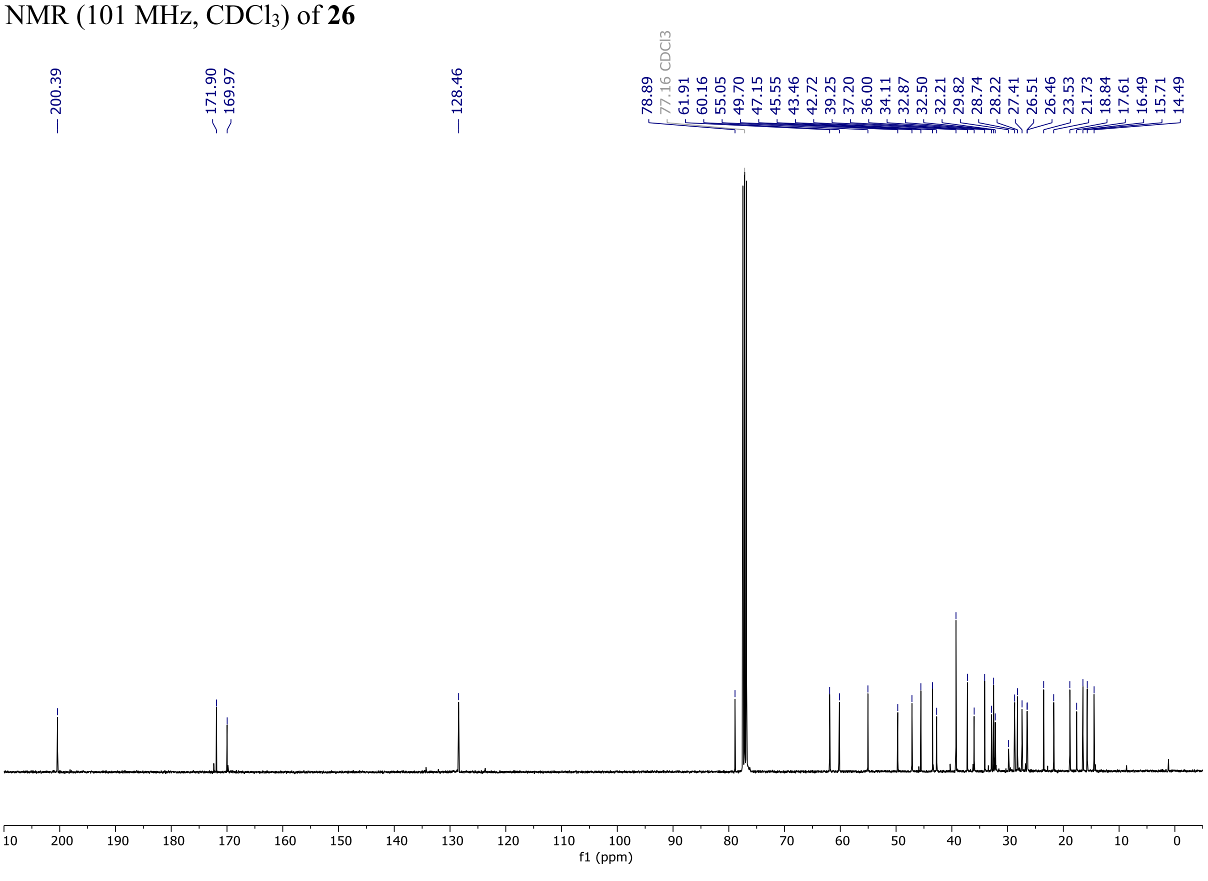

16. NMR spectra of products 27-58 (alkylation)

^{1}H NMR (400 MHz, $CDCl_3$) of **27**

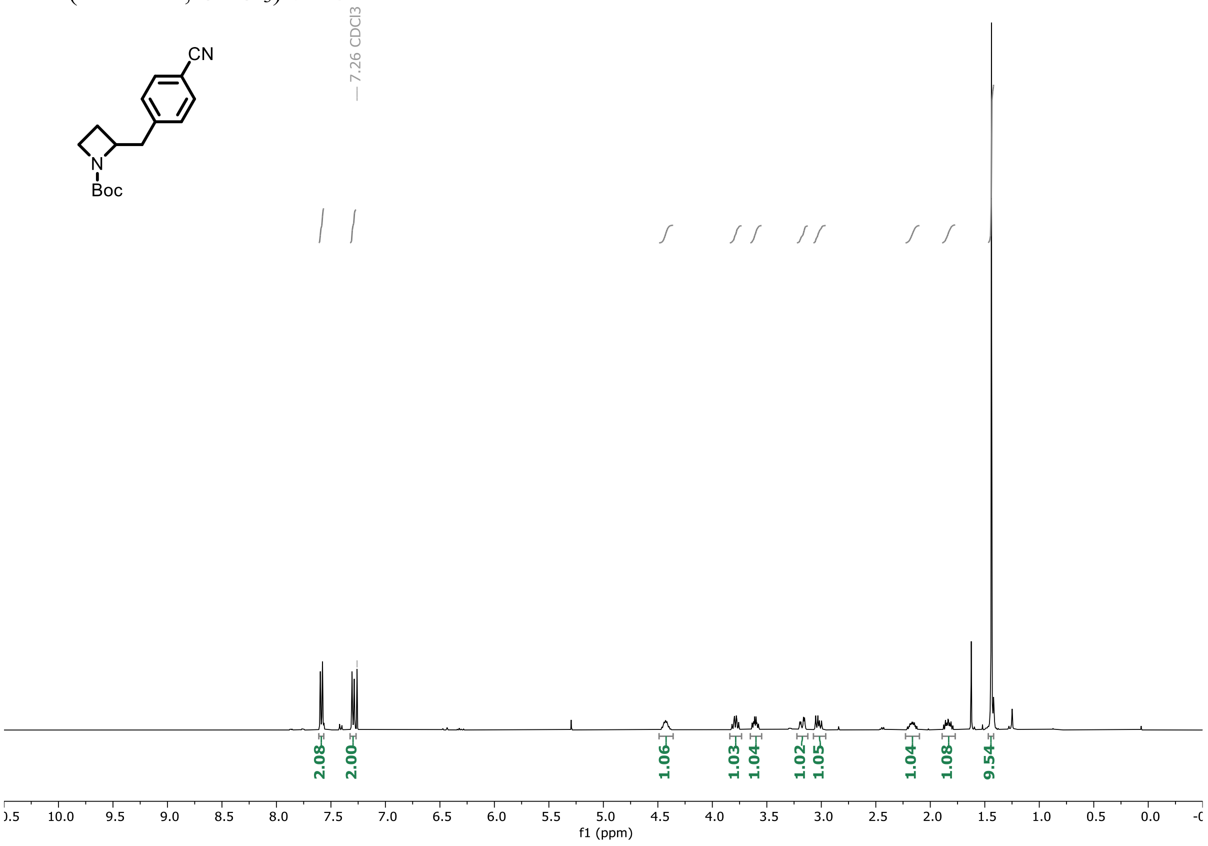

^{13}C NMR (101 MHz, $CDCl_3$) of **27**

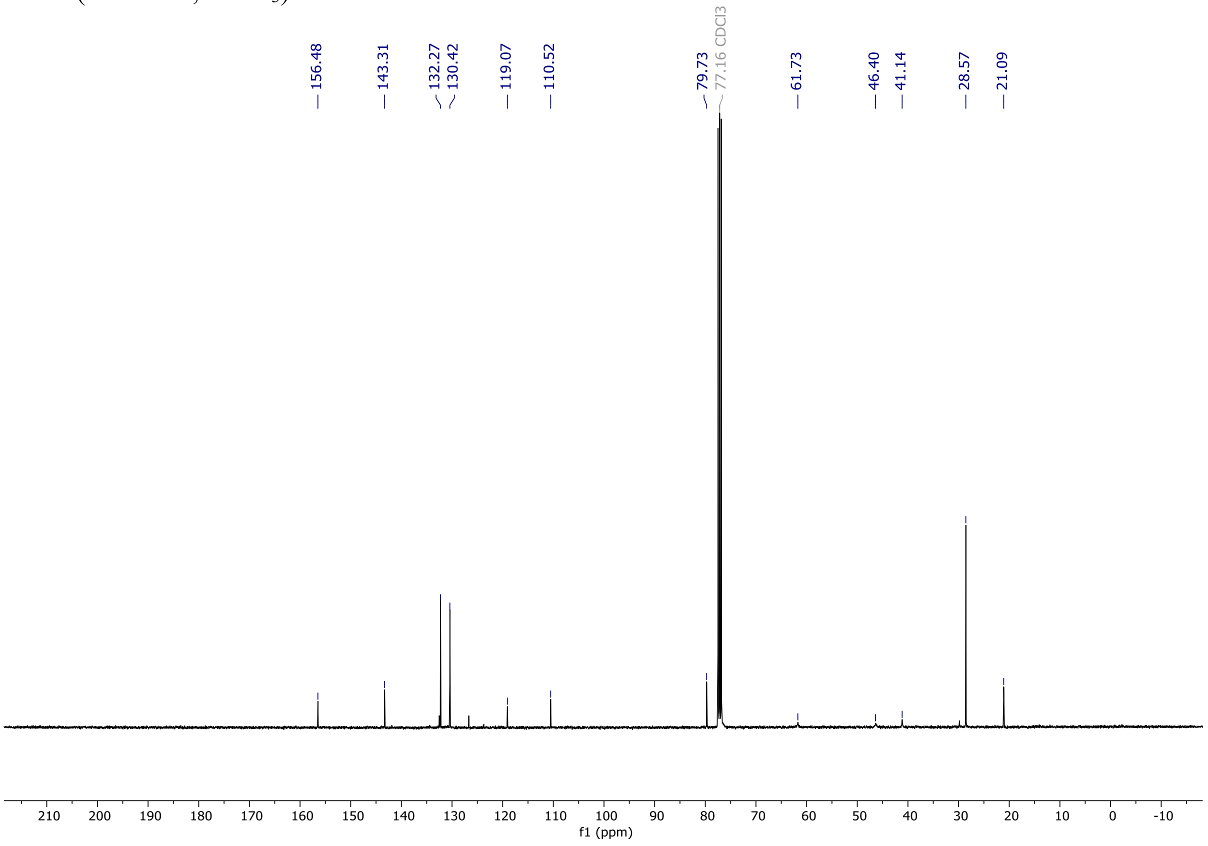

^{1}H NMR (400 MHz,$CDCl_3$) of **28**

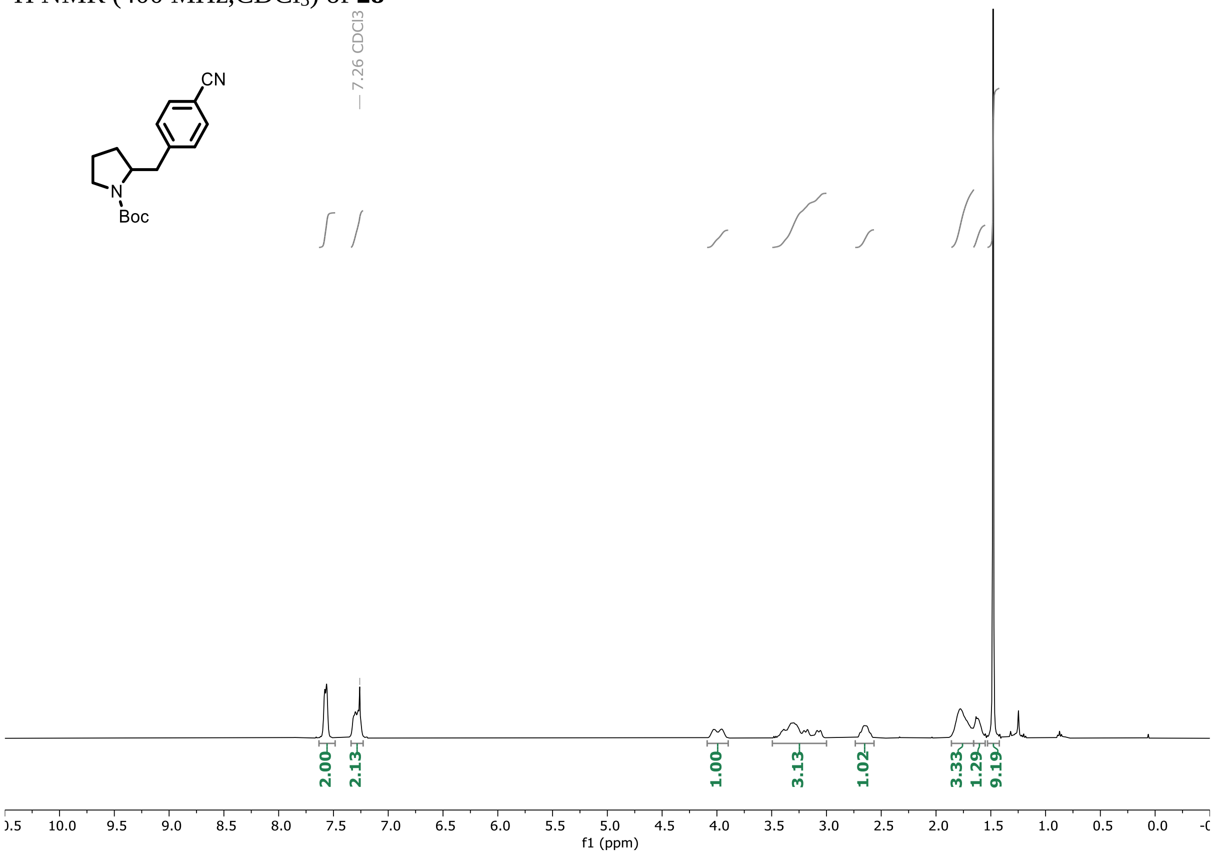

^{13}C NMR (101 MHz, $CDCl_3$) of **28**

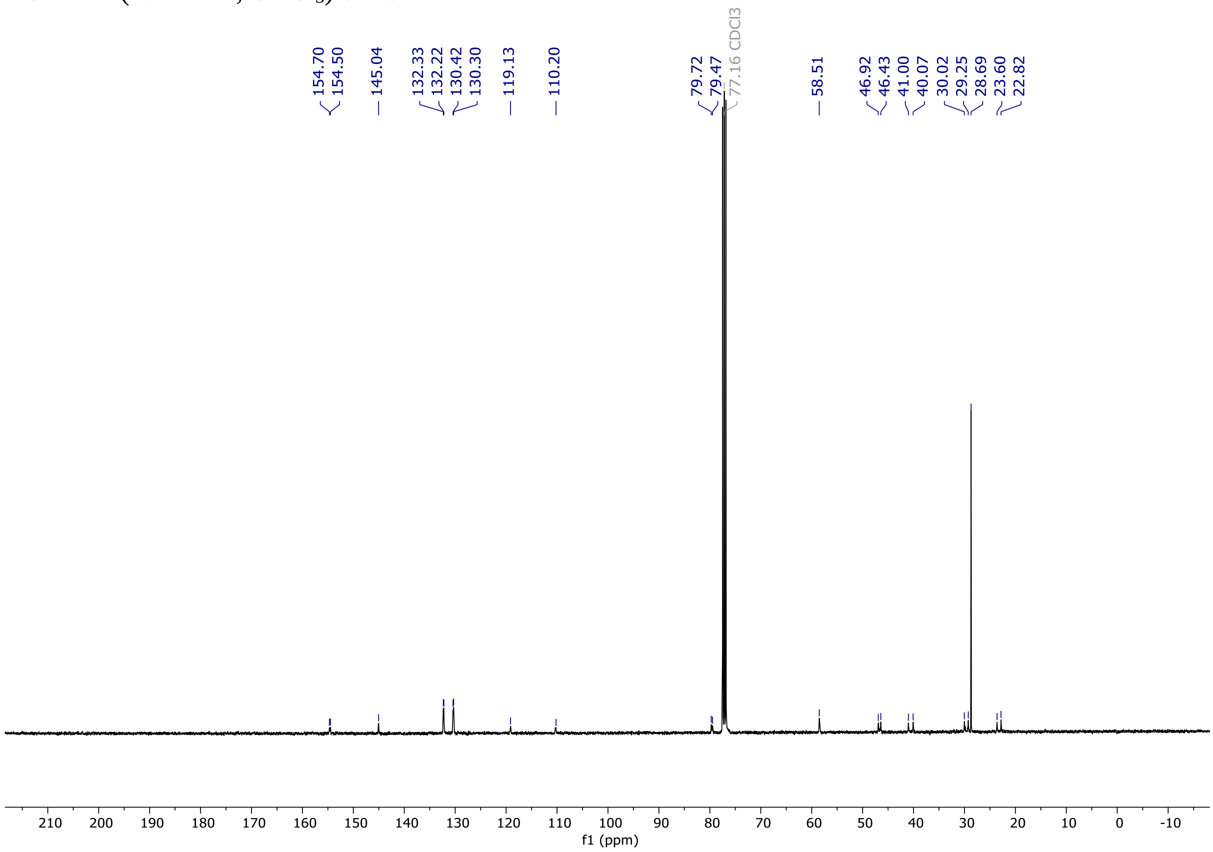

^{1}H NMR (400 MHz, $CDCl_3$) of **29**

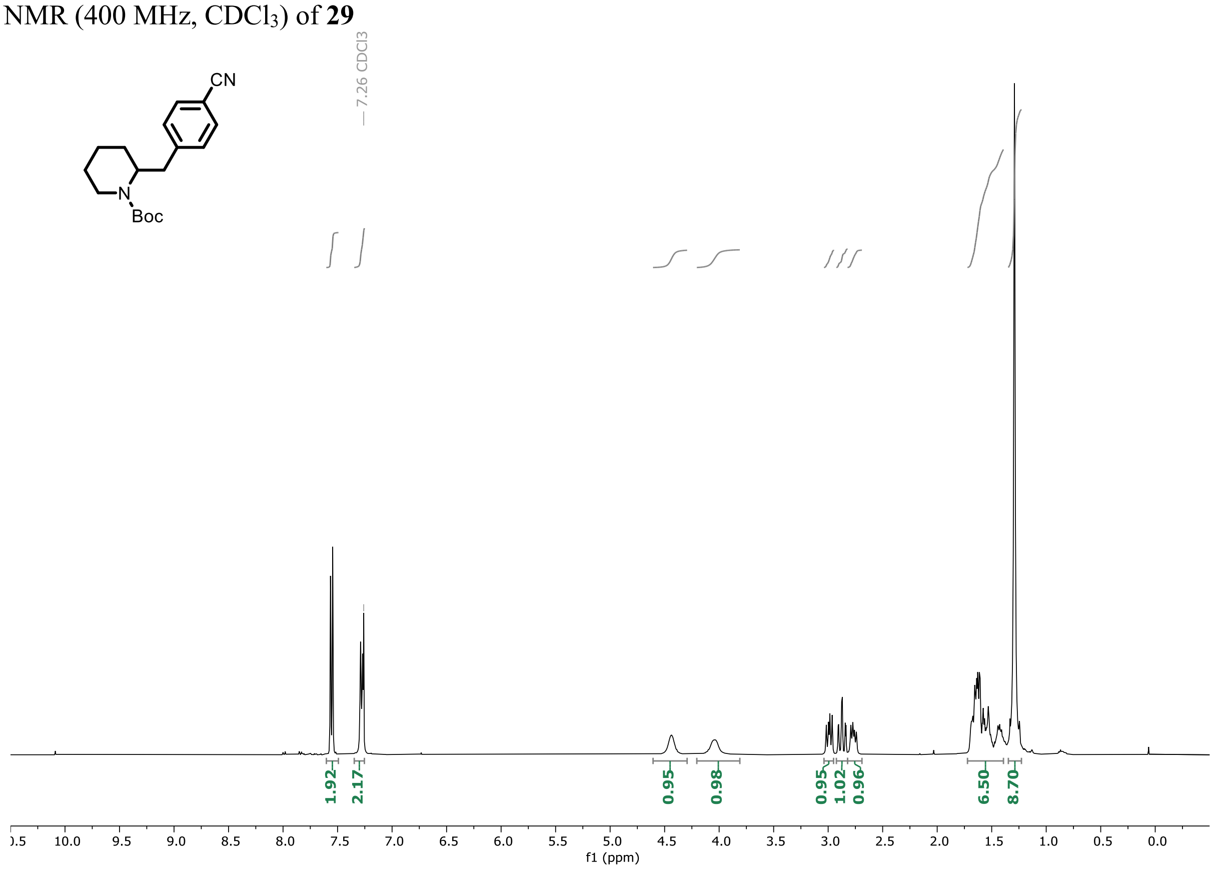

^{13}C NMR (101 MHz, $CDCl_3$) of **29**

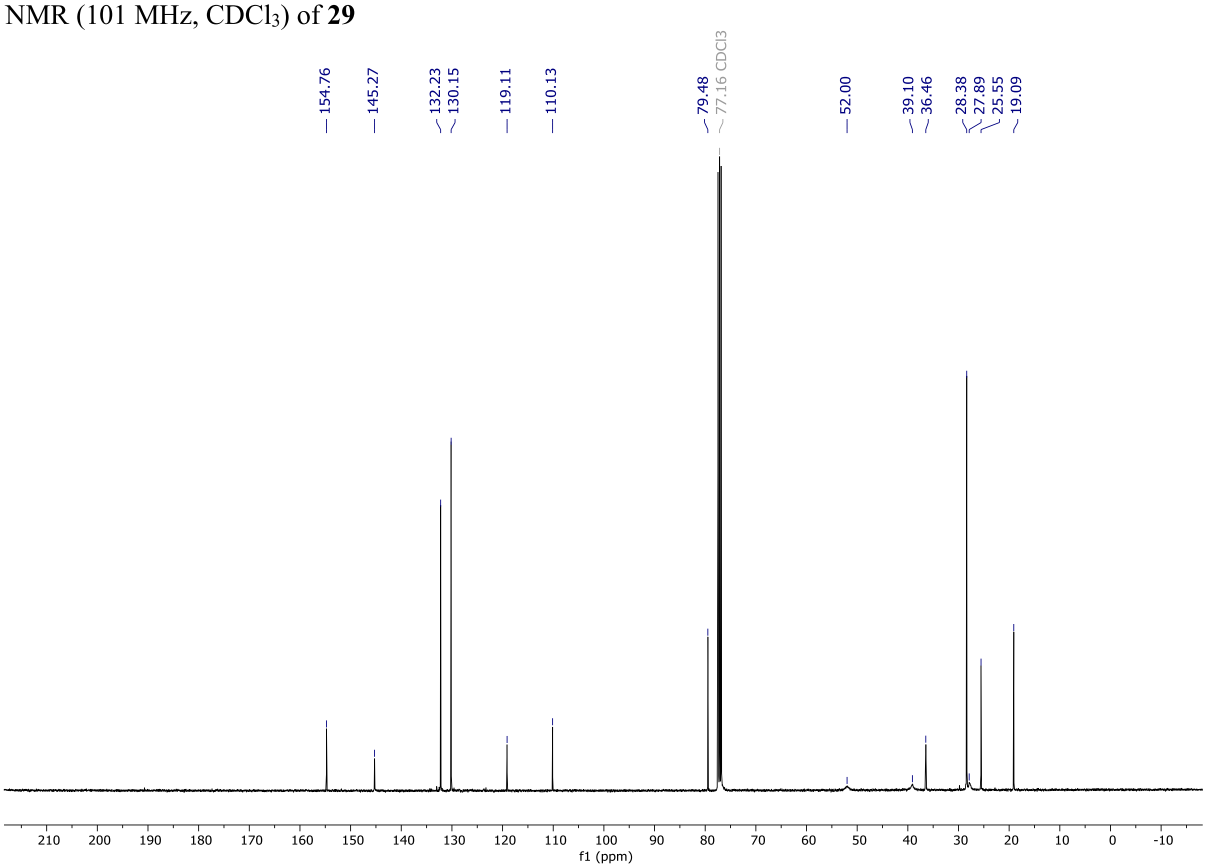

^{1}H NMR (400 MHz, $CDCl_3$) of **30**

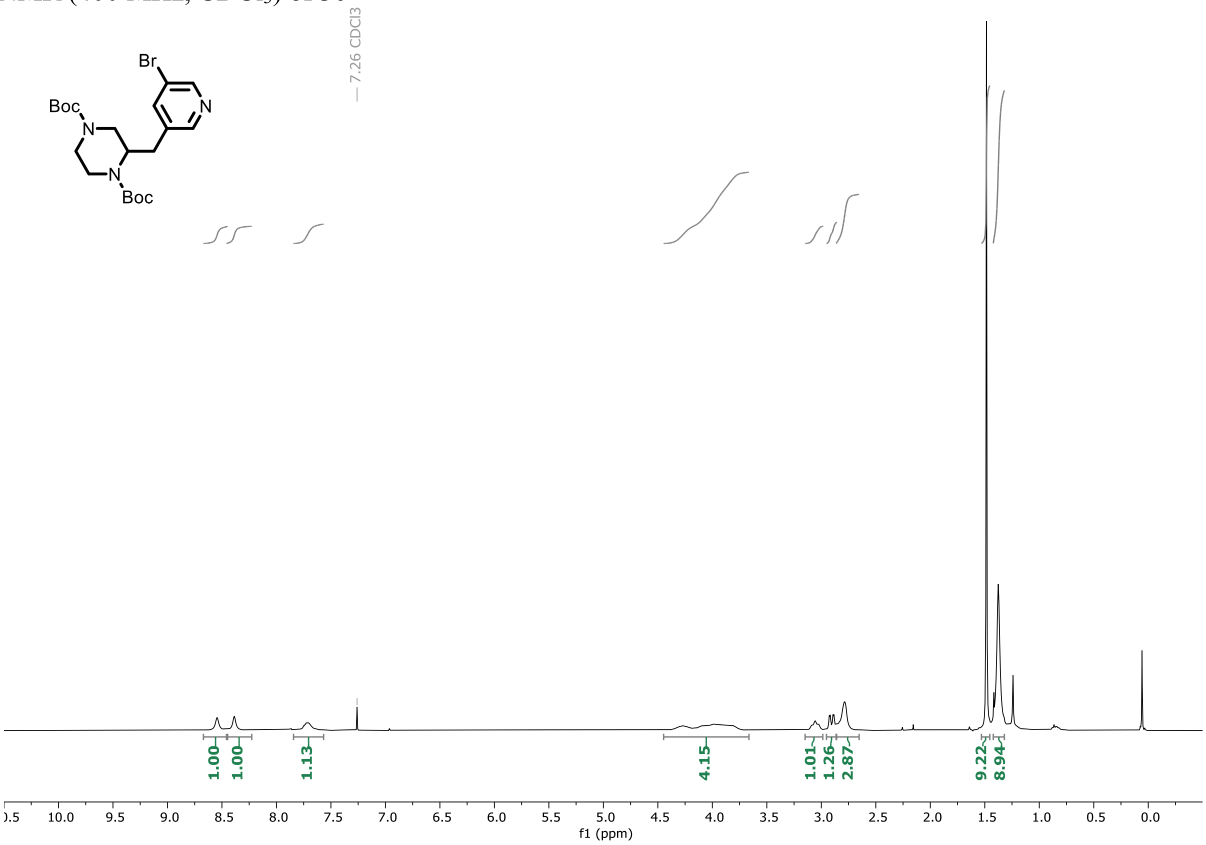

^{13}C NMR (101 MHz, $CDCl_3$) of **30**

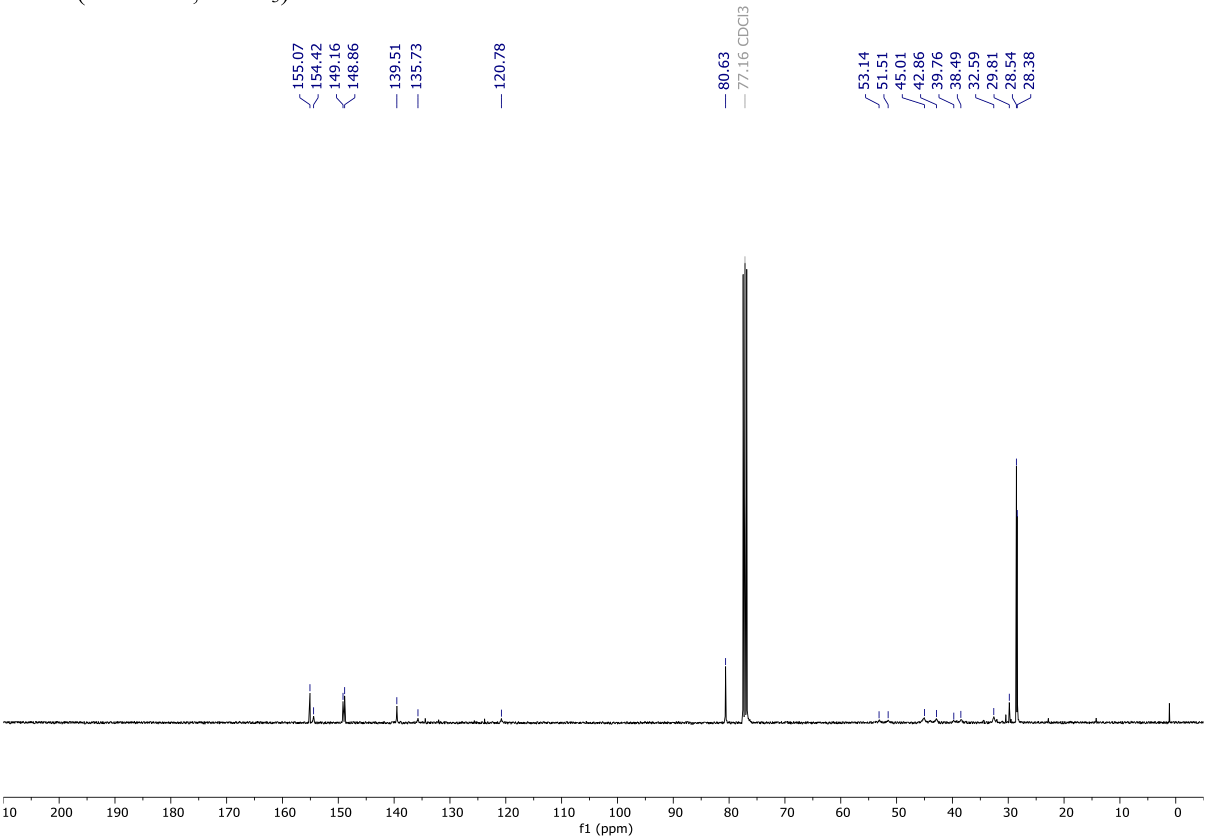

^{1}H NMR (400 MHz, $CDCl_3$) of **31**

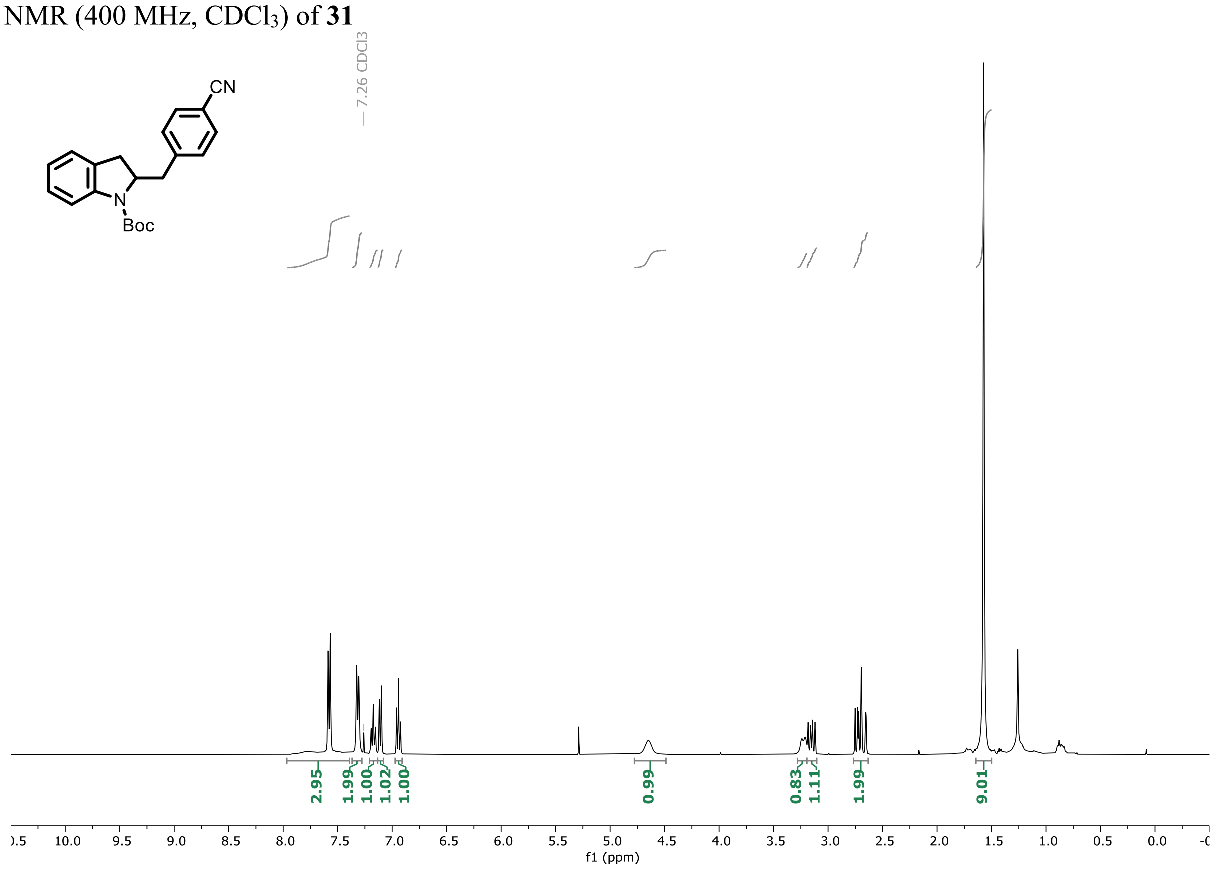

^{13}C NMR (101 MHz, $CDCl_3$) of **31**

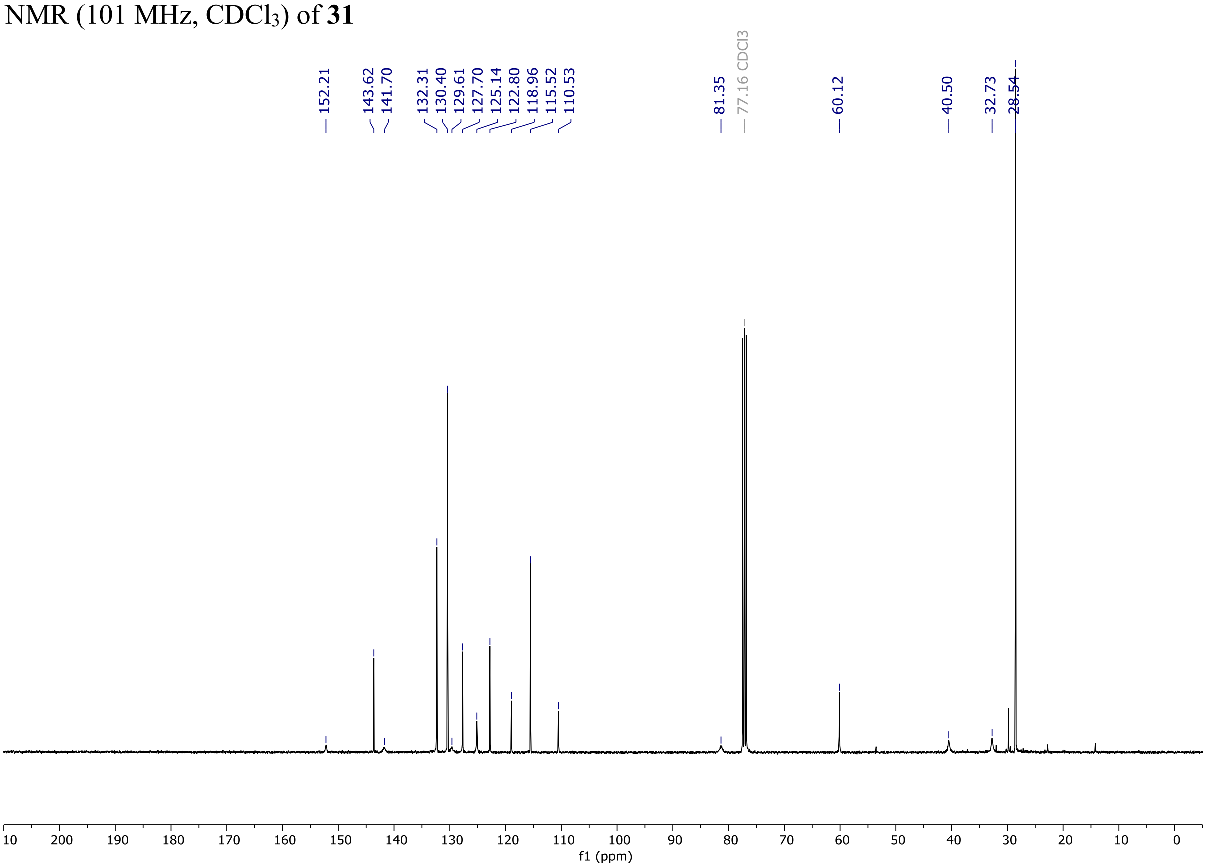

^{1}H NMR (400 MHz, $CDCl_3$) of **32**

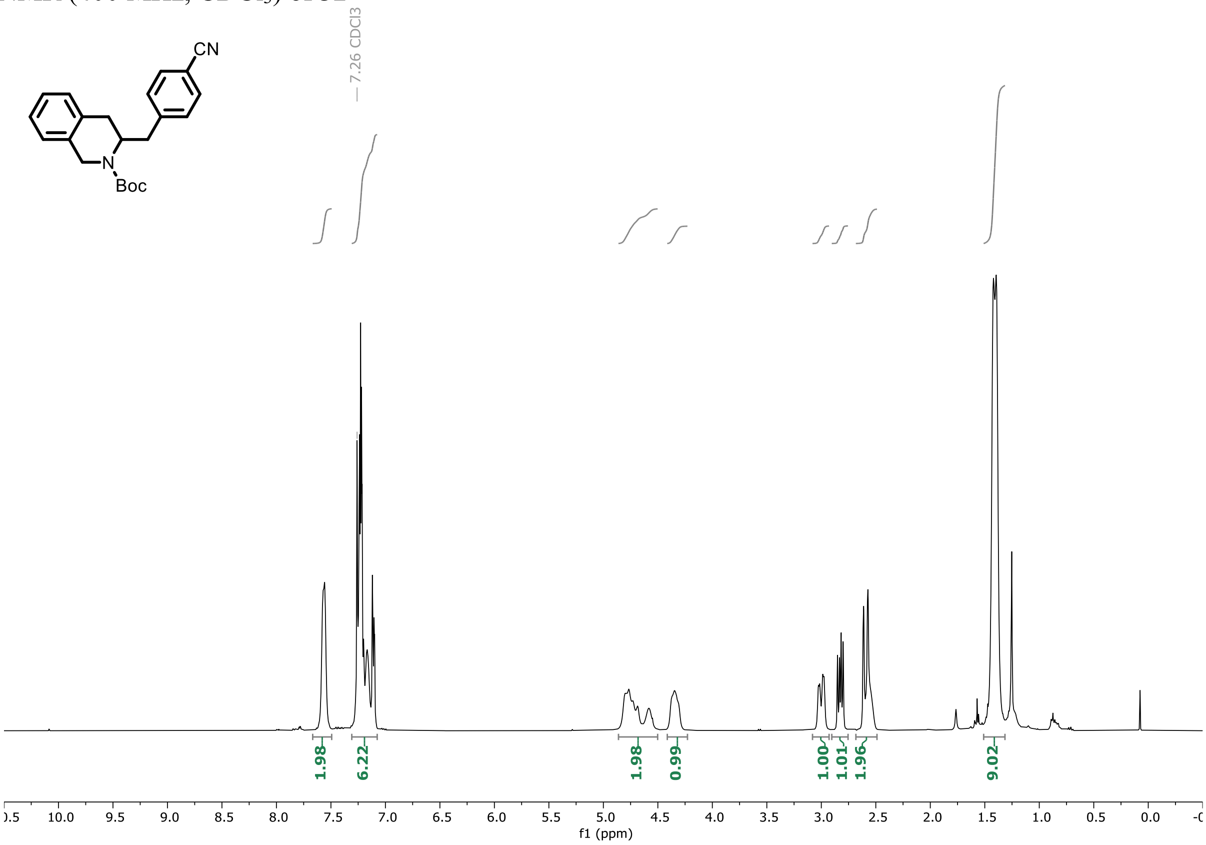

^{13}C NMR (101 MHz, $CDCl_3$) of **32**

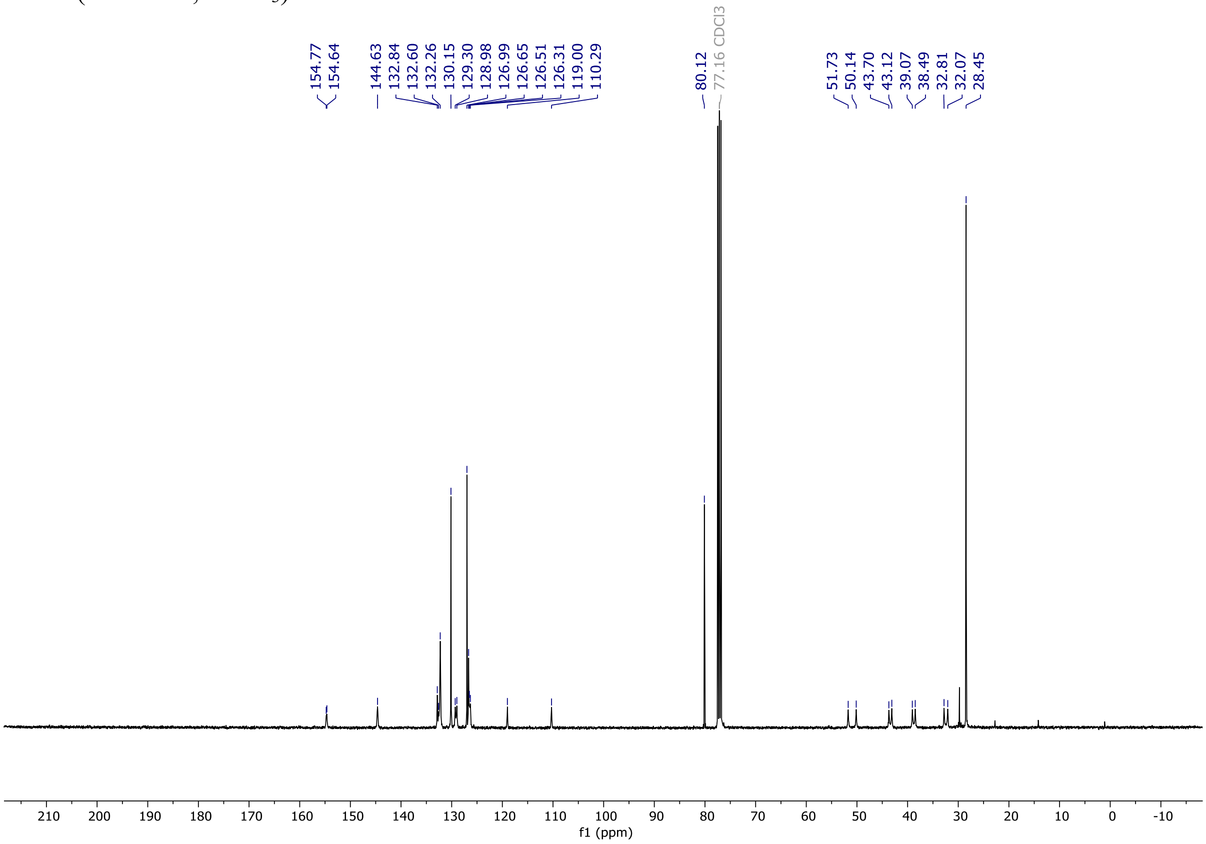

^{1}H NMR (400 MHz,CDCl$_3$) of **33**

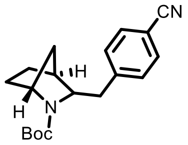

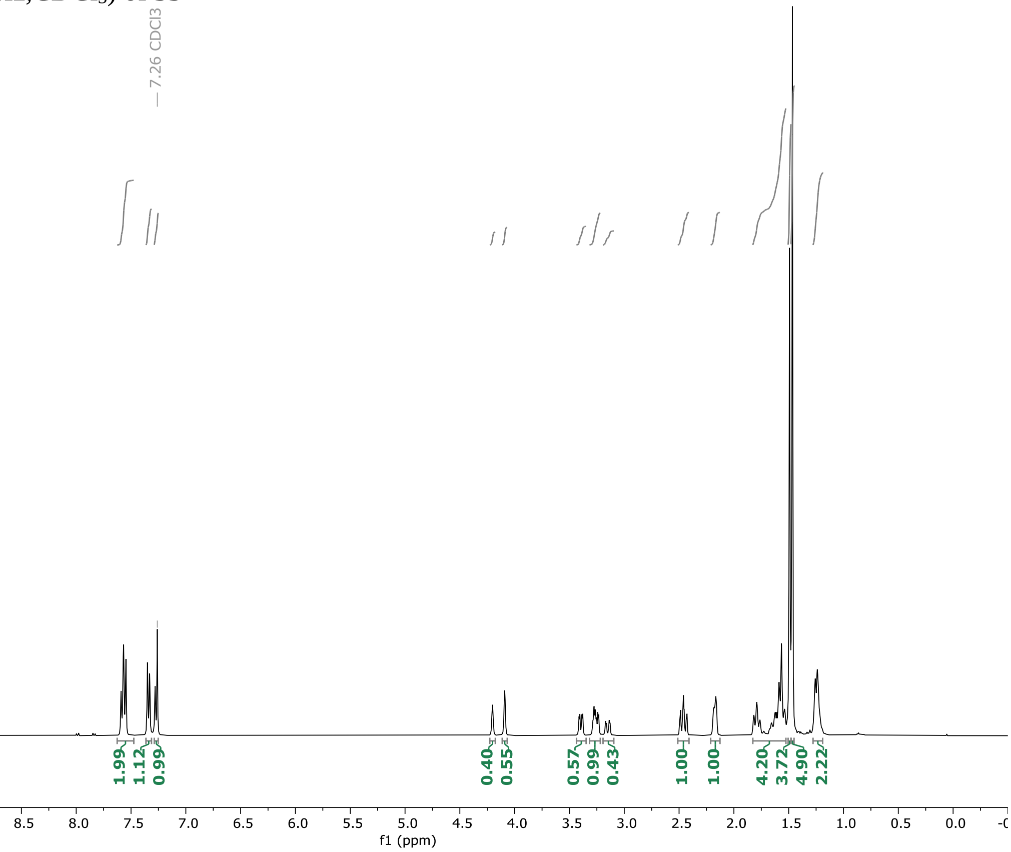

^{13}C NMR (101 MHz, CDCl$_3$) of **33**

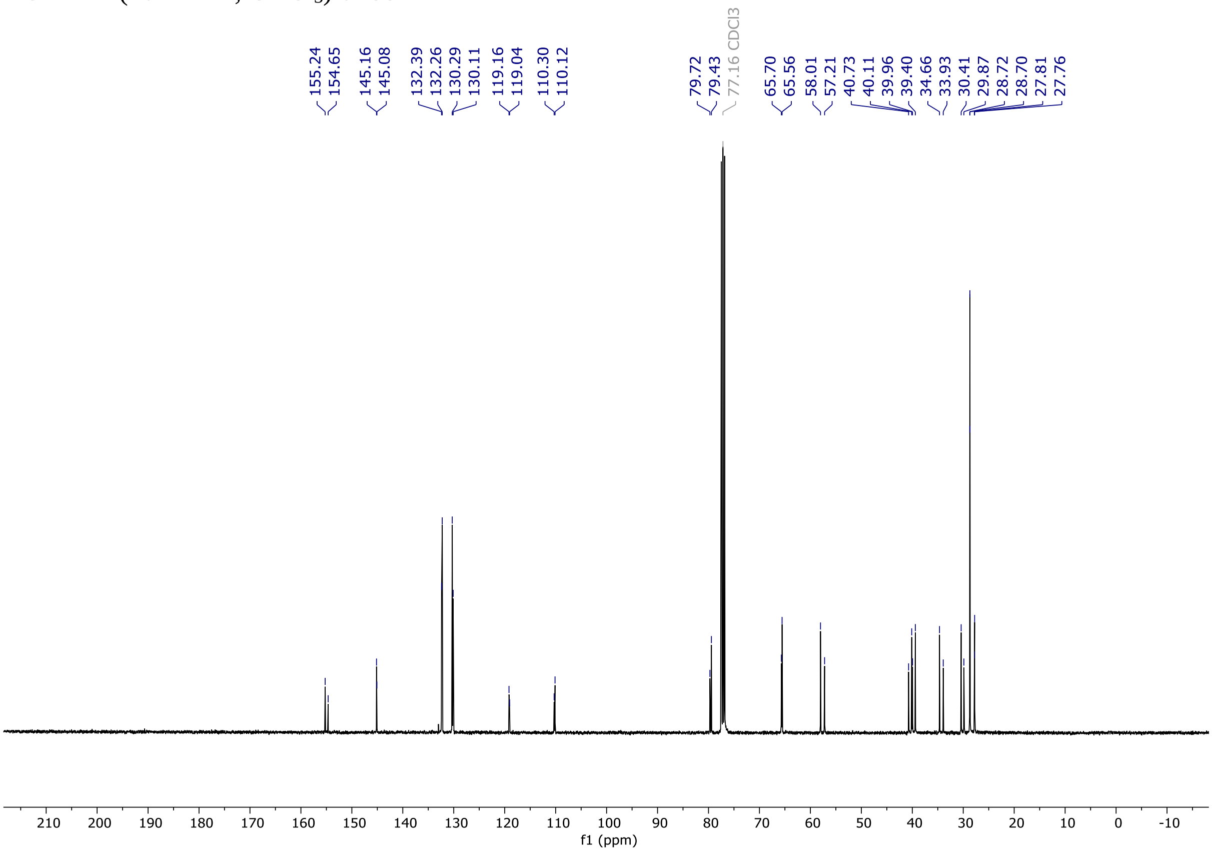

^{1}H NMR (500 MHz,$CDCl_3$) of **34**

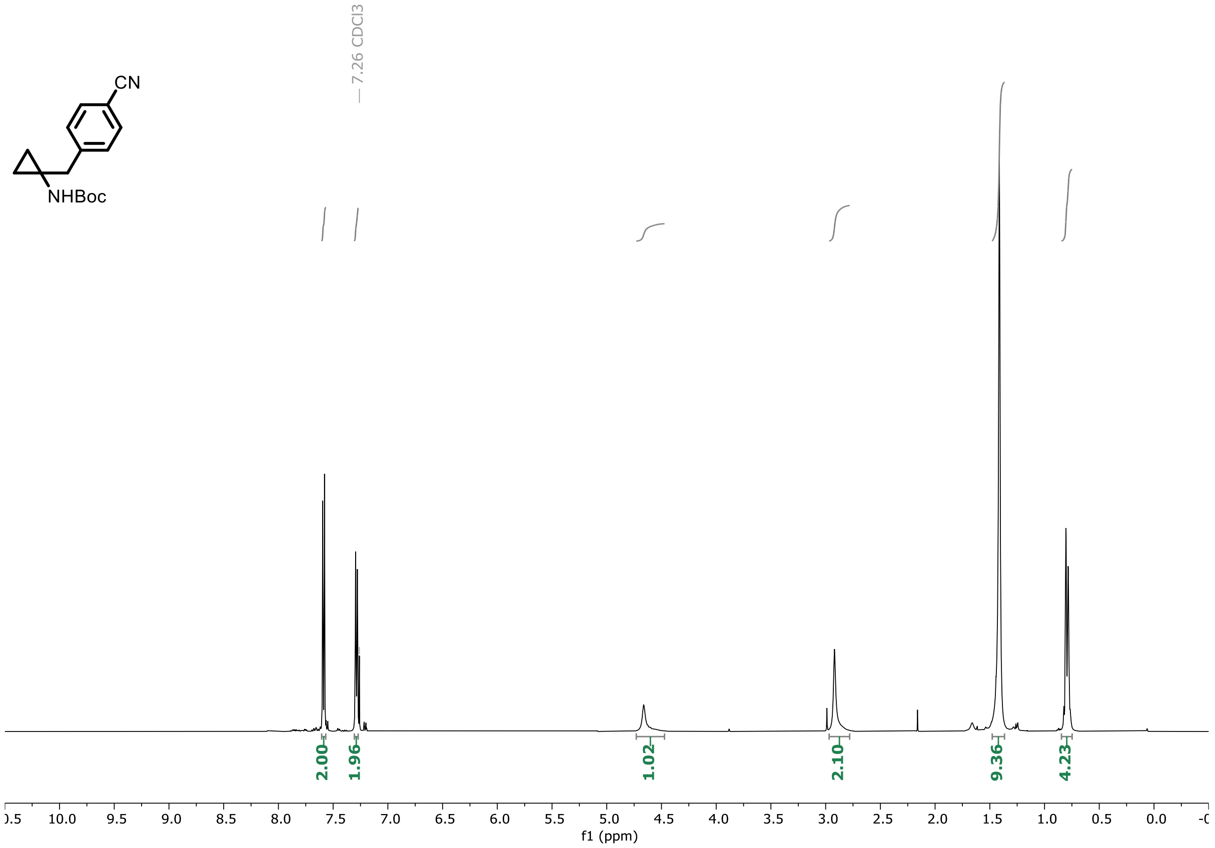

^{13}C NMR (126 MHz, $CDCl_3$) of **34**

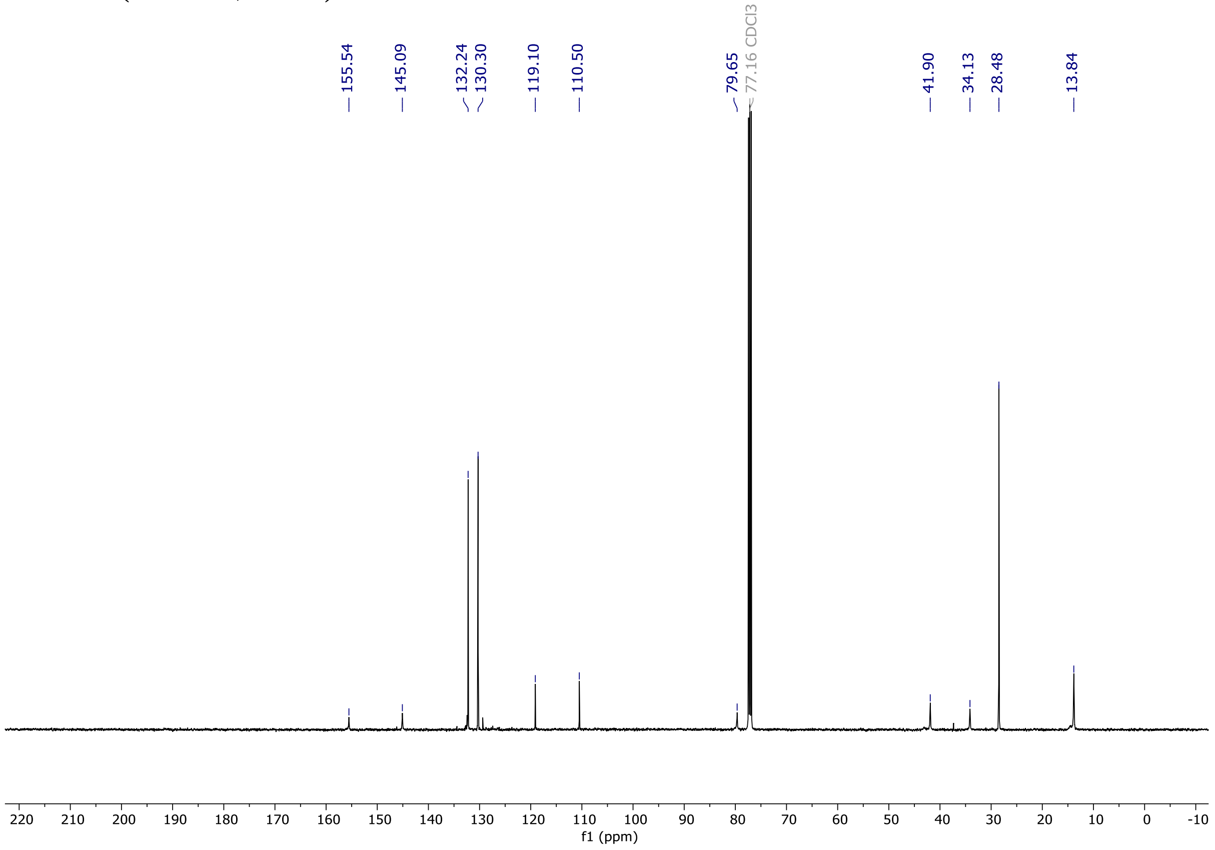

^1H NMR (400 MHz, $CDCl_3$) of **35**

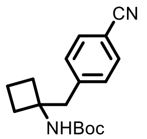

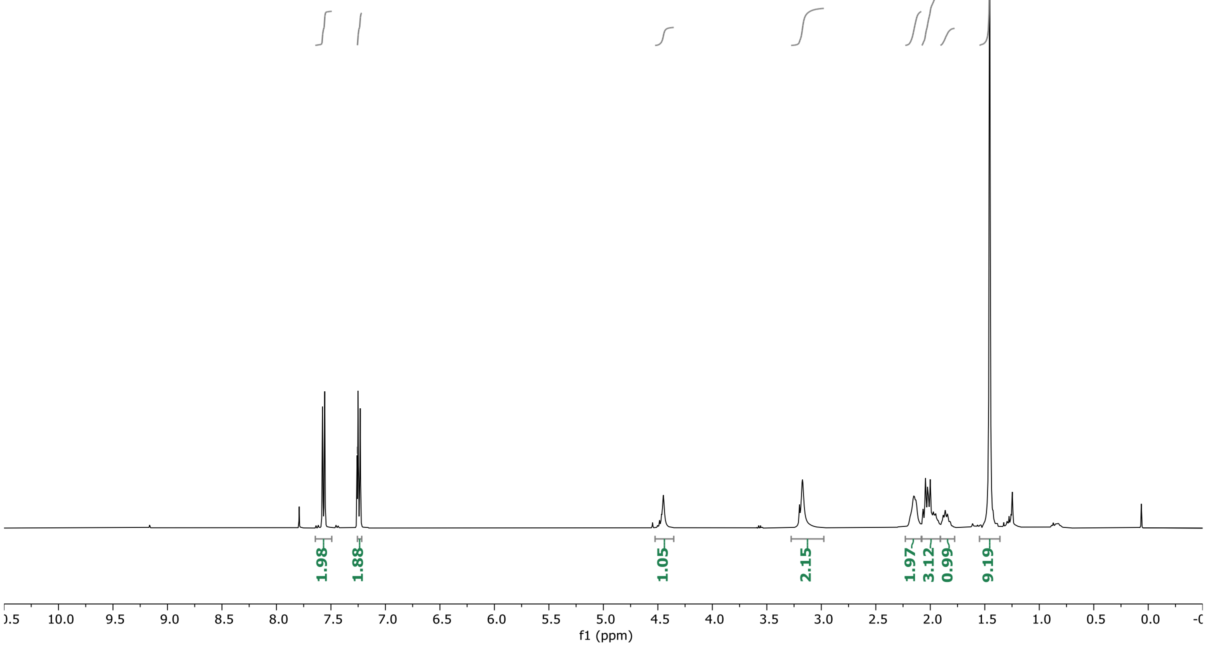

^{13}C NMR (101 MHz, $CDCl_3$) of **35**

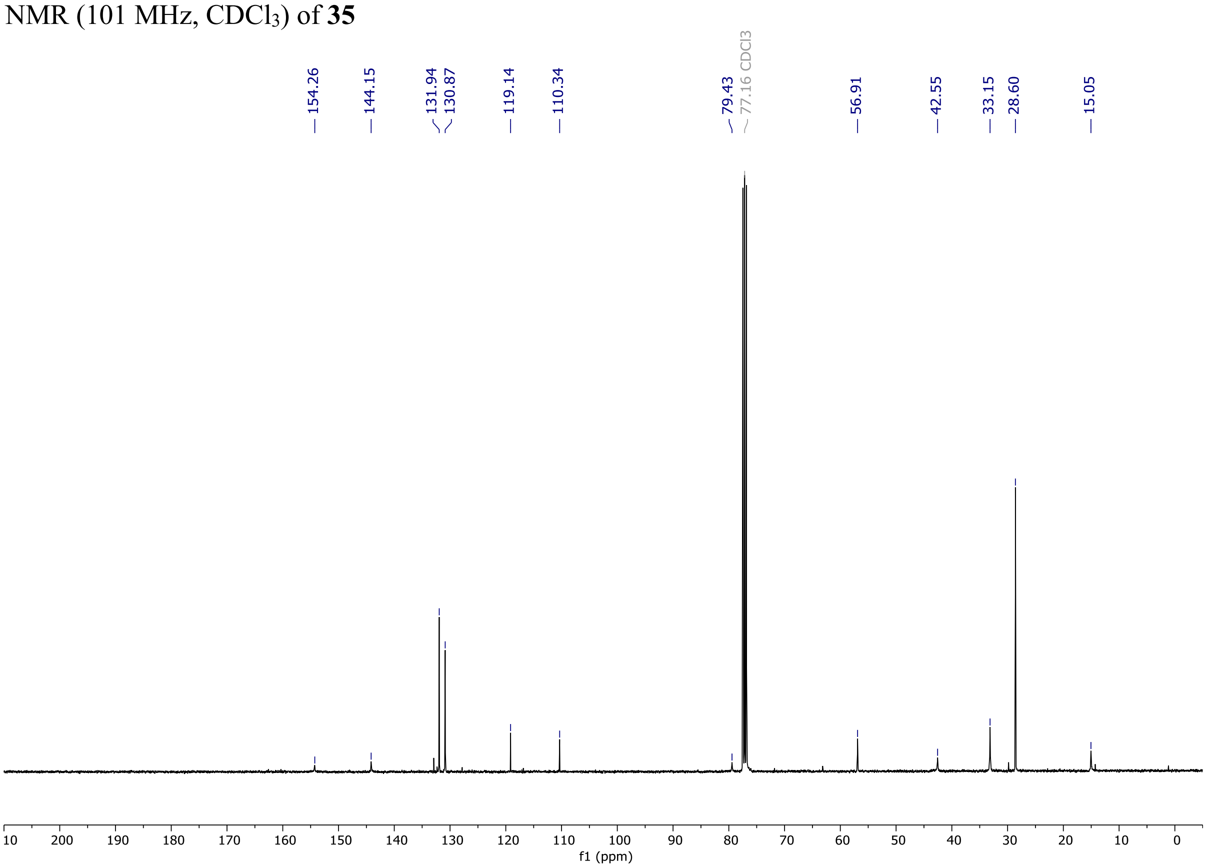

^1H NMR (400 MHz, $CDCl_3$) of **36**

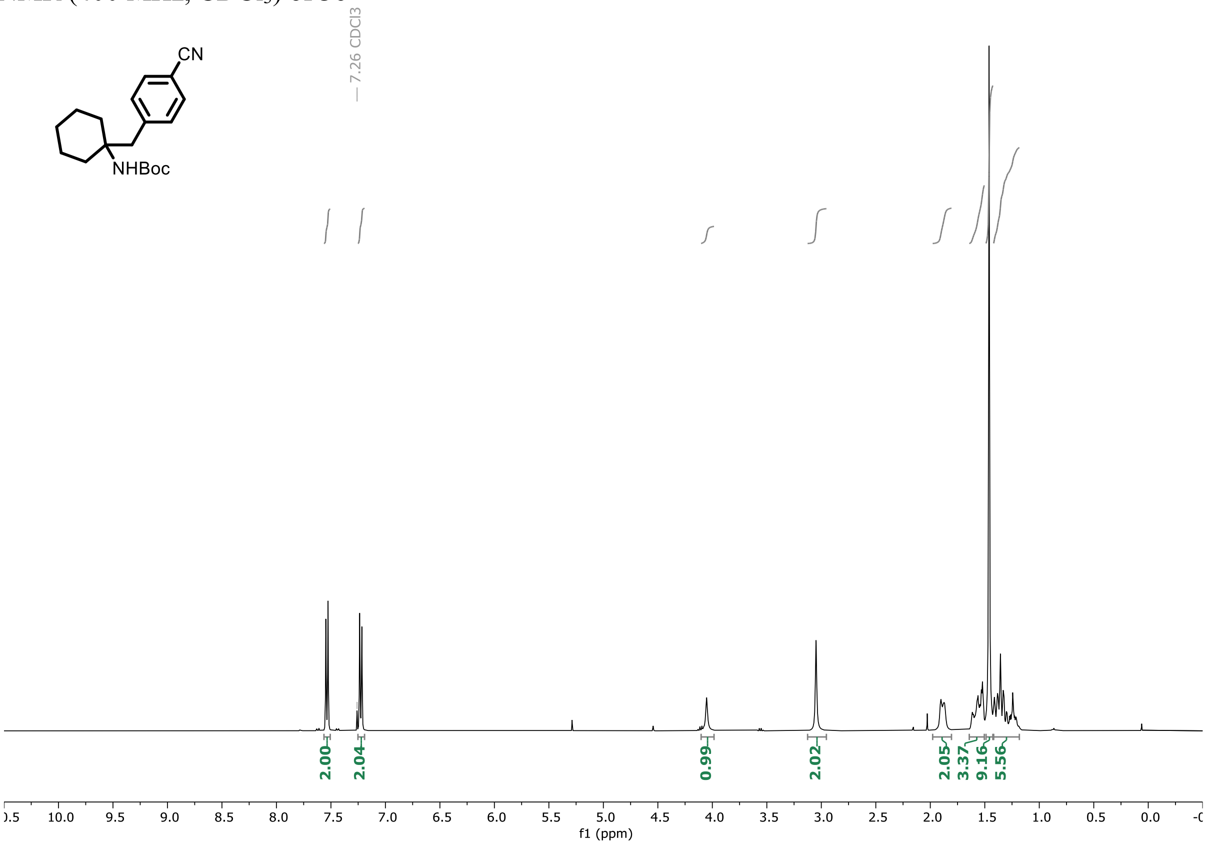

^{13}C NMR (101 MHz, $CDCl_3$) of **36**

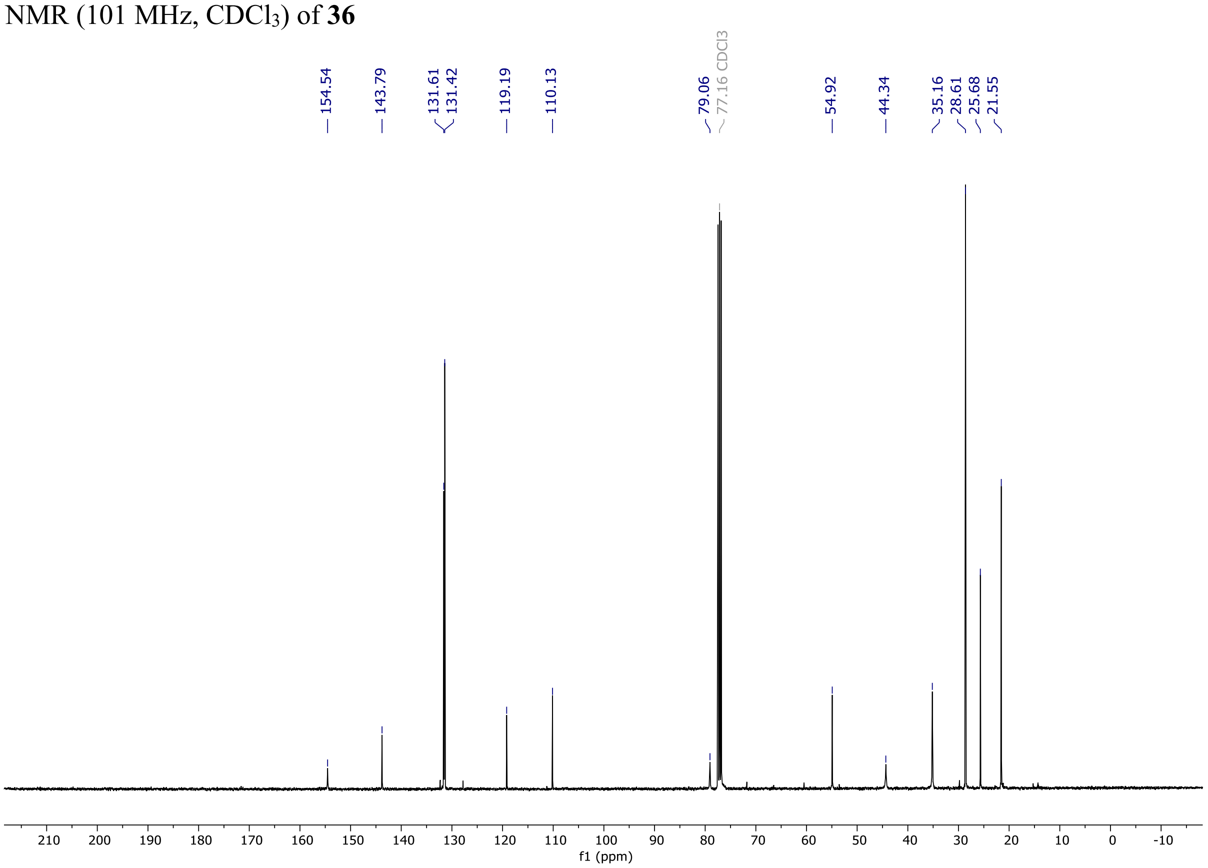

^{1}H NMR (500 MHz,CDCl$_3$) of **37**

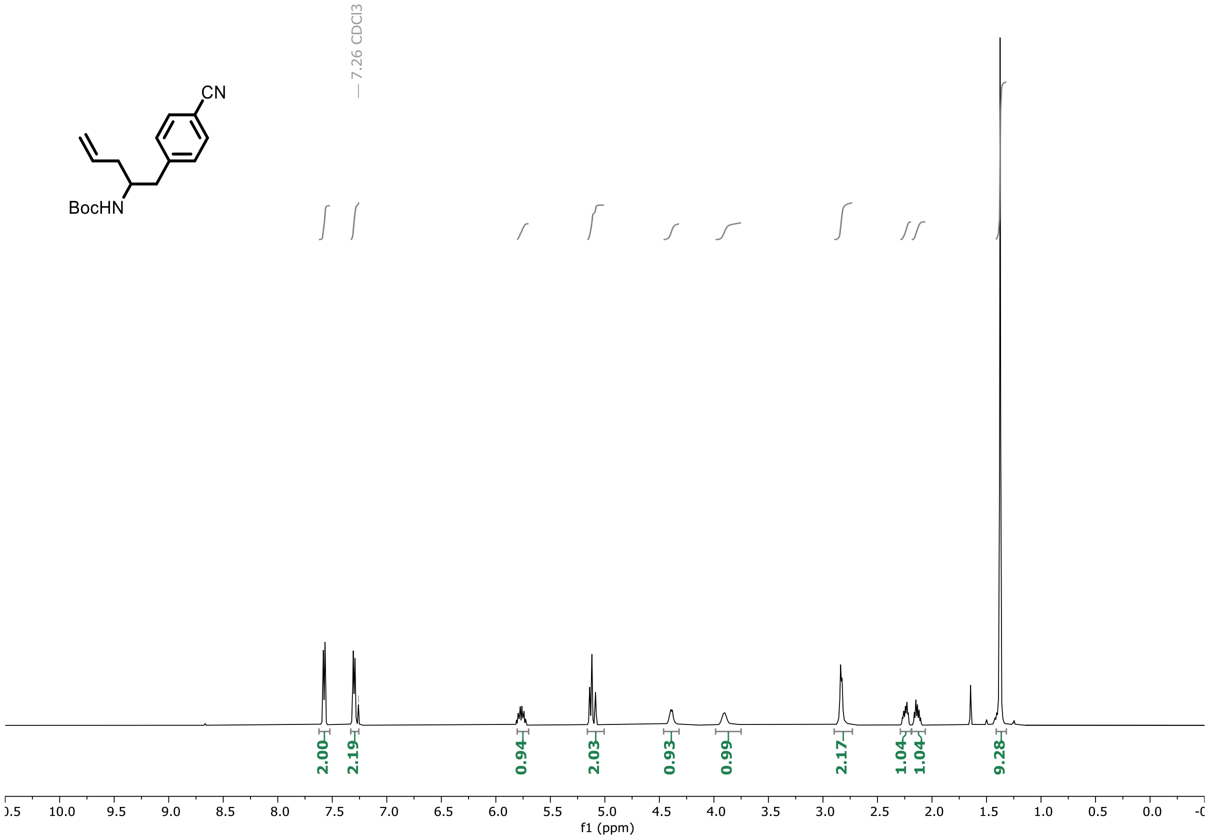

^{13}C NMR (126 MHz, CDCl$_3$) of **37**

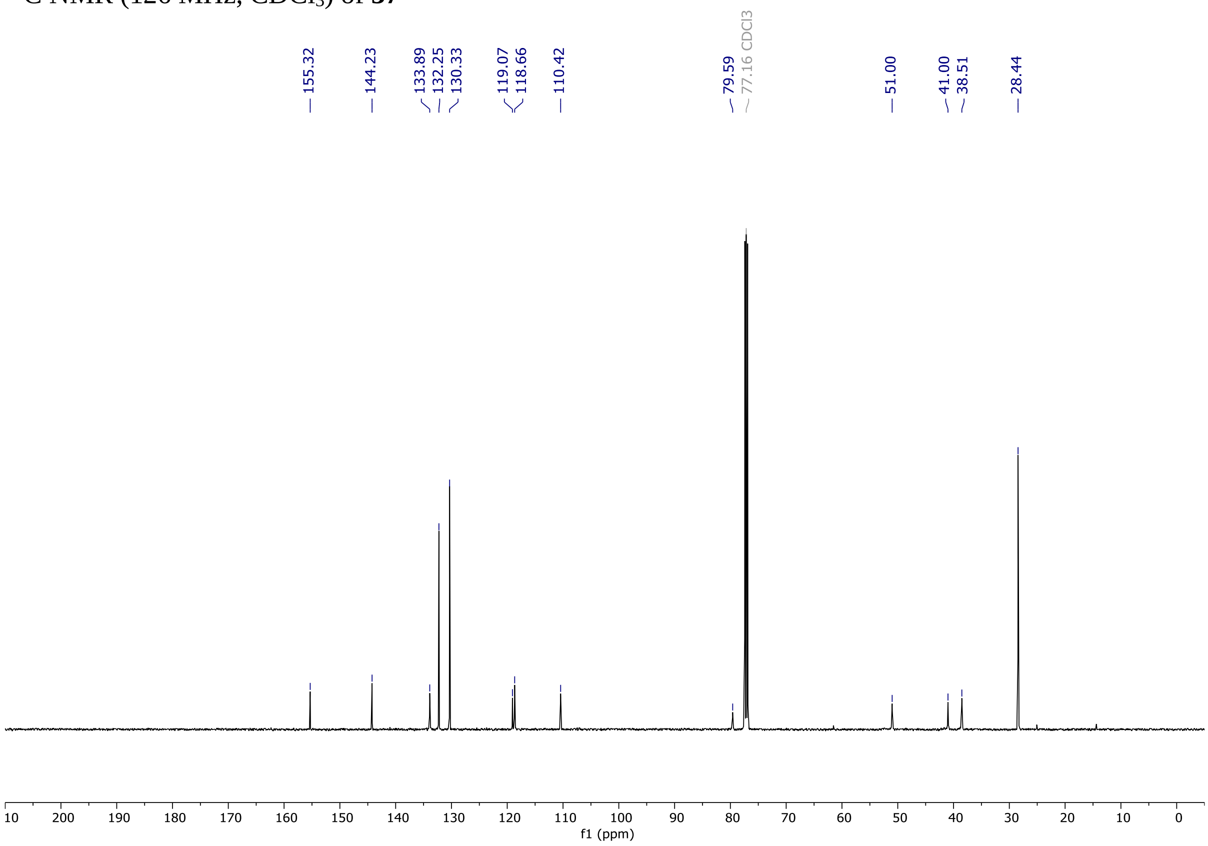

^{1}H NMR (400 MHz, $CDCl_3$) of **38**

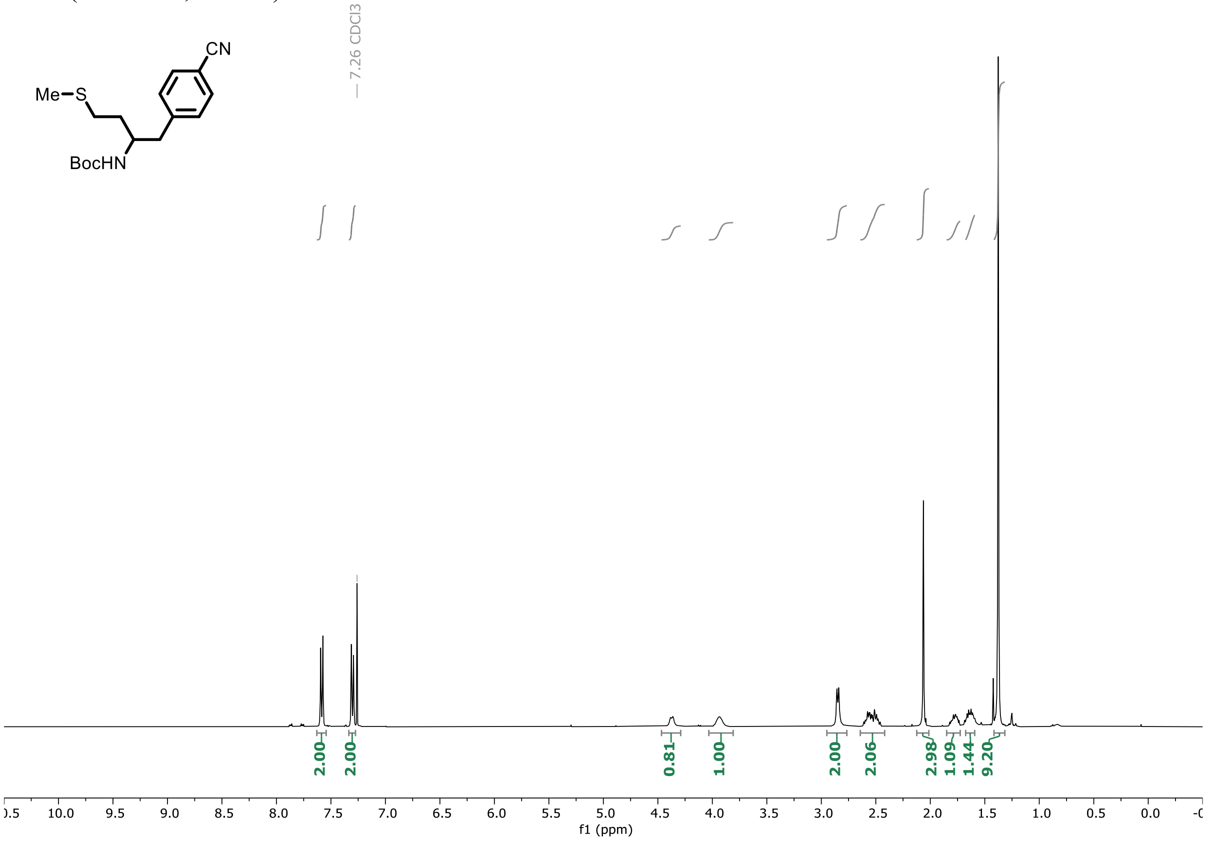

^{13}C NMR (101 MHz, $CDCl_3$) of **38**

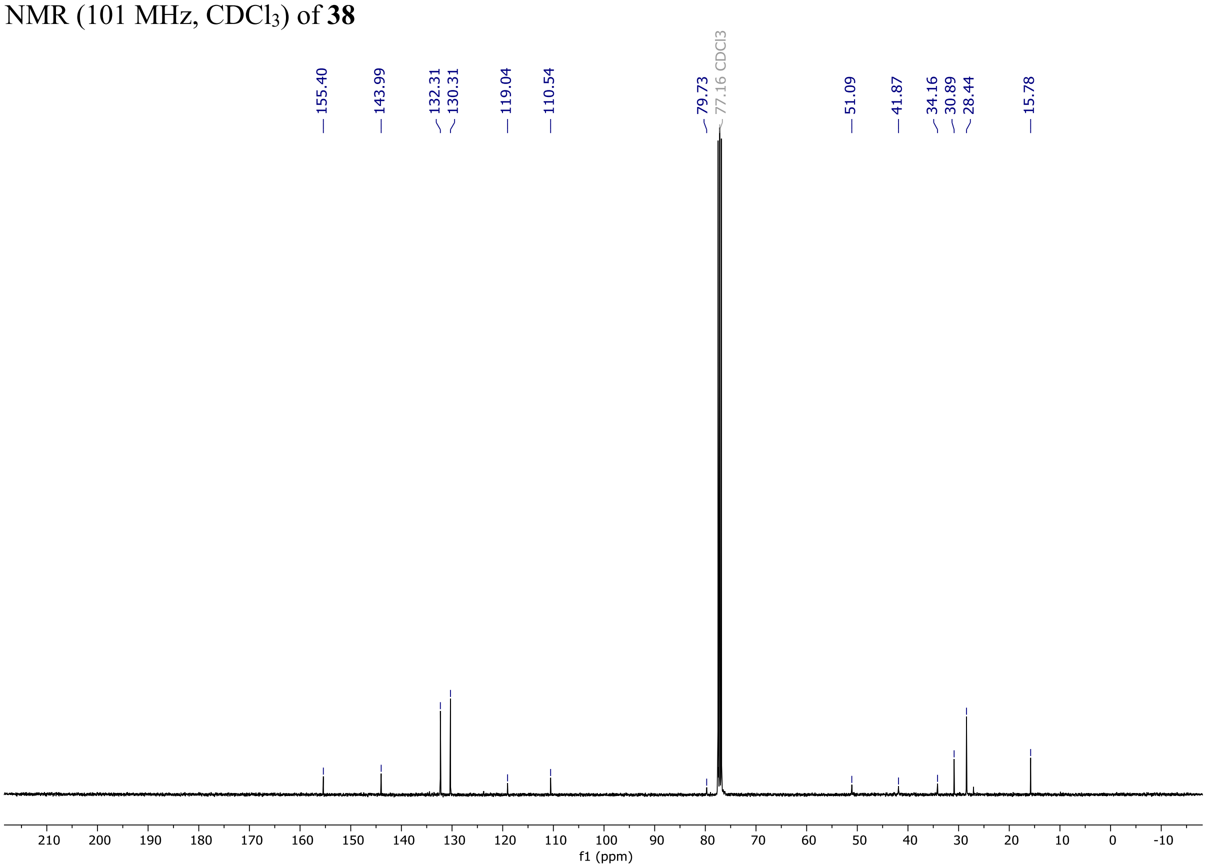

^{1}H NMR (400 MHz,CDCl$_3$) of **39**

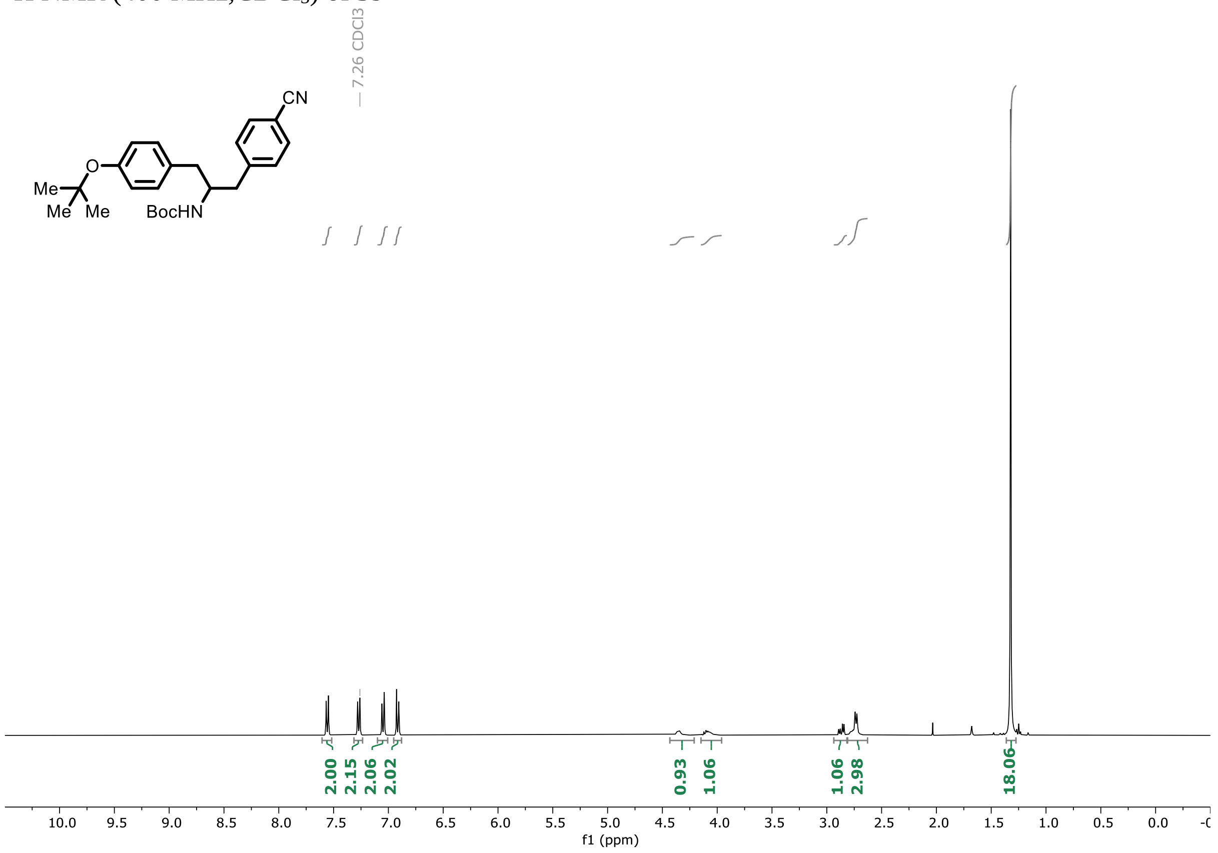

^{13}C NMR (101 MHz, CDCl$_3$) of **39**

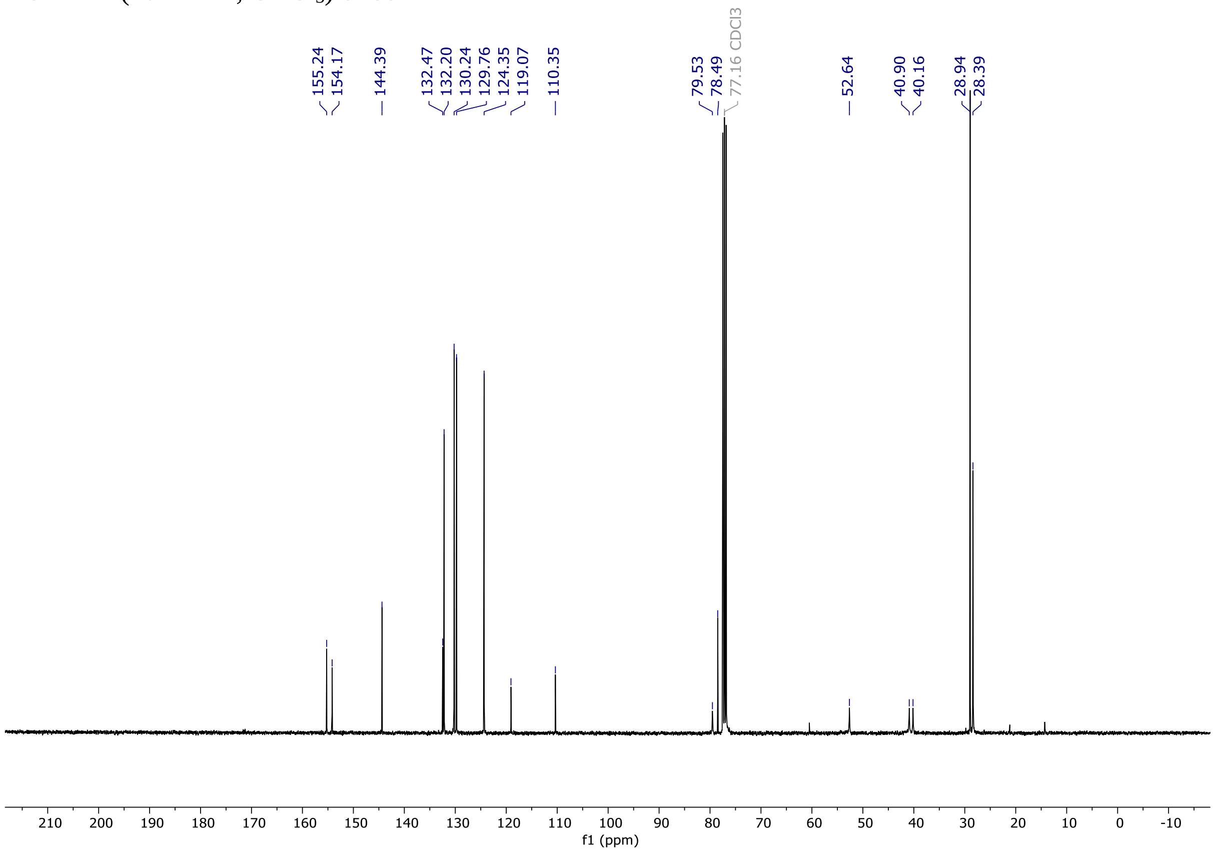

^{1}H NMR (400 MHz, $CDCl_3$) of **40**

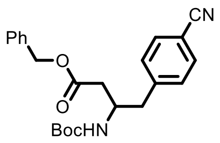

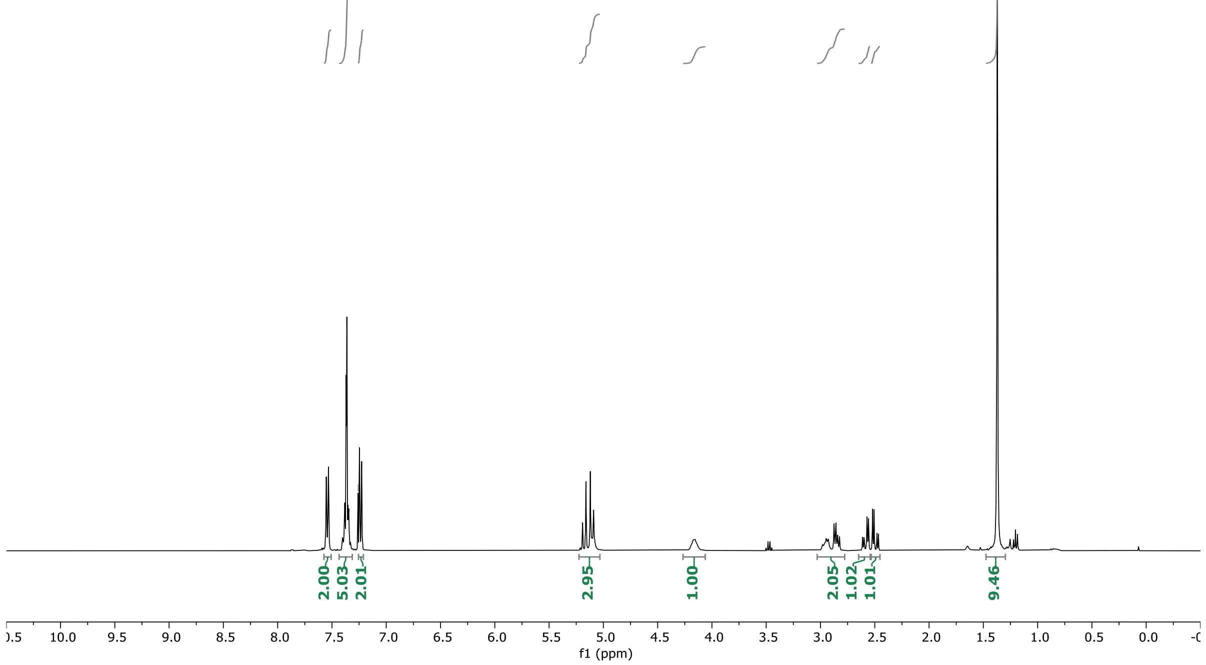

^{13}C NMR (101 MHz, $CDCl_3$) of **40**

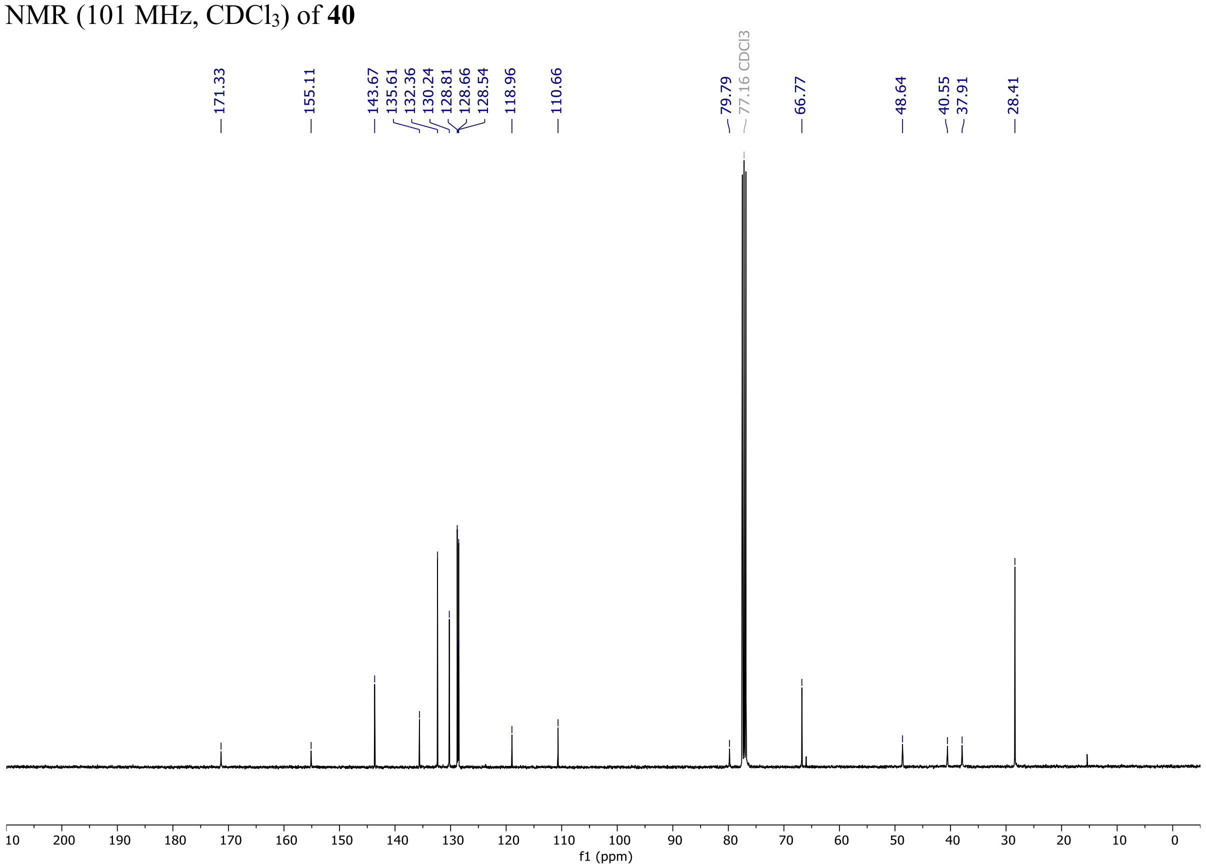

^{1}H NMR (400 MHz,CDCl$_3$) of **41**

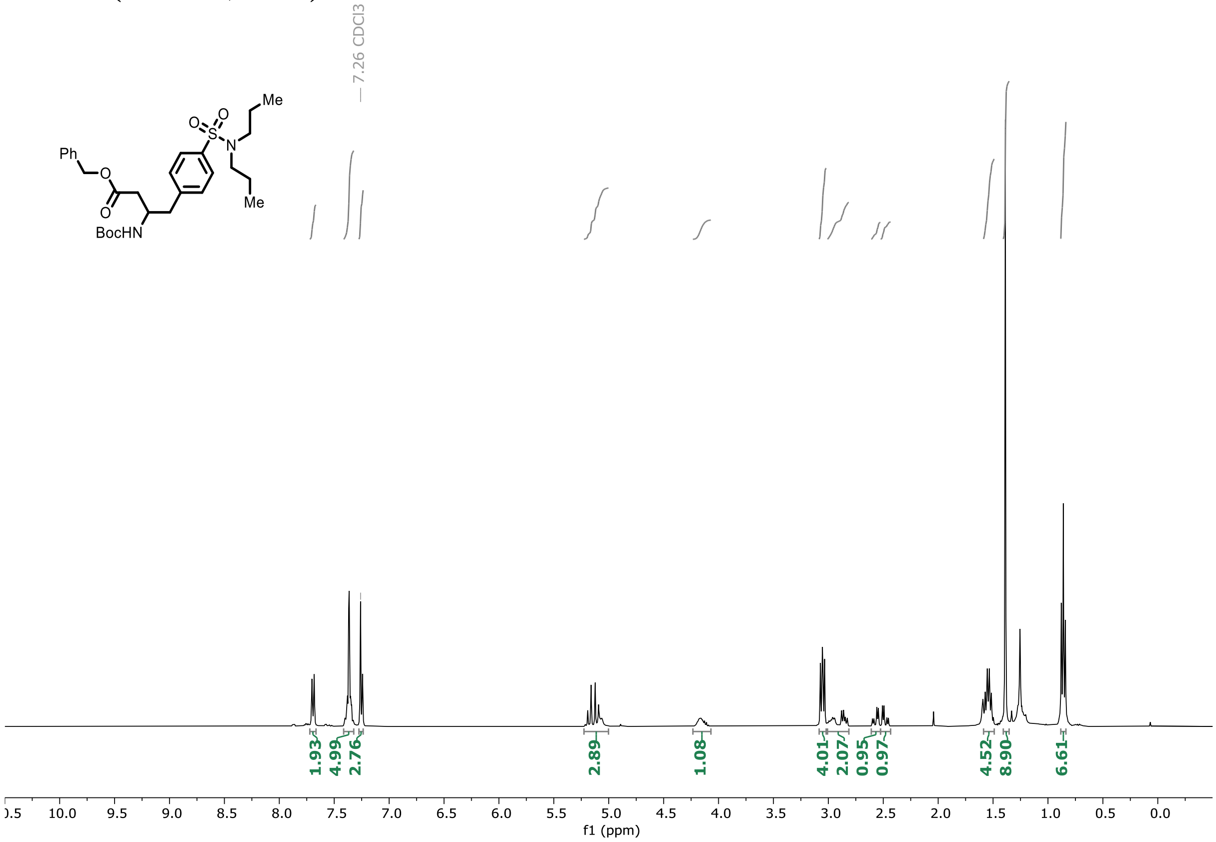

^{13}C NMR (101 MHz, CDCl$_3$) of **41**

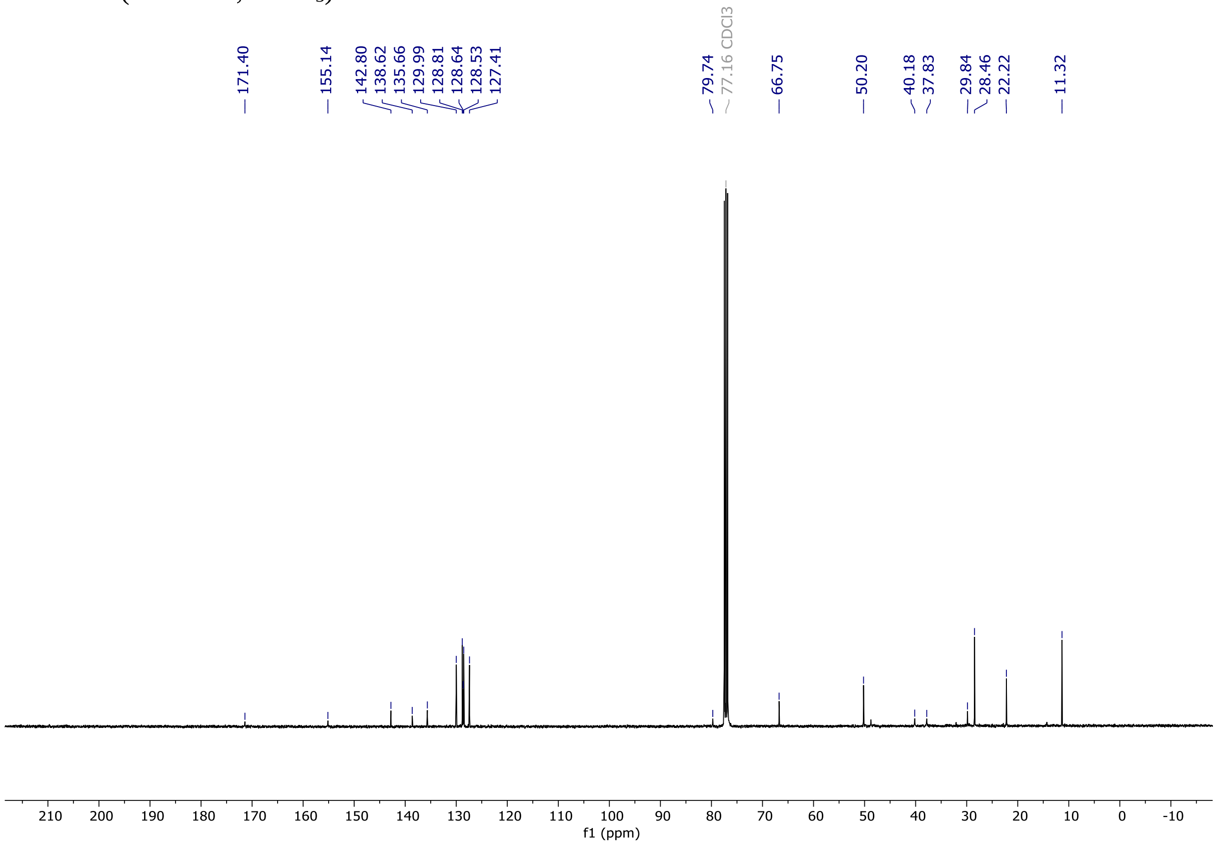

^{1}H NMR (400 MHz, $CDCl_3$) of **42**

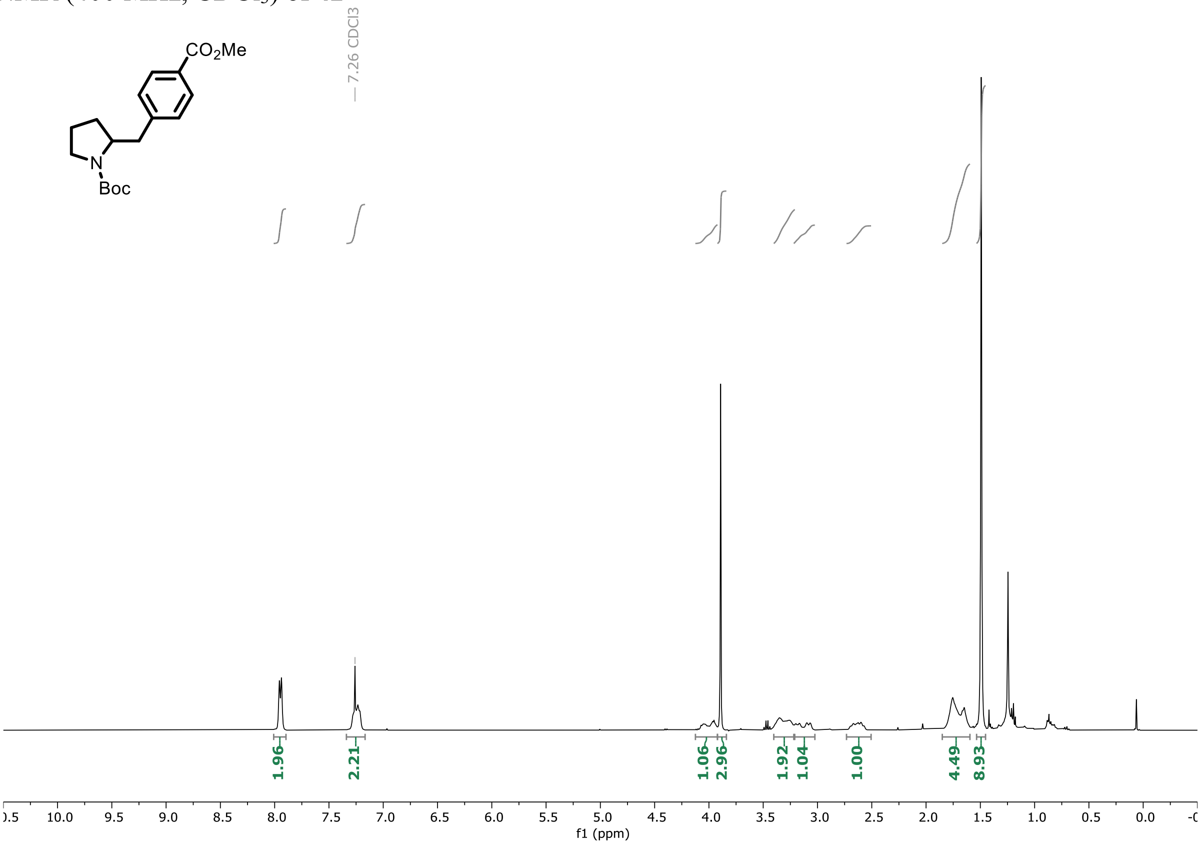

^{13}C NMR (101 MHz, $CDCl_3$) of **42**

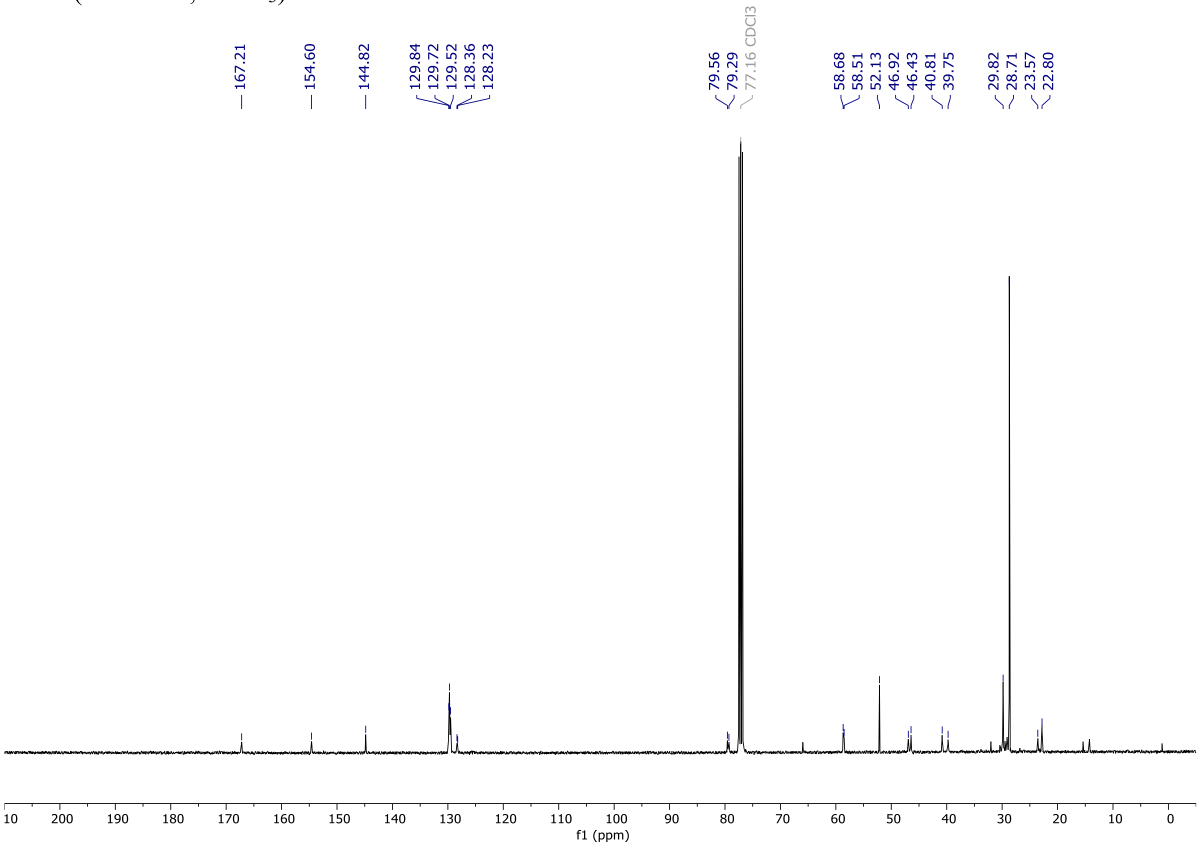

^{1}H NMR (400 MHz, $CDCl_3$) of **43**

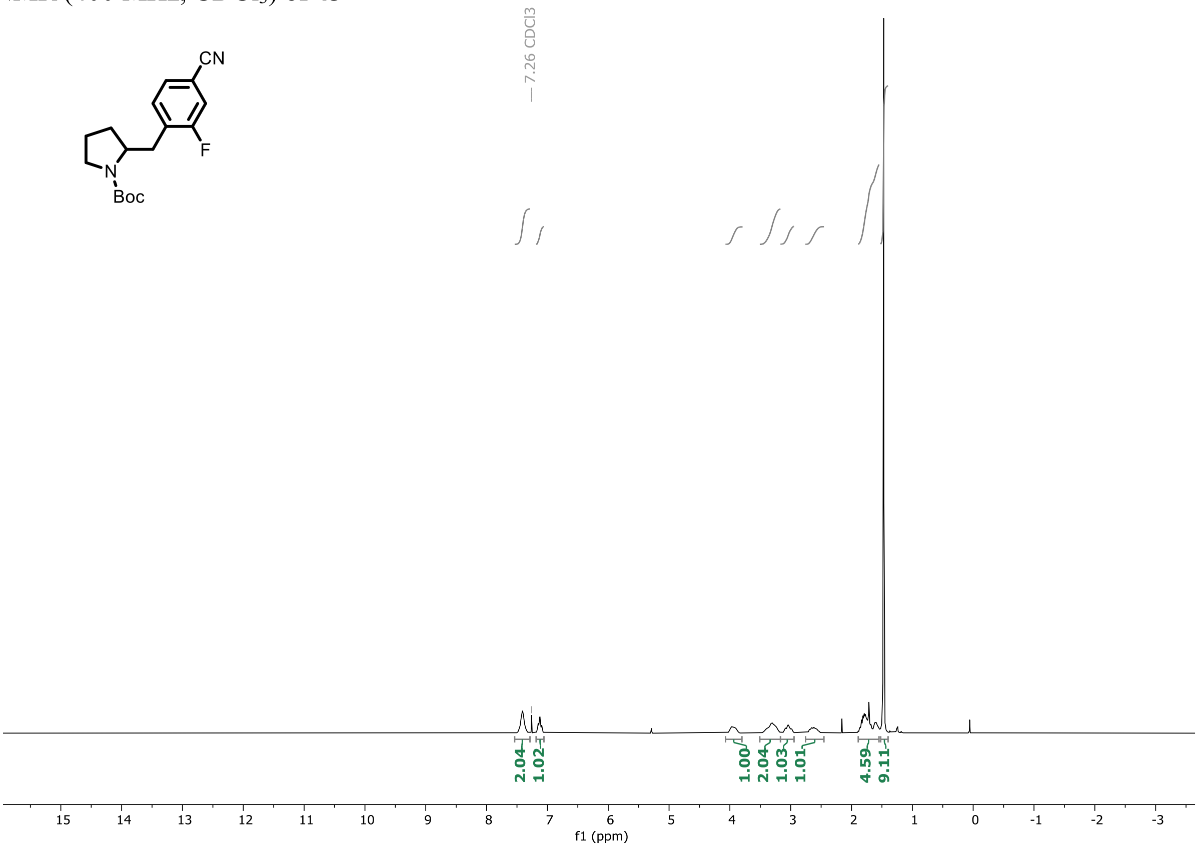

^{13}C NMR (101 MHz, $CDCl_3$) of **43**

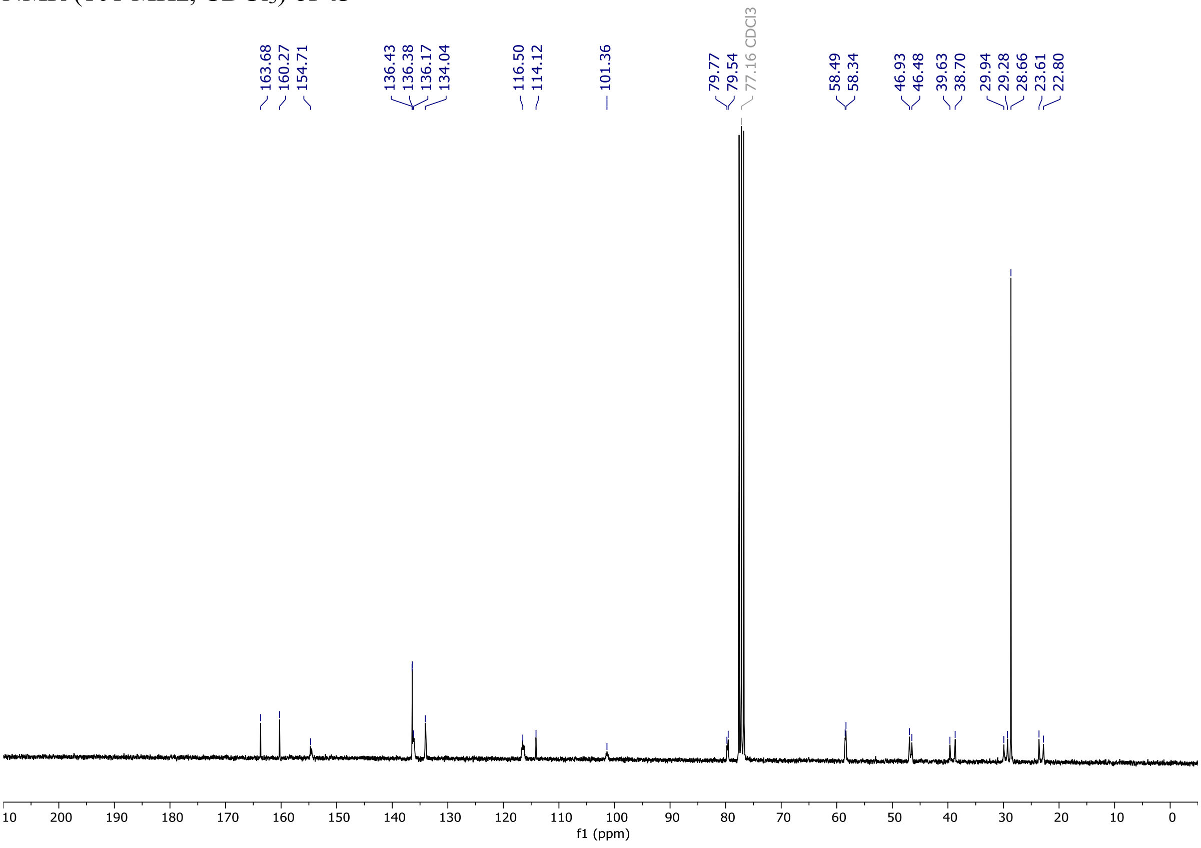

^{19}F NMR (282 MHz, $CDCl_3$) of **43**

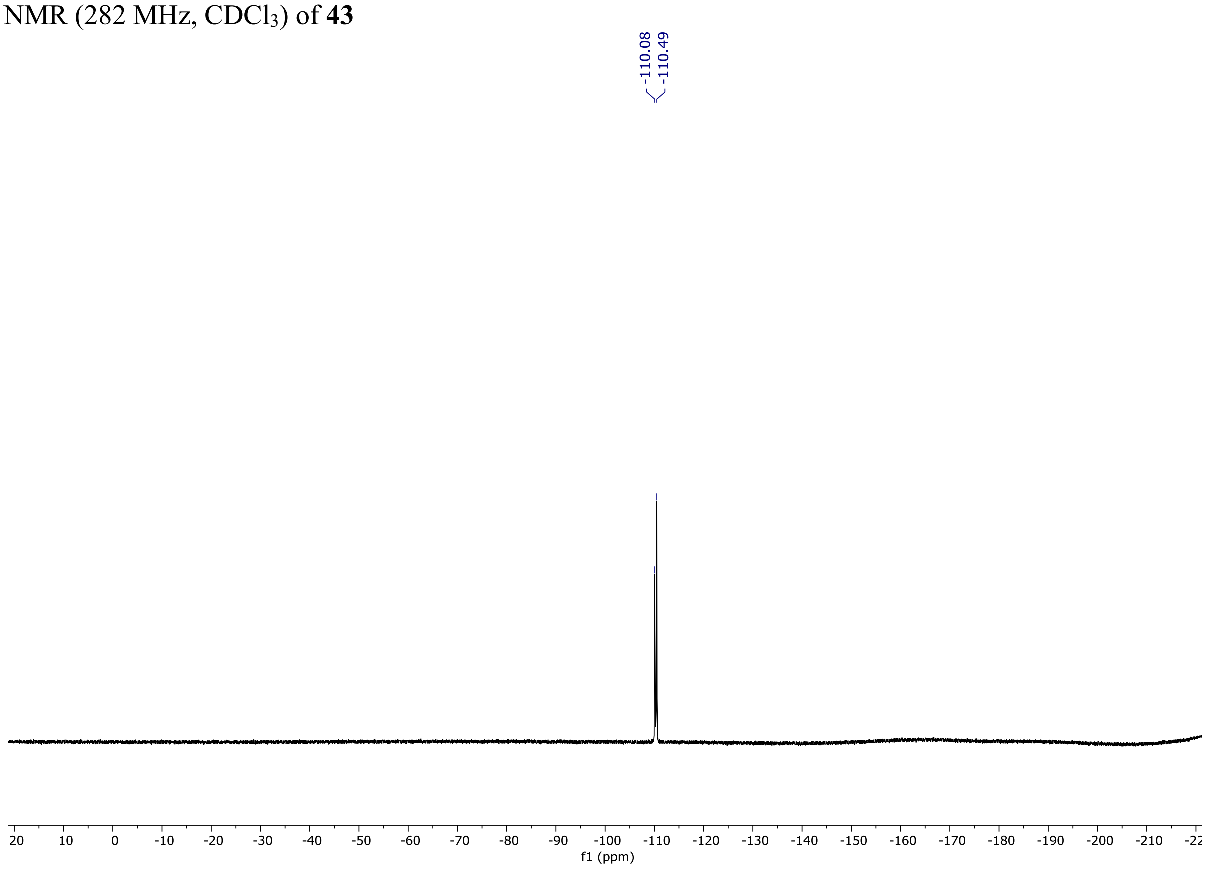

^{1}H NMR (400 MHz, $CDCl_3$) of **44**

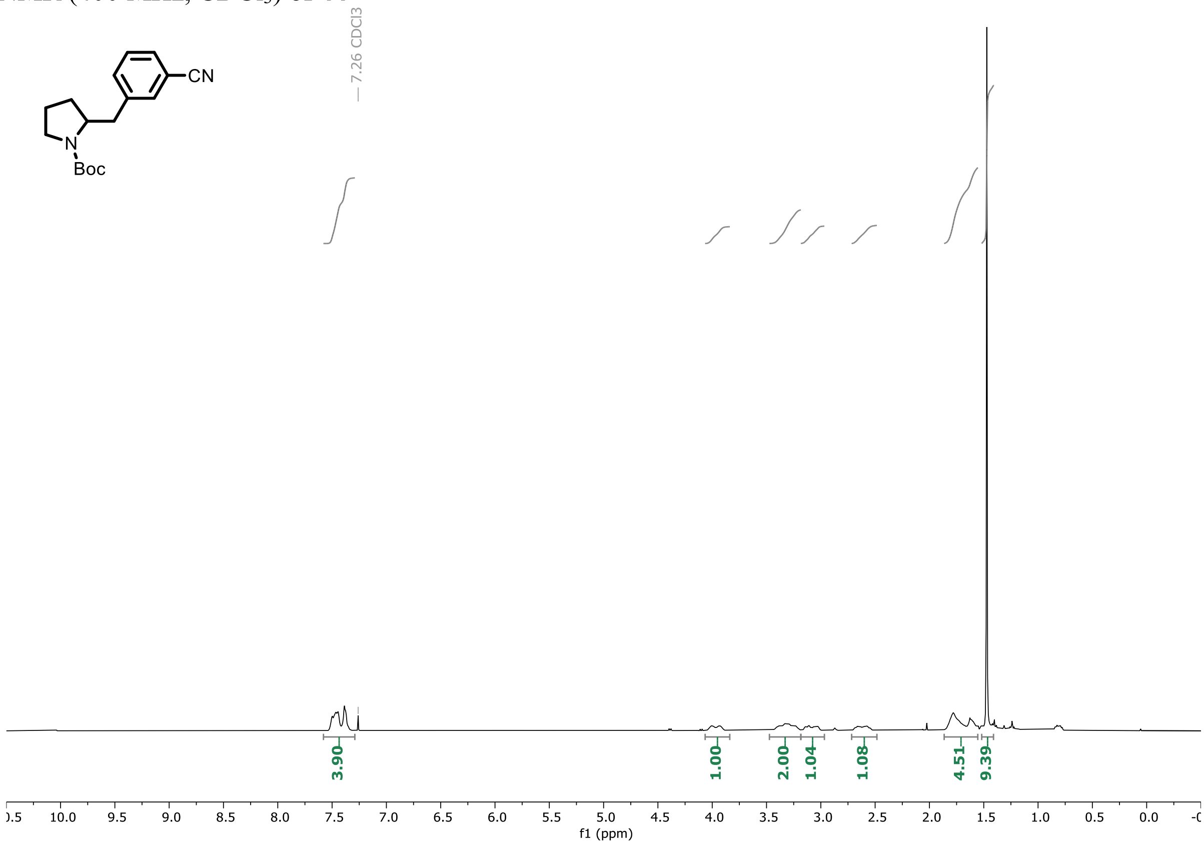

^{13}C NMR (101 MHz, $CDCl_3$) of **44**

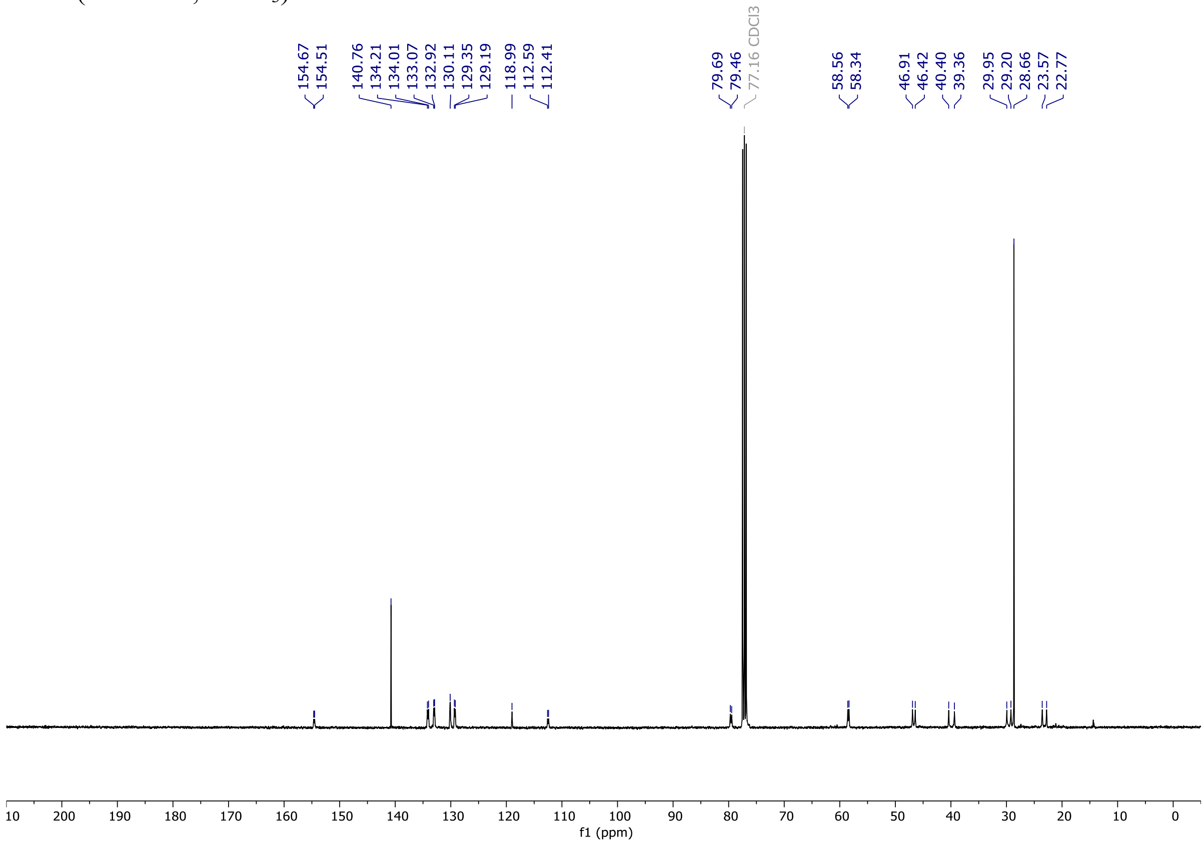

^{1}H NMR (400 MHz, $CDCl_3$) of **45**

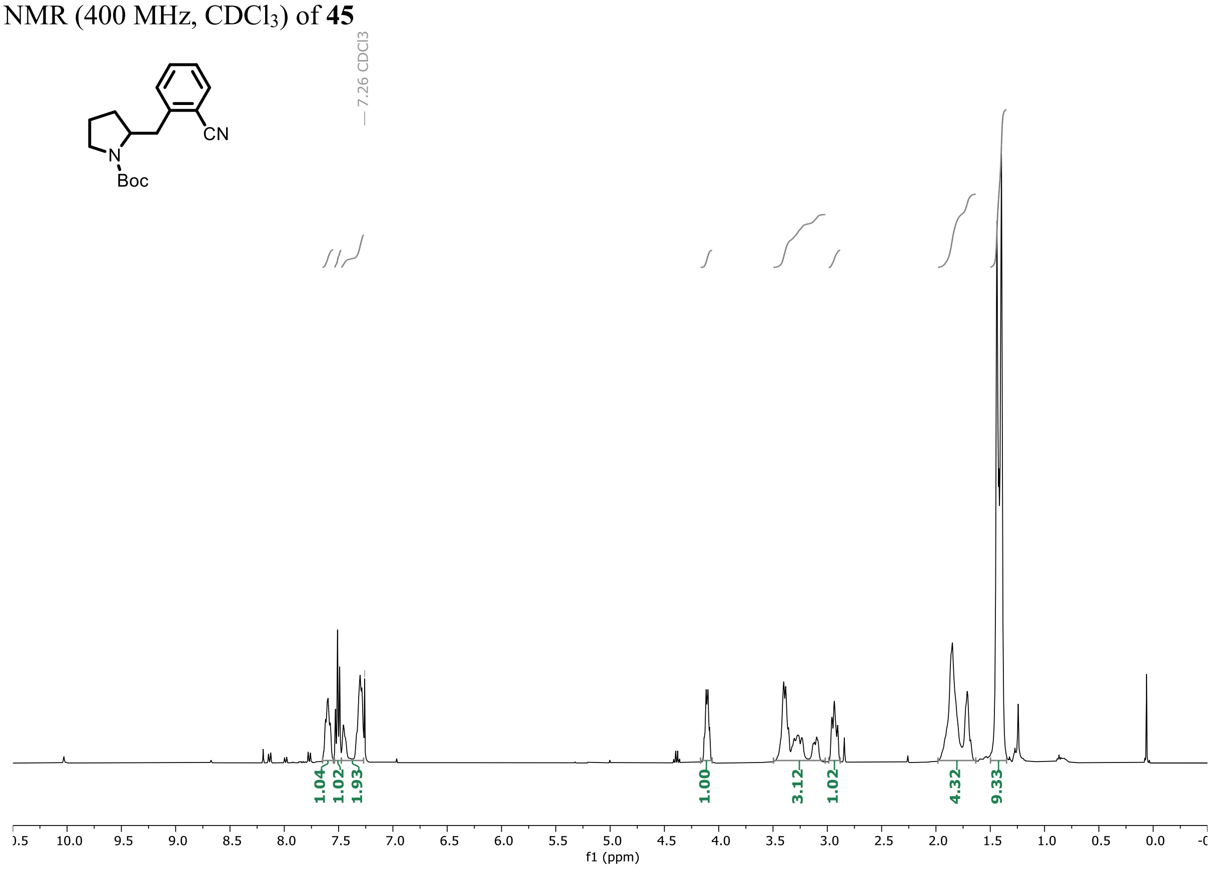

^{13}C NMR (101 MHz, $CDCl_3$) of **45**

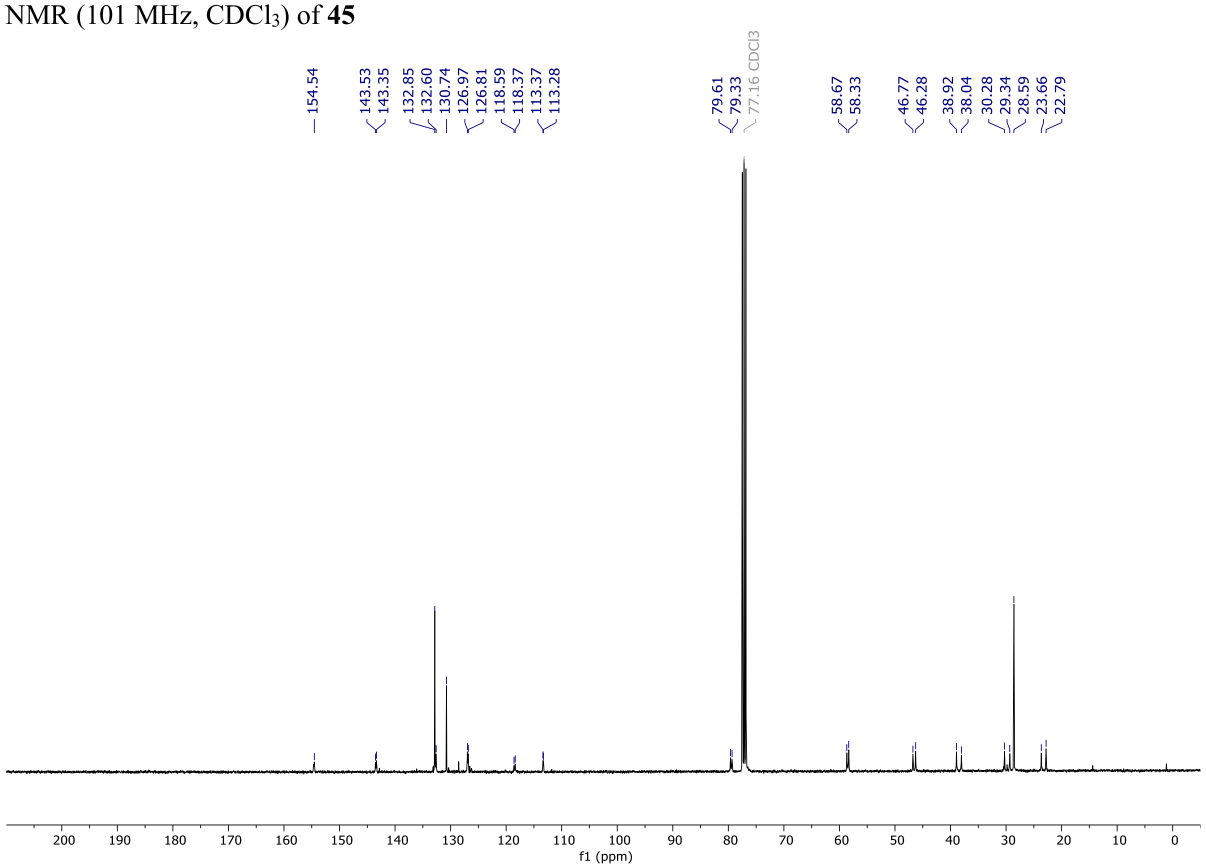

^{1}H NMR (400 MHz,$CDCl_3$) of **46**

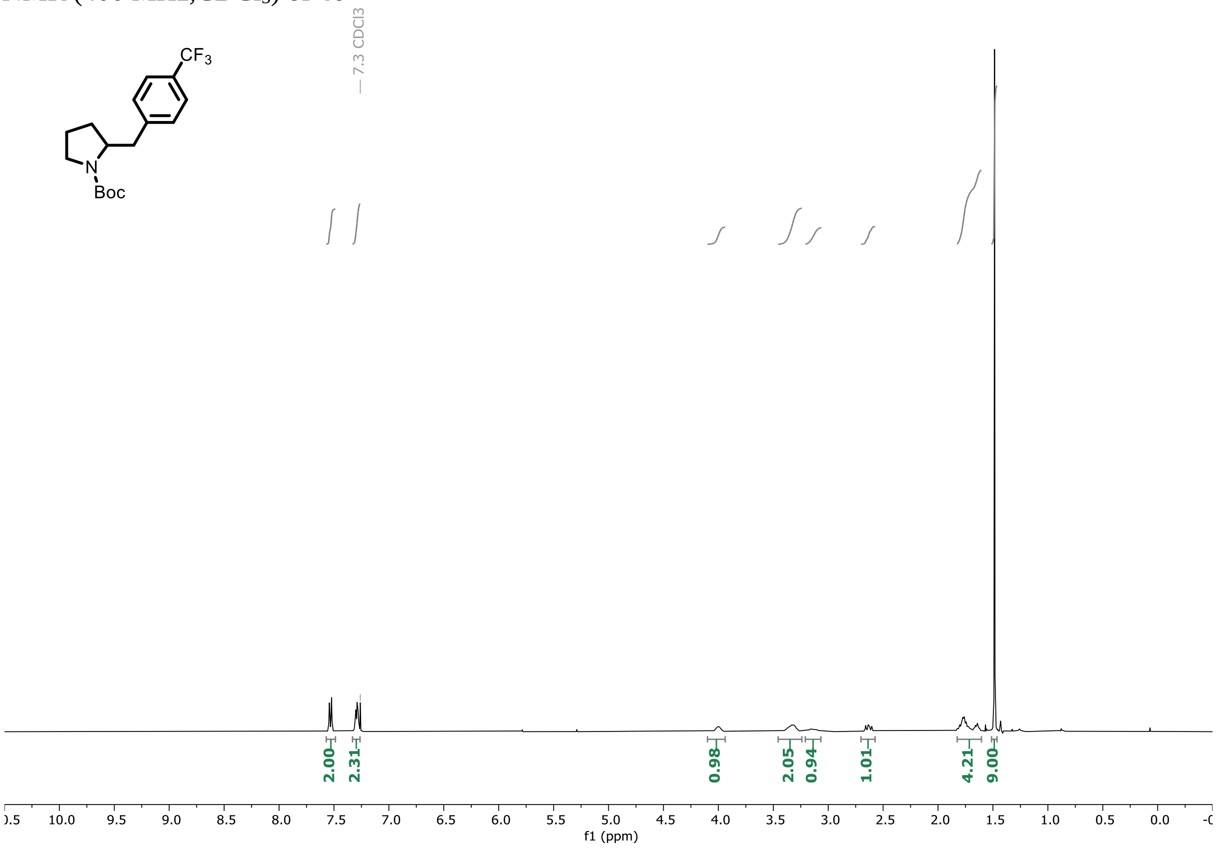

^{13}C NMR (101 MHz, $CDCl_3$) of **46**

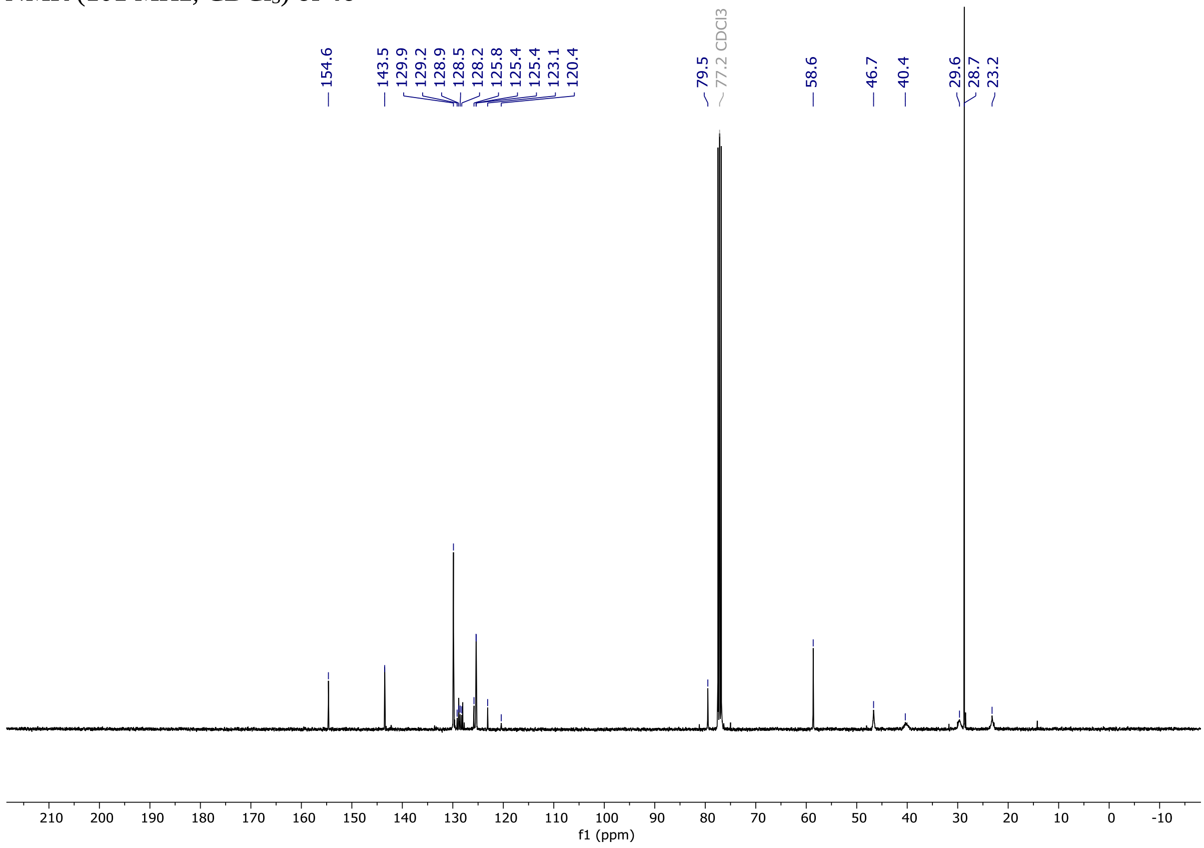

¹⁹F NMR (282 MHz, $CDCl_3$) of **46**

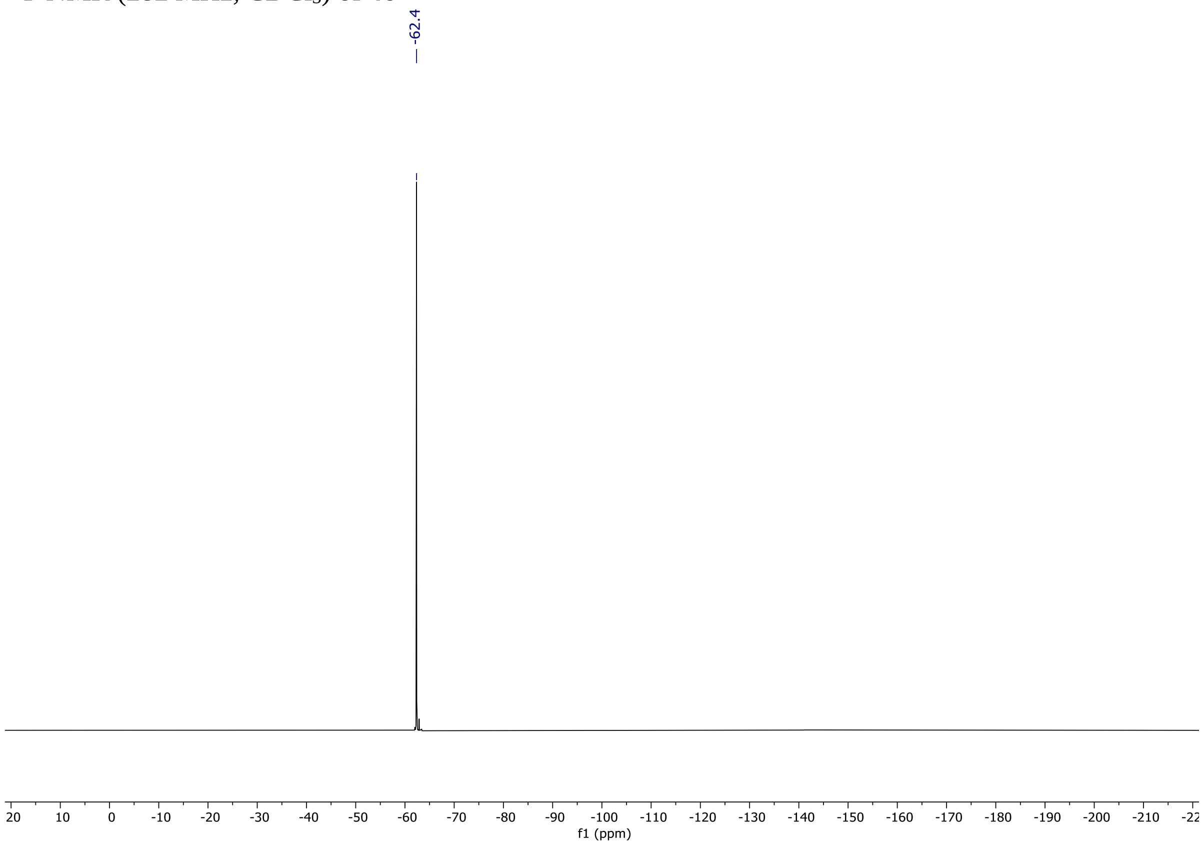

^{1}H NMR (400 MHz, $CDCl_3$) of **47**

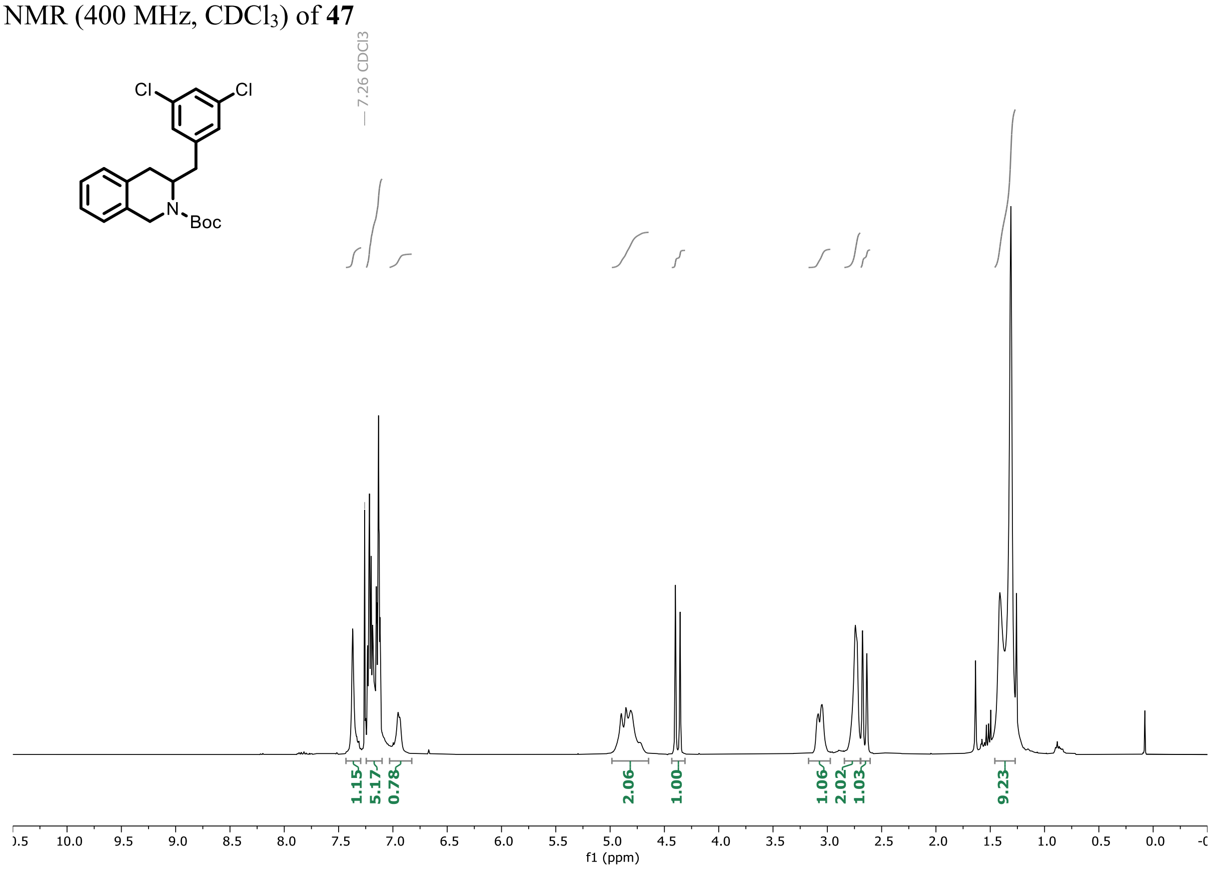

^{13}C NMR (101 MHz, $CDCl_3$) of **47**

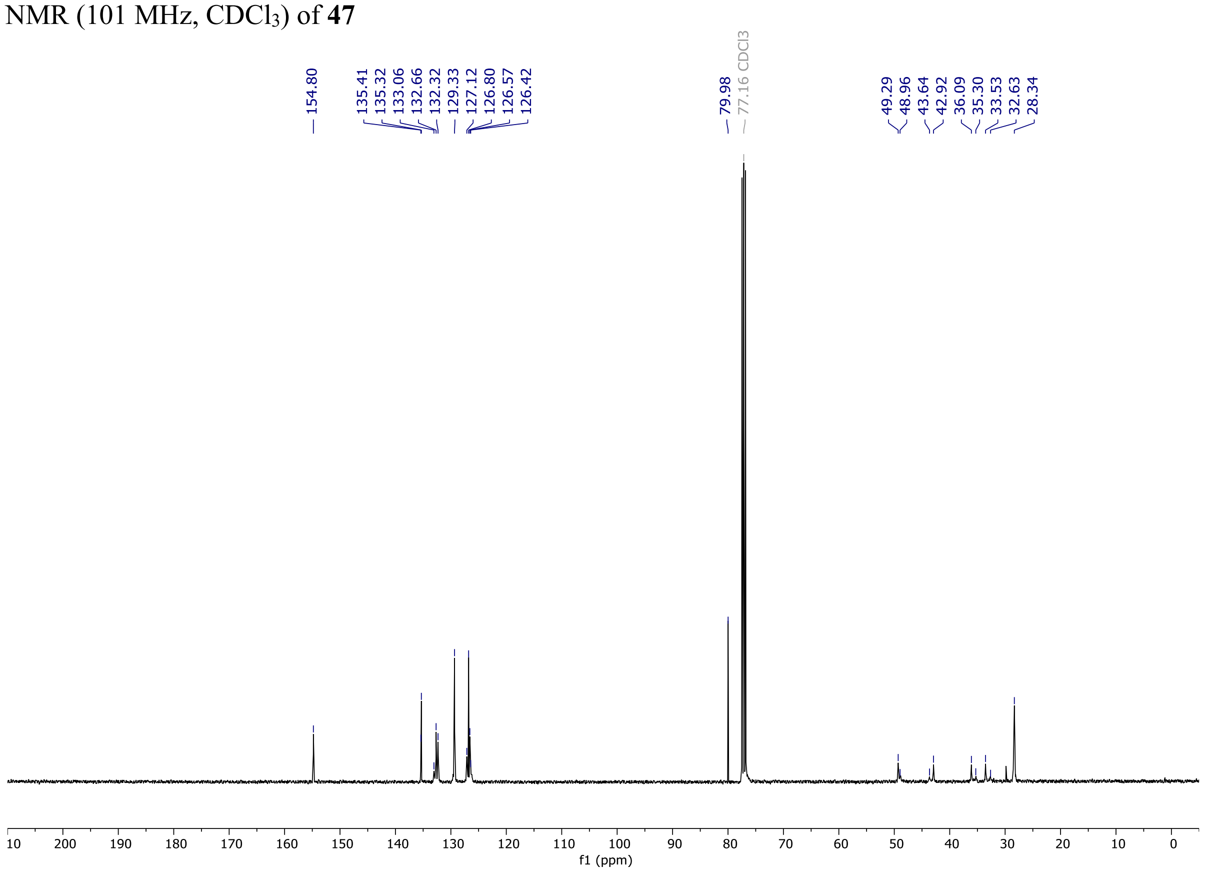

^{1}H NMR (400 MHz,CDCl$_3$) of **48**

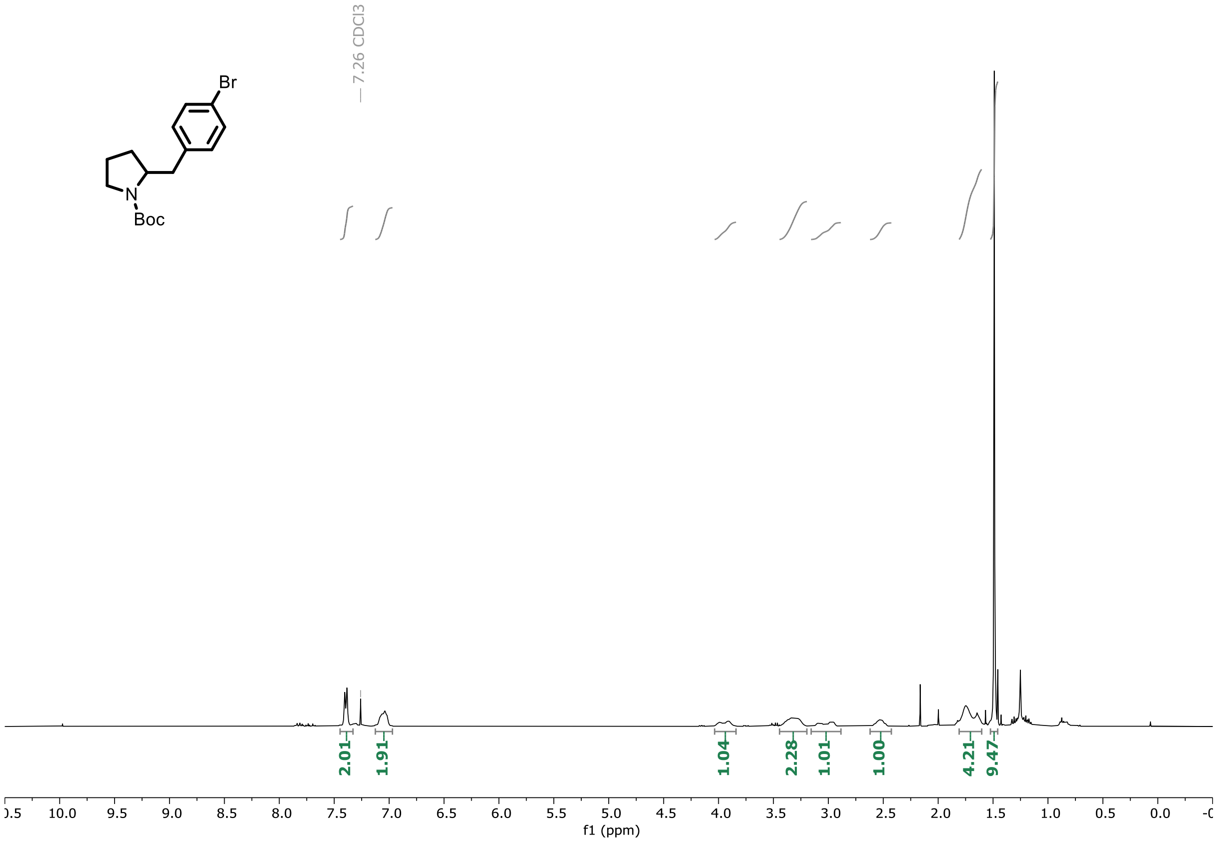

^{13}C NMR (101 MHz, CDCl$_3$) of **48**

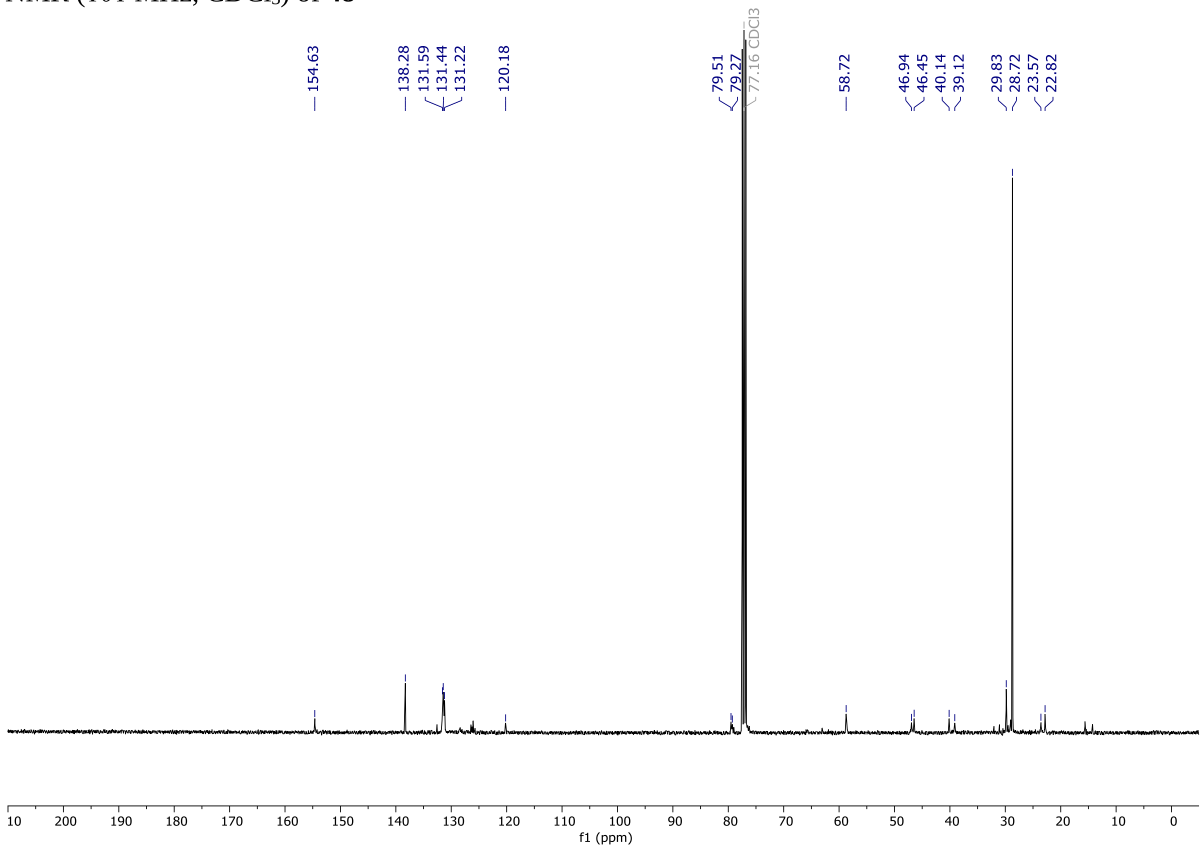

^{1}H NMR (500 MHz,CDCl$_3$) of **49**

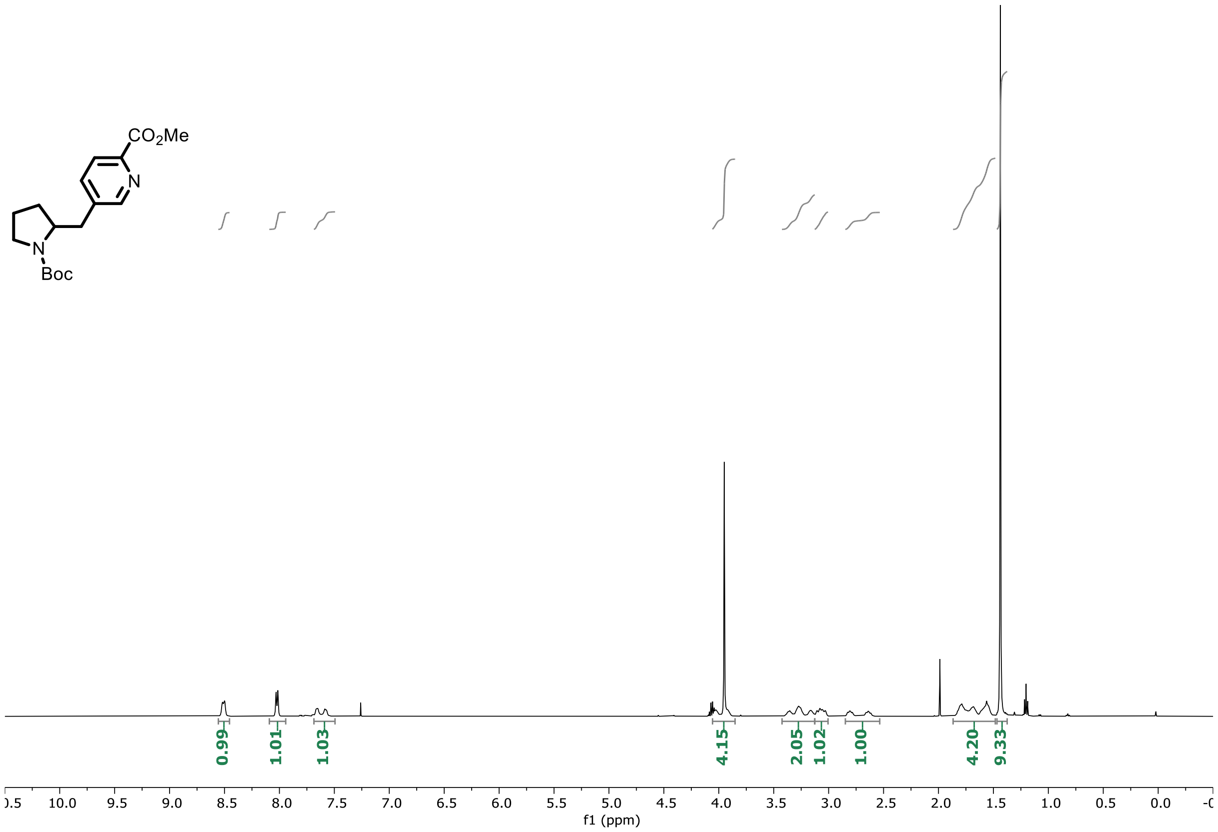

^{13}C NMR (126 MHz, CDCl$_3$) of **49**

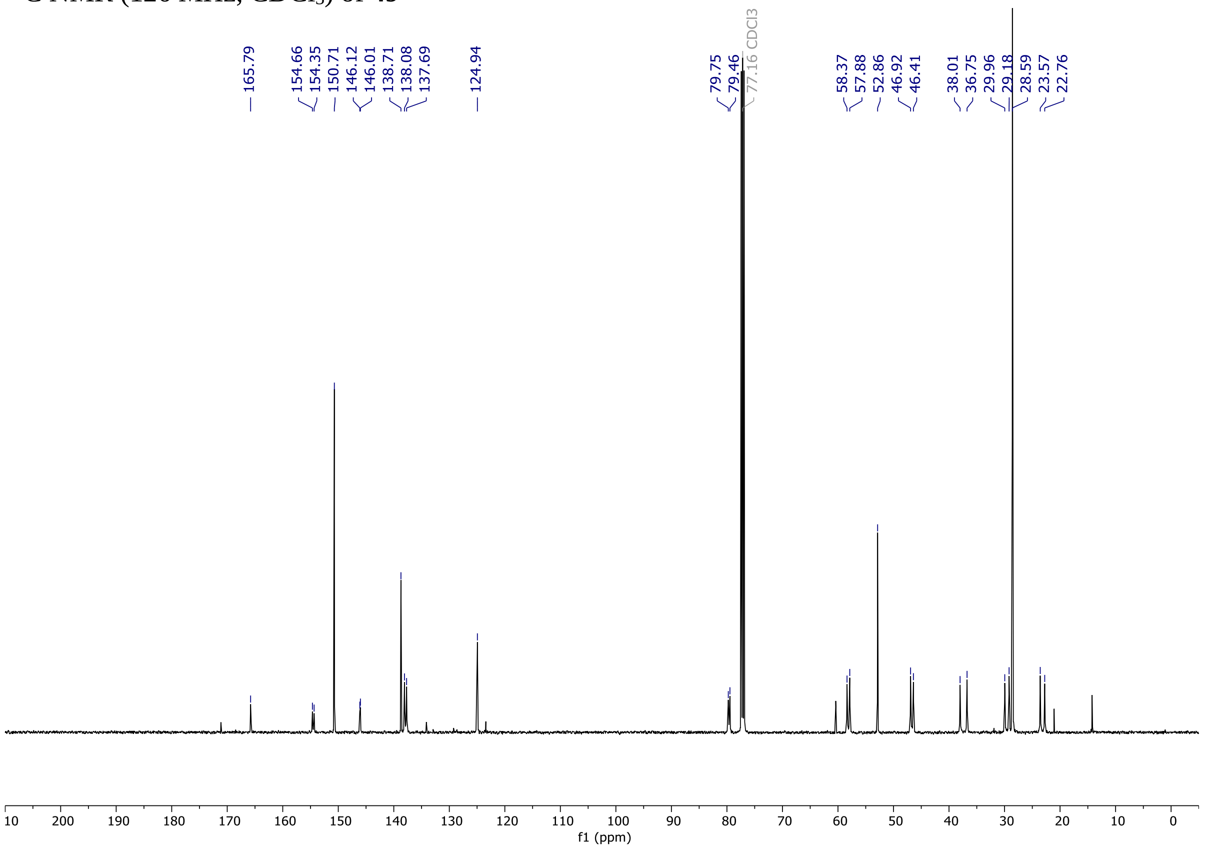

^{1}H NMR (400 MHz,CDCl$_3$) of **50**

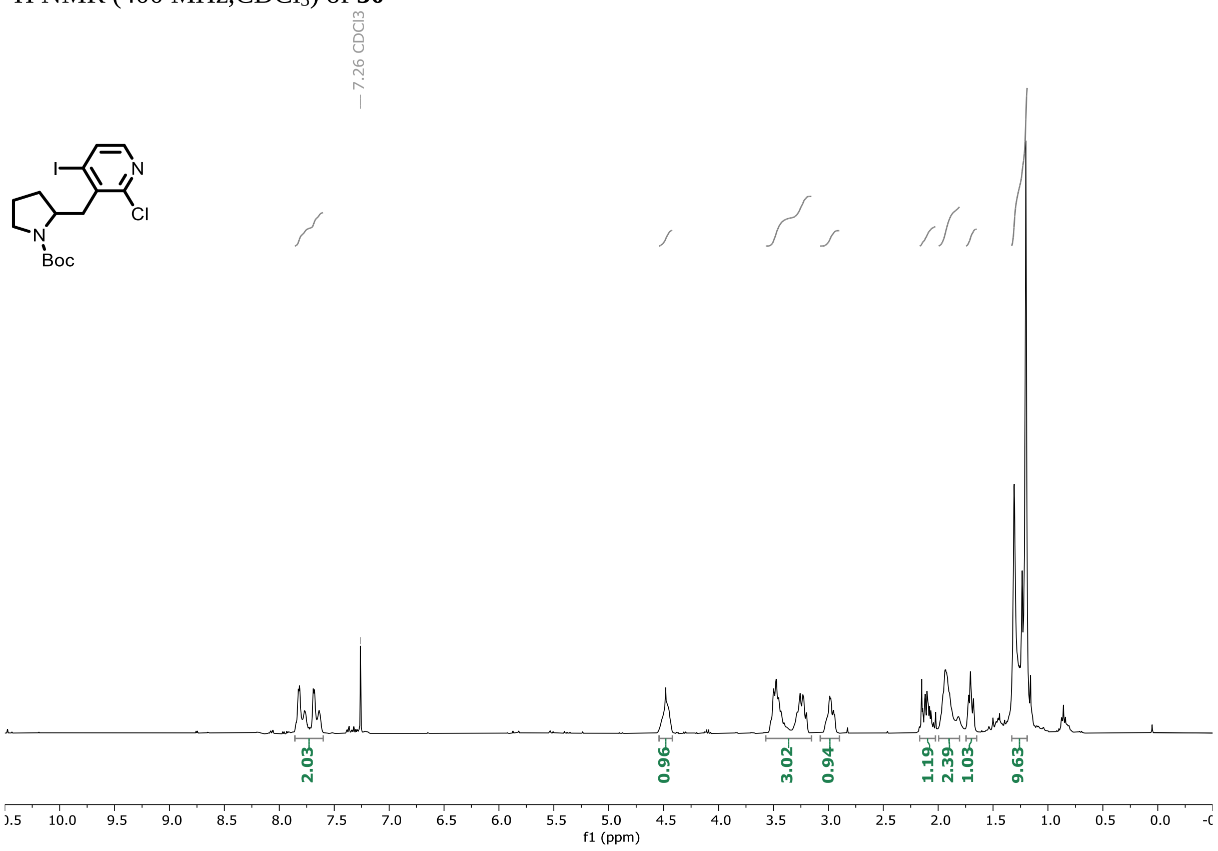

^{13}C NMR (101 MHz, CDCl$_3$) of **50**

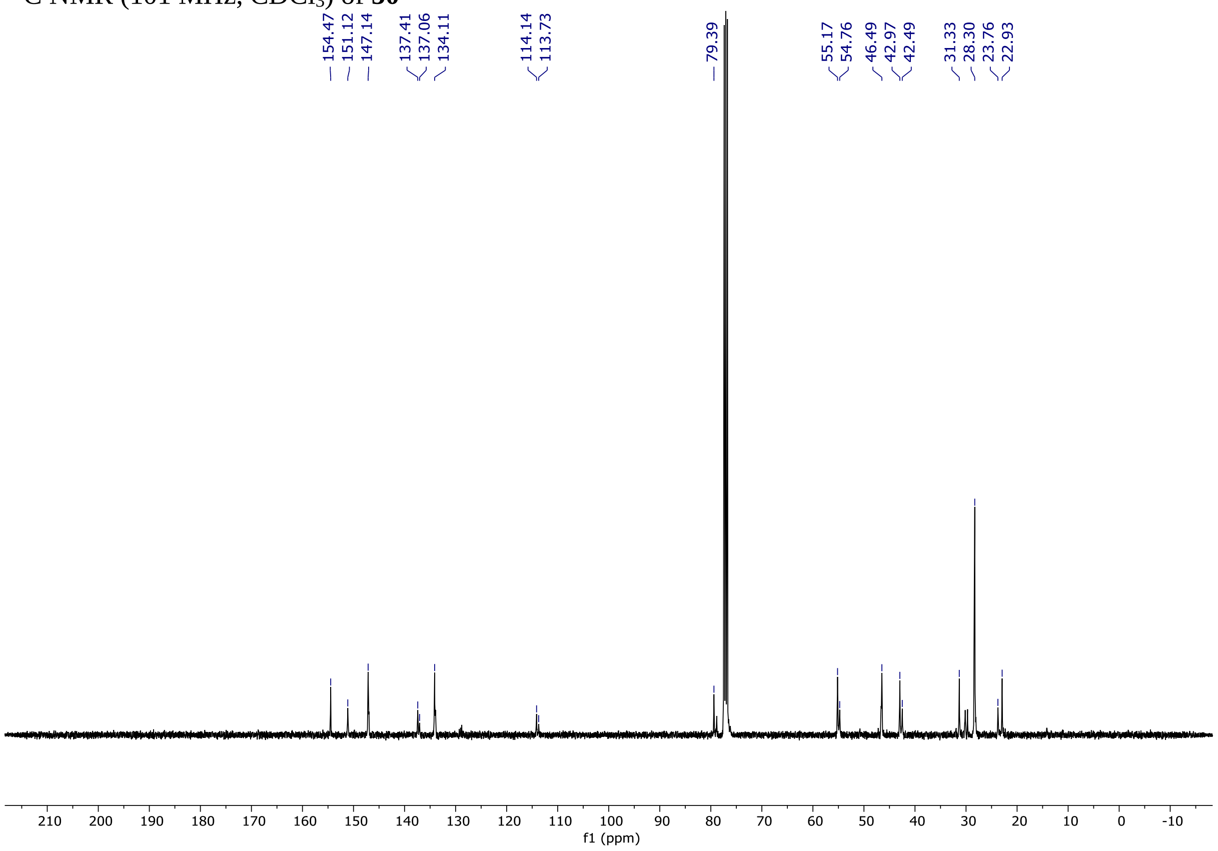

^{1}H NMR (400 MHz, $CDCl_3$) of **51**

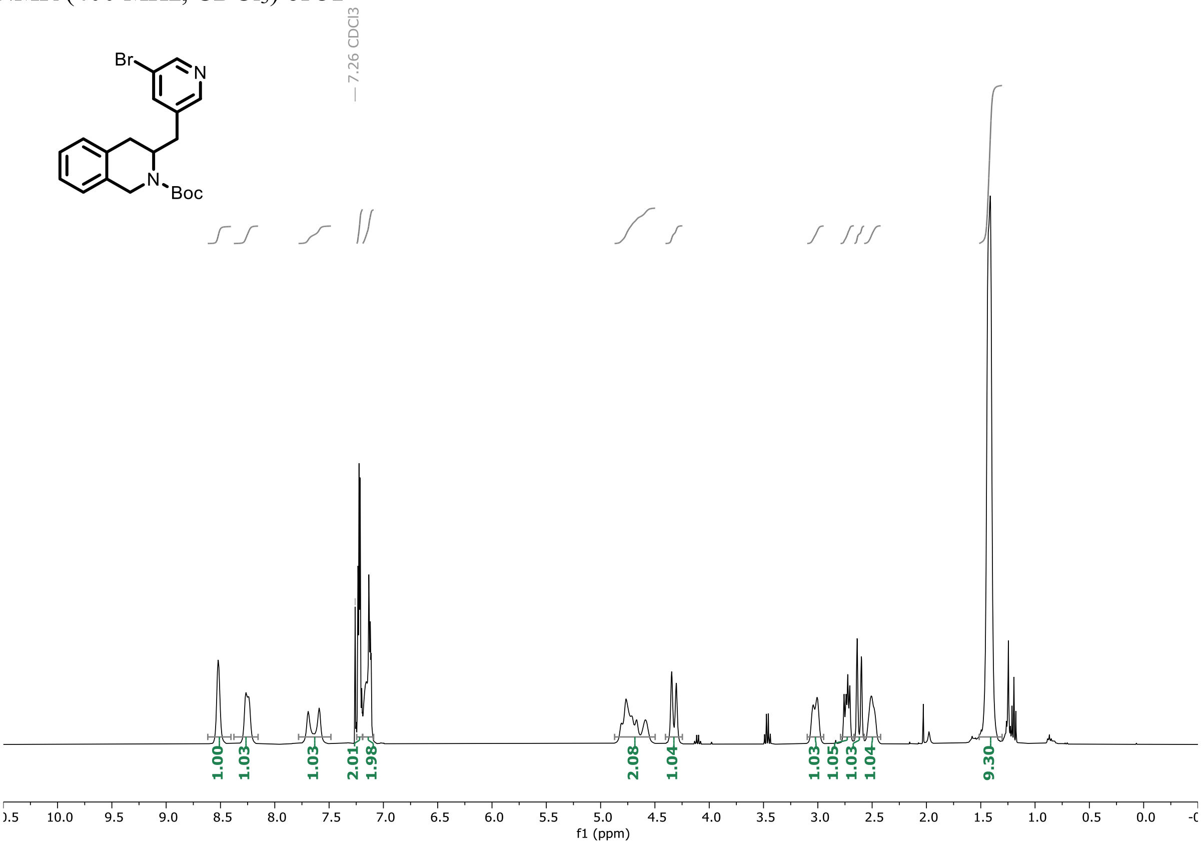

^{13}C NMR (101 MHz, $CDCl_3$) of **51**

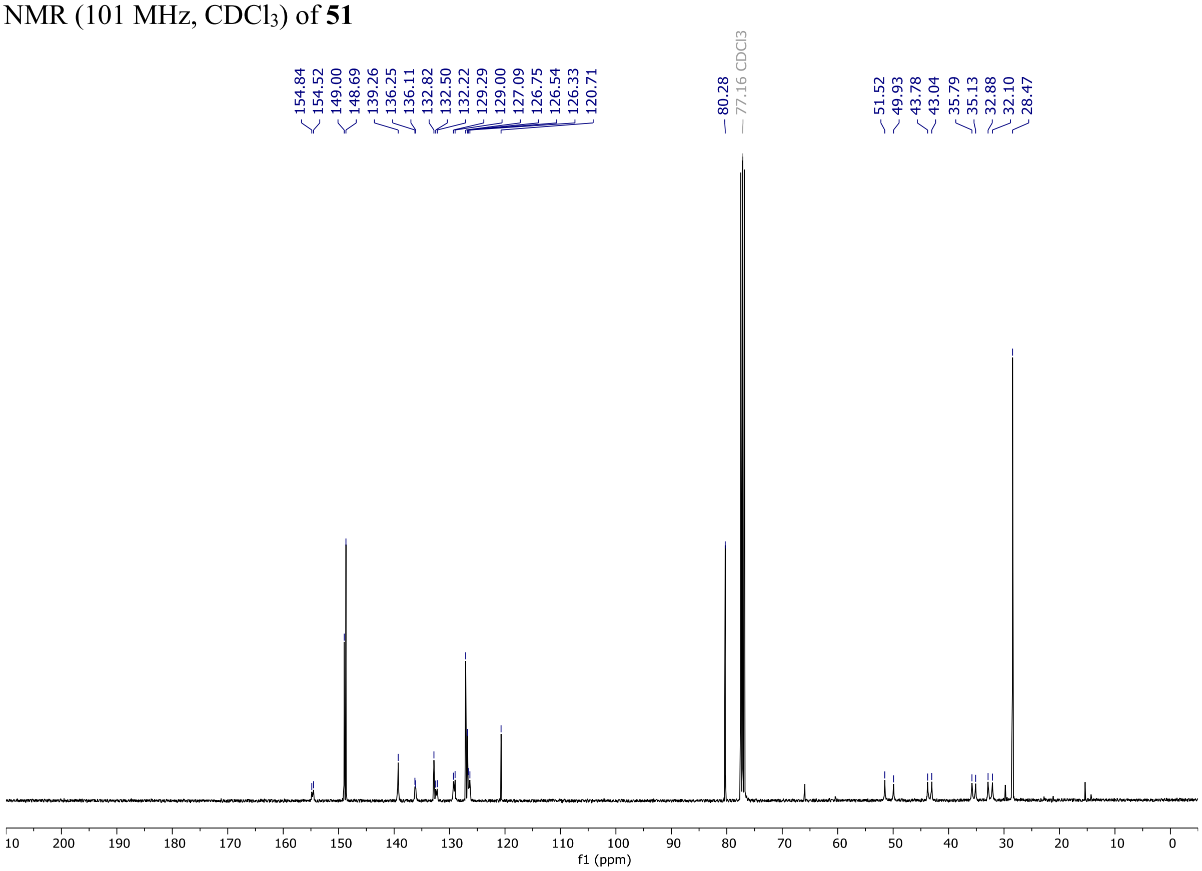

^{1}H NMR (400 MHz,CDCl$_3$) of **52**

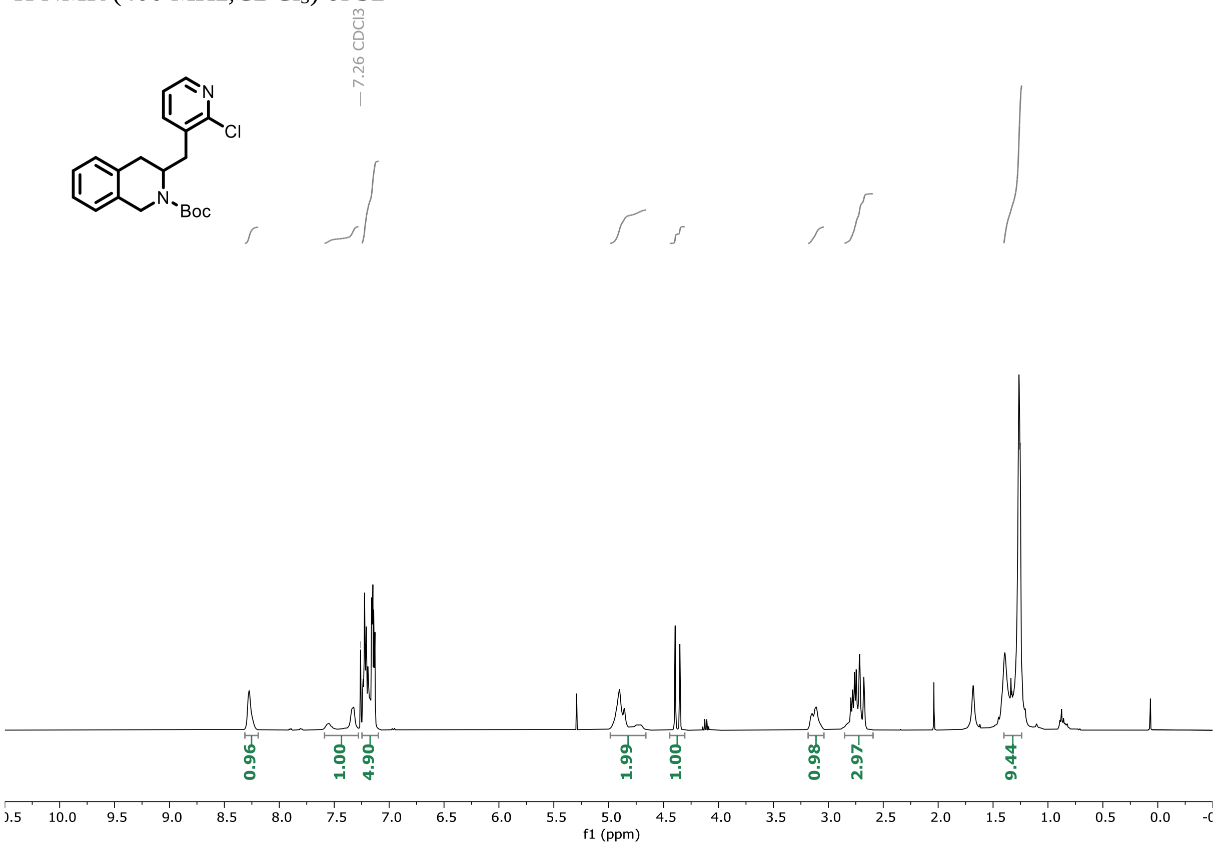

^{13}C NMR (101 MHz, CDCl$_3$) of **52**

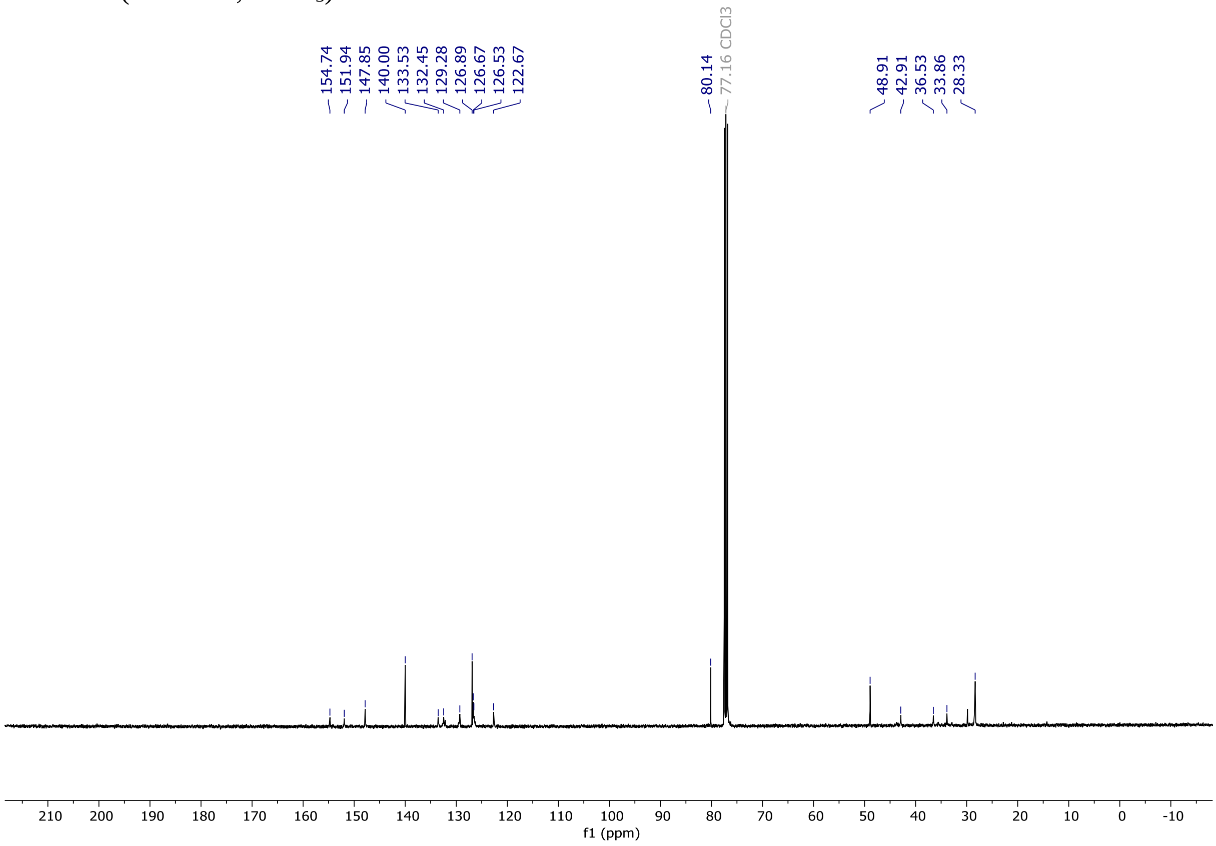

^{1}H NMR (400 MHz,$CDCl_3$) of **53**

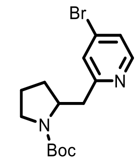

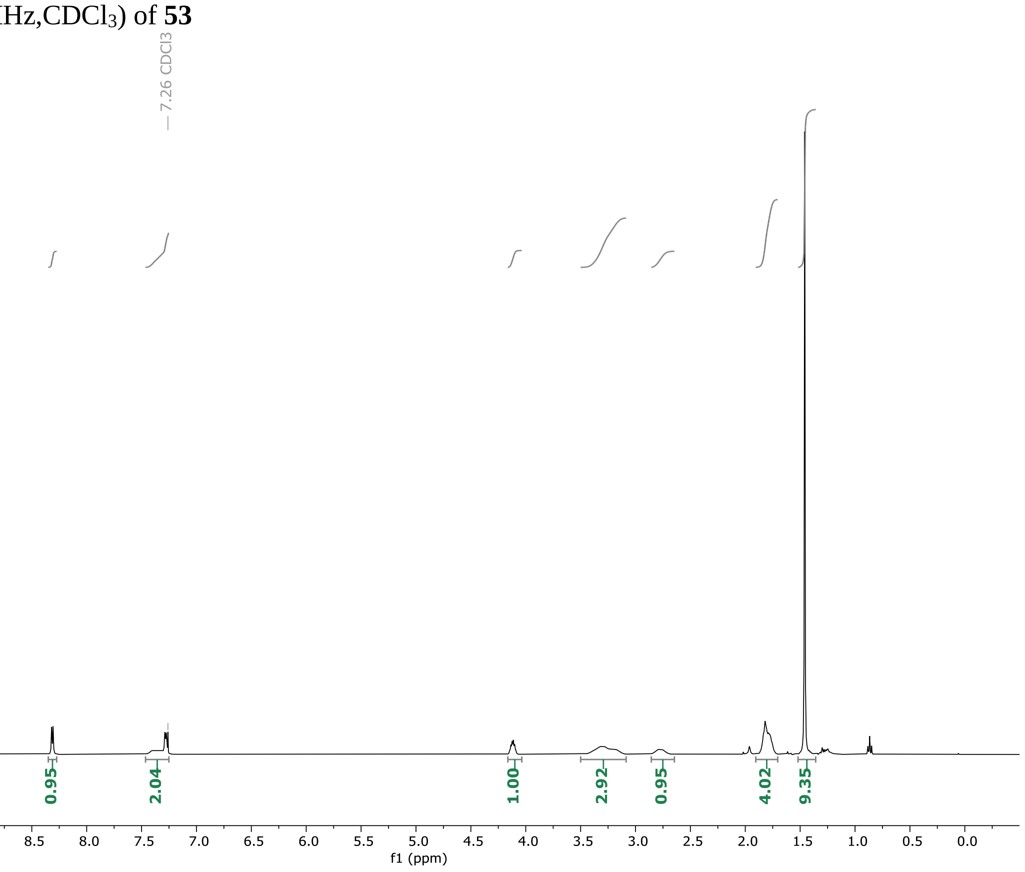

^{13}C NMR (101 MHz, $CDCl_3$) of **53**

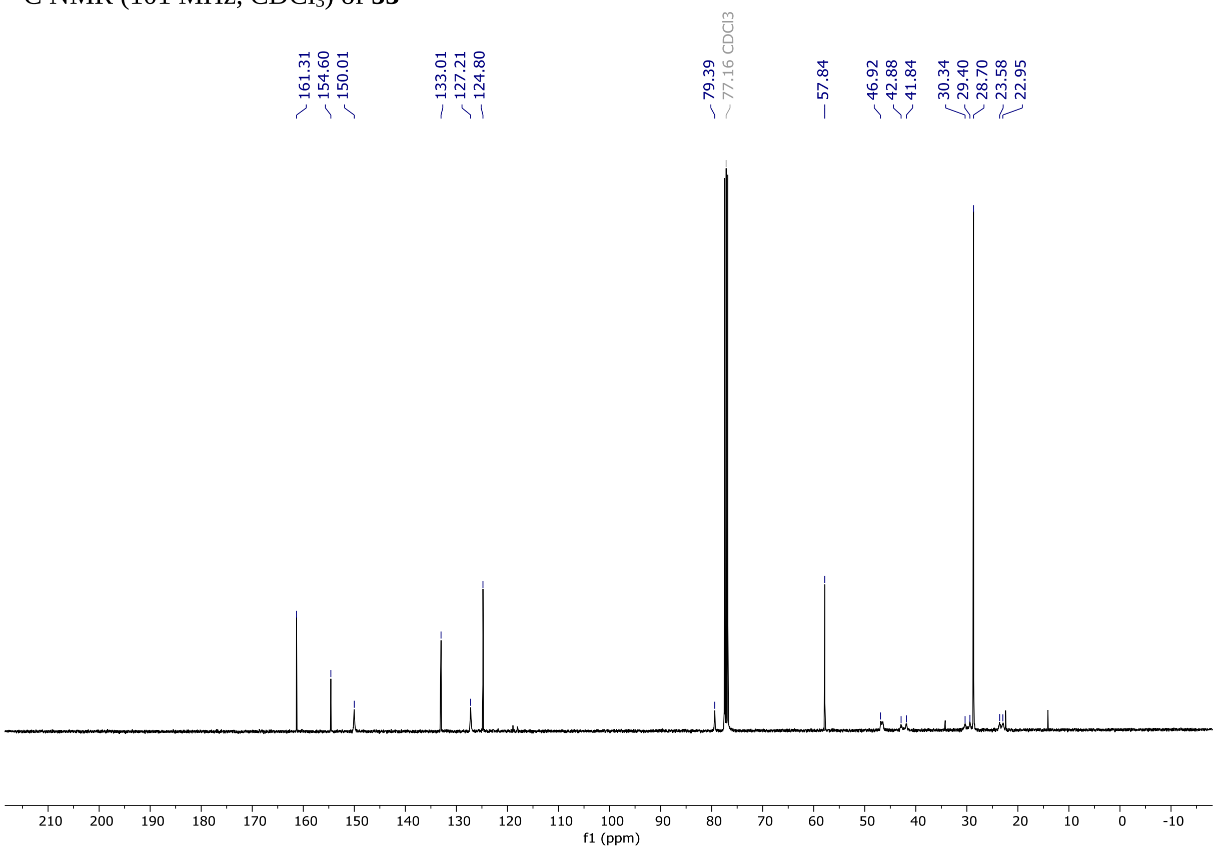

^{1}H NMR (400 MHz, $CDCl_3$) of **54**

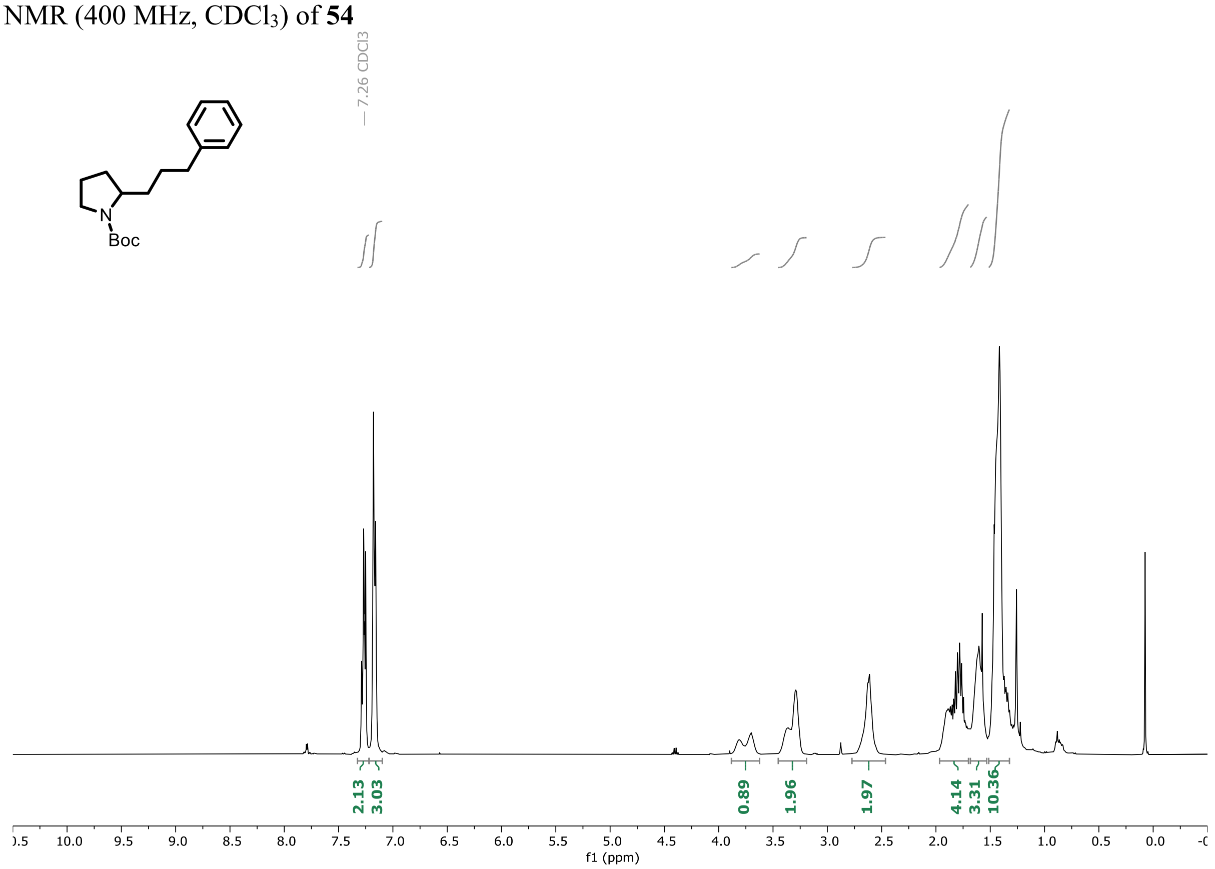

^{13}C NMR (101 MHz, $CDCl_3$) of **54**

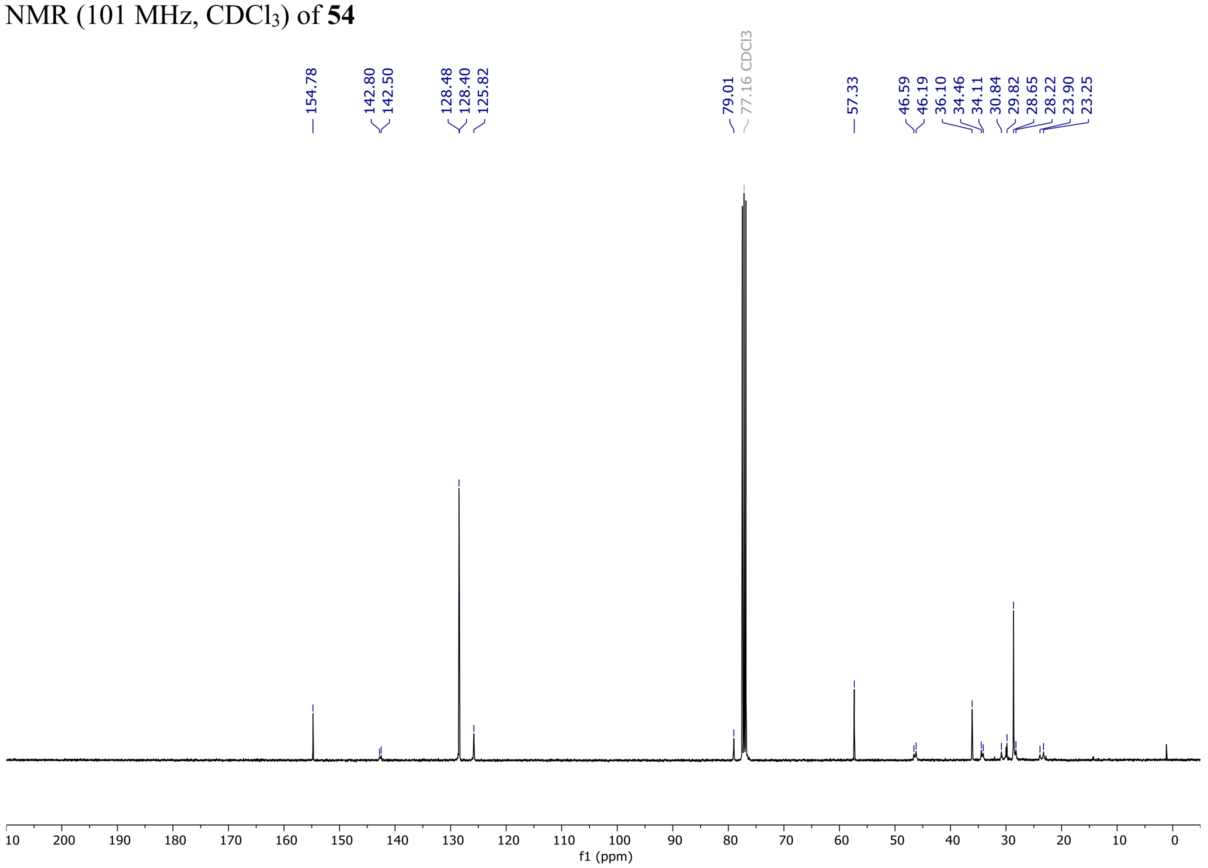

^{1}H NMR (400 MHz, $CDCl_3$) of **55**

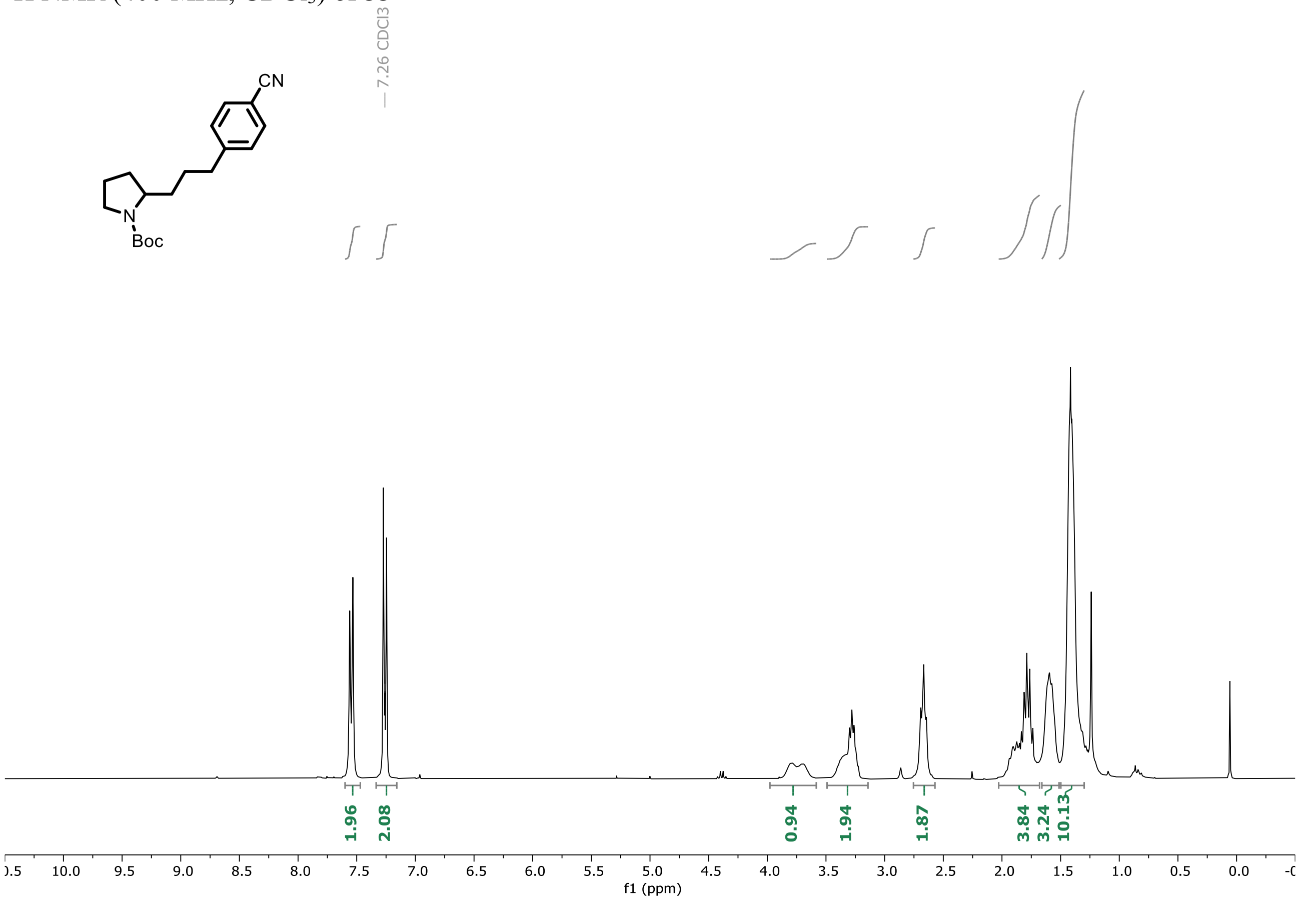

^{13}C NMR (101 MHz, $CDCl_3$) of **55**

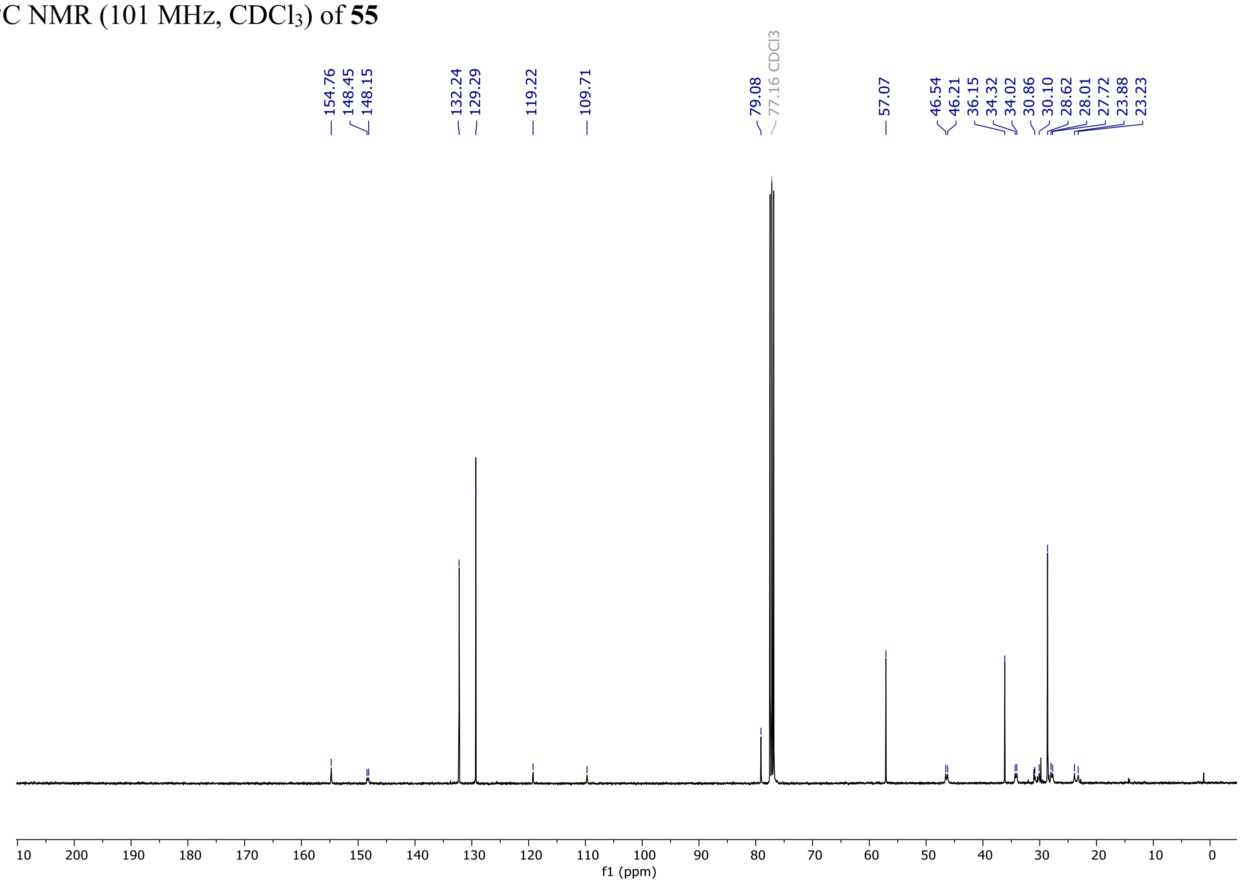

^{1}H NMR (400 MHz, $CDCl_3$) of **56**

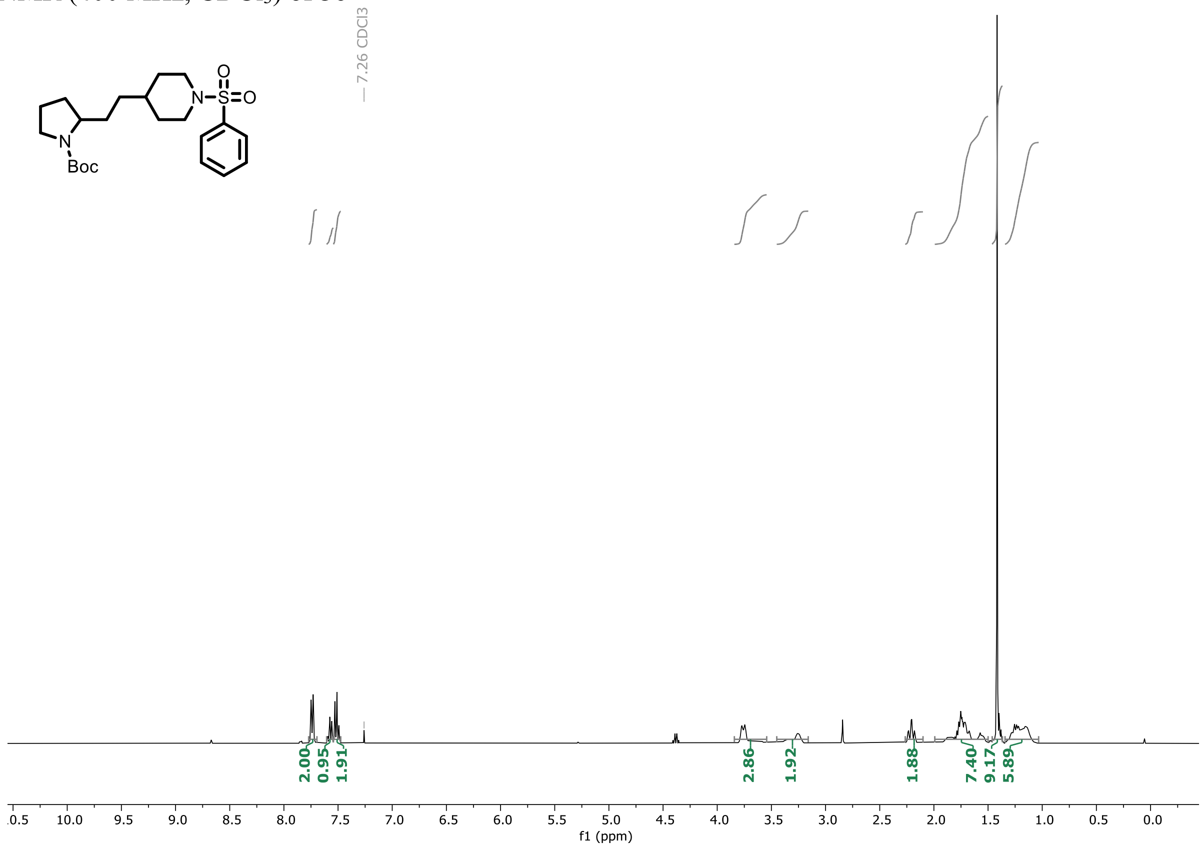

^{13}C NMR (101 MHz, $CDCl_3$) of **56**

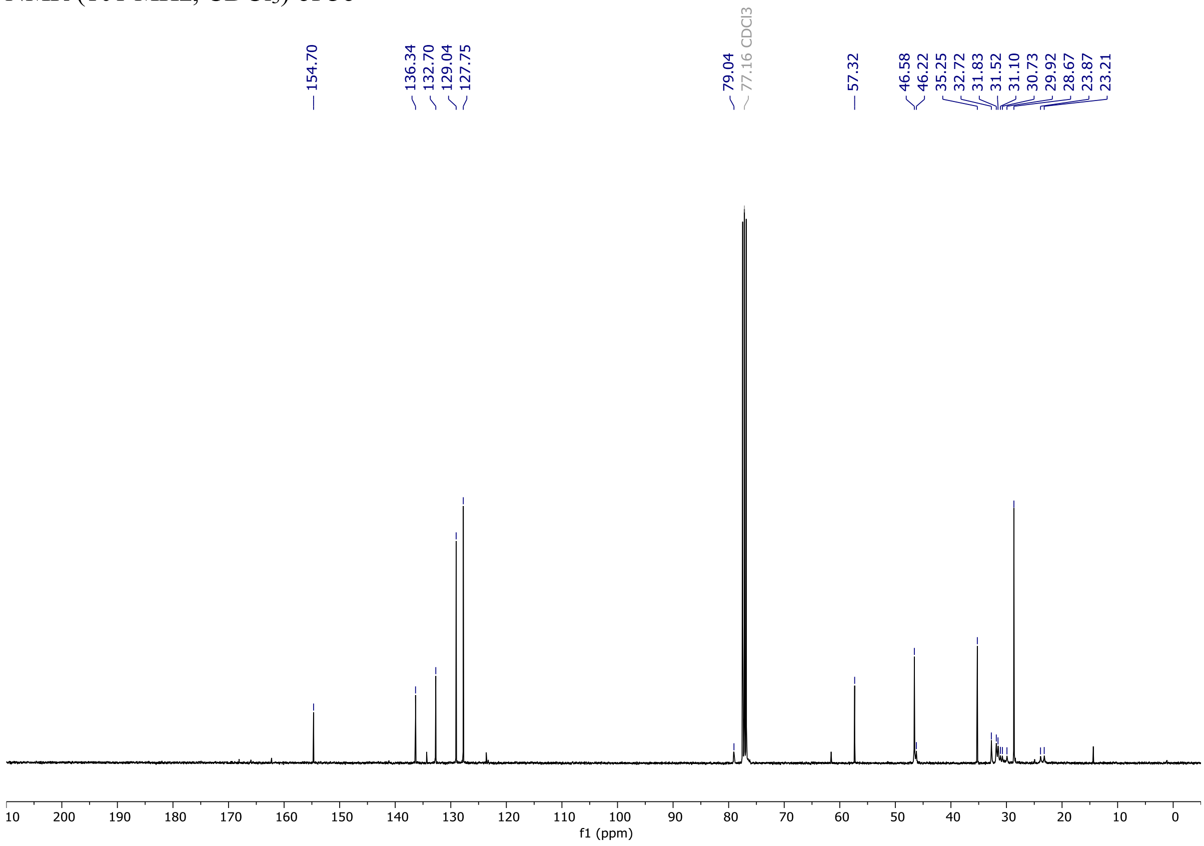

^{1}H NMR (400 MHz,CDCl$_3$) of **57**

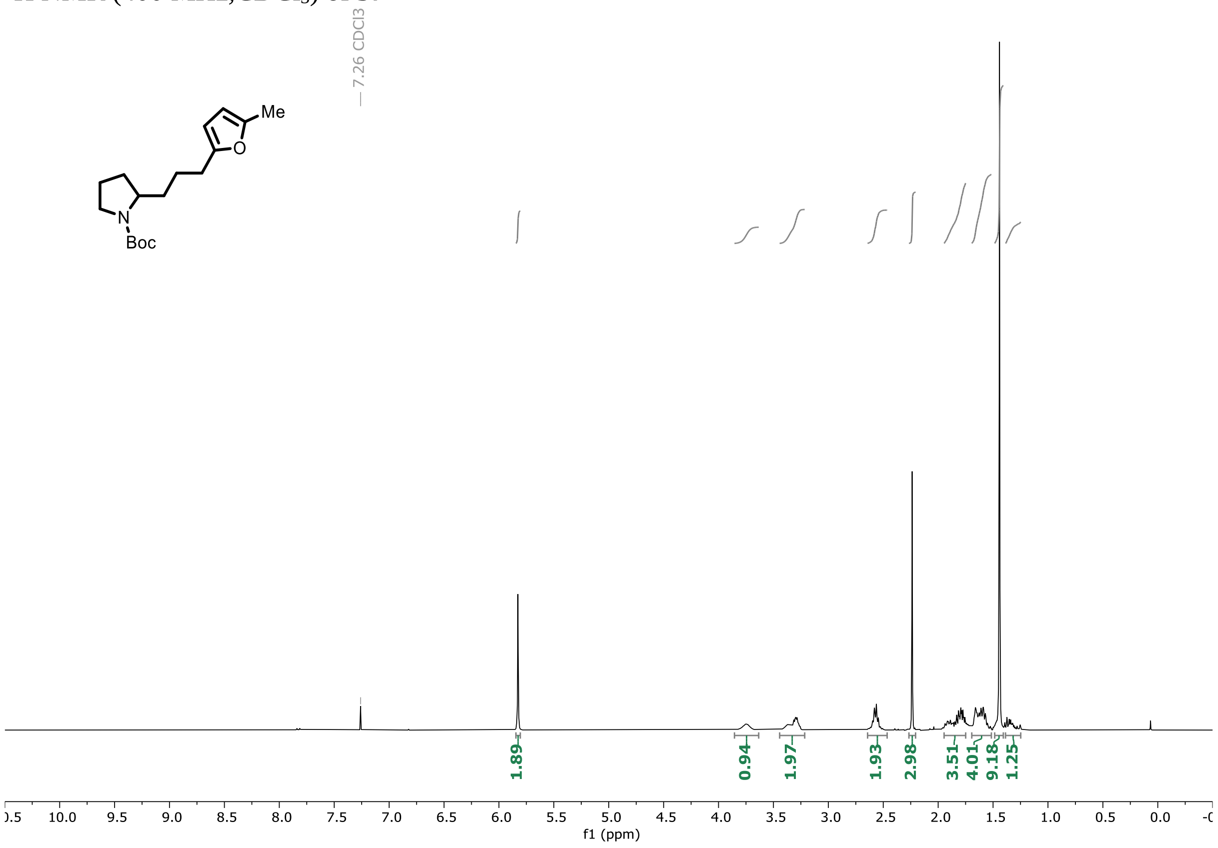

^{13}C NMR (101 MHz, CDCl$_3$) of **57**

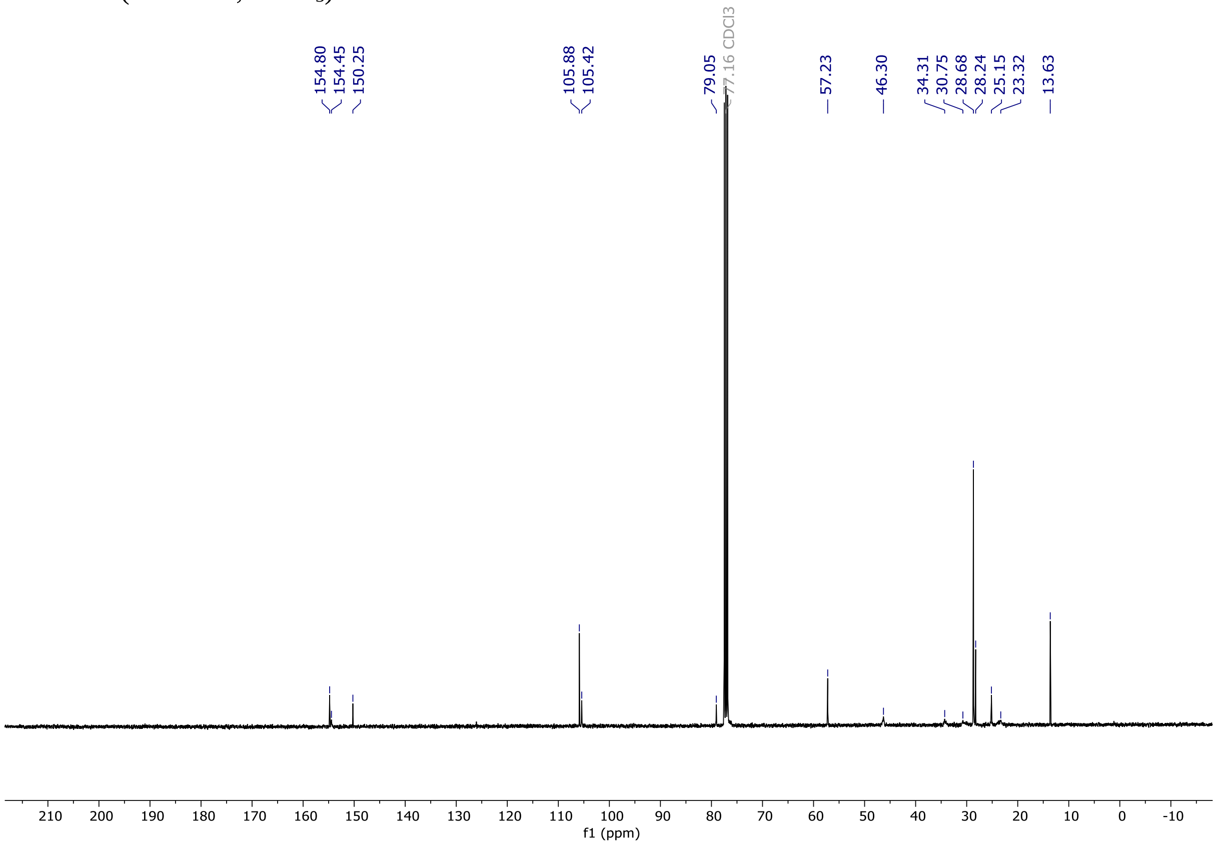

^{1}H NMR (400 MHz, $CDCl_3$) of **58**

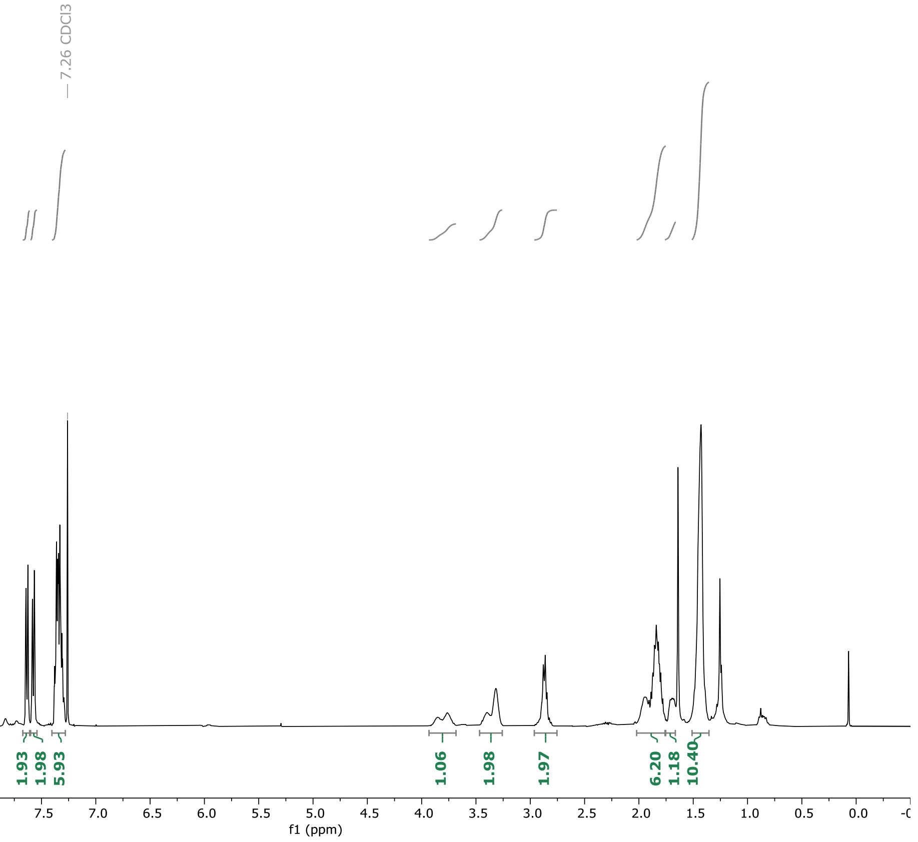

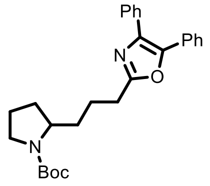

^{13}C NMR (101 MHz, $CDCl_3$) of **58**

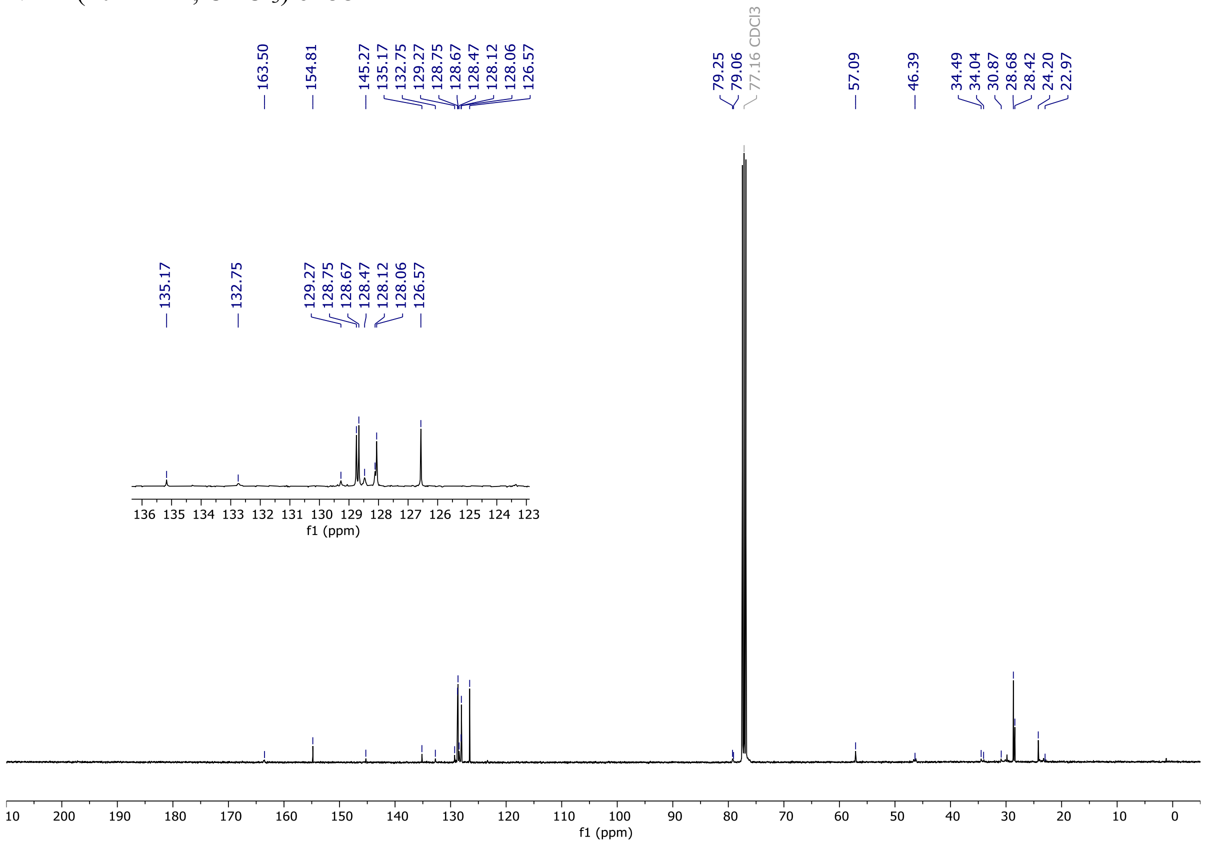

17. References

[1] M. Garreau, F. Le Vaillant, J. Waser, *Angew. Chem. Int. Ed.* **2019**, *58*, 8182–8186.
[2] H. E. Askey, J. D. Grayson, J. D. Tibbetts, J. C. Turner-Dore, J. M. Holmes, G. Kociok-Kohn, G. L. Wrigley, A. J. Cresswell, *J. Am. Chem. Soc.* **2021**, *143*, 15936–15945.
[3] Q. Wang, J. A. May, *Org. Lett.* **2020**, *22*, 9579–9584.
[4] A. Pulcinella, S. Bonciolini, F. Lukas, A. Sorato, T. Noël, *Angew. Chem. Int. Ed.* **2023**, *62*, e202215374.
[5] Z. Bao, M. Huang, Y. Xu, X. Zhang, Y. Wu, J. Wang, *Angew. Chem. Int. Ed.* **2023**, *62*, e202216356.
[6] R. Chowdhury, Z. Yu, M. L. Tong, S. V. Kohlhepp, X. Yin, A. Mendoza, *J. Am. Chem. Soc.* **2020**, *142*, 20143–20151.
[7] G. Pratsch, G. L. Lackner, L. E. Overman, *J. Org. Chem.* **2015**, *80*, 6025–6036.
[8] A. Rana, B. K. Malviya, D. K. Jaiswal, P. Srihari, A. K. Singh, *Green Chem.* **2022**, *24*, 4794–4799.
[9] D. Heine, T. Bretschneider, S. Sundaram, C. Hertweck, *Angew. Chem. Int. Ed.* **2014**, *53*, 11645–11649.
[10] D. Vourloumis, M. Takahashi, K. B. Simonsen, B. K. Ayida, S. Barluenga, G. C. Winters, T. Hermann, *Tetrahedron Lett.* **2003**, *44*, 2807–2811.
[11] A. C. Breman, A. Ruiz-Olalla, J. H. van Maarseveen, S. Ingemann, H. Hiemstra, *Eur. J. Org. Chem.* **2014**, *2014*, 7413–7425.
[12] Q. Cao, J. L. Howard, E. Wheatley, D. L. Browne, *Angew. Chem. Int. Ed.* **2018**, *57*, 11339–11343.
[13] T. Wennekes, R. J. B. H. N. van den Berg, K. M. Bonger, W. E. Donker-Koopman, A. Ghisaidoobe, G. A. van der Marel, A. Strijland, J. M. F. G. Aerts, H. S. Overkleeft, *Tetrahedron Asymmetry* **2009**, *20*, 836–846.
[14] L. Angelini, J. Davies, M. Simonetti, L. Malet Sanz, N. S. Sheikh, D. Leonori, *Angew. Chem. Int. Ed.* **2019**, *58*, 5003–5007.
[15] E. López, C. van Melis, R. Martín, A. Petti, A. de la Hoz, Á. Díaz-Ortíz, A. P. Dobbs, K. Lam, J. Alcázar, *Adv. Synth. Catal.* **2021**, *363*, 4521–4525.
[16] B. Varga, Z. Gonda, B. L. Tóth, A. Kotschy, Z. Novák, *Eur. J. Org. Chem.* **2020**, *2020*, 1466–1471.
[17] Z. Zhang, T. Cernak, *Angew. Chem. Int. Ed.* **2021**, *60*, 27293–27298.
[18] Y. Gong, L. Su, Z. Zhu, Y. Ye, H. Gong, *Angew. Chem. Int. Ed.* **2022**, *61*, e202201662.
